# Engineering Central Substitutions in Heptamethine Dyes for Improved Fluorophore Performance

**DOI:** 10.1021/jacsau.4c00343

**Published:** 2024-08-08

**Authors:** Lei Guo, Meek Yang, Bin Dong, Seth Lewman, Alex Van Horn, Shang Jia

**Affiliations:** † Department of Civil Engineering, University of Arkansas, Fayetteville, Fayetteville, Arkansas 72701, United States; ‡ Department of Chemistry and Biochemistry, University of Arkansas, Fayetteville, Fayetteville, Arkansas 72701, United States

**Keywords:** polymethine dye, cyanine, water solubility, aggregation, stability, fluorescence imaging

## Abstract

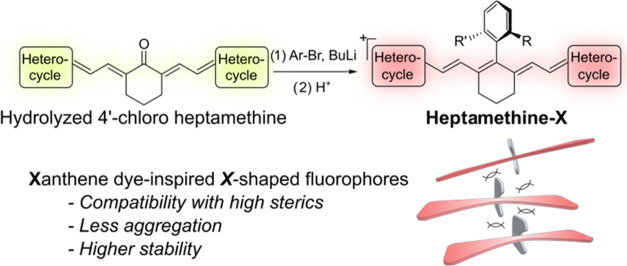

As a major family of red-shifted fluorophores that operate beyond visible light, polymethine dyes are pivotal in light-based biological techniques. However, methods for tuning this kind of fluorophores by structural modification remain restricted to bottom-up synthesis and modification using coupling or nucleophilic substitutions. In this study, we introduce a two-step, late-stage functionalization process for heptamethine dyes. This process enables the substitution of the central chlorine atom in the commonly used 4′-chloro heptamethine scaffold with various aryl groups using aryllithium reagents. This method borrows the building block and designs from the xanthene dye community and offers a mild and convenient way for the diversification of heptamethine fluorophores. Notably, this efficient conversion allows for the synthesis of heptamethine-X, the heptamethine scaffold with two ortho-substituents on the 4′-aryl modification, which brings enhanced stability and reduced aggregation to the fluorophore. We showcase the utility of this method by a facile synthesis of a fluorogenic, membrane-localizing fluorophore that outperforms its commercial counterparts with a significantly higher brightness and contrast. Overall, this method establishes the synthetic similarities between polymethine and xanthene fluorophores and provides a versatile and feasible toolbox for future optimizing heptamethine fluorophores for their biological applications.

## 1 Introduction

**Figure 1:**
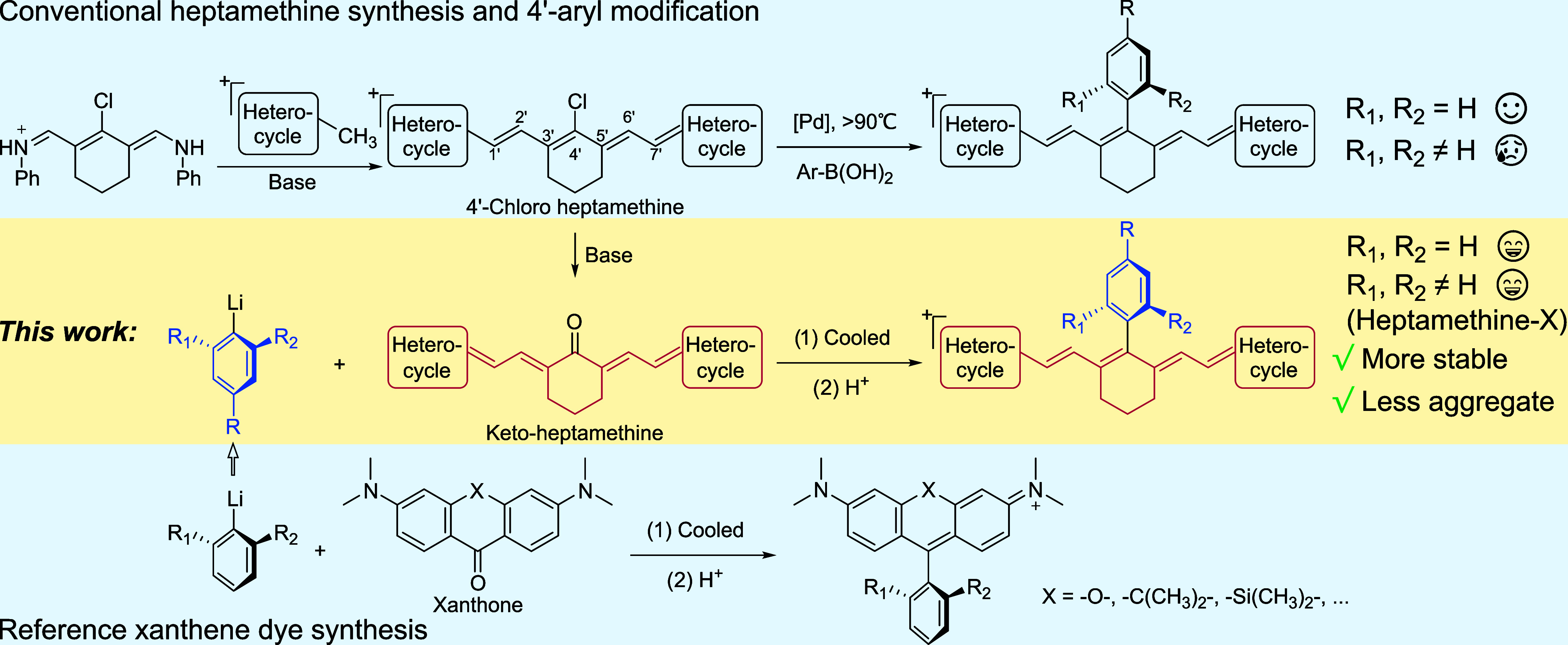
Heptamethine fluorophore synthesis and its 4′-aryl modification with the conventional method (top) and our strategy (middle), which is inspired by the synthesis of xanthene dyes (bottom).

Polymethine dyes, characterized by two heterocycles connected by odd numbers of methine units, are a major family of dyes with more than 150 years of history.^1^ In particular, heptamethine fluorophores, which are polymethine dyes with seven methine units (Figure 1), have gained significant interest due to their favorable near-infrared (NIR, 700–1000 nm) excitation and emission properties that enable a deeper penetration and reduced background than visible fluorophores, making them highly suitable for in vivo imaging applications. The successful implementation of indocyanine green (ICG) in various clinical settings exemplifies the potential of this fluorophore family.^2^ Furthermore, the recent FDA approval of OTL-38, a folate receptor-targeted heptamethine conjugate, underscores the growing impact of these dyes in clinical translation.^3,4^ Moreover, by strategically incorporating specific heterocycles in the scaffold, researchers can achieve even longer emission wavelengths, extending into the shortwave-infrared (SWIR, 1000–2000 nm, also known as NIR-II) region for in vivo imaging with a deeper penetration and an extraordinary contrast.^5−8^

As with any fluorophores, heptamethine dyes require modification prior to biological applications to introduce a proper physical property, chemical behavior, and biological activity. In particular, the planar conjugated systems in heptamethine dyes result in the strong tendency toward nonemissive aggregation with little solubility in water, which requires the modification with hydrophilic groups and/or steric bulk to allow their imaging in aqueous conditions.^9−12^ Additionally, the stability of heptamethine dyes can introduce complexity into their imaging applications. For example, heptamethine dyes are photobleached relatively fast under illumination compared to other families of fluorophores,^13,14^ and many, including ICG, have been reported to degrade in the presence of physiological nucleophiles or even in simple aqueous solutions.^10−12,15,16^ While these limitations can be partially overcome by using formulation methods, the heterogeneous nature of the formulation brings about batch-to-batch variations as well as potential in vivo instability. Therefore, the structural modification of heptamethine fluorophores is often essential to enhance their physical and chemical properties for reliable biological applications in physiological conditions.

Despite the necessity of heptamethine dye modifications, the limited scope of modification methods remains as a hurdle to an even broader utilization of these fluorophores. In this context, the late-stage functionalization of existing heptamethine dyes primarily focuses on the replacement of the central chlorine atom in the popular 4′-chloro heptamethine scaffold, which is synthesized from the condensation between two heterocyclic salt moieties and an accessible Schiff base bearing the chlorine atom (Figure 1).^5−8,17^ Such a replacement can take place via the substitution reaction with N, O, or S nucleophiles, whose products can be labile under physiological conditions.^18−23^ The center chlorine can also be replaced with robust C–C bonds by coupling reactions, although such conversion usually requires a high temperature and a prolonged reaction time that can be harmful to the fluorophore (Figure 1).^24,25^ On the other hand, a more complex modification on the polymethine chain, including bulky 4′-substitutions or substitutions on other positions, requires the bottom-up synthesis of the fluorophore core, such as the use of a customized Schiff base linkage carrying such modifications (Figure S1a).^26,27^ Recent development using the ring-opening of pyridium reagents in place of the Schiff base offers a viable way to build fluorophores with one or more substituents on the polymethine chain (Figure S1b,c).^28,29^ However, there are uncertainties in synthesizing fluorophore cores with these customized building blocks, as many heptamethine dyes with demanding heterocycles require different reaction conditions (i.e., selection of the base, solvent, temperature, and reaction time) and rigorous purification even with established Schiff base building blocks.^30−34^ Moreover, for some challenging heptamethine scaffolds, thermal truncation can occur besides other side reactions during their synthesis, resulting in compromised yields and complex mixtures that are difficult to separate.^35,36^ As such, a mild and effective modification method for heptamethine dyes is highly desired to facilitate the tuning of these fluorophores toward various requirements.

We herein report an efficient aryllithium addition pathway to introduce various aryl substitutions to the 4′-position of polymethine dyes inspired by the synthesis of xanthene dyes. Xanthene dyes, such as rhodamine and fluorescein, can be constructed from the nucleophilic addition between a xanthone and an aryl carboanion, usually obtained from aryl bromide after a lithium–halogen exchange, followed by acidic dehydration (Figure 1). This popular transformation has furnished a variety of xanthene dye derivatives, including red-shifted fluorophores with a heteroatom exchange^37−41^ and has been incorporated in many fluorescent probes for advanced bioimaging and biosensing.^42−44^ Inspired by these successes, we have noticed the structural similarity between the xanthone substrate in xanthene dye synthesis and the keto-form of heptamethine dyes, the S_RN_1 hydrolysis product of common 4′-chloro heptamethine fluorophores, and discovered that such a lithium addition reaction can be used to construct 4′-aryl-modified heptamethine dyes (Figures 1 and S1d). Using this strategy, we show our successful preparation of a panel of 4′-aryl-substituted heptamethine fluorophores starting from the 4′-chloro precursor. This conversion is compatible with a variety of functional groups and tolerates moieties with strong steric demands, affording ortho-disubstituted aryl substitution at the 4′-position of the heptamethine fluorophore (denoted as heptamethine-X), which benefits from the enhanced stability and reduced aggregation but is otherwise inaccessible from palladium-catalyzed modifications. We finally showcase our strategy by a facile synthesis of an **IR-780** derivative that carries two octadecyl chains as a fluorogenic membrane marker with a significantly higher contrast over a commercial DiR stain. Collectively, this xanthene dye-inspired synthesis of 4′-modified polymethine fluorophores is a further step in unifying the design and synthetic principles between these two families toward the make of next-generation fluorophores with combined benefits. Additionally, our effective modification method pushes forward the modularization of imaging agents, where generic heptamethine dyes can be easily functionalized for improved chemical, physical, and biological activities for biomedical imaging.

## 2 Results and Discussion

### 2.1 Synthesis Development

We are interested in the development of this new polymethine dye synthesis because the building blocks have been well studied and can be used as modular building blocks to create new fluorophores with the desired properties. From the xanthene dye community, a variety of aryllithium reagents have been previously prepared from respective aryl bromide reagents to prepare xanthene fluorophores in diverse imaging and sensing requirements (Figure 1).^37,45−49^ On the other hand, polymethine chemists have made copious examples of heptamethine dyes with 4′-chloro substitution spanning wavelengths from NIR to SWIR. Additionally, a few 4′-chloro heptamethine dyes have been conveniently converted to the keto-form via the S_RN_1 reaction in the presence of sodium acetate^50−52^ or *N*-hydroxysuccinimide.^53,54^ Against this backdrop, relating these two building blocks to the creation of 4′-aryl-substituted heptamethine dyes makes it easier to correlate synthetic and design strategies between the xanthene fluorophores and the red-shifted polymethine scaffolds.

We selected **IR-780** (Figure S2) as our initial substrate, as this heptamethine dye is commercially available with a low cost.^55^ The conversion of **IR-780** to the keto-form **1** (**IR780**=**O**) using sodium acetate proceeded smoothly with a 93% yield. With this substrate, we tested the scope of the reaction with aryl bromide reagents found in the reported xanthene dye synthesis, and the results are summarized in Table 1. We started with bromobenzene, the simplest substitution, and found that the conversion to **2a** (**IR780-Ph**) was near quantitative. Notably, the reaction condition employed here was generic for the addition of the lithium–halogen-exchanged intermediate to the xanthone derivatives, except that we used an excess amount of the easily accessible aryllithium reagent to ensure the full consumption of the more expensive fluorophore intermediate. We then introduced ortho-substituents to the aryl group, which is a frequently used structure in the xanthene dyes. We started with 2-bromotoluene, which was used in the synthesis of xanthene dyes such as TokyoGreen^37^ and TokyoMagenta,^38^ and as expected, the reaction afforded **2b** [**IR780-Ph(Me)**] also with a high yield (93%). The reaction to introduce larger inert functionalities at the ortho-position of the central phenyl substitution, including bulky isopropyl in **2c** [**IR780-Ph(*i*Pr)**] and methoxy in **2d** [**IR780-Ph(OMe)**], also succeeded, with good to excellent yields. We then turned to aryl substitutions with ortho-nucleophilic functionalities, as this has been widely explored in the xanthene dye designs for on–off modulation of the fluorophore. With nucleophilic groups protected in aryl bromide, we were able to synthesize **2e** [**IR780-Ph(CH**_**2**_**OH)**] using trimethylsilyl ether as protection for the hydroxyl group and **2f** [**IR780-Ph(COOH)**] using *tert*-butyl ester as protection for the carboxyl group, both resulting in appreciable yields. To note, these protection groups do not need an additional deprotection step, likely due to the help from the newly formed alkoxide prior to acid quenching. The reaction is also compatible with aryl bromide containing the *tert*-butyl thioether group to furnish **2g** [**IR780-Ph(CH**_**2**_**S*t*Bu)**], a precursor toward the thiol-containing heptamethine dye.

**Table 1:**
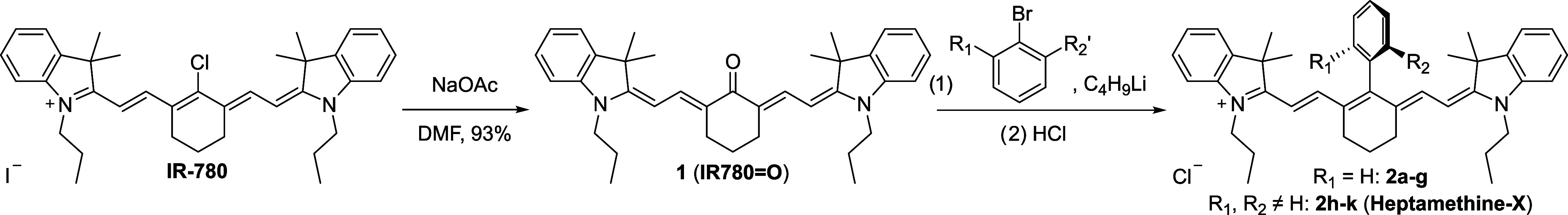
Synthesis of 4′-Aryl-Substituted **IR-780** Derivatives

product	R_1_	R_2_	C_4_H_9_Li	yield^a^ (%)
**2a** (**IR780-Ph**)	H	H	*n-*BuLi^b^	>99
**2b** [**IR780-Ph(Me)**]	H	Me	*n*-BuLi^b^	93
**2c** [**IR780-Ph(*i*Pr)**]	H	*i*Pr	*n*-BuLi^b^	75
**2d** [**IR780-Ph(OMe)**]	H	OMe	*n*-BuLi^b^	98
**2e** [**IR780-Ph(CH_2_OH)**]	H	CH_2_OH^c^	*n*-BuLi^b^	78
**2f** [**IR780-Ph(COOH)**]	H	COOH^d^	*n*-BuLi^b^	50
**2g** [**IR780-Ph(CH_2_S*t*Bu)**]	H	CH_2_S*t*-Bu	*n*-BuLi^b^	95
**2h** [**IR780-Ph(2Me)**]	Me	Me	*t*-BuLi^e^	75
**2i** [**IR780-Ph(2*i*Pr)**]	*i*Pr	*i*Pr	*t*-BuLi^e^	31
**2j** [**IR780-Ph(2CH_2_OAllyl)**]	CH_2_OAllyl	CH_2_OAllyl	*t*-BuLi^e^	91
**2k** [**IR780-Ph(2CH_2_OH)**]^f^	CH_2_OH	CH_2_OH	na	60^f^

a Isolated yields.

b Reaction condition: aryl bromide (12 equiv) was reacted with *n*-BuLi (8 equiv) in tetrahydrofuran (THF) at −84 °C for 10 min, then **IR780**=**O** (1 equiv) was added and reacted at room temperature for 30 min before quenching with HCl.

c R_2_′ = OTMS.

d R_2_′ = COO*t*-Bu.

e Reaction condition: same as (b), except that aryl bromide (0.34 mmol) was reacted with *t*-BuLi (0.68 mmol) for 40 min.

f Synthesized from the deprotection of **2j** (Supporting Information), overall yield listed.

Encouraged by the mild condition and high yields of our modification method, we then seeked to introduce steric bulk to the 4′-position of Cy7, as the increasing steric demand has been reported to not only discourage undesired fluorophore aggregation but also enhance the stability of the molecule.^11^ Against this backdrop, we first used 2-bromo-1,3-dimethylbenzene as a simple substrate. To compensate for the increased steric demand, we used *tert*-butyllithium as a more reactive reagent while keeping the rest of the procedure the same. Satisfyingly, the reaction afforded **2h** [**IR780-Ph(2Me)**] in a 75% yield. Taking a step further, we used 2-bromo-1,3-diisopropylbenzene as the substrate, with considerable steric bulk at the reaction site that is challenging even for the canonical synthesis of xanthene dyes.^56^ Although a part of the reactants underwent uncharacterized isomerization, affording a blue-shifted heptamethine side product, the reaction still furnished **2i** [**IR780-Ph(2*i*Pr)**] with a lower yield of 31%. Additionally, to incorporate both steric bulk and hydrophilicity into the fluorophore scaffold, we incorporated two hydroxymethyl groups as ortho-substitutions on 4′-phenyl. The addition reaction proceeded smoothly to give **2j** [**IR780-Ph(2CH**_**2**_**OAllyl)**] with allyl ether as the protection group of the hydroxyls. Further deprotection of allyl ether by palladium catalysis provided **2k** [**IR780-Ph(2CH**_**2**_**OH)**] with two free hydroxyl groups around the methine bridge with an overall yield of 60%. Owing to their xanthene dye-inspired synthesis and the “X” shape of the molecule when viewed along the 4′ C–C bond, we coined the name “heptamethine-X” for heptamethine fluorophores **2h**–**2k** with 4′ aryl substitution carrying two ortho-functionalities. Collectively, these successes highlight the merit of our method in the synthesis of challenging polymethine fluorophores using existing and new aryl bromide building blocks.

Remarkably, several of these modifications, especially the heptamethine-X scaffold, are previously not accessible from conventional Suzuki coupling conditions on 4′-chloro Cy7 scaffolds. Specifically, the introduction of ortho-unsubstituted or monosubstituted aryl moieties included in **2a** and **2f**, by Suzuki coupling with the corresponding boronic acids, generally requires prolonged heating (several hours to days) at a high temperature (≥90 °C) with modest yields.^24,25,57−59^ Due to the increasing steric demand, the Suzuki coupling of ortho-methyl-substituted phenyl was only reported on the Schiff base prior to the fluorophore synthesis under even harsher conditions,^11^ and the ortho-disubstituted counterpart was only reported using bottom-up synthesis from customized Zincke salt.^60^ These difficulties showcase the advantage of our mild and fast pathway for the introduction of 4′-aryl substitutions to existing heptamethine fluorophore scaffolds without rebuilding the fluorophore core.

**Scheme 1:**
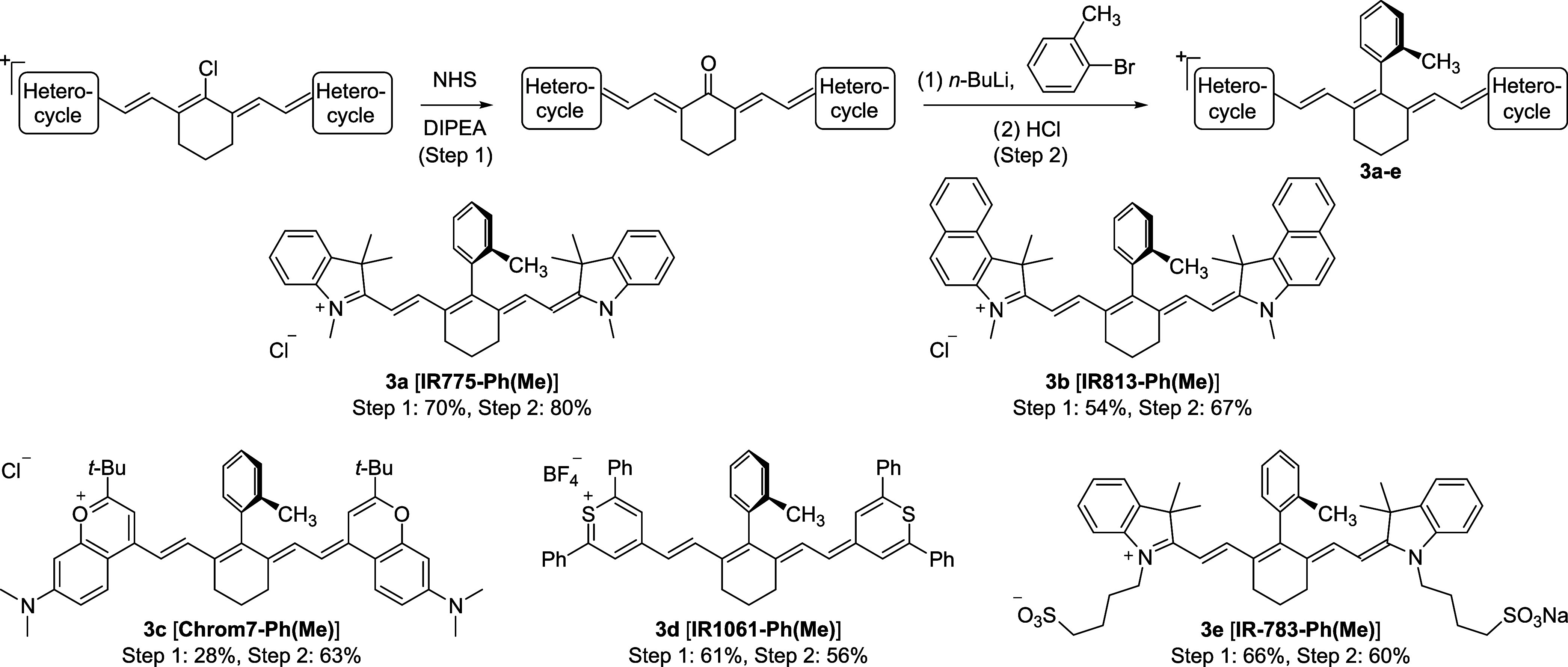
Synthesis of 4′-(2-Methyl)phenyl-Substituted Heptamethine Dyes with Different Heterocycles Reaction conditions for step 1: the 4′-chloroheptametine dye (1 equiv), *N*-hydroxysuccinimide (NHS, 3 equiv), and *N*,*N*-diisopropylethylamine (DIPEA, 3 equiv) were reacted under N_2_ in dimethylformamide (DMF) until the starting dye was consumed as monitored by thin layer chromatography (TLC). Reaction conditions for step 2: 2-bromotoluene (12 equiv) was reacted with *n*-BuLi (8 equiv) or *t*-BuLi (24 equiv) in THF at −84 °C for 10 min, and then keto-dyes (1 equiv) were added and reacted at room temperature for 30 min before quenching with the acid. Isolated yields are listed.

We then tested the compatibility of our methods on heptamethine fluorophores with other heterocycles (Scheme 1). While the hydrolytic conversion of these substrates to keto-intermediates was observed using low-cost sodium acetate as in our initial work with **IR-780**, more side reactions took place for these heptamethine dyes carrying different heterocycles. Moreover, the reduced solubility of these scaffolds in organic solvents became problematic for their purification. Luckily, previous investigations have identified the succinimide N-oxide anion as a more efficient base for this S_RN_1 hydrolysis,^53,54^ and we opted for this reaction condition for examples in Scheme 1. We began with **IR-775**, another Cy7 dye with dimethyl substitutions in indolium nitrogen (Figure S2). The lack of longer aliphatic chains in **IR-775** makes this compound less soluble than **IR-780**. Despite this complication, the first step offered its keto-intermediate with a good conversion, and the aryllithium addition to prepare **3a** [**IR775-Ph(Me)**] in the second step readily proceeded with an 80% yield. We then moved to heptamethine dyes with longer working wavelengths, including the NIR dye **IR-813** and SWIR emitters **Chrom7** and **IR-1061** (Figure S2). Although these extended conjugated structures exhibit an even lower solubility in organic solvents, their synthesis was carried out successfully without the need to adapt reaction conditions to afford **3b**–**d**. Lastly, to showcase the compatibility with heptamethine dyes carrying multiple sulfonate groups, a well-established functionality for promoting hydrophilicity to hydrophobic fluorophores, we picked **IR-783** (Figure S2) as a substrate. Although more soluble in water, molecules with sulfonate groups are generally insoluble in less polar organic solvents. To circumvent this issue, we exchanged the sodium counterion into tetrabutylammonium for the intermediate during its purification by high performance liquid chromatography (HPLC), affording the keto-form of **IR-783** freely soluble in THF during the second step. With this modification, we employed our general synthetic methods and successfully obtained **3e** [**IR783-Ph(Me)**]. Taken together, these results demonstrate the versatility of our method to introduce aryl substitutions at the 4′-position of heptamethine dyes with a variety of heterocycles, enabling further tuning of the properties of the fluorophore for various imaging applications.

### 2.2 Improved Performances of Heptamethine Fluorophores by 4′-Aryl Substitution

With the fluorophores in hand, we further tested their photophysical properties and compared them to those of the parent fluorophore. As summarized in Table 2, all newly synthesized **IR-780** derivatives show absorption maxima within a narrow range of 762–768 nm and emission maxima in the range of 784–795 nm in ethanol (Figures S3 and S4), which confirms that the 4′-aryl substituents are not coplanar with the fluorophore core, participating little in the conjugation system of the fluorophore. These wavelengths are slightly blue-shifted compared to the parent **IR-780** due to the removal of the conjugated chlorine atom. Similarly, modification with ortho-methylphenyl on other heptamethine scaffolds results in a slight blue shift of the emission and excitation wavelengths (Table S2). These **IR-780** derivatives show high absorption coefficients (>2 × 10^5^ M^–1^ cm^–1^ in ethanol), which is characteristic of polymethine fluorophores. Additionally, by replacing the chlorine atom in **IR-780** with aryl substituents, these fluorophores exhibit 1.5- to 2-fold higher fluorescent quantum yields in ethanol as the starting **IR-780**, which is in agreement with previous observations of similar modifications.^25,57,63^ The lower quantum yields with derivatives carrying isopropyl groups might originate from the added relaxation pathways resulting from these bulky substitutions. We also tested photophysical properties of some fluorophores in aqueous solutions and observed similar absorption coefficients and quantum yields with **IR-780** (Table 2, Figure S5). The overall similar photophysical behavior of the new derivatives suggests that aryl modification at the 4′ position does not much affect the fluorophore core.

**Table 2:** Photophysical Properties of Cy7 Derivatives^a^,^b^

compound	solvent	λ_max,abs_/nm	ε/10^5^ M^–1^ cm^–1^	λ_max,ems_/nm	Φ_F_
**IR-780**	ethanol	784	2.17	806	0.21
	water	753	1.51	777	0.11
**2a** (**IR780-Ph**)	ethanol	762	2.37	784	0.37
	water	774	1.41	795	0.051
**2b** [**IR780-Ph(Me)**]	ethanol	764	2.73	787	0.37
**2c** [**IR780-Ph(*i*Pr)**]	ethanol	765	2.46	785	0.258
**2d** [**IR780-Ph(OMe)**]	ethanol	764	2.46	788	0.34
**2e** [**IR780-Ph(CH_2_OH)**]	ethanol	765	2.76	789	0.37
	water	757	1.71	782	0.096
**2f** [**IR780-Ph(COOH)**]	ethanol	762	2.05	784	0.34
	water	753	1.18	777	0.12
**2g** [**IR780-Ph(CH_2_S*t*Bu)**]	ethanol	767	2.56	791	0.33
**2h** [**IR780-Ph(2Me)**]	ethanol	766	2.73	789	0.36
**2i** [**IR780-Ph(2*i*Pr)**]	ethanol	774	2.72	798	0.245
**2j** [**IR780-Ph(2CH_2_OAllyl)**]	ethanol	768	2.34	794	0.38
**2k** [**IR780-Ph(2CH_2_OH)**]	ethanol	768	2.47	794	0.39
	water	762	1.95	785	0.11

a See Table S1 for error values.

b ICG in ethanol (Φ_F_ = 0.132)^61,62^ was used as a reference.

**Figure 2:**
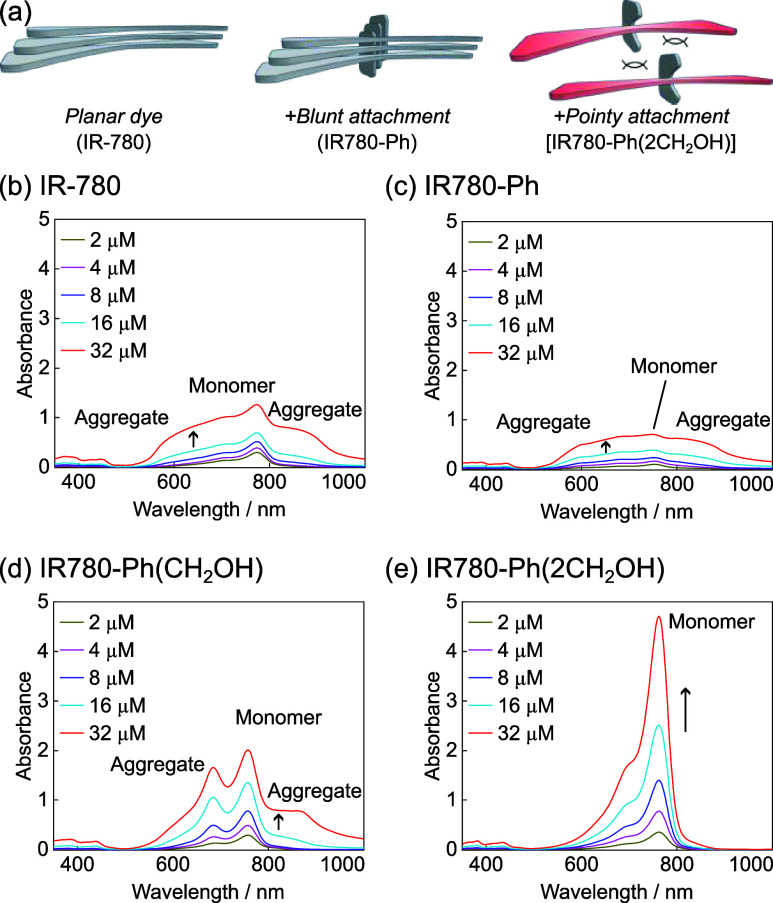
4′-Aryl modification on heptamethine for reduced aggregation. (a) Schematic showing the reduction of aggregation by introducing bulky and pointy attachment to the planar dye. (b–e) Normalized absorption spectra of increasing concentrations of (b) **IR-780**, (c) **IR780-Ph**, (d) **IR780-Ph(CH**_**2**_**OH)**, and (e) **IR780-Ph(2CH**_**2**_**OH)** in phosphate buffered saline (PBS). Normalized spectra are shown in Figure S6.

We then tried to verify the reduction of the aggregation tendency for the fluorophore carrying 4′-phenyl with ortho-disubstitutions on heptamethine-X compared to the parent dye and its monosubstituted derivative. The perpendicular orientation of the 4′-aryl group positions its ortho-functionalities right over the polymethine bridge. This pointy attachment can discourage the close stacking between fluorophores to reduce the aggregation of fluorophores (Figure 2a). Previous reports have utilized Zincke salt carrying ortho-substituents to create “shielded” Cy7 derivatives for reduced aggregation, which requires lengthy synthesis.^60^ A simpler strategy uses Suzuki coupling on the Schiff base to access 4′-(ortho-methyl)phenyl substitution on a SWIR-emitting heptamethine, also showing a greatly reduced aggregation behavior compared with the analogue without the ortho-methyl group;^11^ however, the difficulty of synthesizing the ortho-disubstituted derivative prevents further exploration of this effect.

Toward this end, we selected **IR780-Ph**, **IR780-Ph(CH**_**2**_**OH)**, and **IR780-Ph(2CH**_**2**_**OH)** and compared their water solubility against **IR-780**. This series of compounds are examples of how we can apply our modification toward more soluble biocompatible fluorophores, where we first replace the electrophilic C–Cl bond with a stable C–C bond and then introduce steric bulk as well as hydrophilicity on one or two sides of the original hydrophobic planar dye molecule. Since all dyes are able to form monomeric absorption in dilute solutions in water (Figure S5), we used PBS as the solvent, where the existence of saline can induce the aggregation of the heptamethine dyes even with multiple hydrophilic functionalities.^64^ We measured the absorption profile of each dye with the increasing concentration to determine the monomer population in solution (Figures 2b–e and S6). While **IR-780** is able to dissolve in PBS at 2 μM and show primarily monomer absorption, the aggregated population, both red-shifted and blue-shifted, increases dramatically as concentration increases, resulting in an absorption spectrum largely deviating from monomer cyanine absorption profiles at 32 μM (Figure 2b). The aggregation propensity of **IR780-Ph** is even larger (Figure 2c), as the hydrophobic phenyl group can also form stacking perpendicular to the dye plane and thus promote aggregation (Figure 2a). In contrast, with the introduction of hydroxymethyl as the ortho-substituent in **IR780-Ph(CH**_**2**_**OH)**, which brings in steric bulk and hydrophilicity to one side of the molecule, the monomeric absorption becomes more dominant at higher concentrations with a more defined blue-shifted absorption peak, likely from H-dimers (Figure 2d). With hydroxymethyl groups on two sides in **IR780-Ph(2CH**_**2**_**OH)**, this solubilization effect is significantly enhanced, affording a cyanine-like absorption profile almost independent of the concentration (Figure 2e), reaching an absorbance of 4.7 at 32 μM with a 1 cm light path, which is almost 4-fold compared to **IR-780** at the same concentration. This strong reduction in aggregation in **IR780-Ph(2CH**_**2**_**OH)** demonstrates the necessity of introducing modifications on both sides of the fluorophore, and thus the introduction of 4′-aryl substitution carrying the ortho-dihydroxymethyl functionality in our heptamethine-X scaffold provides a facile choice for modifying existing fluorophores toward reduction of aggregation in water.

**Figure 3:**
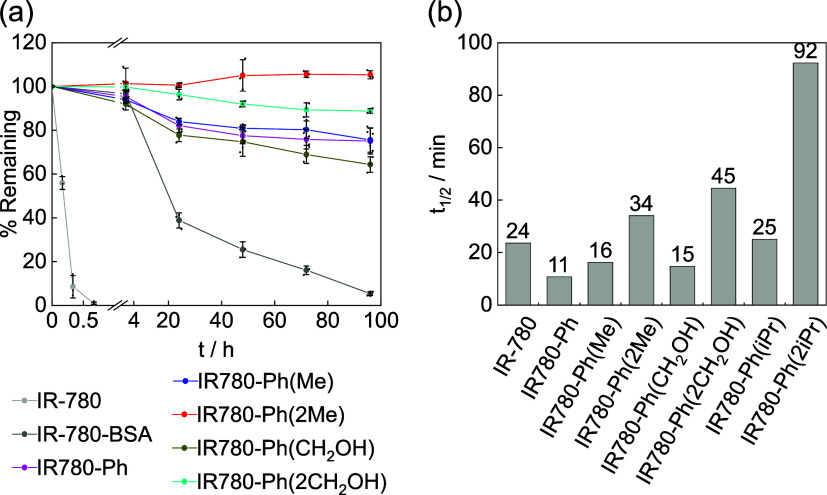
Improved stability of new **IR-780** derivatives with 4′-aryl substitutions. (a) Degradation of dyes over time in bovine calf serum at 37 °C in the dark. **IR780-BSA** refers to the dye-serum protein conjugate. (b) Half-lives of dyes in 1:1 methanol/water solution under 730 nm light-emitting diode (LED) illumination (6.8 mW cm^–2^); see Figure S7 for photobleaching curves, dye absorption, and the LED luminescence profile.

We next sought to verify the improvement of stability of fluorophores upon the introduction of ortho-substituents on 4′-aryl modifications. Heptamethine dyes, including ICG, undergo degeneration under aqueous conditions due to the oxidation and/or nucleophilic breakdown of the methine bridge,^15^ and we hypothesize that by introducing steric bulk over the polymethine bridge, such processes can be slowed down to afford more stable fluorophores. We thus incubated the serum solutions of **IR-780** and its derivatives with 4′-aryl substitution carrying mono/dimethyl or mono/dihydroxymethyl at the ortho-positions at 37 °C in the dark and monitored the remaining dyes in solution over time. As expected, the 4′-position of **IR-780** underwent rapid substitution with nucleophilic side-chains of serum proteins to afford fluorophore-labeled proteins within 40 min (Figure 3a), which indicates the incompatibility of using 4′-chloro heptamethine fluorophores as a solution under physiological condition. The labeled protein also showed fast degradation, as shown by the decrease of absorption, and minimal absorption was left after 4 days (Figure 3a). In contrast, more than 70% of **IR780-Ph** remained after 4 days of incubation due to the replacement of Cl as a strong leaving group with a robust C–C-bonded substitution. The stability is further enhanced with the introduction of a single ortho-methyl group on the 4′-aryl ring, whereas little degradation of **IR780-Ph(2Me)** with two ortho-methyl groups was observed during the experiment. Although ortho-hydroxymethyl groups made **IR780-Ph(CH**_**2**_**OH)** more susceptible to degradation than **IR780-Ph**, the disubstitution of hydroxymethyl on **IR780-Ph(2CH**_**2**_**OH)** resulted in only 10% degradation over 4 days for this heptamethine-X scaffold (Figure 3a).

We also characterized the influence of such steric bulk toward the photostability and, similarly, observed a higher stability under light with larger functionalities on ortho-positions of the 4′-phenyl substitution. The substitution of 4′-chloro in **IR-780** with 4′-phenyl in **IR780-Ph** almost doubles the photobleaching rate, likely due to the higher electron density on **IR780-Ph** that makes the heptamethine bridge more reactive toward photogenerated oxidants (Figure 3b). On the other hand, the introduction of ortho-substitutions significantly slows down the photobleaching rate. Again, while ortho-mono substitution on the 4′-aryl ring modestly increases the photostability, such an effect is more pronounced with two ortho-substitutions in heptamethine-X, producing fluorophores more photostable than the parent **IR-780**. Notably, a single isopropyl at the ortho-position as in **IR780-Ph(*i*Pr)** makes the photostability of the fluorophore already comparable to the parent **IR-780**, whereas the introduction of two isopropyl groups in **IR780-Ph(2*i*Pr)** significantly enhances the photostability, producing a fluorophore with almost a 4-times longer half-life than the parent **IR-780** and more than 8-fold longer than **IR780-Ph** under the same illumination condition (Figure 3b). Collectively, these improvements in the stability underscore the efficacy of stability improvement by placing steric protection over the methine bridge in heptamethine dyes, particularly when using bulky functionalities and on the two sides of the fluorophore as in our heptamethine-X scaffold.

### 2.3 Fluorogenic Cy7 Fluorophore for Membrane Staining

**Figure 4:**
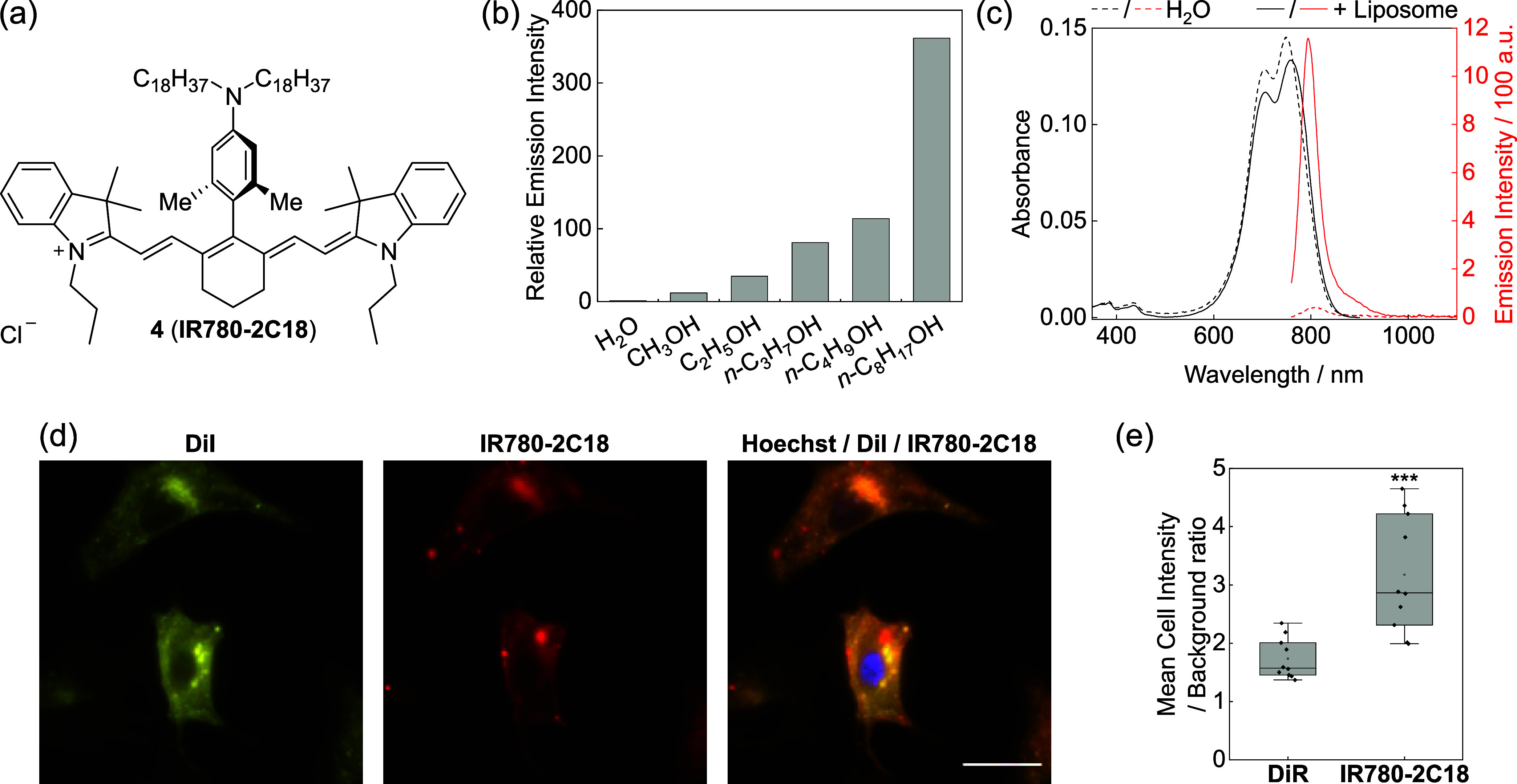
Imaging of cell membrane with **IR780-2C18**. (a) Structure of the compound **IR780-2C18**. (b) Relative fluorescence intensity of **IR780-2C18** (1 μM) in water and alcohol solvents, normalized to the intensity in a water solution. (c) Absorption and emission spectra of **IR780-2C18** (1 μM) in water with or without preformed liposomes (200 μM, 55:45 DPPC/CHOL). (d) Epifluorescence imaging of the cell membrane on A549 cells using 1.7 μM DiI and **IR780-2C18**. Scale-bar: 20 μm. (e) Comparison of the mean cellular signal to the background ratio for cells incubated with 2.5 μM DiR or **IR780-2C18** under the same imaging condition. Data are calculated on 10 cells from 4 independent incubations for each dye. ***P ≤ 0.001; two-tailed Student’s *t*-test.

Encouraged by these added benefits with the heptamethine-X scaffold, we last pursued their implementation in bioimaging. We synthesized fluorophore **4** (**IR780-2C18**, Figure 4a) using **IR780** = **O** and aryl bromide from the one-step synthesis from commercial compounds. In this fluorophore, the 4′-aryl group carries two ortho-methyl groups for stability improvement and aggregation reduction and two octadecyl groups on the amino functionality at the para-position for membrane localization. Notably, in this molecule, the fluorescence core (from **IR780**=**O**) and the targeting group (4′-substitution) are facilely coupled together as a late-stage functionalization, which is an example of the convergent synthesis of molecular dyes, highlighting the modularity of our method.

Upon obtaining **IR780-2C18**, we tested its photophysical behavior. Different from **2a**–**i**, this fluorophore shows minimal fluorescence in ethanol and methanol, whereas the emission intensity increases when a longer-chain alcohol was used as the solvent (Figures 4b and S8a,b). Indeed, although the molecule shows similar absorption coefficients, its fluorescence quantum yield increases from 0.015 in ethanol to 0.18 in octanol (Table S1). The 33-fold fluorescence turn on from methanol to octanol and the 362-fold turn on from water to octanol, mimics for the membrane environment, suggest that **IR780-2C18** is a fluorogenic dye for membrane imaging. This phenomenon is further supported by the similar absorption spectra but the greatly enhanced emission intensity in the presence of preformed liposomes compared to that in water without liposomes (Figure 4c). Further testing reveals that the emission intensity of **IR780-2C18** is higher in more nonpolar solvents until aggregation happens (Figure S8c,d), suggesting the existence of a charged species during the quenching process. We also observed that in ethanol, the fluorescence of **IR780-2C18** can be rescued by adding HCl (Figure S8e,f), which protonates the amino group on the 4′-aryl substitution while minimally affecting the fluorophore core as shown in **IR780-Ph** (Figure S8g,h). These observations combined suggest that this quenching effect of **IR780-2C18** in polar conditions likely comes from the photoinduced electron transfer (PeT) from the aminophenyl ring to the fluorophore, which is less favorable in nonpolar solvents due to the charge reorganization. While this quenching process on the Cy7 scaffold can be leveraged for designing future biosensors, the fluorogenic effect of **IR780-2C18** on membrane mimics makes it a promising candidate for wash-free imaging of the cell membrane with a higher contrast owing to its fluorogenic property.

We then carried out epifluorescence microscopy to show the membrane-staining properties of **IR780-2C18**. Here, we coincubated the cells with **IR780-2C18** and DiI, a commercial Cy3 derivative bearing two C18 chains on indolium nitrogen to stain the cell membrane in the yellow channel. Indeed, both dyes outlined the cell contour under microscopy with only a slight variation of the bright dots, which could be from the aggregation from each dye (Figure 4d). The high correlation between these two channels (Pearson’s *R* value = 0.93 between the two channels) supports the same membrane-staining capability of **IR780-2C18**. We also used DiR (Figure S2) as a comparison, a commercial Cy7 derivative of DiI. While both dyes successfully stained the cell membrane in a wash-free imaging experiment (Figure S9), the membrane fluorescence to the background signal ratio of **IR780-2C18** was significantly higher than that of DiR (Figure 4e), indicating that the fluorogenic property of **IR780-2C18** results in an enhanced contrast compared to the commercial dye. Additionally, the cellular brightness of **IR780-2C18** was on average 6.6 times higher than that of DiR (Figure S9c), likely due to the reduced aggregation of this new dye in the complex cell membrane environment owing to the heptamethine-X structure. Taken together, the high contrast and brightness of **IR780-2C18** and its retention of the membrane-binding property as the commercial dye counterparts highlight the merit of our modular synthesis of functional heptamethine dyes with improved performance.

## 3 Concluding Remarks

To close, we have reported a general synthetic strategy to convert common heptamethine fluorophores carrying 4′-Cl substitution into 4′-aryl modifications carrying various substituents linked by robust C–C bonds. The synthesis involves the aryllithium addition to the keto-form of the heptamethine dye, which is analogous to the popular synthesis of xanthene dyes including rhodamine, fluorescein, and their derivatives. The transformation is fast, mild, and, most importantly, capable of the late-stage modification of existing 4′-chloro heptamethine dyes without the synthesis of the fluorophore core. The high efficiency of this method allows for the introduction of 4′-aryl with a high steric demand exemplified by the heptamethine-X series dyes, which is inaccessible with the previously reported Suzuki coupling pathway. While this strategy is particularly useful for furnishing heptamethine dyes with high steric demands, it provides insights in the structural analogy of xanthene dyes and polymethine fluorophores and serves as a further step in consolidating the synthetic methods, design principles, and application fields among seemingly different fluorophore families for the next generation of fluorescent probes and sensors. Additionally, this facile late-stage functionalization serves as a starting point for the mild and efficient modification of polymethine scaffolds, allowing future dye chemists to focus on constructing fluorophores with ideal photophysical properties, knowing that their products can be subsequently modified toward soluble, stable, and bioconjugatable imaging agents.

Besides the new synthetic method, we identified improvements of introducing 4′-aryl substitution on heptamethine dyes, especially in heptamethine-X with two ortho-substituents in the central aryl ring. In addition to providing Cy7 derivatives with higher fluorescent quantum yields in organic solvents, introducing the C–C bond in place of the C–Cl bond results in dyes being more resistant to nucleophilic degradation. Additionally, increased steric bulk around the methine bridge from ortho-substituents in heptamethine-X can not only boost the stability in the presence of serum proteins or under light but can also remarkably reduce the aggregation propensity in water when two hydroxymethyl groups are incorporated. Through our facile synthesis of the fluorogenic membrane-staining fluorophore **IR780-2C18**, we showcased the benefits of our new scaffold with its high brightness and contrast in cell imaging experiments compared to commercial membrane-staining dyes. We expect the heptamethine-X structure to be employed in the development of future heptamethine dyes, by both our team and others, for enhanced stability, reduced aggregation, and introduction of hydrophilicity, all of which are particularly pertinent for challenging fluorophores with extremely red-shifted wavelengths.

## 4 Methods

### 4.1 Synthetic Procedures

All synthetic procedures are described in the Supporting Information.

### 4.2 Materials

Indocyanine green (ICG) was purchased from Ambeed. DiR was purchased from MedChemExpress. DiI was purchased from AAT Bioquest. Hyclone calf serum was purchased from Cytiva and supplemented with 0.01% (w/v) NaN_3_ as a preservative. The preformed liposome in PBS (DPPC/CHOL 55:45 mol/mol, 60 mM total concentration) was prepared according to the published procedure and diluted to the desired concentration.^65^ HPLC-grade solvents were used for all photophysical characterizations.

### 4.3 Dye Handling and Storage

All dyes were stored as pure solid after purification in a −20 °C freezer. Stock solutions were prepared as 2 mM solutions in absolute ethanol and diluted to the desired concentration for characterization.

### 4.4 Photophysical Characterization

Absorption spectra were collected on a VWR UV-1600PC scanning spectrophotometer after blanking with the appropriate solvent. Photoluminescence spectra were obtained on a StellarNet SILVER-Nova spectrometer coupled to a Spectral Products ASTN-W100L-CM light source. Quartz or glass cuvettes (10 mm × 10 mm) or polystyrene cuvettes (10 mm × 5 mm) were used for absorption measurements. Quartz cuvettes (10 mm × 10 mm) were used for photoluminescence measurements. All spectra were obtained at ambient temperature. Fluorescence quantum yields weremeasured with 730 nm excitation using indocyanine green (ICG) in absolute ethanol as a reference (Φ_F_ = 0.132).^61,62^ Six or more data points were acquired for the calculation of the absorption coefficient and quantum yield by linear regression, where the standard error of slopes of the unknowns were used to determine error values.

### 4.5 Stability Assay in Fetal Bovine Serum (FBS)

Dyes were diluted to 4 μM in 1 mL of bovine calf serum and placed in disposable cuvettes (10 mm × 5 mm). The cuvettes were sealed with Parafilm and placed in a 37 °C incubator for given time periods. Absorption spectra were taken using the maximum absorption to represent dye concentrations. For cysteine-reacted **IR-780**, pristine **IR-780** was preincubated in the serum at 37 °C for 1 h for the reaction to complete before starting the experiment. For **IR-780**, incubation was carried out in 4 μM 250 μL solutions in microcentrifuge tubes, quenched at given time points with 800 μL of cold methanol, cooled at −20 °C for 30 min, centrifuged (17,000*g*, 5 min) to remove precipitated serum proteins, and measured on a spectrometer. Each condition was performed in triplicates.

### 4.6 Photostability Assay

Dyes were diluted to 4 μM in 1 mL of 1:1 methanol/water and placed in disposable cuvettes (10 mm × 5 mm). The cuvettes were illuminated through a 5 mm light path in front of a self-made LED light (LEDLightsWorld, 730 nm LED strips) matrix (6.8 mW cm^–2^) for given time periods. Absorption spectra were taken using the maximum absorption to represent dye concentrations.

### 4.7 Cell Culture

The A549 human lung cancer cell line (CCL-185, ATCC) was obtained from the American Type Culture Collection. A T25 cell culture flask (Greiner Bio-One) was adopted to culture the A549 cells in the Dulbecco’s modified Eagle’s medium (DMEM) cell culture medium (Corning) containing 1% penicillin–streptomycin (Pen Strep) (Gibco) and 10% fetal bovine serum (FBS) (Gibco). To subculture cells, in a 35 mm Petri dish (Fisher Scientific), a 22 × 22 mm^2^ coverslip (Corning) was added, followed by adding the cell suspension in 150 μL of DMEM with 10% FBS and 1% Pen Strep to submerge the coverslip (2 mL). The Petri dish was left in the cell culture incubator at 37 °C and 5% CO_2_ for 48 h before imaging.

### 4.8 Fluorescence Microscopy

The Nikon Eclipse 80i upright microscope was used to perform the imaging in this study. The microscope was configured in epifluorescence mode and differential interference contrast (DIC) mode. In epifluorescence mode, three sets of filters, Newport filter sets (HPF1425; exc/emi 710/800 nm), the 4′,6-diamidino-2-phenylindole (DAPI) filter (Nikon V-2A; exc/emi 400/450 nm), and the DiI filter (Nikon r-DiI; exc/emi 535/610 nm), were used. A mercury lamp, namely, a Nikon Intenslight C-HGFI lamp, was used. In DIC mode, the sample was illuminated by a halogen lamp. Two Nomarski prisms, one polarizer, one analyzer, and one-quarter-wave plate were used in the optical path. A DIC oil immersion condenser (numerical aperture (NA) 1.40) (Nikon D-CUO) and a 100X Plan Apo VC oil immersion objective (NA 1.40) (Nikon) was used in DIC mode. The images were collected using an electron multiplying charge coupled device (EMCCD) camera (iXonEM+Ultra897 BVF, Andor Technology). Images were recorded with a frame rate of 1–20 frames per second (fps).

For imaging of the cell membrane, the DiI staining medium with a final concentration of 5 μM was made by adding 1 μL of 5 mM DiI stock solution into 999 μL of plain DMEM. The **IR780-2C18** staining medium (final concentration 5 μM) was made by adding 2.5 μL of 2 mM **IR780-2C18** stock solution to 997.5 μL of plain DMEM. The Hoechst staining medium was made by adding 0.5 μL of 16.23 mM Hoechst stock solution into 1000 μL of PBS (1:2000 dilution). The three dyes were mixed in a conical tube (VWR) to furnish the staining medium. Cells were washed once with PBS before incubating with the staining medium at 37 °C and 5% CO_2_ for 20 min before imaging.

Acquired images were analyzed with Fiji distribution of ImageJ.^66^ Pearson’s *R* value was calculated with the Coloc2 plugin. The brightness comparison between DiR and **IR780-2C18** was performed by calculating the absolute intensity, where the mean fluorescence intensity in cells and in the background were selected as regions of interests (ROIs) and measured. The absolute intensity was calculated by subtracting the background intensity from the cell fluorescence intensity.

## Supporting Information Available

Schemes of existing synthetic methods and our late-stage functionalization strategy for modified Cy7 fluorophores; structures of commercially-available or previously-reported heptamethine dyes in this work; normalized absorption and emission spectra of new dyes in ethanol or water solution; photophysical property tables of new heptamethine dyes; characterization of aggregation and photostability of selected dyes, comparison of membrane imaging with **IR780-2C18** and **DiR**; and NMR and mass spectra (PDF)
