# Supplementary material for: Engineering Central Substitutions in Heptamethine Dyes for Improved Fluorophore Performance
Source: JACS Au. 2024 Aug 8;4(8):3007–17. doi: 10.1021/jacsau.4c00343 (PMC11350720; doi:10.1021/jacsau.4c00343)
Supplement: Supplementary file 1 — au4c00343_si_001.pdf [file au4c00343_si_001.pdf]

Supporting Information for

Engineering Central Substitutions in Heptamethine Dyes for Improved Fluorophore Performance

Lei Guo[a,*], Meek Yang[b], Bin Dong[b], Seth Lewman[b], Alex Van Horn[b], Shang Jia[b,c,*]

[a] Department of Civil Engineering, University of Arkansas, Fayetteville, Fayetteville, AR 72701, United States

[b] Department of Chemistry and Biochemistry, University of Arkansas, Fayetteville, Fayetteville, AR 72701, United States

[c] Present address: Department of Chemistry, Rutgers University-Newark, Newark, NJ 07102, United States

* email: leiguo@uark.edu (L.G.), shang.jia@rutgers.edu (S.J.)

Table of Contents

Supplementary Figures 2
- Figure S1 2
- Figure S2 3
- Figure S3 4
- Figure S4 5
- Figure S5 6
- Figure S6 7
- Figure S7 7
- Figure S8 8
- Figure S9 9
- Table S1 10
- Table S2 10

Synthetic Procedures 11
- Synthetic materials and methods 11

NMR and mass Spectra 22

References 65

Supplementary Figures

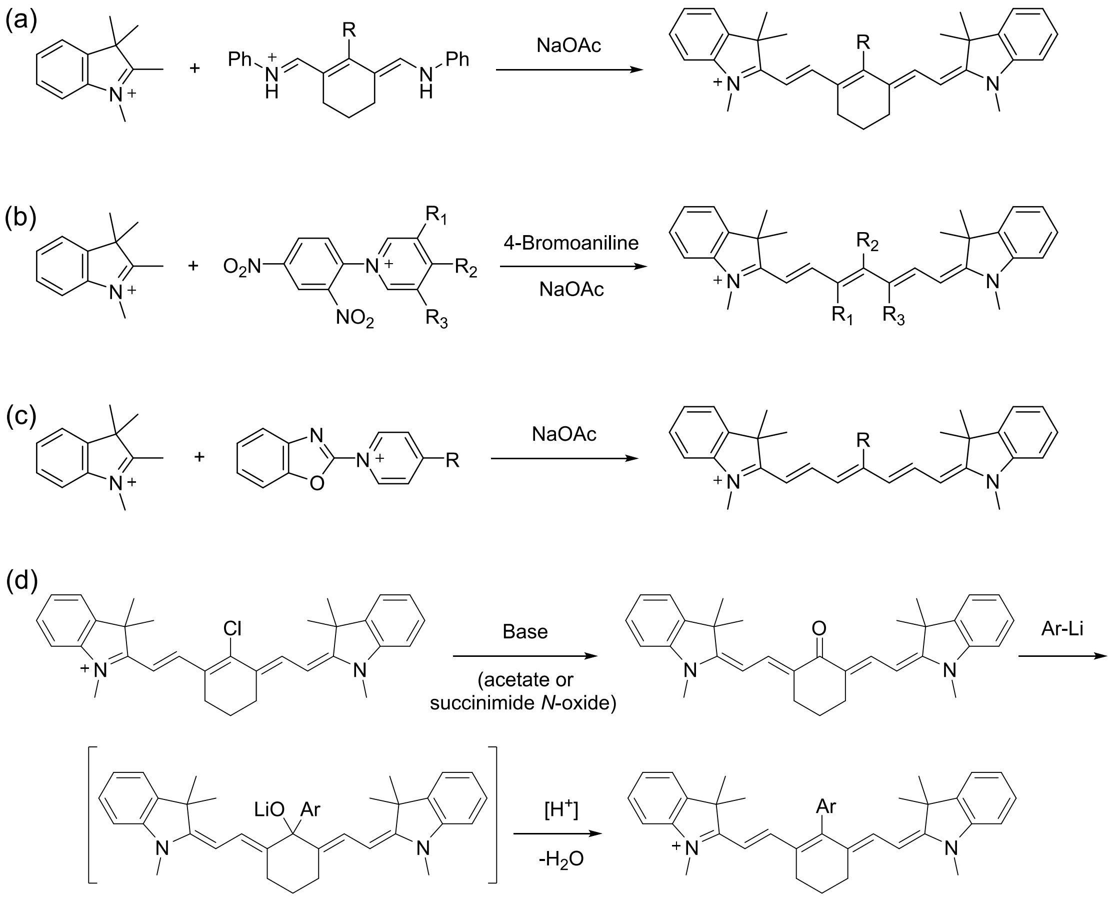

Figure S1 Existing synthetic methods and our late-stage functionalization strategy for indolinium heptamethine cyanine (Cy7) fluorophores containing modification on the methine linkage. (a-c) Synthesis of modified Cy7 dyes from custom Schiff bases (a),[1,2] from Zincke salts (b),[3] and from pyridinium benzoxazoles (c).[4] (d) Our modification starting from the hydrolysis of 4'-chloro Cy7 dyes, followed by aryllithium addition to construct the C-C bond and the subsequent acid workup to reconstitute the fluorophore. The elimination of the tertiary alcohol intermediate can be triggered using strong acid (HCl), weak acid (acetic acid) or during purification on silica.

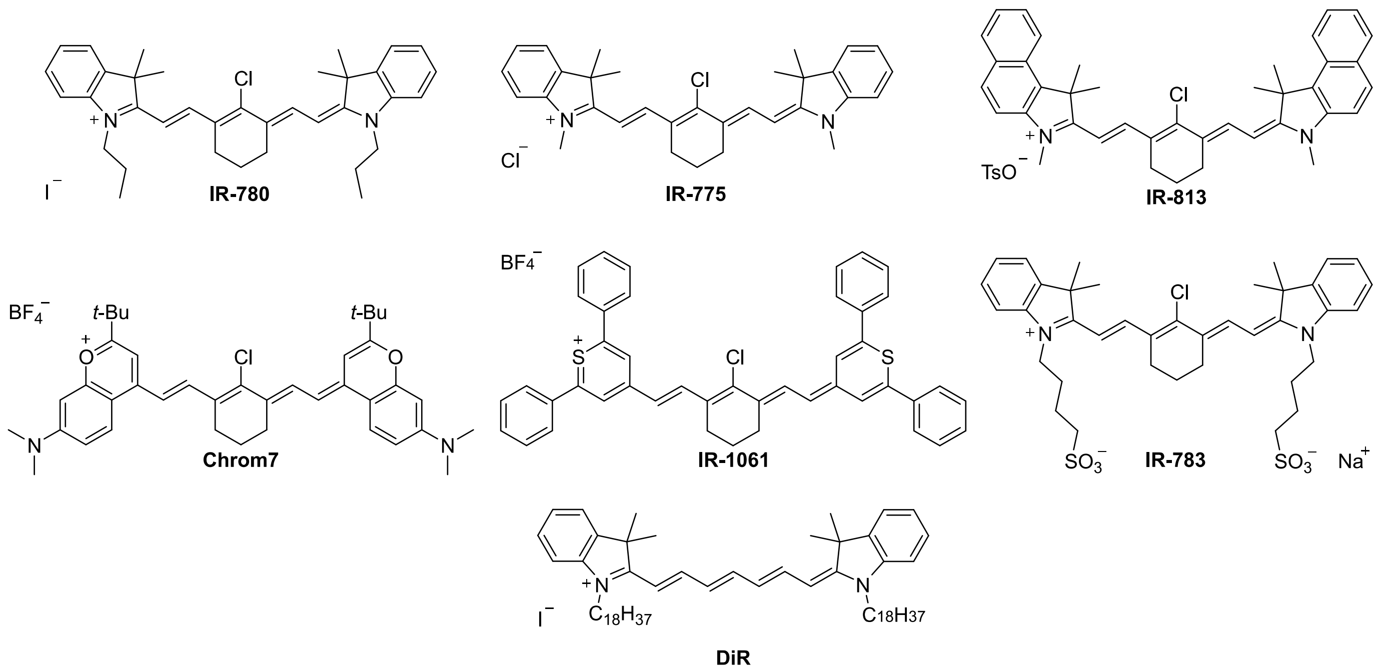

Figure S2 Structures of commercially-available or previously-reported heptamethine dyes in this work.

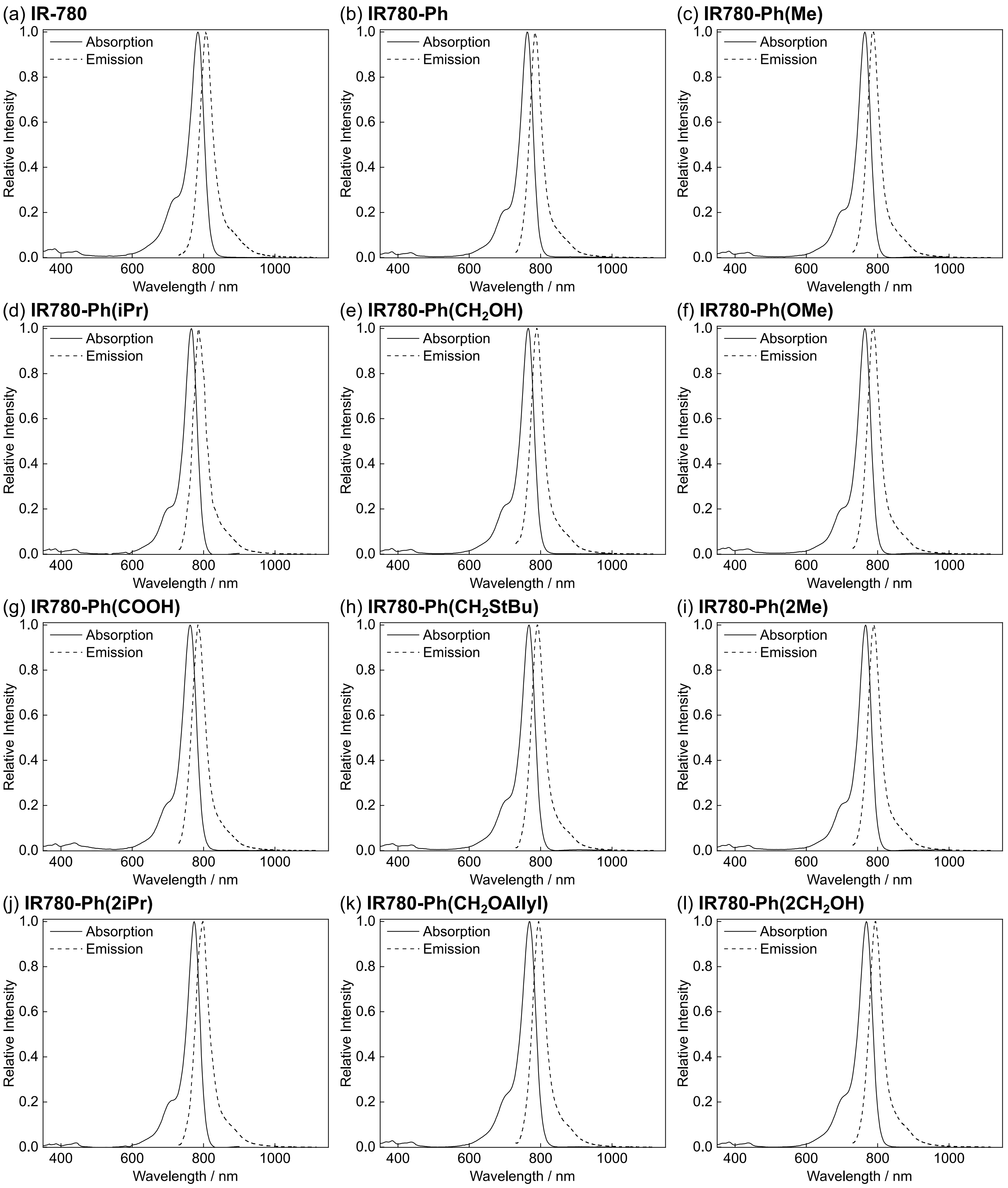

Figure S3 Normalized absorption and emission spectra of ethanol solution containing 2 μM (a) **IR-780**, (b) **IR780-Ph**, (c) **IR780-Ph(Me)**, (d) **IR780-Ph(iPr)**, (e) **IR780-Ph(CH_2OH)**, (f) **IR780-Ph(OMe)**, (g) **IR780-Ph(COOH)**, (h) **IR780-Ph(CH_2StBu)**, (i) **IR780-Ph(2Me)**, (j) **IR780-Ph(2iPr)**, (k) **IR780-Ph(CH_2OAllyl)** and (l) **IR780-Ph(2CH_2OH)**.

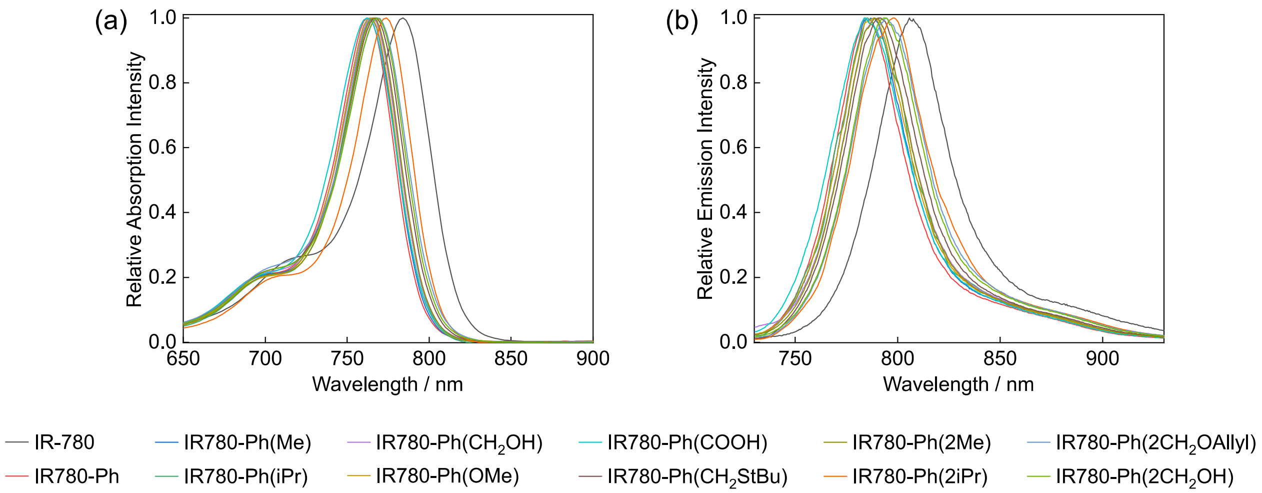

Figure S4 Combined (a) absorption and (b) emission spectra of **IR-780** and its derivatives in ethanol.

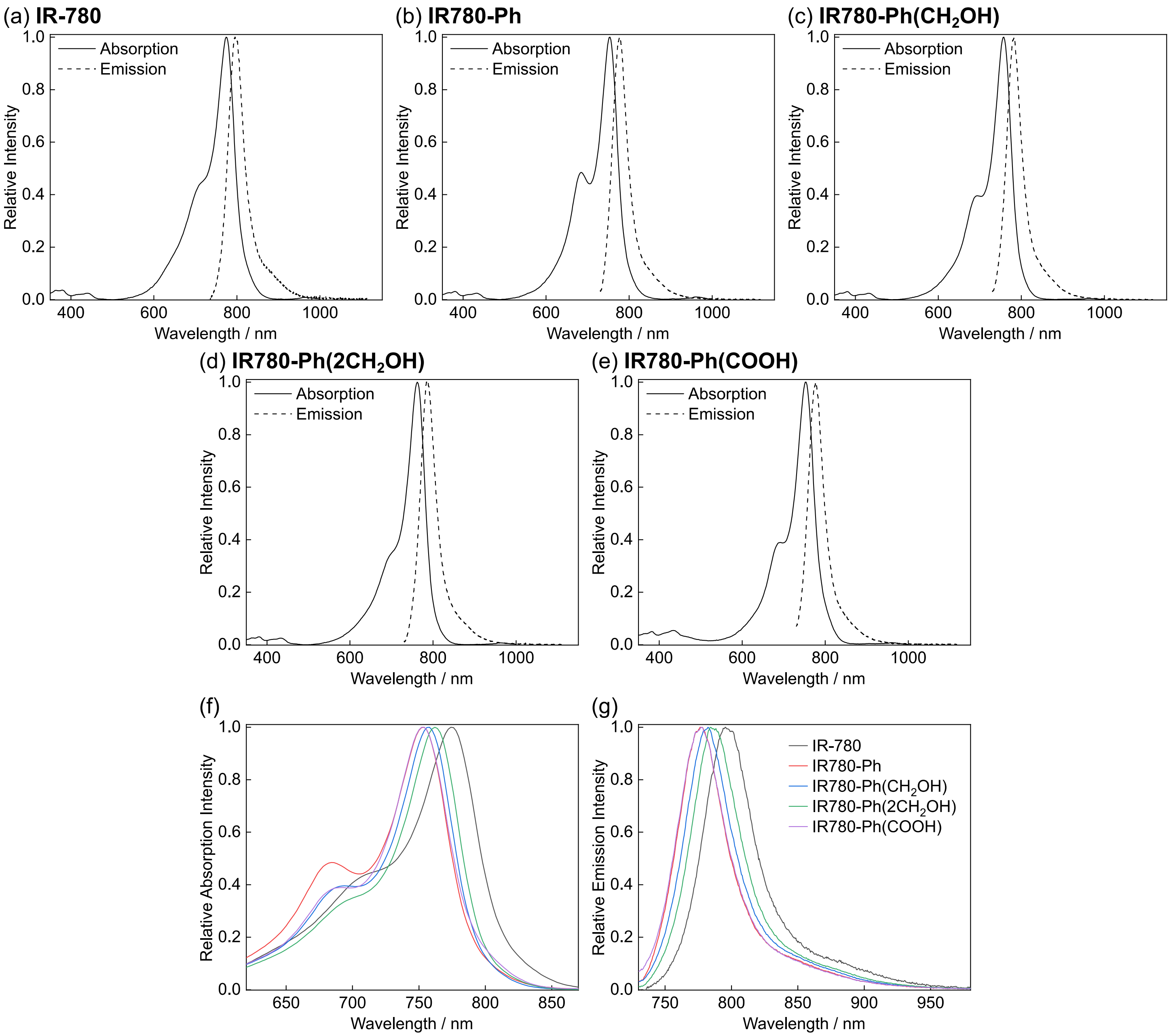

Figure S5 Normalized absorption and emission spectra of water solution containing 2 μM (a) **IR-780**, (b) **IR780-Ph**, (c) **IR780-Ph(CH_2OH)**, (d) **IR780-Ph(2CH_2OH)** and (e) **IR780-Ph(COOH)**. Combined absorption and emission spectra are shown in (f) and (g), respectively.

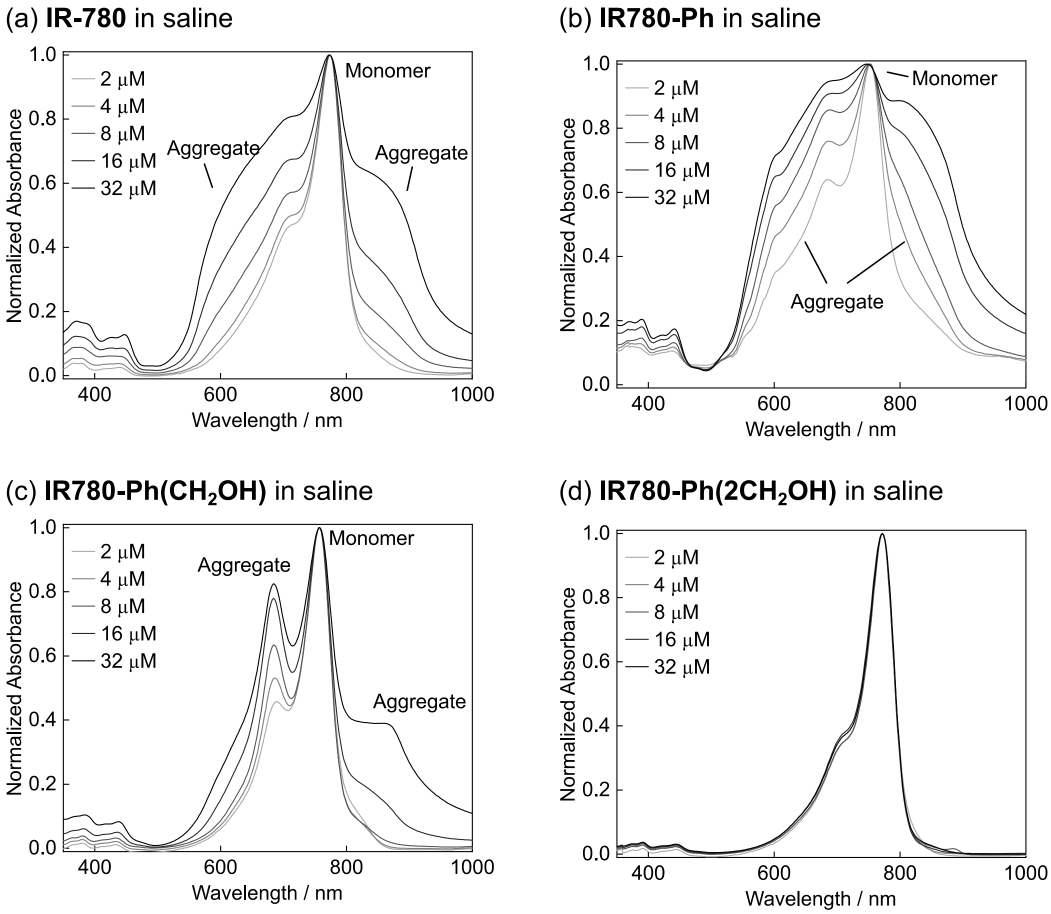

Figure S6 Reduction of aggregation by 4'-aryl modifications. (a-d) Normalized absorption spectra of increasing concentrations of (a) **IR-780**, (b) **IR780-Ph**, (c) **IR780-Ph(CH₂OH)**, and (d) **IR780-Ph(2CH₂OH)** in PBS. Absorbance smaller than 2 was measured through a 1 cm light path. Absorbance larger than 2 was measured through a 0.5 cm light path and doubled to represent absorbance at 1 cm.

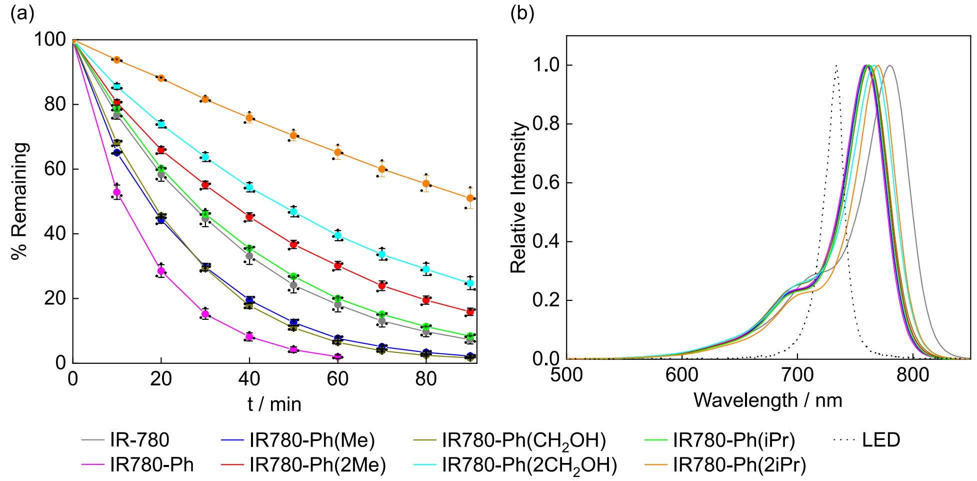

Figure S7 Photobleach experiment of **IR-780** and its derivatives. (a) Photobleach curves of dyes in 1:1 methanol/water solution under 730 nm LED illumination (6.8 mW/cm^2) determined by reduction of their absorption over time. Results are shown as mean ±s.d. (n = 3). (b) Comparison of absorption spectra of dyes in 1:1 methanol/water for photobleach experiment and the spectrum of LED light source.

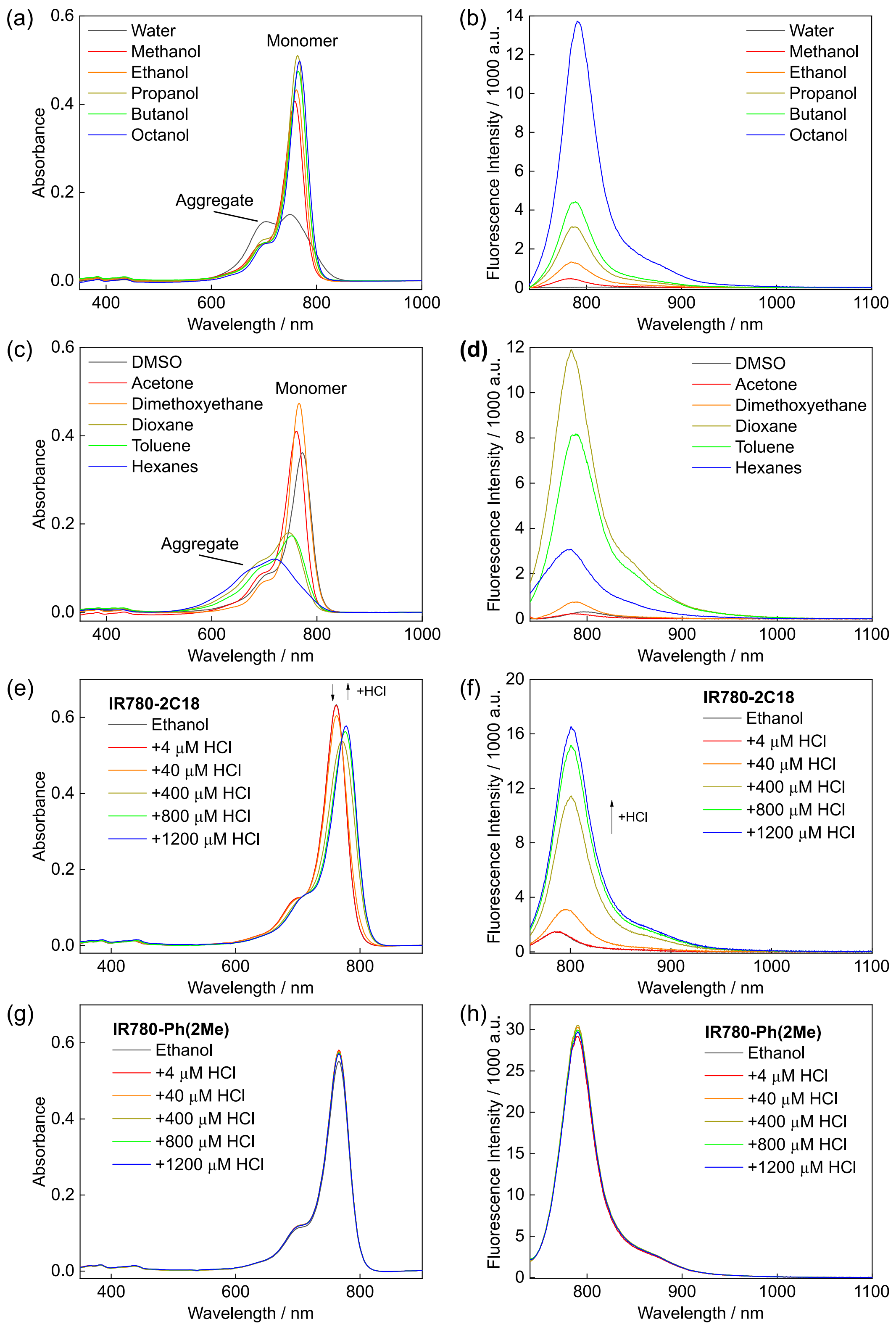

Figure S8 Absorption and emission of **IR780-2C18** in different conditions. (a-b) Absorption (a) and emission (b) spectra of 2 μM **IR780-2C18** in alcoholic solvents. (c-d) Absorption (c) and emission (d) spectra of **IR780-2C18** in solvents with varying polarity. (e-f) Absorption (e) and emission (f) spectra of **IR780-2C18** in ethanol with varying concentrations of HCl. (g-h) Absorption (g) and emission (h) spectra of **IR780-Ph** with varying concentrations of HCl for comparison. HCl stock solution (200 mM) was prepared by adding cold ethanol into acetyl chloride.

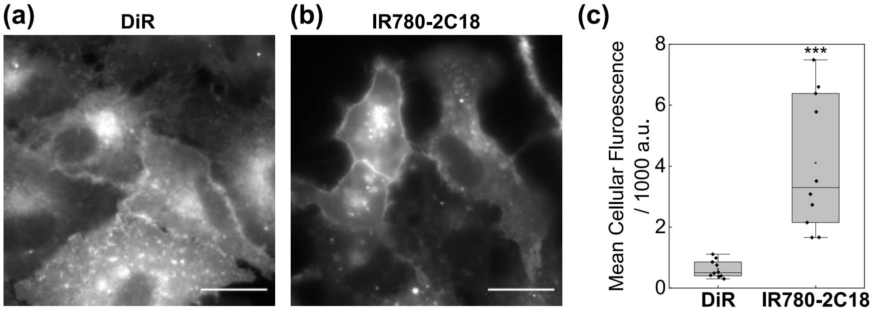

Figure S9 Comparison of membrane imaging using **DiR** and **IR780-2C18**. (a-b) Representative images of cell stained with 2.5 μM **DiR** (a) and **IR780-2C18** (b). The brightness and contrast in (a) and (b) are adjusted to show the staining of the cell. Scale-bar: 20 μm. (c) Mean fluorescence intensity in A549 cells after incubation with 2.5 μM **DiR** or **IR780-2C18** under same imaging condition after background subtraction. Data are calculated on 10 cells from four independent incubations for each dye. *** P≤0.001; two-tailed Student's *t*-test.

Table S1 Photophysical properties of Cy7 derivatives.

Compound	Solvent	$\lambda_{max,abs}$ / nm	ε_{max} / 10^5 $M^{-1}cm^{-1}$	$\lambda_{max,ems}$ / nm	Φ_F [a]
**IR-780**	Ethanol	784	2.17±0.03	806	0.208±0.007
	Water	753	1.51±0.06	777	0.109±0.004
**2a (IR780-Ph)**	Ethanol	762	2.37±0.05	784	0.37±0.02
	Water	774	1.41±0.02	795	0.051±0.001
**2b** [**IR780-Ph(Me)**]	Ethanol	764	2.73±0.07	787	0.37±0.03
**2c** [**IR780-Ph(iPr)**]	Ethanol	765	2.46±0.04	785	0.258±0.005
**2d** [**IR780-Ph(OMe)**]	Ethanol	764	2.46±0.09	788	0.34±0.02
**2e** [**IR780-Ph(CH2OH)**]	Ethanol	765	2.76±0.05	789	0.37±0.01
	Water	757	1.71±0.04	782	0.096±0.003
**2f** [**IR780-Ph(COOH)**]	Ethanol	762	2.05±0.04	784	0.34±0.01
	Water	753	1.18±0.03	777	0.118±0.003
**2g** [**IR780-Ph(CH2StBu)**]	Ethanol	767	2.56±0.07	791	0.33±0.02
**2h** [**IR780-Ph(2Me)**]	Ethanol	766	2.73±0.05	789	0.36±0.02
**2i** [**IR780-Ph(2iPr)**]	Ethanol	774	2.72±0.03	798	0.245±0.007
**2j** [**IR780-Ph(2CH2OAllyl)**]	Ethanol	768	2.34±0.04	794	0.38±0.01
**2k** [**IR780-Ph(2CH2OH)**]	Ethanol	768	2.47±0.04	794	0.39±0.01
	Water	762	1.95±0.02	785	0.113±0.003
**4** (**IR780-2C18**)	Ethanol	761	3.3±0.1	787	0.0150±0.0004
	Octanol	767	3.39±0.09	790	0.180±0.004

[a] ICG in ethanol (Φ_F = 0.132)[5,6] was used as a reference.

Table S2 Absorption and emission maxima of **3a-d** and their starting 4'-chloro dyes in dichloromethane.

Compound	$\lambda_{max,abs}$ / nm	$\lambda_{max,ems}$ / nm	Compound	$\lambda_{max,abs}$ / nm	$\lambda_{max,ems}$ / nm
**IR-775**	785	812	**3a** [**IR775-Ph(Me)**]	764	785
**IR-813**	825	856	**3b** [**IR813-Ph(Me)**]	801	826
**Chrom7**	975	996	**3c** [**Chrom7-Ph(Me)**]	952	982
**IR-1061**	1058	n.d.[a]	**3d** [**IR1061-Ph(Me)**]	1040	n.d.[a]
**IR-783**	782[b]	804[b]	**3e [IR783-Ph(Me)]**	762[b]	784[b]

[a] Not determined due to fluorometer wavelength limit

[b] Measured in methanol.

Synthetic Procedures

Synthetic materials and methods

Unless otherwise noted, all commercial reagents were used without further purification. All reactions utilizing air- or moisture-sensitive reagents were performed under an atmosphere of dry N_2. Dry solvents were purchased from Thermo Scientific Chemicals and stored over sieves under an atmosphere of dry N_2. Chemical reagents were purchased from Ambeed, Oakwood Chemicals and Thermo Scientific Chemicals. Heptamethine dyes were purchased from Thermo Scientific Chemicals (IR-780, IR-775), TCI America (IR-813) and Enamine (IR-1061). Chrom7,[7] (2-bromobenzyl)(*tert*-butyl)sulfane,[8] and 2-bromo-1,3-benzenedimethanol [9] were synthesized according to published procedures. ^{1}H NMR and ^{13}C NMR spectra were collected in $CDCl_3$, CD_3CN or MeOD at 25 °C on Bruker 400 MHz or 500 MHz spectrometers at the NMR Facility at the Department of Chemistry and Biochemistry in the University of Arkansas, Fayetteville. All chemical shifts in ^{1}H NMR and ^{13}C NMR are reported in the standard notation of ppm relative to residual solvent peak ($CDCl_3$ δH=7.26, δC=77.16; CD_3CN δH=1.94, δC=1.32; MeOD δH=3.31, δC=49.00; CD_2Cl_2 δH=5.32, δC=53.84). High resolution mass spectrometry was acquired on an IT-TOF (Shimadzu) at the University of Arkansas Statewide Mass Spectrometry Facility.

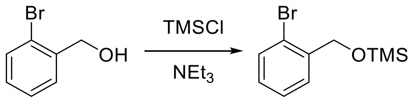

S1

((2-Bromo-3-methylbenzyl)oxy)trimethylsilane (**S1**): To a flask containing 2-bromophenylmethanol (200 mg, 1.1 mmol) and triethylamine (0.22 mL, 1.6 mmol) dissolved in CH_2Cl_2 (10 mL) was added trimethylsilyl chloride (0.16 mL, 1.3 mmol). The mixture was stirred for 2.5 h at room temperature and concentrated to dryness. The crude product was separated by column chromatography (1:50 ethyl acetate/hexanes) to give **S1** as a colorless liquid (274 mg, 99%), which is used without further purification. ^{1}H NMR (400 MHz, $CDCl_3$) δ 7.53 (d, J = 7.7 Hz, 1H), 7.50 (d, J = 8.1 Hz, 1H), 7.32 (t, J = 7.5 Hz, 1H), 7.12 (t, J = 7.5 Hz, 1H), 4.72 (s, 2H), 0.19 (s, 9H). This compound has also been characterized elsewhere.[10]

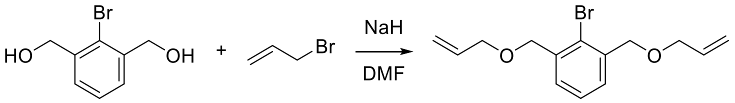

S2

1,3-Bis((allyloxy)methyl)-2-bromobenzene (**S2**): To a flask containing 2-bromo-1,3-benzenedimethanol (305 mg, 1.41 mmol) and NaH (60% dispersion in mineral oil, 281 mg, 7.03 mmol) under N_2 was added dry DMF (7 mL). The reaction was stirred at room temperature for 0.5 h, followed by the dropwise addition of allyl bromide (0.35 mL, 4.2 mmol). The reaction was further stirred for 1 h, quenched with addition of MeOH and H_2O, and extracted into ethyl acetate. The organic layer was washed with H_2O (×4) and saturated NaCl, dried (Na_2SO_4) and concentrated. The crude product was separated by column chromatography (1:50 ethyl acetate/hexanes) to give **S2** as a colorless liquid (417 mg, >99%). ^{1}H NMR (400 MHz, $CDCl_3$) δ 7.44 (d, J = 7.5 Hz, 2H), 7.33 (t, J = 7.7 Hz, 1H), 5.99 (ddd, J = 22.8, 10.8, 5.6 Hz, 2H), 5.36 (dq, J = 17.2, 1.7 Hz, 2H), 5.24 (dd, J = 10.4, 1.6 Hz, 2H), 4.61 (s, 4H), 4.12 (dt, J = 5.6, 1.5 Hz, 4H). ^{13}C NMR (101 MHz, $CDCl_3$) δ 138.16, 134.67, 127.98, 127.32, 122.84, 117.36, 71.84, 71.79.

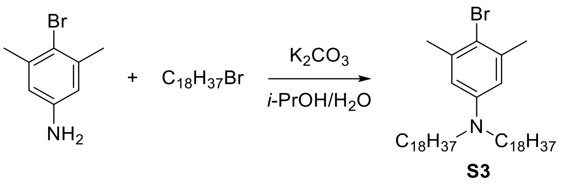

4-Bromo-3,5-dimethyl-*N*,*N*-dioctadecylaniline (**S3**): To a microwave vessel containing 4-bromo-3,5-dimethylaniline (0.20 g, 1.0 mmol), 1-bromooctadecane (1.67 g, 5.0 mmol) and K_2CO_3 (0.55 g, 4.0 mmol) was added 15 mL of 1:2 H_2O/isopropanol. The reaction was carried out in a CEM Discover SP Microwave reactor at 120℃ for 4h. The mixture was cooled, diluted with H_2O and extracted with CH_2Cl_2 (×5), dried (Na_2SO_4) and concentrated. The crude product was separated by column chromatography (hexanes) to give **S3** as a white solid (481 mg, 68%). ^1H NMR (500 MHz, $CDCl_3$) δ 6.38 (s, 2H), 3.20 (t, *J* = 7.6 Hz, 4H), 2.36 (s, 6H), 1.60 – 1.51 (m, 4H), 1.36 – 1.22 (m, 64H), 0.90 (t, *J* = 6.9 Hz, 6H). ^{13}C NMR (126 MHz, $CDCl_3$) δ 147.12, 138.48, 113.07, 112.27, 51.23, 32.11, 29.88, 29.80, 29.67, 29.53, 27.38, 27.34, 24.49, 22.85, 14.24. HRMS (ESI^+) calcd 704.5703, found 704.5695for $C_{44}H_{83}BrN^+$ ($M+H^+$).

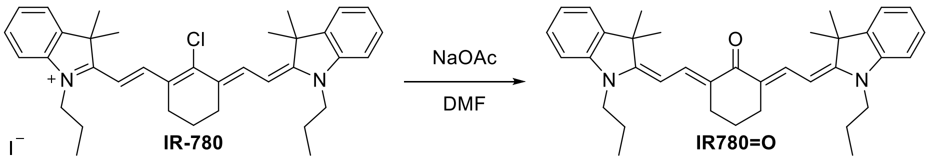

2,6-Bis(2-(3,3-dimethyl-1-propylindolin-2-ylidene)ethylidene)cyclohexan-1-one (**IR780=O**): To a flask containing **IR-780** iodide (500 mg, 0.75 mmol) and sodium acetate (184 mg, 2.25 mmol) was added dry DMF (10 mL) followed by three freeze-pump-thaw cycles. The reaction was then stirred at 80°C under N_2 for 3 h. The mixture was diluted in ethyl acetate, washed with H_2O (×4) and saturated NaCl, dried (Na_2SO_4) and concentrated. The crude product was separated by column chromatography (1:7.5 ethyl acetate/hexanes) to give **IR-780=O** as a dark red solid (365 mg, 93%). ^1H NMR (400 MHz, $CDCl_3$) δ 8.17 (d, *J* = 13.2 Hz, 2H), 7.26 – 7.13 (m, 4H), 6.90 (t, *J* = 7.4 Hz, 2H), 6.68 (d, *J* = 8.0 Hz, 2H), 5.46 (d, *J* = 13.2 Hz, 2H), 3.64 (t, *J* = 7.4 Hz, 4H), 2.61 (t, *J* = 6.2 Hz, 4H), 1.91 – 1.83 (m, 2H), 1.76 (h, *J* = 7.5 Hz, 4H), 1.67 (s, 12H), 1.01 (t, *J* = 7.4 Hz, 6H). ^{13}C NMR (101 MHz, $CDCl_3$) δ 186.45, 162.51, 144.48, 139.77, 132.97, 127.68, 126.58, 121.84, 120.48, 106.81, 92.63, 46.63, 44.21, 28.88, 25.93, 22.68, 19.84, 11.84. HRMS (ESI^+) calcd 521.3526, found 521.3530 for $C_{36}H_{45}N_2O^+$ ($M+H^+$).

General procedure A: preparation of heptamethine=O. The preparation is adapted from previous reports.[11,12] Specifically, heptamethine-Cl (1.0 equiv.), *N*-hydroxysuccinimide (NHS, 3.0 equiv.) and *N*,*N*-diisopropylethylamine (3.0 equiv.) were dissolved in DMF (2 mL) and stirred at room temperature until complete conversion of the starting heptamethine-Cl as determined by TLC. The mixture was then diluted in ethyl acetate, washed with H_2O (×4) and saturated NaCl, dried (Na_2SO_4) and concentrated. The crude product was separated by column chromatography (1:200 methanol/CH_2Cl_2) to give heptamethine=O.

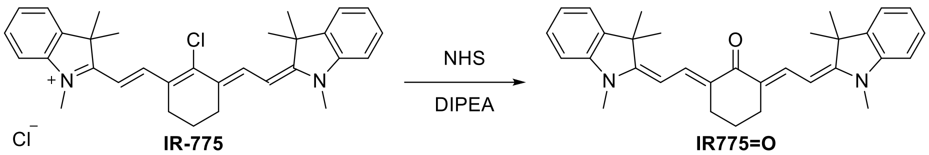

2,6-Bis(2-(1,3,3-trimethylindolin-2-ylidene)ethylidene)cyclohexan-1-one (**IR775=O**): Following General Procedure A, **IR-775** chloride (50 mg, 0.096 mmol) was reacted with NHS (33 mg, 0.29 mmol) and DIPEA (50 µL, 0.29 mmol) in DMF (2 mL) for 18 h to give **IR775=O** as a dark red-orange solid (31 mg, 70%). ^{1}H NMR (400 MHz, $CDCl_3$) δ 8.18 (d, *J* = 13.2 Hz, 2H), 7.18 (t, *J* = 7.3 Hz, 4H), 6.90 (t, *J* = 7.4 Hz, 2H), 6.68 (d, *J* = 7.9 Hz, 2H), 5.41 (d, *J* = 13.2 Hz, 2H), 3.21 (s, 6H), 2.62 (t, *J* = 6.2 Hz, 4H), 1.87 (p, *J* = 6.4 Hz, 2H), 1.68 (s, 12H). ^{13}C NMR (101 MHz, $CDCl_3$) δ 186.60, 163.33, 144.68, 139.70, 132.91, 127.73, 126.93, 121.80, 120.59, 106.52, 92.65, 46.52, 29.36, 28.78, 25.95, 22.63. HRMS (ESI$^+$) calcd 465.2900, found 465.2928 for $C_{32}H_{37}N_2O^+$ (M+H$^+$).

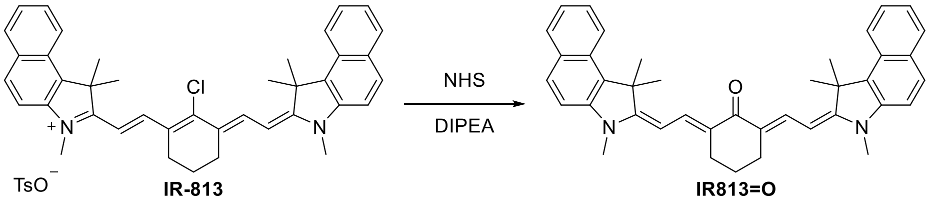

2,6-Bis(2-(1,1,3-trimethyl-1,3-dihydro-2H-benzo[e]indol-2-ylidene)ethylidene)cyclohexan-1-one (**IR813=O**): Following General Procedure A, **IR-813** *p*-toluenesulfonate (100 mg, 0.152 mmol) was reacted with NHS (46 mg, 0.40 mmol) and DIPEA (69 µL, 0.40 mmol) for 1 h to give **IR813=O** as a deep red to dark magenta solid (39.5 mg, 54%). ^{1}H NMR (400 MHz, $CDCl_3$) δ 8.37 (d, *J* = 13.2 Hz, 2H), 8.07 (d, *J* = 8.6 Hz, 2H), 7.83 (d, *J* = 8.3 Hz, 2H), 7.77 (d, *J* = 8.8 Hz, 2H), 7.50 (ddd, *J* = 8.3, 6.7, 1.3 Hz, 2H), 7.33 – 7.25 (m, 2H), 7.10 (d, *J* = 8.7 Hz, 2H), 5.48 (d, *J* = 13.3 Hz, 2H), 3.33 (s, 6H), 2.69 (t, *J* = 5.5 Hz, 4H), 2.04 (s, 12H), 1.97 – 1.90 (m, 2H). ^{13}C NMR (101 MHz, $CDCl_3$) δ 186.53, 165.26, 141.92, 132.81, 130.04, 129.85, 129.82, 129.48, 129.07, 126.93, 126.81, 122.60, 121.93, 109.10, 92.37, 48.54, 29.61, 28.05, 25.99, 22.69. HRMS (ESI$^+$) calcd 565.3213, found 565.3235 for $C_{40}H_{41}N_2O^+$ (M+H$^+$).

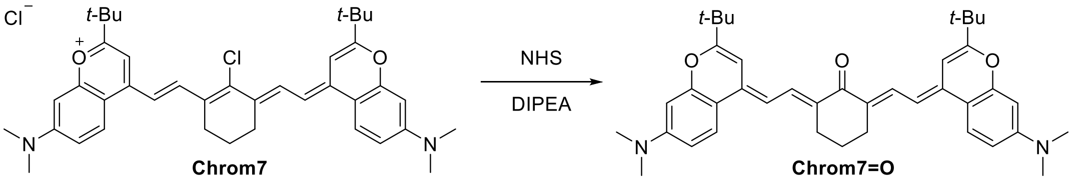

2,6-Bis(2-(2-(*tert*-butyl)-7-(dimethylamino)-4*H*-chromen-4-ylidene)ethylidene)cyclohexan-1-one (**Chrom7=O**): Following General Procedure A, **Chrom7** chloride (40 mg, 0.061 mmol) was reacted with NHS (23 mg, 0.20 mmol) and DIPEA (32 µL, 0.18 mmol) for 2 h to give **Chrom7=O** as a dark purple solid (10 mg, 28 %). ^{1}H NMR (400 MHz, $CDCl_3$) δ 8.03 (d, *J* = 12.8 Hz, 2H), 7.63 (d, *J* = 9.0 Hz, 2H), 6.58 (dd, *J* = 9.0, 2.6 Hz, 2H), 6.49 (s, 2H), 6.39 – 6.28 (m, 4H), 3.01 (s, 12H), 2.74 (t, *J* = 5.9 Hz, 4H), 1.87 (p, *J* = 5.9 Hz, 2H), 1.28 (s, 18H). ^{13}C NMR (101 MHz, $CDCl_3$) δ 163.37, 154.01, 152.03, 135.45, 131.76, 130.44, 123.79, 111.21, 109.88, 104.82, 98.94, 97.89, 40.36, 35.86, 28.18, 26.66, 22.69. HRMS (ESI$^+$) calcd 605.3738, found 605.3730 for $C_{40}H_{49}N_2O_3^+$ (M+H$^+$).

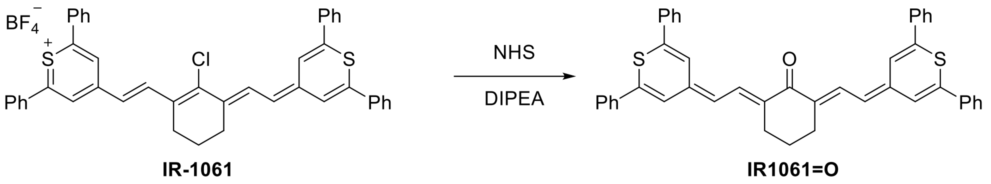

2,6-Bis(2-(2,6-diphenyl-4*H*-thiopyran-4-ylidene)ethylidene)cyclohexan-1-one (**IR1061=O**): Following General Procedure A, **IR-1061** tetrafluoroborate (95 mg, 0.13 mmol) was reacted with NHS (48 mg, 0.42 mmol) and DIPEA (66 µL, 0.38 mmol) for 2 h to give **IR1061=O** as a dark brown solid (50 mg, 61%). ^{1}H NMR (400 MHz, $CDCl_3$) δ 8.00 (d, *J* = 12.8 Hz, 2H), 7.69 – 7.53 (m, 8H), 7.50 – 7.36 (m, 14H), 6.86 (s, 2H), 6.08 (d, *J* = 13.0 Hz, 2H), 2.68 (t, *J* = 6.0 Hz, 4H), 1.85 (p, *J* = 6.0 Hz, 2H). ^{13}C NMR (101 MHz, $CDCl_3$) δ 187.77, 140.00, 139.87, 137.99, 137.64, 137.58, 132.49, 130.76, 129.66, 129.43, 129.06, 126.43, 126.37, 126.13, 119.57, 118.58, 26.58, 22.30. HRMS (ESI$^+$) calcd 643.2124, found 643.2086 for $C_{44}H_{35}OS_2^+$ (M+H$^+$).

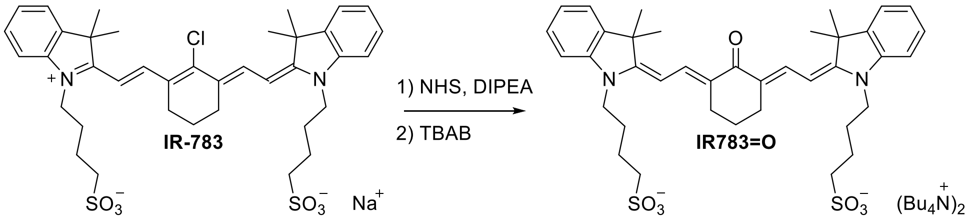

Tetrabutylammonium 4,4'-(((2-oxocyclohexane-1,3-diylidene)bis(ethane-2,1-diylidene))bis(3,3-dimethylindoline-1-yl-2-ylidene))bis(butane-1-sulfonate) (**IR783=O** tetrabutylammonium salt): Following Gneral Procedure B, **IR783** (80 mg, 0.11 mmol) was reacted with NHS (37 mg, 0.32 mmol) and DIPEA (56 µL, 0.32 mmol) in DMF (3 mL) for 3.5 h to afford a red orange solution. The mixture was diluted with H_2O (6 mL) and loaded onto preparative HPLC (Phenomenex Kinetex 5 µm phenyl-hexyl, 250×21.2 mm). The column was flushed three times with tetrabutylammonium bromide solution (7 mL, 0.2 M dissolved in 30% methanol in H_2O) and separated with a gradient of 30% – 90% methanol to give **IR783=O** tetrabutylammonium salt as a dark, red orange solid (84 mg, 66%). ^{1}H NMR (500 MHz, MeOD) δ 8.19 (d, *J* = 12.7 Hz, 2H), 7.26 (d, *J* = 7.5 Hz, 2H), 7.21 (t, *J* = 7.8 Hz, 2H), 7.04 – 6.81 (m, 4H), 5.61 (d, *J* = 13.3 Hz, 2H), 3.83 (t, *J* = 6.9 Hz, 4H), 3.23 (t, *J* = 8.6 Hz, 16H), 2.87 (t, *J* = 7.6 Hz, 4H), 2.62 (t, *J* = 6.4 Hz, 4H), 2.03 – 1.81 (m, 10H), 1.69 – 1.60 (m, 28H), 1.41 (h, *J* = 7.7 Hz, 16H), 1.01 (t, *J* = 7.6 Hz, 24H). ^{13}C NMR (126 MHz, MeOD) δ 188.52, 165.19, 145.28, 140.81, 136.15, 129.01, 127.14, 122.74, 122.16, 108.65, 94.00, 59.57, 52.30, 47.90, 43.33, 29.10, 26.69, 26.56, 24.79, 23.82, 23.72, 20.67, 13.91. HRMS (ESI$^-$) calcd 707.2830, found 707.2829 for $C_{38}H_{47}N_2O_7S_2^-$ [M^{2-}+H^+].

General procedure B: preparation of *meso*-substituted heptamethine dyes. Unless otherwise noted, aryl bromide (0.51 mmol) was dissolved in dry THF (1 mL) and cooled to −84 °C. To this solution was added *n*-BuLi (2.3 M in cyclohexane/hexanes, 147 µL, 0.34 mmol). The mixture was stirred for 10 min at this temperature. A red to orange solution of keto-heptamethine (0.042 mmol) in dry THF (1 mL) was added dropwise. The mixture was allowed to warm up to room temperature and stirred for 30 min. The yellow or orange reaction mixture was quenched by adding 1:10 HCl which resulted in a rapid color change into dark green. The mixture was diluted in H_2O and extracted with CH_2Cl_2 (×4) and dried (Na_2SO_4). The crude product was purified by column chromatography (1:30 to 1:20 methanol / CH_2Cl_2).

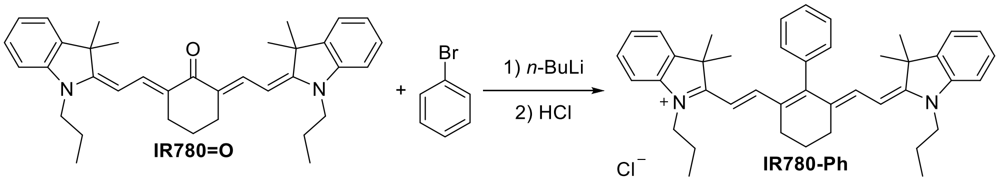

2-(2-(6-(2-(3,3-Dimethyl-1-propylindolin-2-ylidene)ethylidene)-3,4,5,6-tetrahydro-[1,1'-biphenyl]-2-yl)vinyl)-3,3-dimethyl-1-propyl-3H-indol-1-ium chloride (**IR780-Ph**): Following General Procedure B, **IR780=O** (22 mg, 0.042 mmol) was reacted with bromobenzene (80 mg, 0.51 mmol) and *n*-BuLi (2.3 M, 0.15 mL, 0.34 mmol) to give **IR780-Ph** as a green solid (27 mg, >99%) ^{1}H NMR (400 MHz, MeOD with a few drops of $CDCl_3$) δ 7.65 – 7.52 (m, 3H), 7.33 (td, *J* = 7.6, 1.2 Hz, 2H), 7.29 – 7.21 (m, 6H), 7.16 (t, *J* = 7.0 Hz, 4H), 6.11 (d, *J* = 14.0 Hz, 2H), 4.01 (t, *J* = 7.3 Hz, 4H), 2.70 (t, *J* = 6.3 Hz, 4H), 2.07 (p, *J* = 6.3 Hz, 2H), 1.84 (h, *J* = 7.4 Hz, 4H), 1.17 (s, 12H), 1.02 (t, *J* = 7.4 Hz, 6H). ^{13}C NMR (101 MHz, MeOD with a few drops of $CDCl_3$) δ 172.91, 163.77, 149.34, 143.28, 141.61, 139.86, 132.19, 130.32, 129.50, 129.41, 129.13, 125.68, 122.94, 111.40, 100.49, 49.53, 46.22, 28.03, 25.43, 22.10, 21.45, 11.76. HRMS (ESI$^+$) calcd 581.3890, found 581.3887 for $C_{42}H_{49}N_2^+$ (M^+).

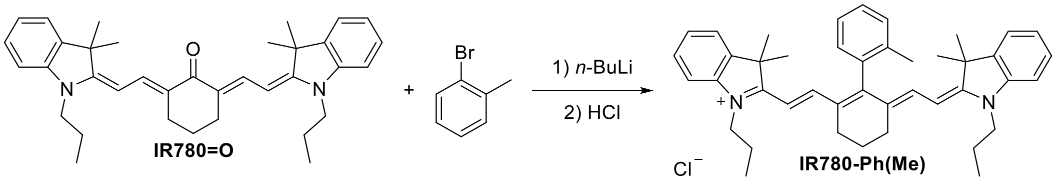

2-(2-(6-(2-(3,3-Dimethyl-1-propylindolin-2-ylidene)ethylidene)-2'-methyl-3,4,5,6-tetrahydro-[1,1'-biphenyl]-2-yl)vinyl)-3,3-dimethyl-1-propyl-3H-indol-1-ium chloride [**IR780-Ph(Me)**]: Following General Procedure B, **IR780=O** (22 mg, 0.042 mmol) was reacted with 2-bromotoluene (87 mg, 0.51 mol) and *n*-BuLi (2.3 M, 0.15 mL, 0.34 mmol) to give **IR780-Ph(Me)** as a green solid film (23 mg, 93%). ^{1}H NMR (400 MHz, MeOD) δ 7.51 – 7.40 (m, 3H), 7.39 – 7.28 (m, 4H), 7.25 – 7.15 (m, 6H), 7.09 (d, *J* = 7.3 Hz, 1H), 6.19 (d, *J* = 14.0 Hz, 2H), 4.05 (t, *J* = 7.3 Hz, 4H), 2.73 (t, *J* = 6.3 Hz, 4H), 2.14 (s, 2H), 2.07 (p, *J* = 6.2 Hz, 2H), 1.83 (h, *J* = 7.4 Hz, 4H), 1.16 (s, 6H), 1.13 (s, 6H), 1.01 (t, *J* = 7.4 Hz, 6H). ^{13}C NMR (101 MHz, MeOD) δ 172.05, 161.89, 147.06, 142.37, 140.75, 138.23, 135.93, 130.43, 130.33, 129.15, 128.37, 126.11, 124.61, 121.97, 115.00, 110.49, 99.57, 53.48, 48.52, 45.00, 26.86, 26.66, 24.12, 21.22, 20.35, 10.33. HRMS (ESI$^+$) calcd 595.4048, found 595.4047 for $C_{43}H_{51}N_2^+$ (M^+).

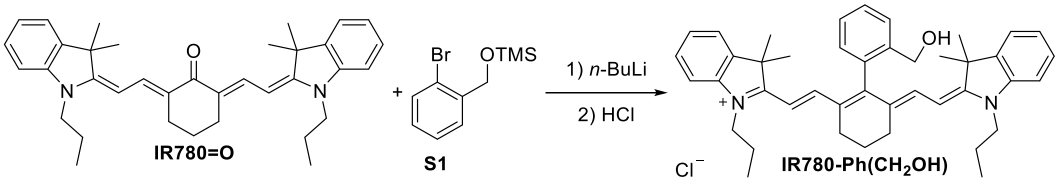

2-(2-(6-(2-(3,3-Dimethyl-1-propylindolin-2-ylidene)ethylidene)-2'-(hydroxymethyl)-3,4,5,6-tetrahydro-[1,1'-biphenyl]-2-yl)vinyl)-3,3-dimethyl-1-propyl-3H-indol-1-ium chloride [**IR780-Ph(CH_2OH)**]: Following General Procedure B, **IR780=O** (22 mg, 0.042 mmol) was reacted with ((2-bromo-3-methylbenzyl)oxy)trimethylsilane (131 mg, 0.51 mol) and *n*-BuLi (2.3 M, 0.15 mL, 0.34 mmol) to give **IR780-Ph(CH_2OH)** as a green solid film

(21 mg, 78%). ^{1}H NMR (400 MHz, MeOD) δ 7.80 (d, *J* = 7.7 Hz, 1H), 7.61 (t, *J* = 7.6 Hz, 1H), 7.51 (t, *J* = 7.4 Hz, 1H), 7.38 – 7.29 (m, 4H), 7.27 – 7.14 (m, 6H), 7.11 (d, *J* = 7.4 Hz, 1H), 6.19 (d, *J* = 14.1 Hz, 2H), 4.45 (s, 2H), 4.05 (t, *J* = 7.4 Hz, 4H), 2.73 (s, 4H), 2.07 (p, *J* = 6.5 Hz, 2H), 1.82 (h, *J* = 7.5 Hz, 4H), 1.17 (s, 6H), 1.14 (s, 6H), 1.01 (t, *J* = 7.4 Hz, 6H). ^{13}C NMR (101 MHz, MeOD) δ 173.52, 148.67, 143.74, 142.21, 141.13, 137.89, 132.04, 130.54, 129.76, 129.67, 128.60, 128.49, 125.94, 123.30, 111.83, 101.01, 62.00, 49.93, 46.34, 28.27, 28.02, 25.52, 22.59, 21.72, 11.65. HRMS (ESI$^+$) calcd 611.3996, found 611.4005 for $C_{43}H_{51}N_2O^+$ (M^+).

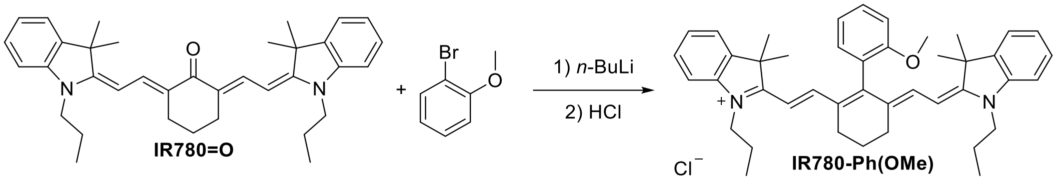

2-(2-(6-(2-(3,3-Dimethyl-1-propylindolin-2-ylidene)ethylidene)-2'-methoxy-3,4,5,6-tetrahydro-[1,1'-biphenyl]-2-yl)vinyl)-3,3-dimethyl-1-propyl-3H-indol-1-ium chloride [**IR780-Ph(OMe)**]: Following General Procedure B, **IR780=O** (22 mg, 0.042 mmol) was reacted with 2-bromoanisole (95 mg, 0.51 mol) and *n*-BuLi (2.3 M, 0.15 mL, 0.34 mmol) to give **IR780-Ph(OMe)** as a green solid film (25 mg, 98%). ^{1}H NMR (400 MHz, MeOD) δ 7.59 (t, *J* = 8.0 Hz, 1H), 7.38 – 7.26 (m, 7H), 7.25 – 7.13 (m, 5H), 7.08 (dd, *J* = 7.4, 1.7 Hz, 1H), 6.16 (d, *J* = 14.1 Hz, 2H), 4.04 (t, *J* = 7.4 Hz, 4H), 3.75 (s, 3H), 2.70 (s, 4H), 2.14 – 2.06 (m, 2H), 1.82 (h, *J* = 7.5 Hz, 4H), 1.21 (s, 6H), 1.16 (s, 6H), 1.02 (t, *J* = 7.4 Hz, 6H). ^{13}C NMR (101 MHz, MeOD) δ 172.92, 157.89, 148.61, 143.81, 142.08, 131.93, 131.32, 129.67, 128.43, 125.80, 123.30, 122.10, 112.60, 111.72, 100.81, 56.27, 49.81, 46.28, 28.16, 27.96, 25.56, 22.47, 21.69, 11.67. HRMS (ESI$^+$) calcd 611.3996, found 611.4004 for $C_{43}H_{51}N_2O^+$ (M^+).

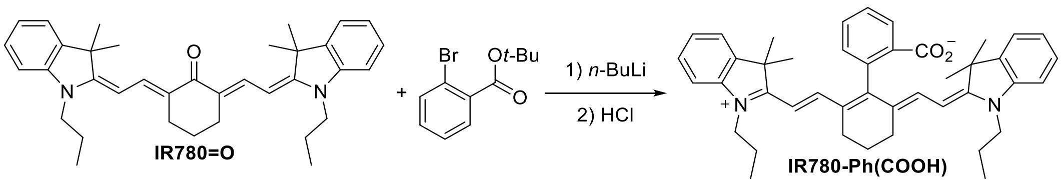

2-(2-(2'-Carboxy-6-(2-(3,3-dimethyl-1-propylindolin-2-ylidene)ethylidene)-3,4,5,6-tetrahydro-[1,1'-biphenyl]-2-yl)vinyl)-3,3-dimethyl-1-propyl-3H-indol-1-ium chloride [**IR780-Ph(COOH)**]: Following General Procedure B, **IR780=O** (22 mg, 0.042 mmol) was reacted with *tert*-butyl 2-bromobenzoate (130 mg, 0.51 mol) and *n*-BuLi (2.3 M, 0.15 mL, 0.34 mmol) to give **IR780-Ph(COOH)** as a green solid (13 mg, 50%). ^{1}H NMR (400 MHz, MeOD) δ 8.20 (d, *J* = 7.7 Hz, 1H), 7.70 (dt, *J* = 27.0, 7.6 Hz, 2H), 7.37 – 7.29 (m, 4H), 7.26 – 7.11 (m, 7H), 6.15 (d, *J* = 14.0 Hz, 2H), 4.02 (t, *J* = 7.3 Hz, 4H), 2.70 (t, *J* = 6.2 Hz, 4H), 2.17 – 1.96 (m, 2H), 1.81 (h, *J* = 7.3 Hz, 4H), 1.19 (s, 6H), 1.12 (s, 6H), 1.00 (t, *J* = 7.4 Hz, 6H). ^{13}C NMR (101 MHz, MeOD) δ 172.97, 164.94, 148.86, 143.83, 142.12, 141.00, 132.65, 132.58, 132.15, 131.90, 129.63, 125.73, 123.28, 111.65, 100.71, 49.80, 46.24, 28.30, 27.97, 25.78, 22.30, 21.66, 11.64. HRMS (ESI$^+$) calcd 625.3789, found 625.3784 for $C_{43}H_{49}N_2O_2^+$ ($M+H^+$).

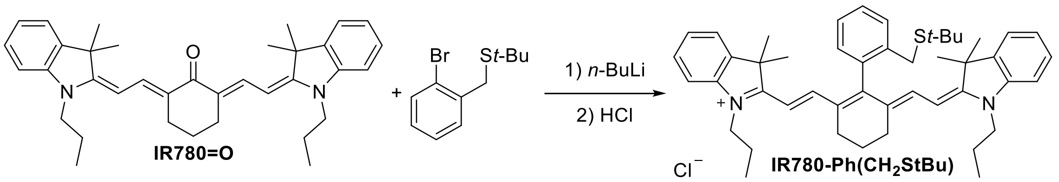

2-(2-(2'-((*tert*-Butylthio)methyl)-6-(2-(3,3-dimethyl-1-propylindolin-2-ylidene)ethylidene)-3,4,5,6-tetrahydro-[1,1'-biphenyl]-2-yl)vinyl)-3,3-dimethyl-1-propyl-3H-indol-1-ium chloride [**IR780-Ph(CH_2StBu)**]: Following General Procedure B, **IR780=O** (22 mg, 0.042 mmol) was reacted with (2-bromobenzyl)(*tert*-butyl)sulfane (133 mg, 0.51 mol) and *n*-BuLi (2.3 M, 0.15 mL, 0.34 mmol) to give **IR780-Ph(CH_2StBu)** as a green solid film (29 mg, 95%). ^{1}H NMR (400 MHz, MeOD) δ 7.62 (dd, *J* = 7.6, 1.5 Hz, 1H), 7.51 (dtd, *J* = 19.7, 7.4, 1.5 Hz, 2H), 7.39 – 7.30 (m, 4H), 7.29 – 7.10 (m, 7H), 6.20 (d, *J* = 14.1 Hz, 2H), 4.07 (t, *J* = 7.3 Hz, 4H), 3.63 (s, 2H), 2.85 – 2.64 (m, 4H), 2.18 – 2.00 (m, 2H), 1.83 (h, *J* = 7.4 Hz, 4H), 1.28 – 1.21 (m, 15H), 1.17 (s, 6H), 1.01 (t, *J* = 7.4 Hz, 6H). ^{13}C NMR (101 MHz, MeOD) δ 173.51, 161.64, 149.13, 143.75, 142.19, 139.52, 138.01, 132.16, 131.83, 131.20, 129.90, 129.66, 128.44, 125.94, 123.30, 111.83, 100.97, 49.98, 46.33, 43.71, 31.16, 30.94, 28.50, 28.05, 25.69, 22.62, 21.74, 11.67. HRMS (ESI$^+$) calcd 683.4393, found 683.4369 for $C_{47}H_{59}N_2S^+$ (M^+).

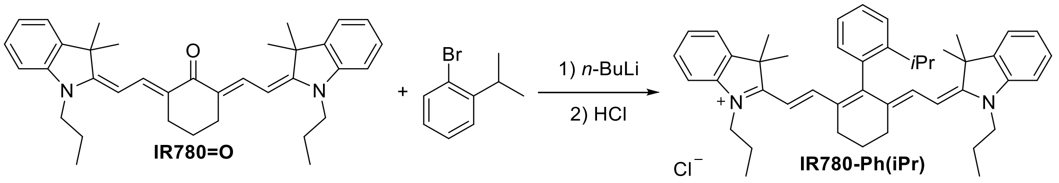

2-(2-(6-(2-(3,3-dimethyl-1-propylindolin-2-ylidene)ethylidene)-2'-isopropyl-3,4,5,6-tetrahydro-[1,1'-biphenyl]-2-yl)vinyl)-3,3-dimethyl-1-propyl-3H-indol-1-ium chloride [**IR780-Ph(iPr)**]: Following General Procedure B, **IR780=O** (22 mg, 0.042 mmol) was reacted with 1-bromo-2-isopropylbenzene (101 mg, 0.51 mmol) and *n*-BuLi (2.3 M, 0.15 mL, 0.34 mmol) to give **IR780-Ph(iPr)** as a green solid film (21 mg, 75%). ^{1}H NMR (500 MHz, $CDCl_3$) δ 7.50 (d, *J* = 3.0 Hz, 2H), 7.39 – 7.24 (m, 3H), 7.19 – 7.10 (m, 6H), 7.06 (d, *J* = 7.7 Hz, 2H), 7.00 (d, *J* = 7.2 Hz, 1H), 6.06 (d, *J* = 14.0 Hz, 2H), 4.01 (t, *J* = 6.4 Hz, 4H), 2.83 – 2.51 (m, 5H), 2.19 – 2.12 (m, 2H), 1.83 (q, *J* = 7.0 Hz, 4H), 1.15 (s, 6H), 1.08 (d, *J* = 6.1 Hz, 12H), 1.01 (t, *J* = 7.2 Hz, 6H). ^{13}C NMR (126 MHz, $CDCl_3$) δ 172.00, 162.16, 148.38, 147.56, 142.70, 141.02, 137.12, 132.39, 129.55, 129.06, 128.76, 126.47, 125.90, 124.94, 122.06, 110.83, 100.32, 48.85, 46.33, 30.67, 29.74, 28.16, 27.79, 25.15, 24.53, 21.59, 20.95, 11.59. HRMS (ESI$^+$) calcd 623.4360, found 623.4362 for $C_{45}H_{55}N_2^+$ (M^+).

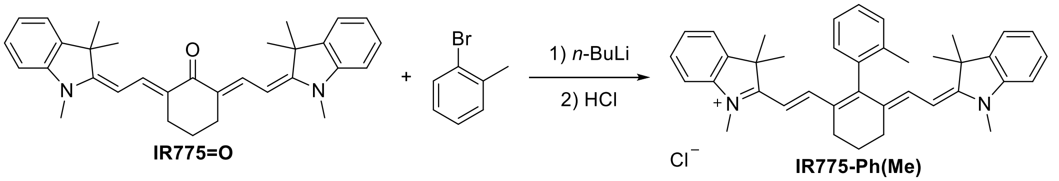

1,3,3-Trimethyl-2-(2-(2'-methyl-6-(2-(1,3,3-trimethylindolin-2-ylidene)ethylidene)-3,4,5,6-tetrahydro-[1,1'-biphenyl]-2-yl)vinyl)-3H-indol-1-ium chloride [(**IR775-Ph(Me)**]: Following General Procedure B, **IR775=O** (18 mg, 0.038 mmol) was reacted with 2-bromotulene (78 mg, 0.46 mmol) and *n*-BuLi (2.3 M, 0.13 mL, 0.31 mmol) to give **IR775-Ph(Me)** as a green solid (17 mg, 80%). ^{1}H NMR (400 MHz, $CDCl_3$) δ 7.47 – 7.27 (m, 5H), 7.19 – 7.02 (m, 9H), 6.07 (d, *J* = 14.0 Hz, 2H), 3.60 (s, 6H), 2.69 (s, 4H), 2.08 (s, 3H), 2.08 – 1.98 (m, 2H), 1.10 (d,

J = 4.7 Hz, 12H). ^{13}C NMR (101 MHz, $CDCl_3$) δ 172.11, 162.35, 147.19, 142.87, 140.65, 138.06, 136.02, 131.46, 130.41, 129.31, 128.74, 128.58, 126.23, 124.88, 121.97, 110.49, 100.26, 48.57, 31.80, 27.74, 27.57, 24.72, 21.32, 18.90. HRMS (ESI$^+$) calcd 539.3421, found 539.3418 for $C_{39}H_{43}N_2^+$ (M$^+$).

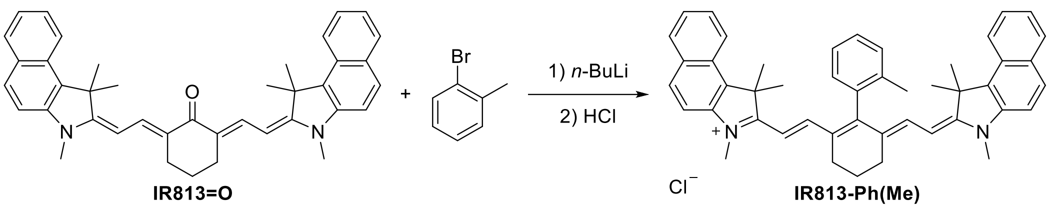

1,1,3-Trimethyl-2-(2-(2'-methyl-6-(2-(1,1,3-trimethyl-1,3-dihydro-2*H*-benzo[e]indol-2-ylidene)ethylidene)-3,4, 5,6-tetrahydro-[1,1'-biphenyl]-2-yl)vinyl)-1H-benzo[e]indol-3-ium chloride [**IR775-Ph(Me)**]: Following General Procedure B, **IR813=O** (25 mg, 0.045 mmol) was reacted with 2-bromotulene (92 mg, 0.54 mmol) and *n*-BuLi (2.3 M, 0.16 mL, 0.36 mmol) to give **IR813-Ph(Me)** as a yellowish-green solid (20 mg, 67%). ^{1}H NMR (400 MHz, $CDCl_3$) δ 8.00 – 7.83 (m, 6H), 7.62 – 7.36 (m, 9H), 7.20 (d, J = 14.1 Hz, 2H), 7.11 (d, J = 7.5 Hz, 1H), 6.10 (d, J = 14.1 Hz, 2H), 3.72 (s, 6H), 2.73 (t, J = 6.6 Hz, 4H), 2.15 (s, 3H), 2.12 – 2.02 (m, 2H), 1.42 (s, 12H). ^{13}C NMR (101 MHz, $CDCl_3$) δ 173.51, 161.67, 146.21, 140.25, 138.23, 136.07, 133.10, 131.80, 131.31, 130.70, 130.47, 130.19, 129.37, 128.73, 128.02, 127.62, 126.31, 124.83, 121.99, 110.70, 99.87, 50.35, 32.12, 27.32, 27.12, 24.75, 21.39, 18.96. HRMS (ESI$^+$) calcd 639.3734, found 639.3698 for $C_{47}H_{47}N_2^+$ (M$^+$).

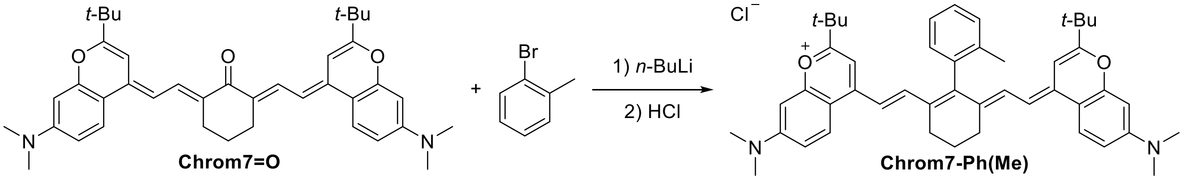

2-(tert-Butyl)-4-(2-(6-(2-(2-(tert-butyl)-7-(dimethylamino)-4*H*-chromen-4-ylidene)ethylidene)-2'-methyl-3,4,5, 6-tetrahydro-[1,1'-biphenyl]-2-yl)vinyl)-7-(dimethylamino)chromenylium chloride [**Chrom7-Ph(Me)**]: Following General Procedure B, **Chrom7=O** (10 mg, 0.017 mmol) was reacted with 2-bromotulene (35 mg, 0.20 mmol) and *n*-BuLi (2.3 M, 0.06 mL, 0.14 mmol) to give **Chrom7-Ph(Me)** as a dark purple solid (7.6 mg, 63%). ^{1}H NMR (400 MHz, MeOD) δ 7.92 (d, J = 9.3 Hz, 2H), 7.49 – 7.33 (m, 3H), 7.13 (d, J = 7.3 Hz, 1H), 7.06 (d, J = 13.7 Hz, 2H), 6.94 (dd, J = 9.3, 2.6 Hz, 2H), 6.85 (d, J = 13.8 Hz, 2H), 6.54 (d, J = 2.5 Hz, 2H), 6.05 (s, 2H), 3.11 (s, 12H), 2.13 (s, 3H), 2.11 – 1.98 (m, 2H), 1.20 (s, 18H). ^{13}C NMR (101 MHz, MeOD) δ 169.35, 158.75, 156.31, 154.49, 145.27, 141.95, 137.98, 133.39, 130.51, 129.66, 127.93, 125.29, 125.18, 117.59, 112.75, 111.51, 109.74, 98.37, 96.89, 39.02, 36.01, 26.74, 24.94, 21.44, 18.14. HRMS (ESI$^+$) calcd 679.4258, found 679.4250 for $C_{47}H_{55}N_2O_2^+$ (M$^+$).

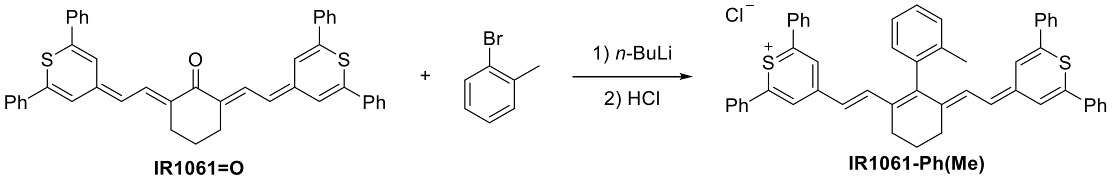

4-(2-(6-(2-(2,6-diphenyl-4*H*-thiopyran-4-ylidene)ethylidene)-2'-methyl-3,4,5,6-tetrahydro-[1,1'-biphenyl]-2-yl)

vinyl)-2,6-diphenylthiopyrylium chloride [**IR1061-Ph(Me)**]: Following General Procedure B, **IR1061=O** (28 mg, 0.043 mmol) was reacted with 2-bromotulene (88 mg, 0.52 mmol) and *n*-BuLi (2.3 M, 0.15 mL, 0.35 mmol) to give **IR1061-Ph(Me)** as a dark purple solid (17 mg, 56%). ^{1}H NMR (400 MHz, $CDCl_3$ and MeOD) δ 7.62 – 7.39 (m, 20H), 7.39 – 7.28 (m, 7H), 7.14 – 7.02 (m, 3H), 6.66 (d, *J* = 14.0 Hz, 2H), 2.88 (s, 6H), 2.83 – 2.64 (m, 4H), 2.09 (s, 3H), 2.07 – 1.90 (m, 2H). ^{13}C NMR (101 MHz, $CDCl_3$ and MeOD) δ 135.68, 131.46, 130.67, 130.16, 129.65, 128.23, 126.59, 125.60, 124.41, 123.02, 25.50, 21.37, 19.48. HRMS (ESI^+) calcd 717.2644, found 717.2656 for $C_{51}H_{41}S_2^+$ ($M+H^+$).

General procedure C: preparation of *meso*-substituted heptamethine dyes. Unless otherwise noted, aryl bromide (0.34 mmol) was dissolved in dry THF (1 mL) and cooled to −84 °C. To this solution was added *t*-BuLi (1.7 M in pentane, 0.40 mL, 0.68 mmol). The mixture was stirred for 40 min at this temperature. A red to orange solution of keto-heptamethine (0.042 mmol) in dry THF (1 mL) was added dropwise. The mixture was allowed to warm up to room temperature and stirred for 30 min. The yellow or orange reaction mixture was quenched by adding 1:10 HCl which resulted in a rapid color change into dark green. The mixture was diluted in H_2O and extracted with CH_2Cl_2 (×4) and dried (Na_2SO_4). The crude product was purified by column chromatography (1:30 to 1:20 methanol / CH_2Cl_2).

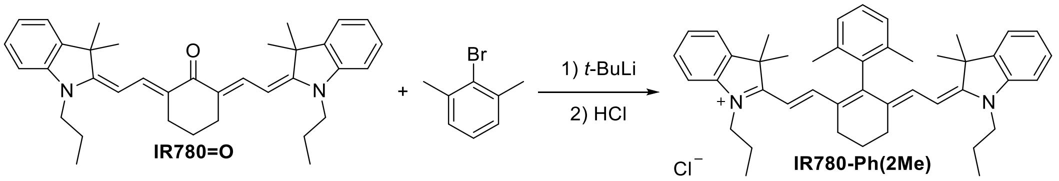

2-(2-(6-(2-(3,3-Dimethyl-1-propylindolin-2-ylidene)ethylidene)-2',6'-dimethyl-3,4,5,6-tetrahydro-[1,1'-biphenyl]-2-yl)vinyl)-3,3-dimethyl-1-propyl-3H-indol-1-ium chloride [**IR780-Ph(2Me)**]: Following General Procedure C, **IR780=O** (22 mg, 0.042 mmol) was reacted with 2-bromo-1,3-dimethylbenzene (62 mg, 0.34 mmol) and *t*-BuLi (1.7 M, 0.40 mL, 0.68 mmol) to give **IR780-Ph(2Me)** as a green solid film (20 mg, 75%). ^{1}H NMR (400 MHz, MeOD) δ 7.42 – 7.28 (m, 7H), 7.28 – 7.21 (m, 4H), 7.18 (t, *J* = 7.5 Hz, 2H), 6.21 (d, *J* = 14.1 Hz, 2H), 4.06 (t, *J* = 7.4 Hz, 4H), 2.75 (s, 4H), 2.12 (s, 6H), 2.10 – 2.05 (m, 2H), 1.83 (h, *J* = 7.3 Hz, 4H), 1.15 (s, 12H), 1.02 (t, *J* = 7.4 Hz, 6H). ^{13}C NMR (101 MHz, MeOD) δ 173.46, 162.69, 146.98, 143.78, 142.22, 138.95, 137.16, 129.70, 129.50, 129.13, 125.92, 123.34, 111.86, 101.06, 49.93, 46.37, 28.19, 25.40, 22.60, 21.72, 19.42, 14.43, 11.65. HRMS (ESI^+) calcd 609.4203, found 609.4196 for $C_{44}H_{53}N_2^+$ (M^+).

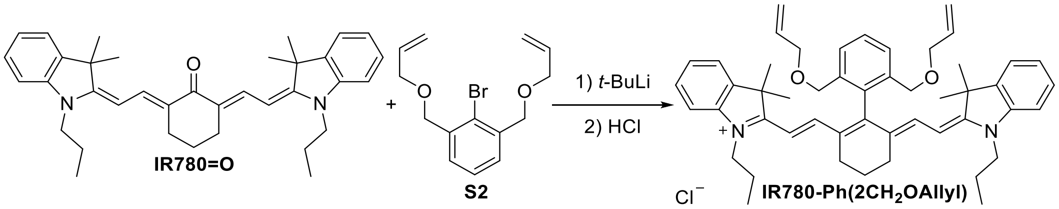

2-(2-(2',6'-Bis((allyloxy)methyl)-6-(2-(3,3-dimethyl-1-propylindolin-2-ylidene)ethylidene)-3,4,5,6-tetrahydro-[1,1'-biphenyl]-2-yl)vinyl)-3,3-dimethyl-1-propyl-3H-indol-1-ium chloride [**IR780-Ph(2CH2OAllyl)**]: Following General Procedure C, **IR780=O** (22 mg, 0.042 mmol) was reacted with **S2** (100 mg, 0.34 mmol) and *t*-BuLi (1.7 M, 0.40 mL, 0.68 mmol) to give **IR780-Ph(2CH2OAllyl)** as a green solid film (29 mg, 91%). ^{1}H

NMR (400 MHz, MeOD) δ 7.71 – 7.65 (m, 2H), 7.65 – 7.59 (m, 1H), 7.39 – 7.29 (m, 4H), 7.26 – 7.14 (m, 6H), 6.19 (d, J = 14.0 Hz, 2H), 5.83 – 5.69 (m, 2H), 5.16 (dd, J = 17.3, 1.8 Hz, 2H), 5.05 (dd, J = 10.4, 1.6 Hz, 2H), 4.28 (s, 4H), 4.06 (t, J = 7.3 Hz, 4H), 3.91 (dt, J = 5.6, 1.5 Hz, 4H), 2.75 (t, J = 6.2 Hz, 4H), 2.10 (p, J = 6.2 Hz, 2H), 1.82 (h, J = 7.4 Hz, 4H), 1.15 (s, 12H), 1.01 (t, J = 7.4 Hz, 6H). ^{13}C NMR (101 MHz, MeOD) δ 173.55, 159.07, 148.22, 143.72, 142.26, 138.10, 137.88, 136.05, 131.82, 129.86, 129.75, 129.66, 125.97, 123.30, 117.23, 111.88, 101.07, 72.97, 70.63, 49.98, 49.85, 46.34, 28.18, 25.56, 22.58, 21.74, 11.66. HRMS (ESI$^+$) calcd 721.4728, found 721.4721 for $C_{50}H_{61}N_2O_2^+$ (M$^+$).

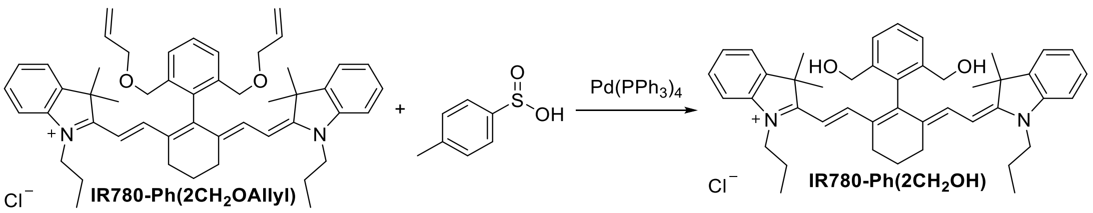

2-(2-6-(2-(3,3-Dimethyl-1-propylindolin-2-ylidene)ethylidene)-2',6'-bis(hydroxymethyl)-3,4,5,6-tetrahydro-[1,1'-biphenyl]-2-yl)vinyl)-3,3-dimethyl-1-propyl-3H-indol-1-ium chloride [**IR780-Ph(2CH₂OH)**]: **IR780-Ph(2CH₂OAllyl)** (22 mg, 0.029 mmol), *p*-toluenesulfinic acid (36 mg, 0.23 mmol) and $Pd(PPh_3)_4$ (6.7 mg, 0.0058 mmol) were dissolved in ethanol (2 mL) followed by three freeze-pump-thaw cycles. The mixture was stirred at 65°C under N_2 for 4.5 h. The reaction was diluted in CH_2Cl_2, washed with sat. $NaHCO_3$ and dried (Na_2SO_4). The crude product was purified by column chromatography (1:8 methanol / CH_2Cl_2) followed by semi-preparative HPLC (Phenomenex Gemini 5 µm C18, 250×10.0 mm, 40% – 100% MeCN in water with 0.1% TFA) to give **IR780-Ph(2CH₂OH)** as a green solid film (13 mg, 66%). ^{1}H NMR (400 MHz, MeOD) δ 7.74 – 7.69 (m, 2H), 7.67 – 7.61 (m, 1H), 7.39 – 7.29 (m, 4H), 7.27 – 7.13 (m, 6H), 6.19 (d, J = 14.1 Hz, 2H), 4.43 (s, 4H), 4.05 (t, J = 7.3 Hz, 4H), 2.74 (t, J = 6.2 Hz, 4H), 2.09 (t, J = 5.8 Hz, 2H), 1.82 (h, J = 7.4 Hz, 4H), 1.16 (s, 12H), 1.01 (t, J = 7.4 Hz, 6H). HRMS (ESI$^+$) calcd 641.4102, found 641.4109 for $C_{44}H_{53}N_2O_2^+$ (M$^+$).

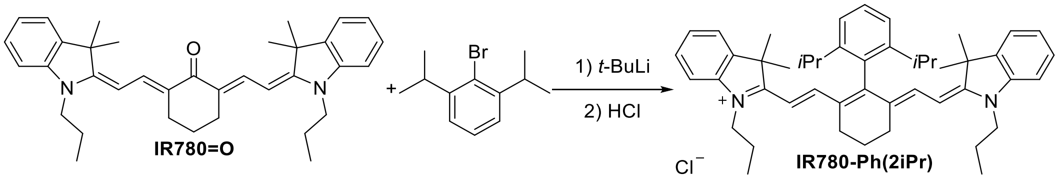

2-(2-(6-(2-(3,3-dimethyl-1-propylindolin-2-ylidene)ethylidene)-2',6'-diisopropyl-3,4,5,6-tetrahydro-[1,1'-biphenyl]-2-yl)vinyl)-3,3-dimethyl-1-propyl-3*H*-indol-1-ium chloride [**IR780-Ph(2iPr)**]: Following General Procedure C, **IR780=O** (22 mg, 0.042 mmol) was reacted with 2-bromo-1,3-diisopropylbenzene (82 mg, 0.34 mmol) and *t*-BuLi (1.7 M, 0.40 mL, 0.68 mmol). The mixture was purified by column chromatography followed by semi-preparative HPLC (Phenomenex Gemini 5 µm C18, 250×10.0 mm, 65% – 100% MeCN in water with 0.1% TFA) to give **IR780-Ph(2iPr)** as a green solid (9.2 mg, 31%). ^{1}H NMR (400 MHz, MeOD) δ 7.56 (t, J = 7.8 Hz, 1H), 7.43 (d, J = 7.8 Hz, 2H), 7.39 – 7.31 (m, 4H), 7.28 – 7.16 (m, 6H), 6.21 (d, J = 14.2 Hz, 2H), 4.06 (t, J = 7.3 Hz, 4H), 2.85 (p, J = 6.8 Hz, 2H), 2.78 (t, J = 6.3 Hz, 4H), 2.11 (p, J = 6.5 Hz, 2H), 1.83 (h, J = 7.4 Hz, 4H), 1.21 (s, 12H), 1.14 (d, J = 6.8 Hz, 12H), 1.01 (t, J = 7.4 Hz, 6H). ^{13}C NMR (101 MHz, MeOD) δ 173.23, 162.07, 148.07, 147.77, 143.75, 141.99, 136.34, 132.75, 130.35, 129.73, 126.03, 124.88, 123.29, 111.90, 101.12, 50.03, 46.42, 32.26, 28.63, 25.69, 25.45, 22.76, 21.72, 11.62. HRMS (ESI$^+$) calcd 665.4829, found 665.4825 for $C_{48}H_{61}N_2^+$ (M$^+$).

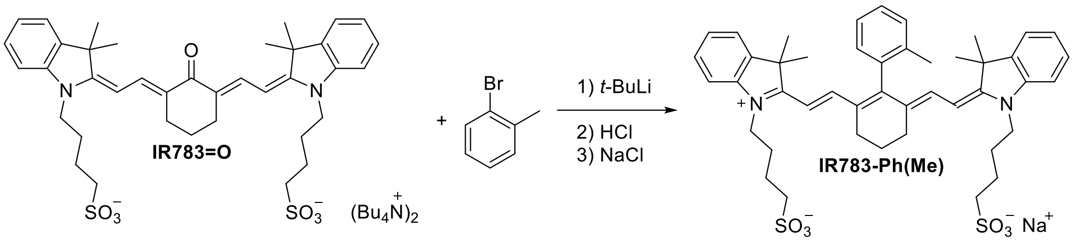

Sodium 4-(2-(2-(6-(2-(3,3-dimethyl-1-(4-sulfonatobutyl)-3*H*-indol-1-ium-2-yl)vinyl)-2'-methyl-4,5-dihydro-[1,1'-biphenyl]-2(3*H*)-ylidene)ethylidene)-3,3-dimethylindolin-1-yl)butane-1-sulfonate [**IR783-Ph(Me)**]: Following General Procedure C, **IR783=O** tetrabutylammonium salt (41 mg, 0.034 mmol) dissolved in dry THF (3 mL) was reacted with 2-bromotoluene (70.6 mg, 0.41 mmol) and *t*-BuLi (1.7 M, 0.49 mL, 0.83 mmol) in dry THF (4 mL). After stirring at room temperature for 30 min, the reaction was quenched by adding excess amount of acetic acid. The mixture was concentrated, diluted with H_2O (6 mL), extracted with hexanes (×2), and loaded onto preparative HPLC (Phenomenex Kinetex 5 µm phenyl-hexyl, 250×21.2 mm). The column was flushed three times with NaCl solution (7 mL, 0.2 M dissolved in 45% methanol in H_2O) and separated with a gradient of 45% – 90% methanol to give **IR783-Ph(Me)** sodium salt as a green solid (17 mg, 60%). ^{1}H NMR (500 MHz, MeOD) δ 7.51 – 7.46 (m, 2H), 7.46 – 7.40 (m, 1H), 7.37 – 7.29 (m, 4H), 7.26 (d, *J* = 8.2 Hz, 2H), 7.21 (d, *J* = 14.0 Hz, 2H), 7.16 (t, *J* = 7.4 Hz, 2H), 7.10 (d, *J* = 7.6 Hz, 1H), 6.23 (d, *J* = 14.0 Hz, 2H), 4.11 (t, *J* = 6.9 Hz, 4H), 2.88 (t, *J* = 6.2 Hz, 4H), 2.75 (t, *J* = 6.6 Hz, 4H), 2.14 (s, 3H), 2.07 (p, *J* = 6.3 Hz, 2H), 1.99 – 1.88 (m, 8H), 1.16 (s, 6H), 1.13 (s, 6H). ^{13}C NMR (126 MHz, MeOD) δ 173.31, 163.39, 148.60, 143.68, 142.22, 139.74, 137.40, 132.25, 131.66, 130.62, 129.73, 129.65, 127.42, 125.86, 123.23, 111.87, 101.06, 51.86, 44.81, 28.21, 28.03, 27.20, 25.52, 23.61, 22.63, 19.07. HRMS (ESI$^-$) calcd 781.3344, found 781.3351 for $C_{45}H_{53}N_2O_6S_2^-$ (M^-).

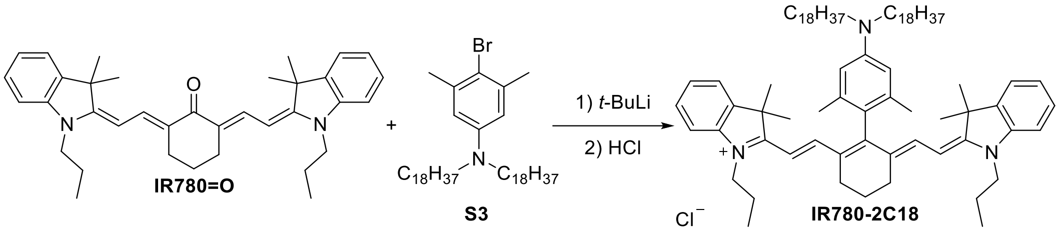

2-(2-(6-(2-(3,3-dimethyl-1-propylindolin-2-ylidene)ethylidene)-4'-(dioctadecylamino)-2',6'-dimethyl-3,4,5,6-tet rahydro-[1,1'-biphenyl]-2-yl)vinyl)-3,3-dimethyl-1-propyl-3H-indol-1-ium chloride (**IR780-2C18**): Following General Procedure C, *t*-BuLi (1.7 M, 0.40 mL, 0.68 mmol) was added to **S3** (238 mg, 0.34 mmol) in THF (3 mL) frozen at −84 °C and warmed up to room temperature until all solids were dissolved, at which point **IR780=O** (22 mg, 0.042 mmol) was added and stirred for 15 min. Subsequent work-up and column chromatography gave **IR780-2C18** as a green solid (25 mg, 51%). ^{1}H NMR (500 MHz, $CDCl_3$) δ 7.35 – 7.27 (m, 4H), 7.18 – 7.04 (m, 6H), 6.49 (s, 2H), 6.04 (d, *J* = 14.1 Hz, 2H), 4.00 (t, *J* = 7.3 Hz, 4H), 3.33 (t, *J* = 7.3 Hz, 4H), 2.66 (t, *J* = 5.9 Hz, 4H), 2.03 (p, *J* = 5.3 Hz, 2H), 1.98 (s, 6H), 1.84 (h, *J* = 7.3 Hz, 4H), 1.65 – 1.56 (m, 4H), 1.28 (d, *J* = 69.3 Hz, 60H), 1.16 (s, 12H), 1.02 (t, *J* = 7.4 Hz, 6H), 0.84 (t, *J* = 6.8 Hz, 6H). ^{13}C NMR (126 MHz, $CDCl_3$) δ 171.79, 164.06, 146.57, 142.80, 141.04, 136.54, 131.76, 128.78, 124.66, 121.98, 112.47, 110.73, 100.22, 51.46, 48.79, 46.18, 32.00, 29.90 – 29.72 (m), 29.40, 28.01, 27.55, 27.38, 24.87, 22.72, 21.60, 20.88, 19.97, 14.05, 11.65. HRMS (ESI$^+$) calcd 1128.9946, found 1128.9935 for $C_{80}H_{126}N_3^+$ (M^+).

NMR and mass Spectra

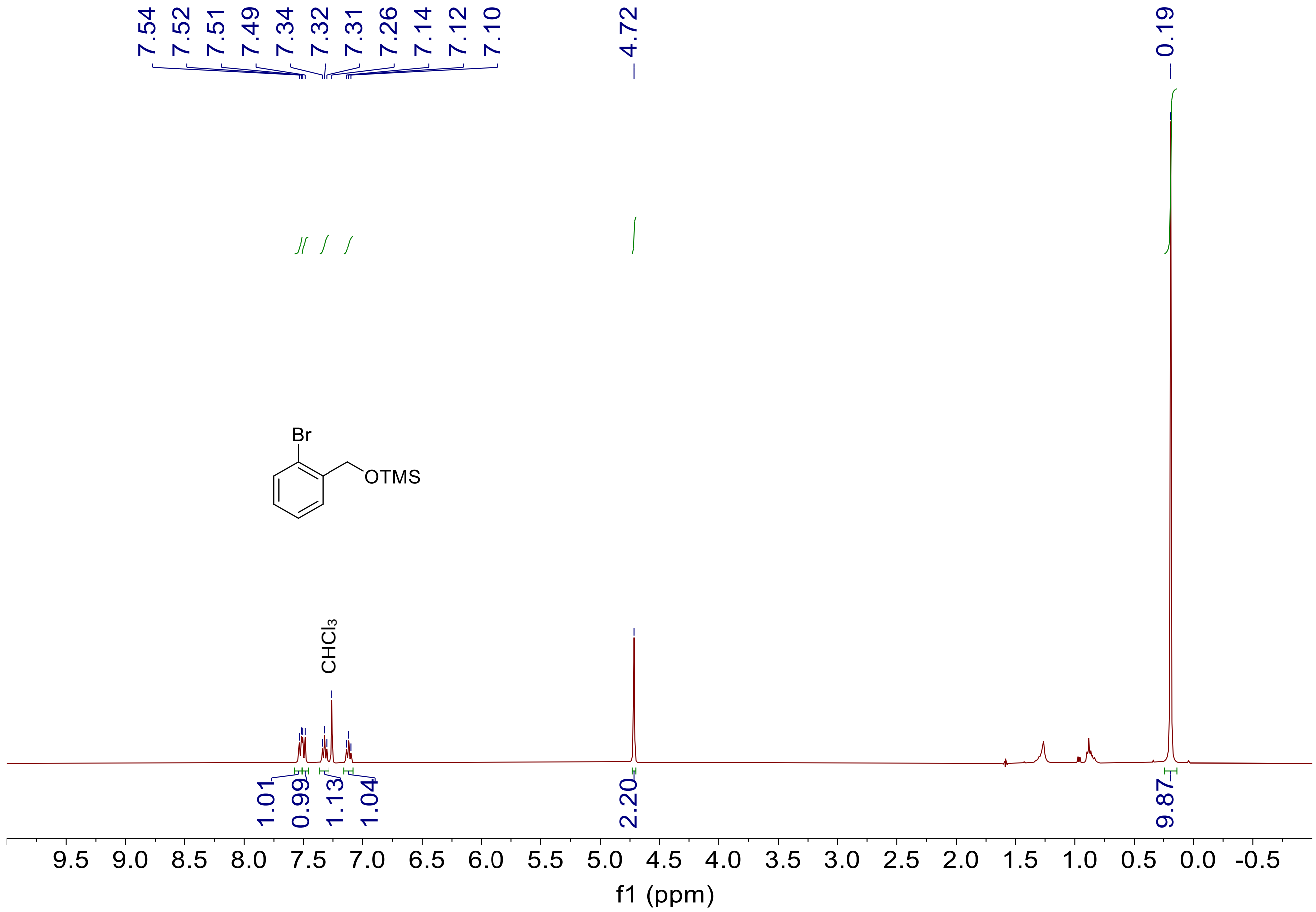

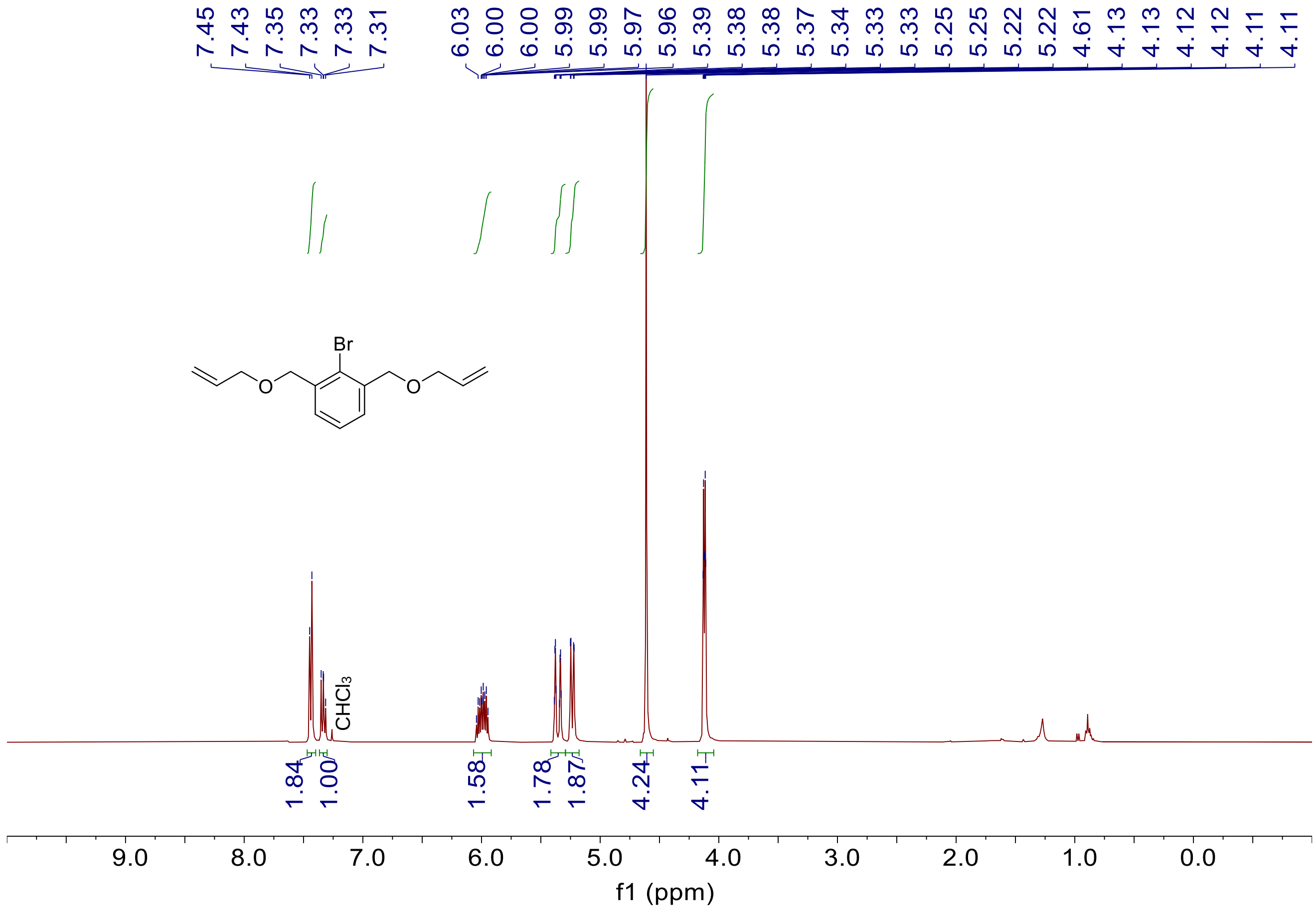
7.45
7.43
7.35
7.33
7.33
7.31
6.03
6.00
6.00
5.99
5.99
5.97
5.96
5.39
5.38
5.38
5.37
5.34
5.33
5.33
5.25
5.25
5.22
5.22
4.61
4.13
4.13
4.12
4.12
4.11
4.11
Br
O
O
$CHCl_3$
1.84
1.00
1.58
1.78
1.87
4.24
4.11
9.0
8.0
7.0
6.0
5.0
4.0
3.0
2.0
1.0
0.0
f1 (ppm)

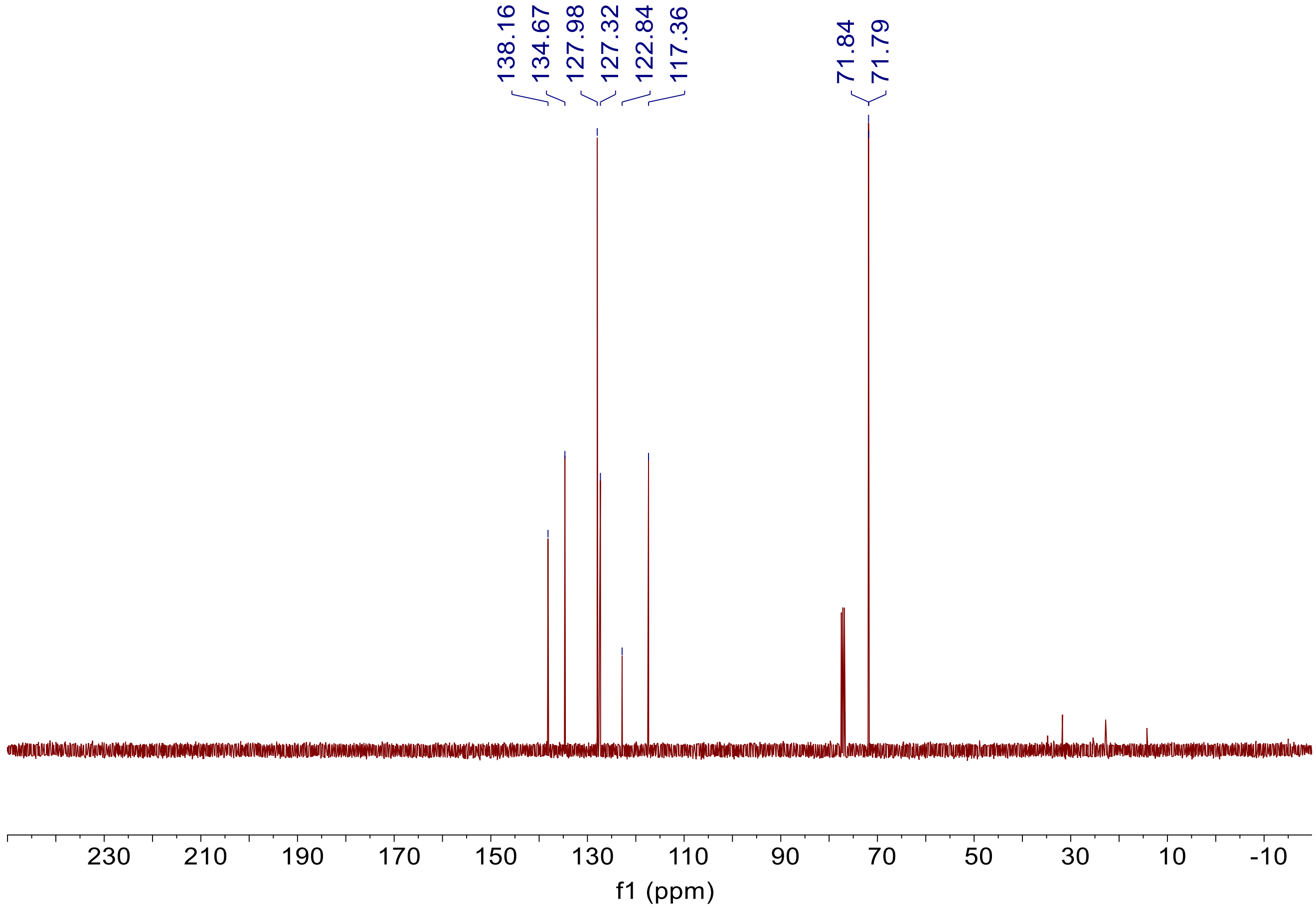
138.16
134.67
127.98
127.32
122.84
117.36
71.84
71.79
230
210
190
170
150
130
110
90
70
50
30
10
-10
f1 (ppm)

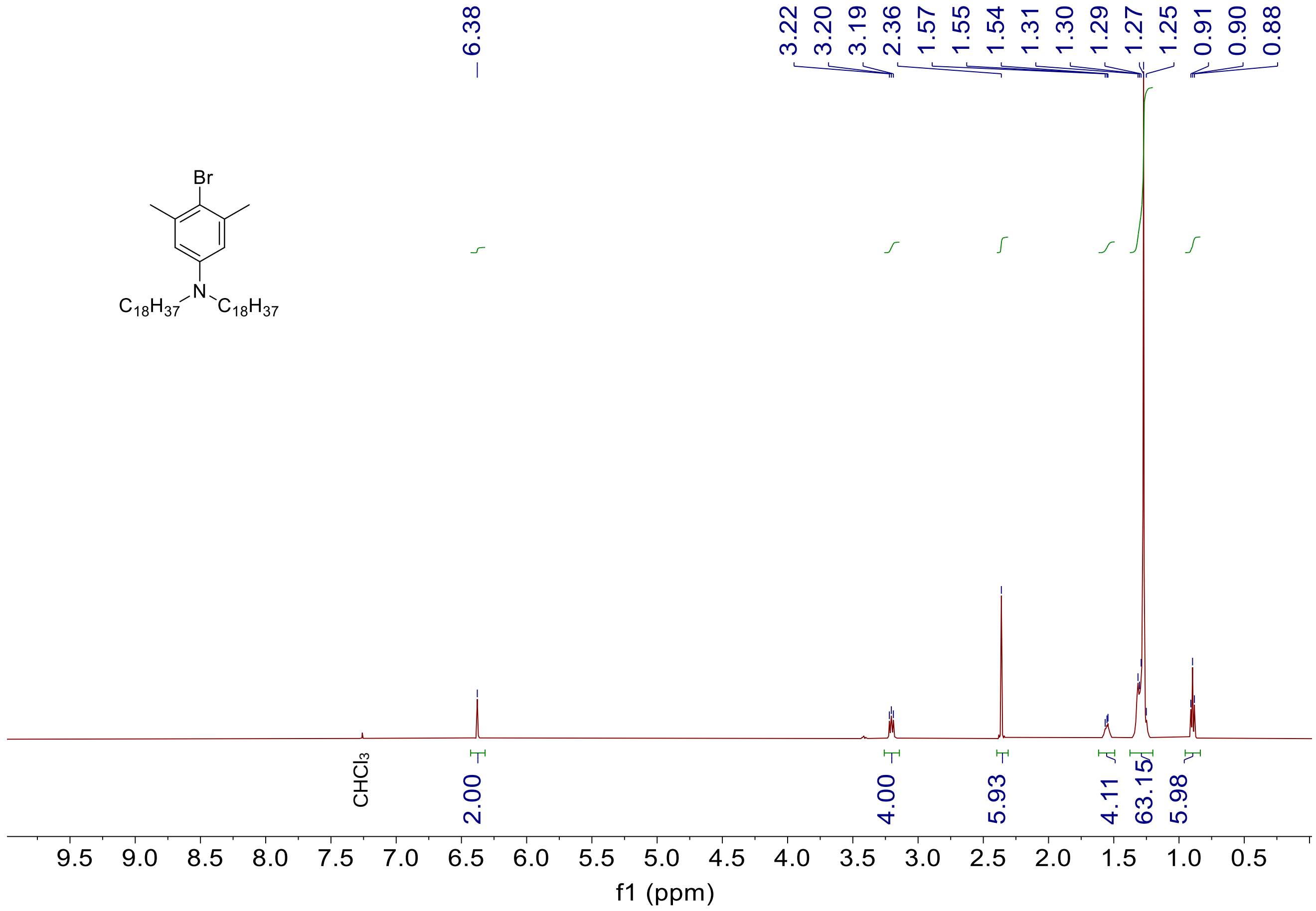

6.38
3.22
3.20
3.19
2.36
1.57
1.55
1.54
1.31
1.30
1.29
1.27
1.25
0.91
0.90
0.88
Br
N
C18H37
C18H37
CHCl3
2.00
4.00
5.93
4.11
63.15
5.98
9.5 9.0 8.5 8.0 7.5 7.0 6.5 6.0 5.5 5.0 4.5 4.0 3.5 3.0 2.5 2.0 1.5 1.0 0.5
f1 (ppm)

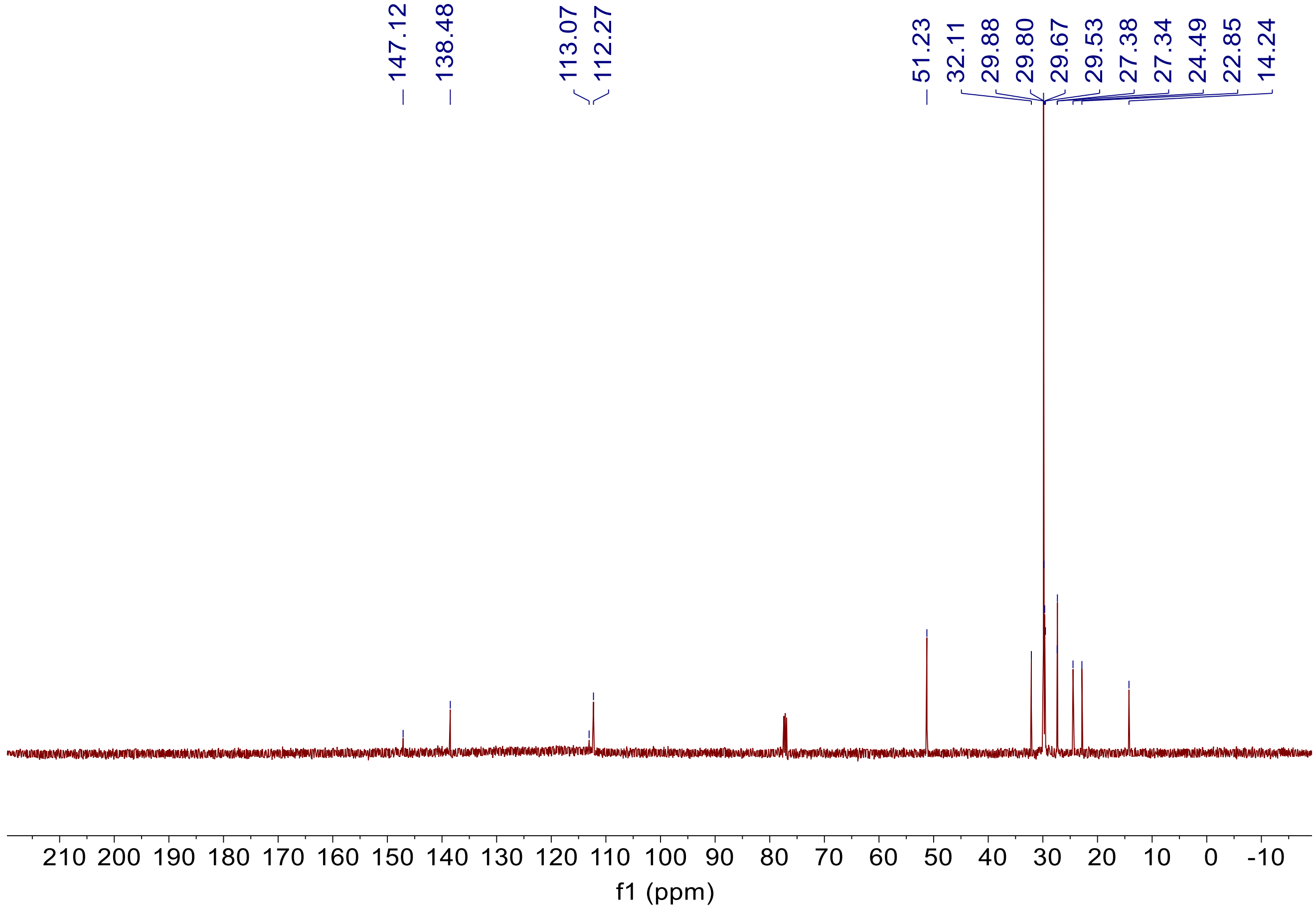

147.12
138.48
113.07
112.27
51.23
32.11
29.88
29.80
29.67
29.53
27.38
27.34
24.49
22.85
14.24
210 200 190 180 170 160 150 140 130 120 110 100 90 80 70 60 50 40 30 20 10 0 -10
f1 (ppm)

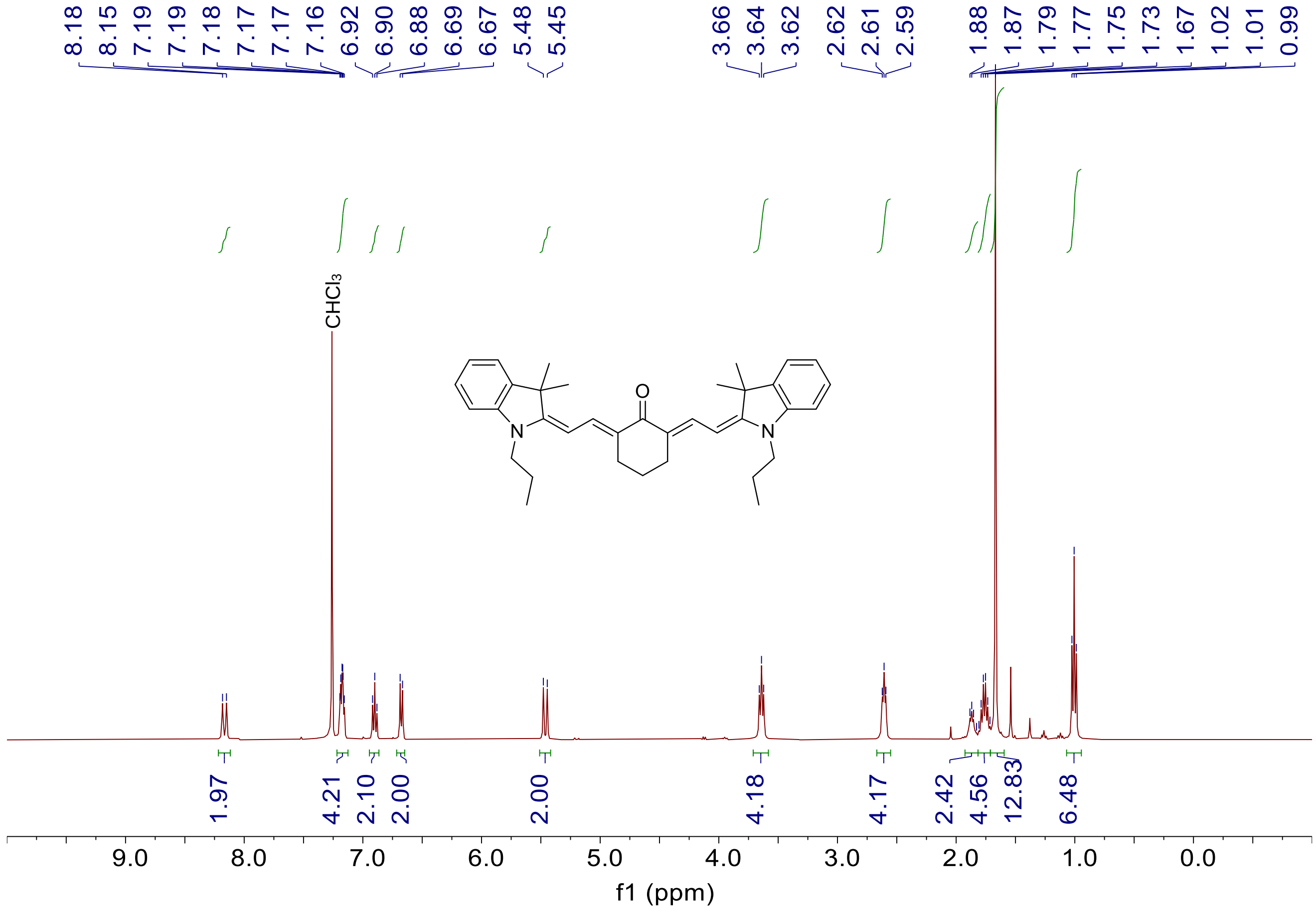
8.18 8.15 7.19 7.19 7.18 7.17 7.17 7.16 6.92 6.90 6.88 6.69 6.67 5.48 5.45
3.66 3.64 3.62 2.62 2.61 2.59
1.88 1.87 1.79 1.77 1.75 1.73 1.67 1.02 1.01 0.99
CHCl3
O
N
N
1.97 4.21 2.10 2.00 2.00 4.18 4.17 2.42 4.56 12.83 6.48
9.0 8.0 7.0 6.0 5.0 4.0 3.0 2.0 1.0 0.0
f1 (ppm)

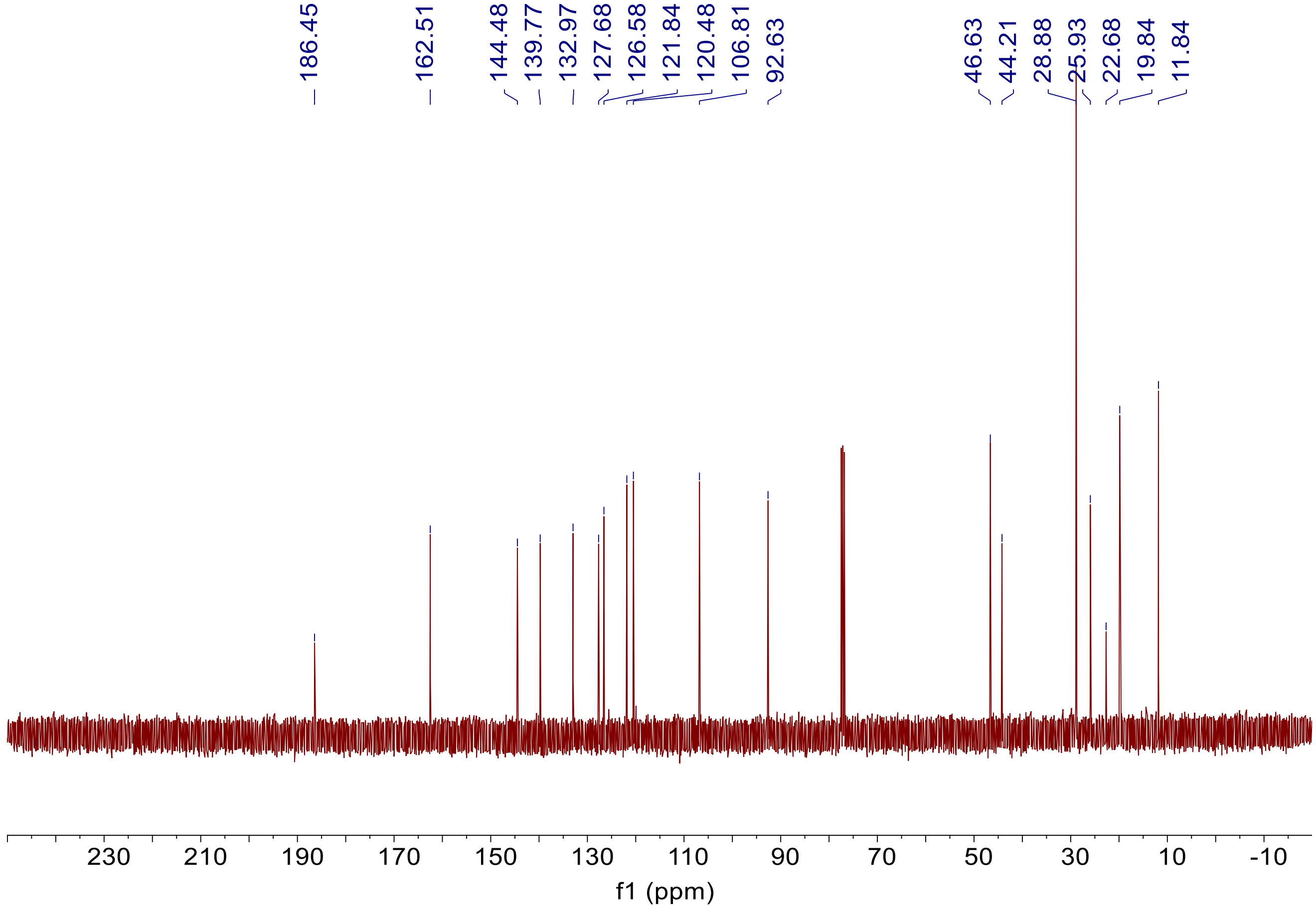
186.45
162.51
144.48 139.77 132.97 127.68 126.58 121.84 120.48 106.81 92.63
46.63 44.21 28.88 25.93 22.68 19.84 11.84
230 210 190 170 150 130 110 90 70 50 30 10 -10
f1 (ppm)

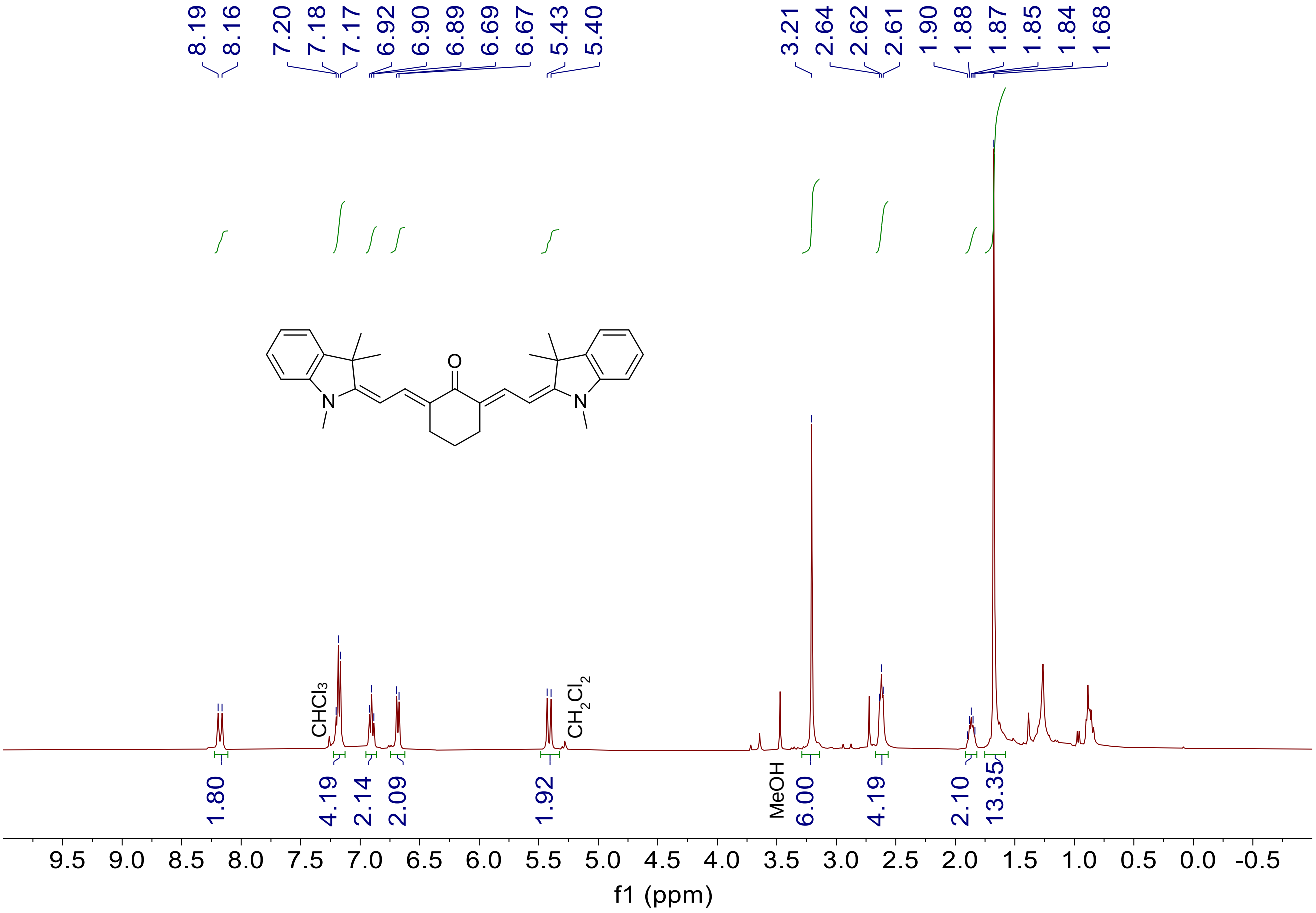
8.19
8.16
7.20
7.18
7.17
6.92
6.90
6.89
6.69
6.67
5.43
5.40
3.21
2.64
2.62
2.61
1.90
1.88
1.87
1.85
1.84
1.68
O
N
N
CHCl3
CH2Cl2
1.80
4.19
2.14
2.09
1.92
MeOH
6.00
4.19
2.10
13.35
9.5 9.0 8.5 8.0 7.5 7.0 6.5 6.0 5.5 5.0 4.5 4.0 3.5 3.0 2.5 2.0 1.5 1.0 0.5 0.0 -0.5
f1 (ppm)

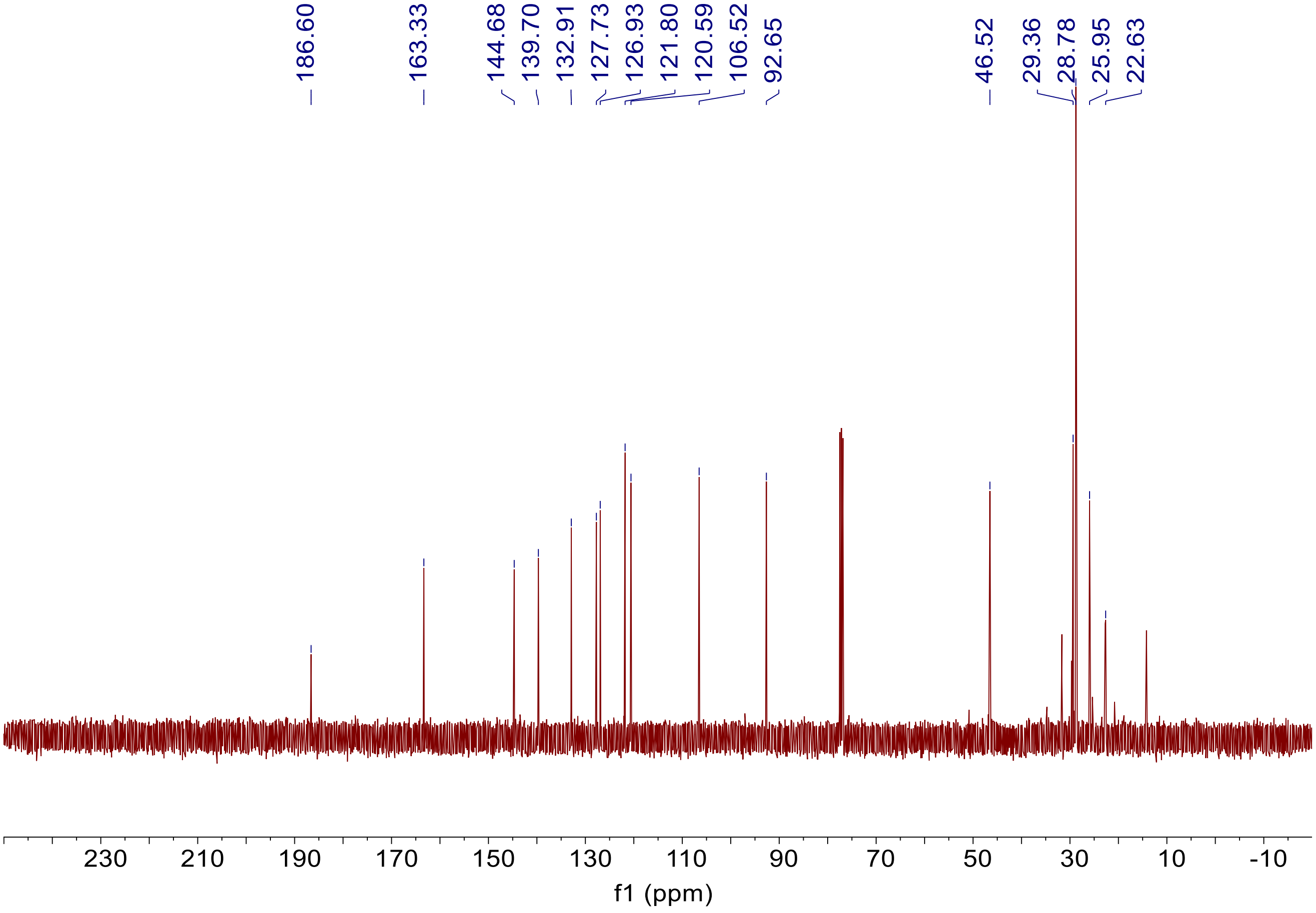
186.60
163.33
144.68
139.70
132.91
127.73
126.93
121.80
120.59
106.52
92.65
46.52
29.36
28.78
25.95
22.63
230 210 190 170 150 130 110 90 70 50 30 10 -10
f1 (ppm)

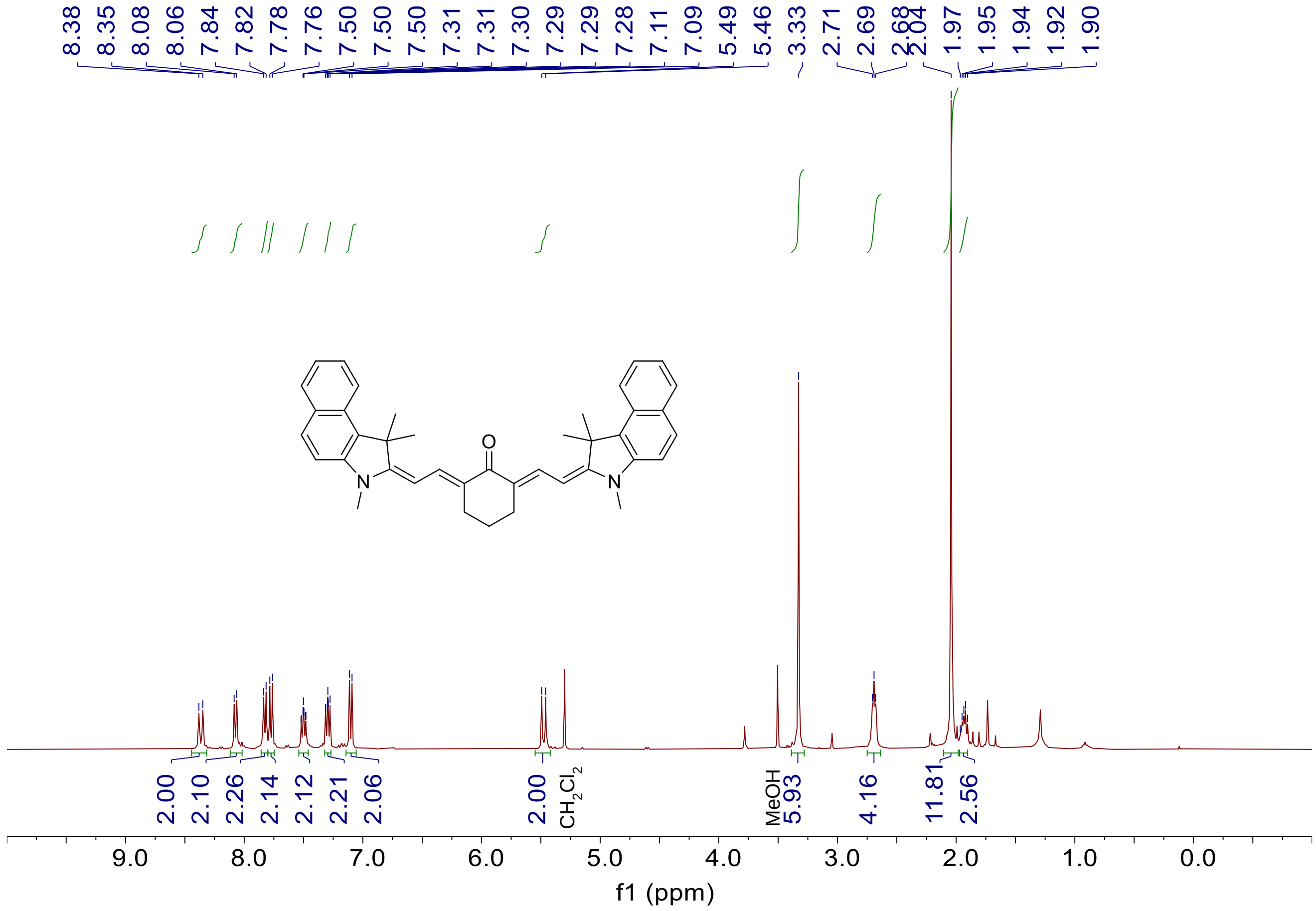
8.38
8.35
8.08
8.06
7.84
7.82
7.78
7.76
7.50
7.50
7.50
7.31
7.31
7.30
7.29
7.29
7.28
7.11
7.09
5.49
5.46
3.33
2.71
2.69
2.68
2.04
1.97
1.95
1.94
1.92
1.90
2.00
2.10
2.26
2.14
2.12
2.21
2.06
2.00
CH_2Cl_2
MeOH
5.93
4.16
11.81
2.56
9.0
8.0
7.0
6.0
5.0
4.0
3.0
2.0
1.0
0.0
f1 (ppm)
O
N
N

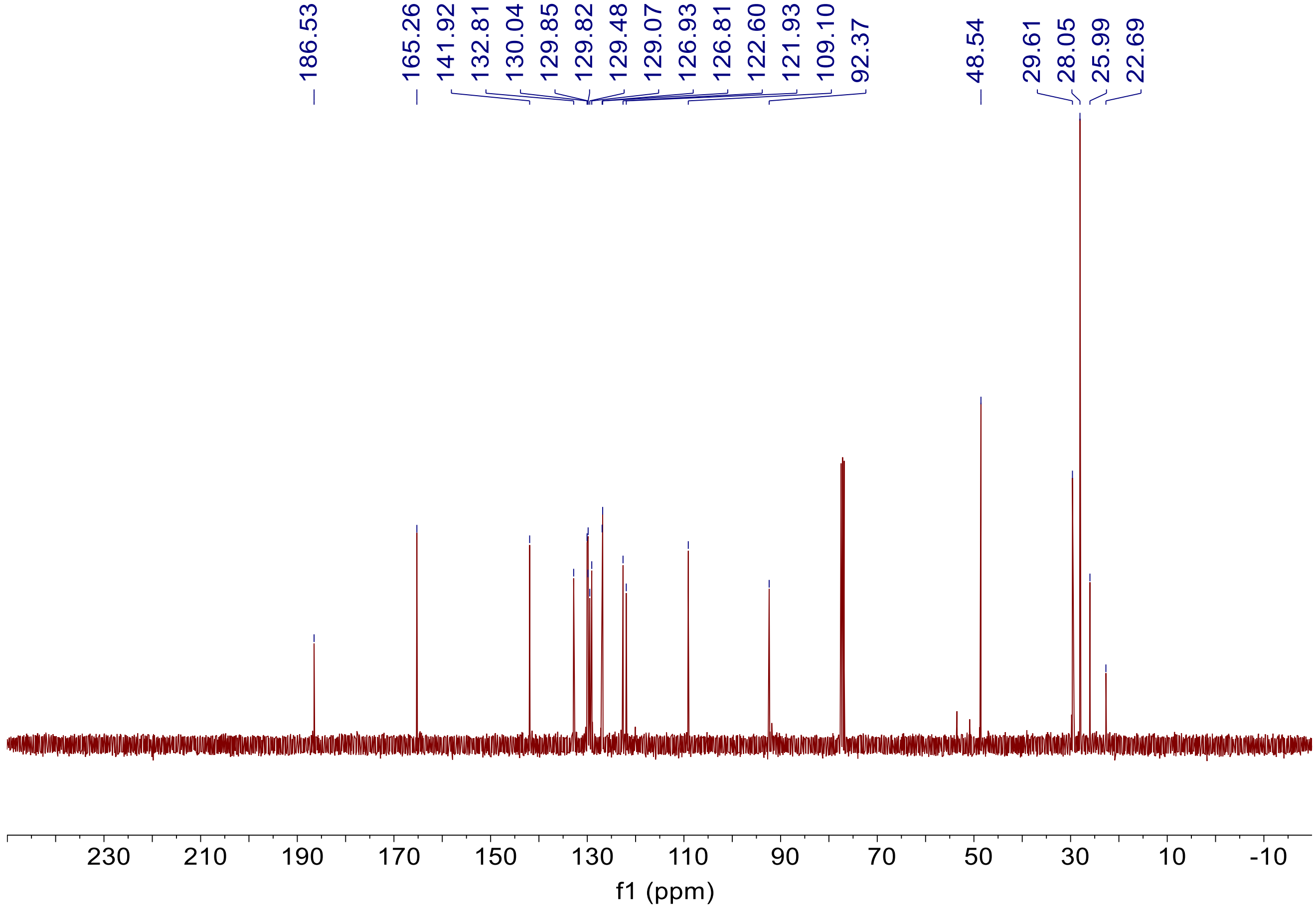
186.53
165.26
141.92
132.81
130.04
129.85
129.82
129.48
129.07
126.93
126.81
122.60
121.93
109.10
92.37
48.54
29.61
28.05
25.99
22.69
230
210
190
170
150
130
110
90
70
50
30
10
-10
f1 (ppm)

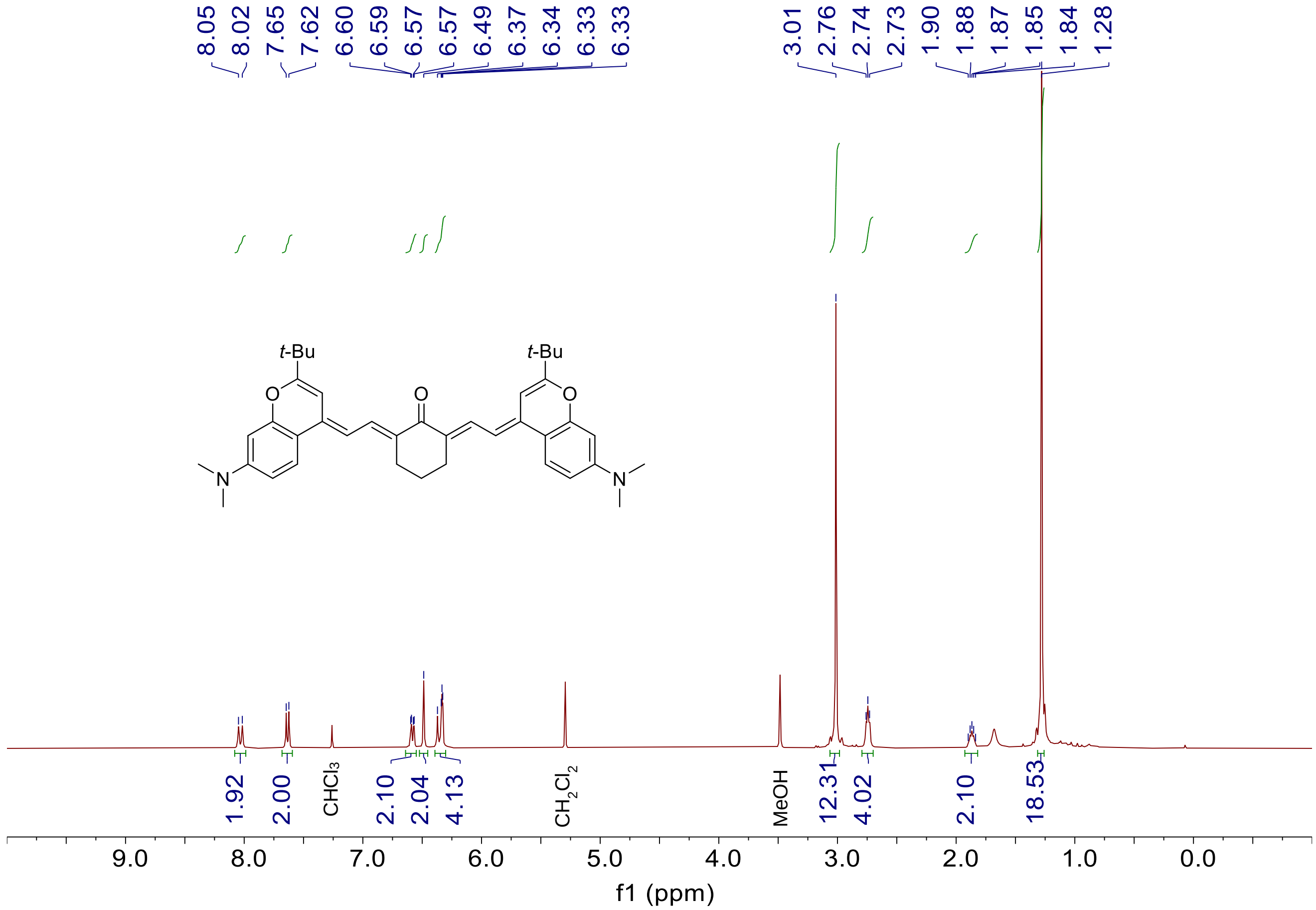
8.05 8.02 7.65 7.62 6.60 6.59 6.57 6.57 6.49 6.37 6.34 6.33 6.33
3.01 2.76 2.74 2.73 1.90 1.88 1.87 1.85 1.84 1.28
t-Bu
O
t-Bu
O
O
N
N
1.92
2.00
CHCl3
2.10
2.04
4.13
CH2Cl2
MeOH
12.31
4.02
2.10
18.53
9.0 8.0 7.0 6.0 5.0 4.0 3.0 2.0 1.0 0.0
f1 (ppm)

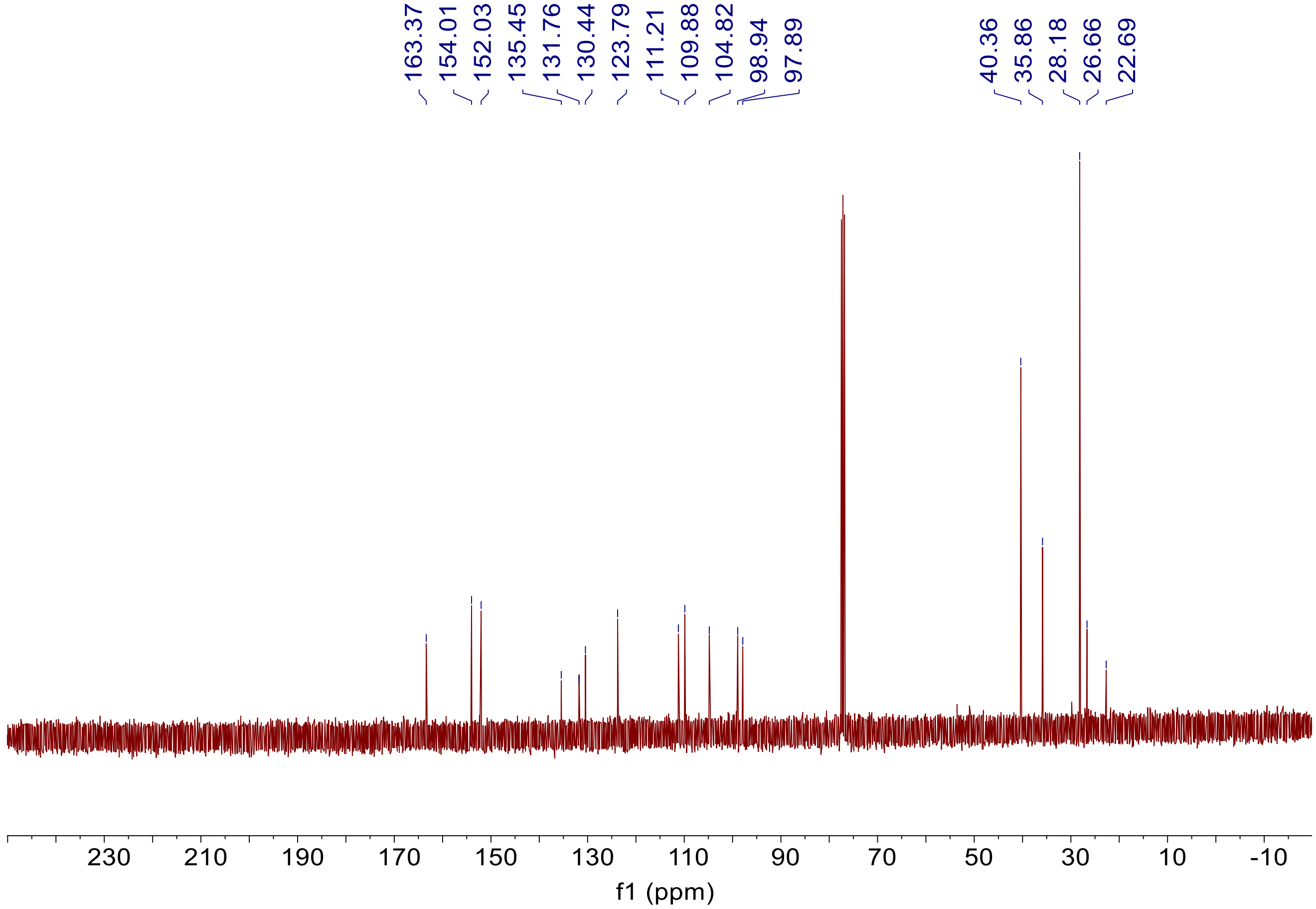
163.37 154.01 152.03 135.45 131.76 130.44 123.79 111.21 109.88 104.82 98.94 97.89
40.36 35.86 28.18 26.66 22.69
230 210 190 170 150 130 110 90 70 50 30 10 -10
f1 (ppm)

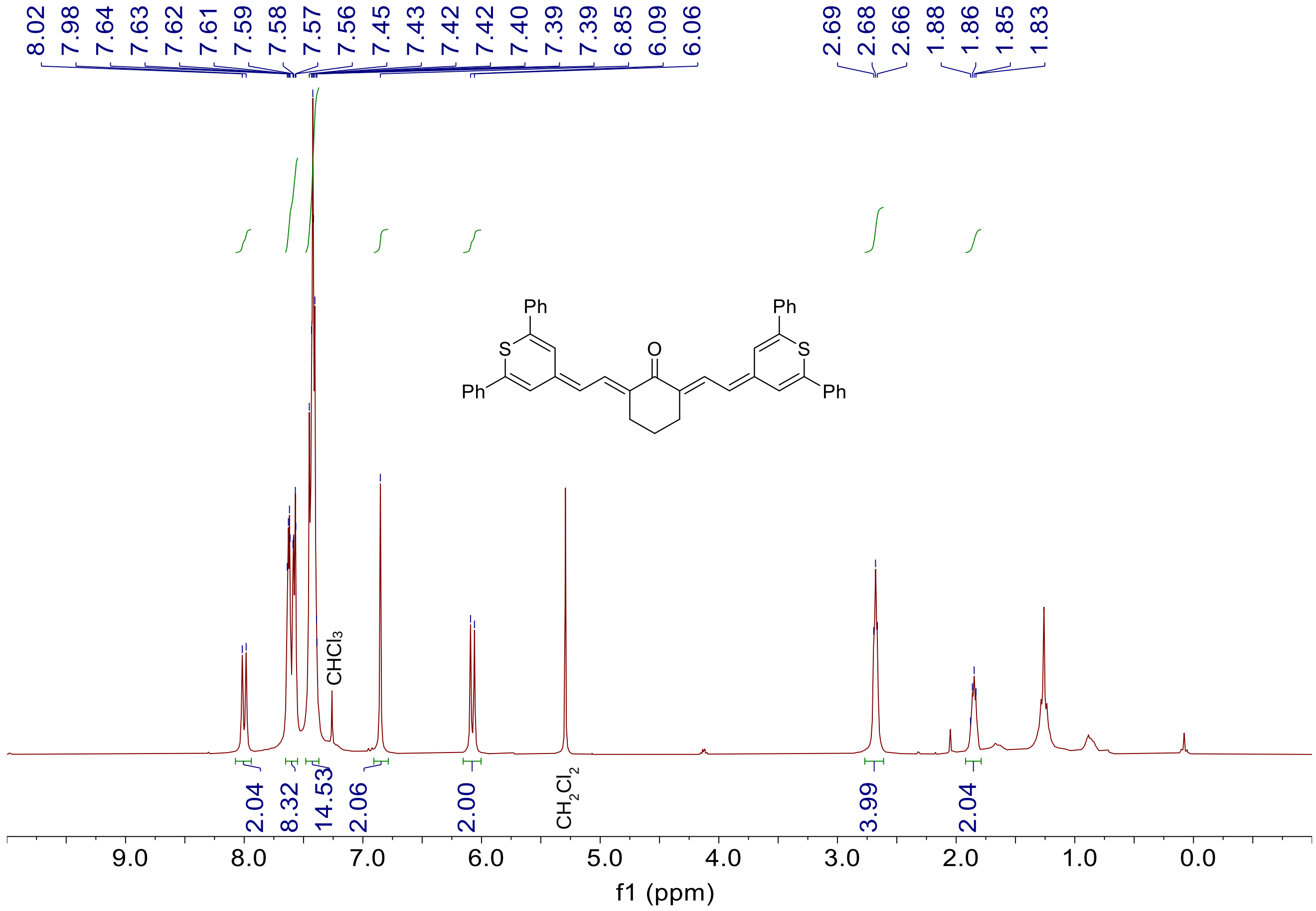

8.02 7.98 7.64 7.63 7.62 7.61 7.59 7.58 7.57 7.56 7.45 7.43 7.42 7.42 7.40 7.39 7.39 6.85 6.09 6.06
2.69 2.68 2.66 1.88 1.86 1.85 1.83
Ph
S
Ph
O
Ph
S
Ph
CHCl3
2.04 8.32 14.53 2.06 2.00 CH2Cl2 3.99 2.04
9.0 8.0 7.0 6.0 5.0 4.0 3.0 2.0 1.0 0.0
f1 (ppm)

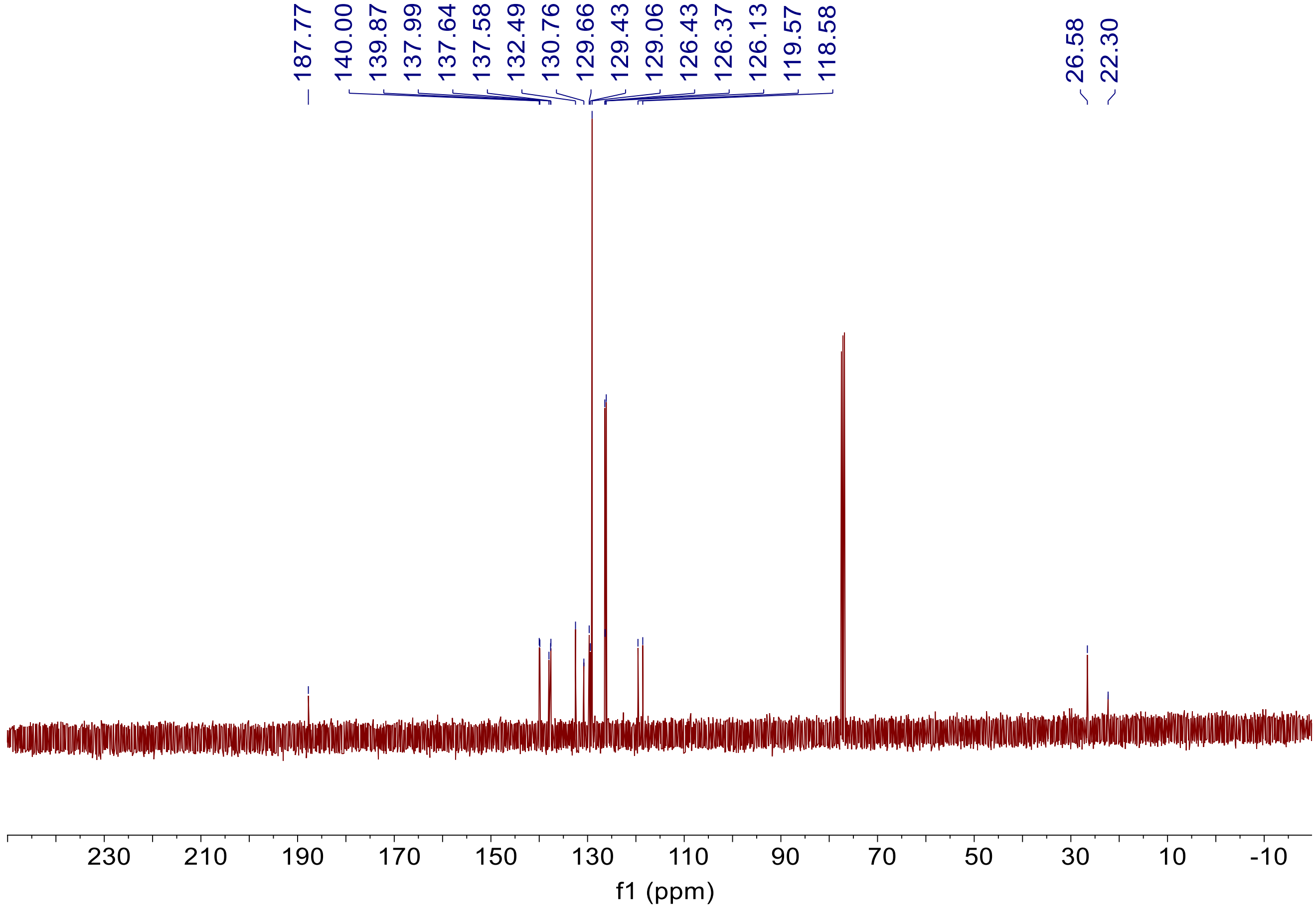

187.77
140.00 139.87 137.99 137.64 137.58 132.49 130.76 129.66 129.43 129.06 126.43 126.37 126.13 119.57 118.58
26.58 22.30
230 210 190 170 150 130 110 90 70 50 30 10 -10
f1 (ppm)

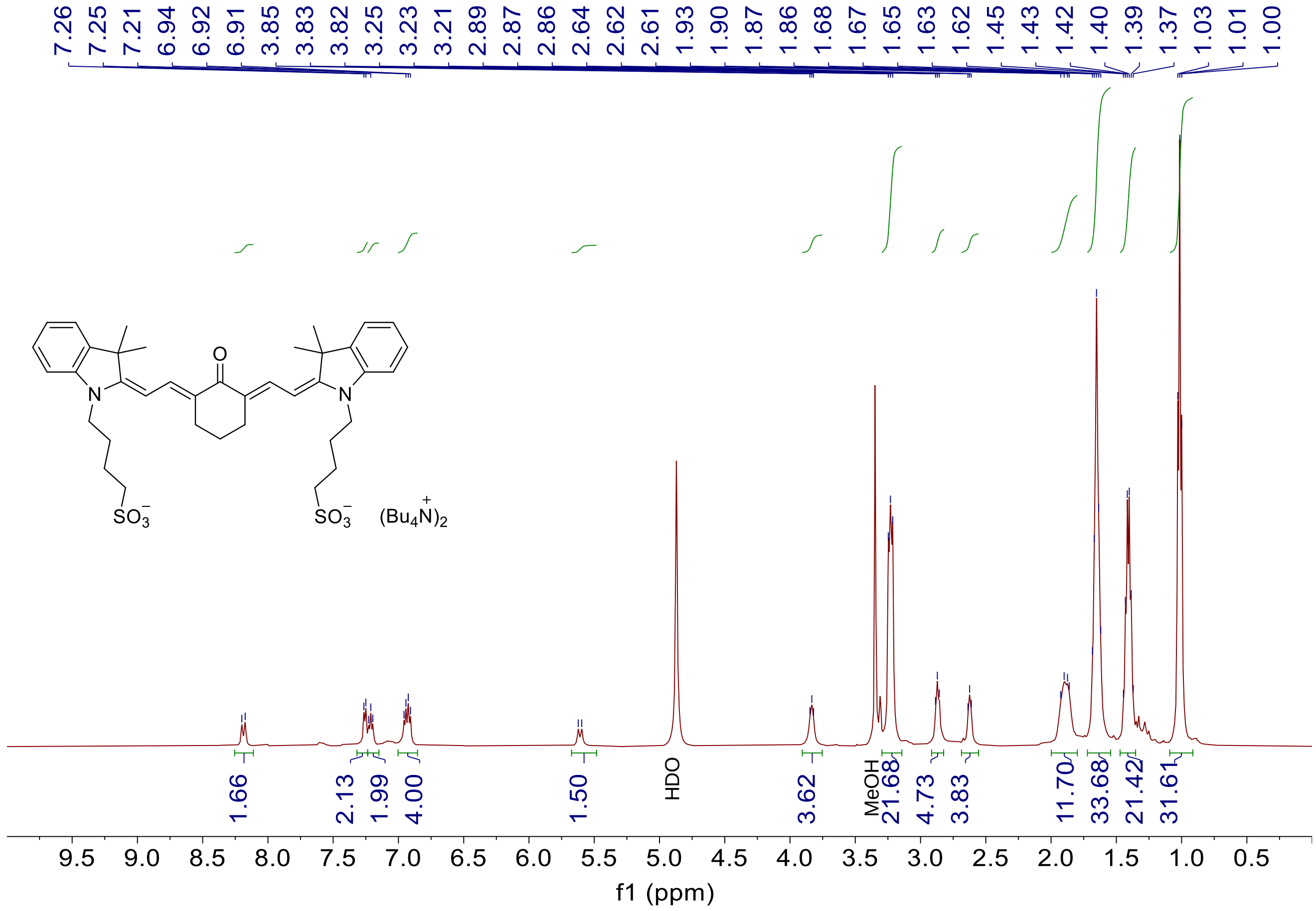
7.26 7.25 7.21 6.94 6.92 6.91 3.85 3.83 3.82 3.25 3.23 3.21 2.89 2.87 2.86 2.64 2.62 2.61 1.93 1.90 1.87 1.86 1.68 1.67 1.65 1.63 1.62 1.45 1.43 1.42 1.40 1.39 1.37 1.03 1.01 1.00
O
N
N
SO_3^-
SO_3^-
$(Bu_4\overset{+}{N})_2$
1.66
2.13
1.99
4.00
1.50
HDO
3.62
MeOH
21.68
4.73
3.83
11.70
33.68
21.42
31.61
9.5 9.0 8.5 8.0 7.5 7.0 6.5 6.0 5.5 5.0 4.5 4.0 3.5 3.0 2.5 2.0 1.5 1.0 0.5
f1 (ppm)

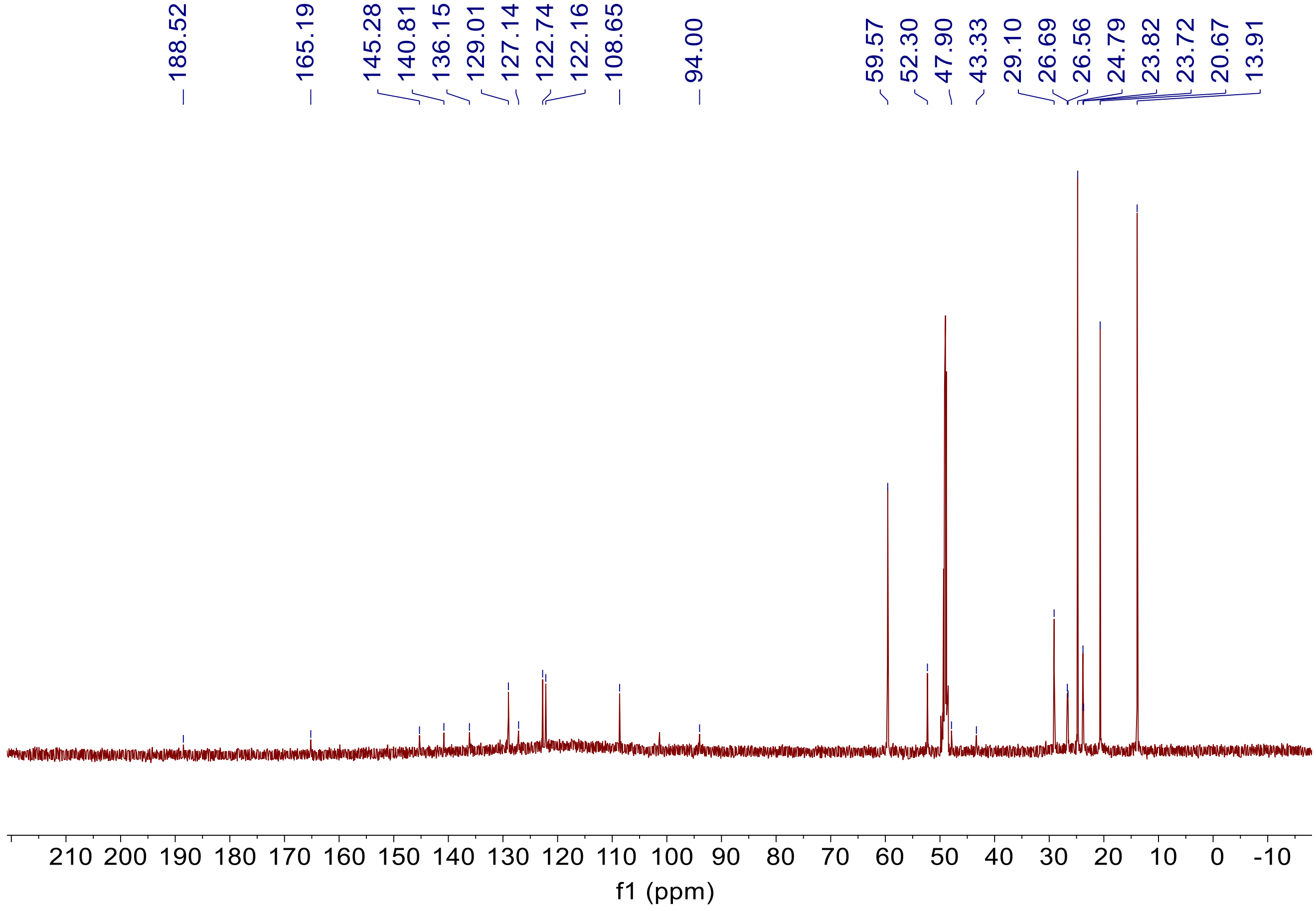
188.52
165.19
145.28 140.81 136.15 129.01 127.14 122.74 122.16
108.65
94.00
59.57 52.30 47.90 43.33 29.10 26.69 26.56 24.79 23.82 23.72 20.67 13.91
210 200 190 180 170 160 150 140 130 120 110 100 90 80 70 60 50 40 30 20 10 0 -10
f1 (ppm)

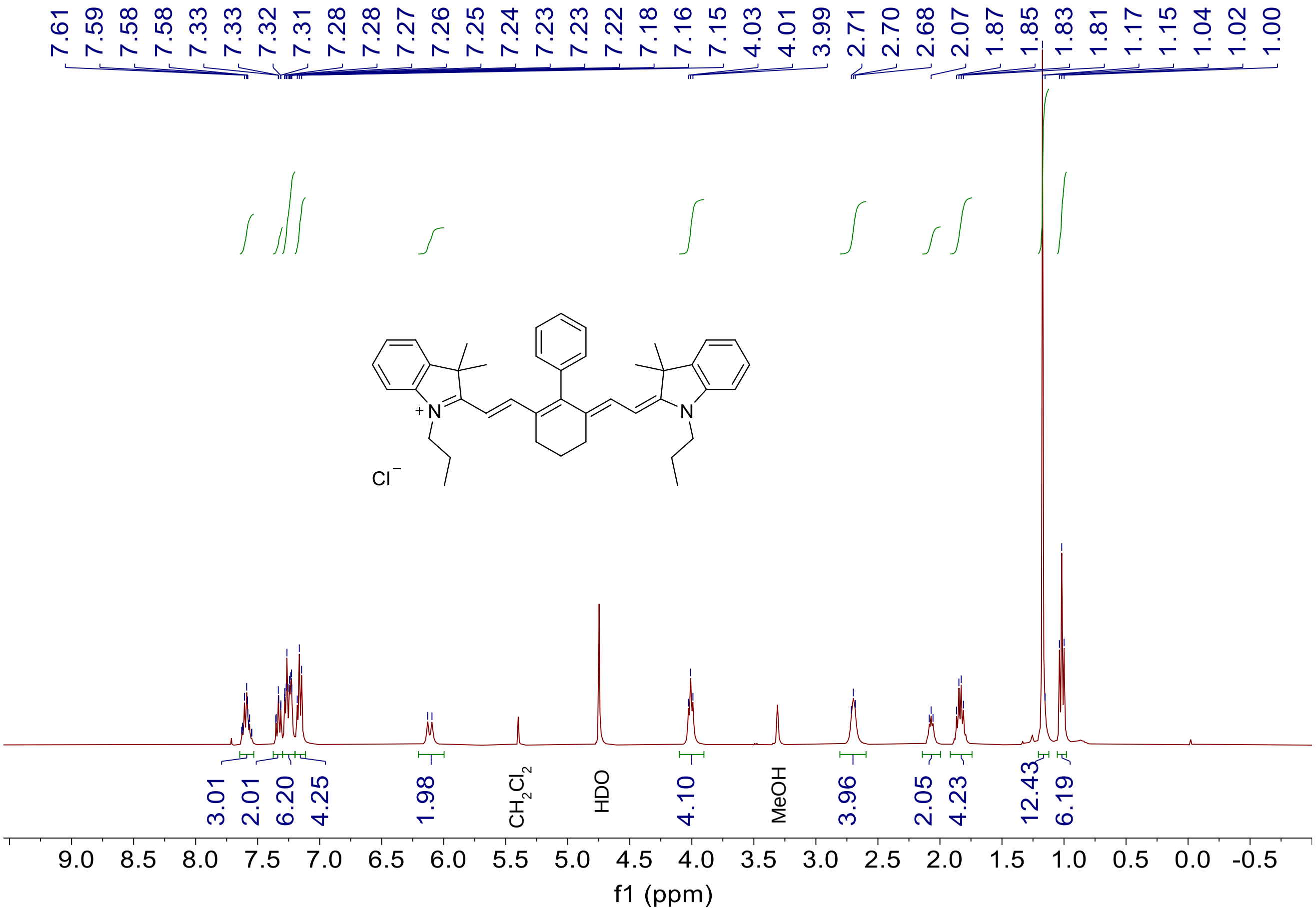
7.61 7.59 7.58 7.58 7.33 7.33 7.32 7.31 7.28 7.28 7.27 7.26 7.25 7.24 7.23 7.23 7.22 7.18 7.16 7.15 4.03 4.01 3.99 2.71 2.70 2.68 2.07 1.87 1.85 1.83 1.81 1.17 1.15 1.04 1.02 1.00
Cl⁻
N
+N
3.01 2.01 6.20 4.25 1.98 4.10 3.96 2.05 4.23 12.43 6.19
CH2Cl2
HDO
MeOH
9.0 8.5 8.0 7.5 7.0 6.5 6.0 5.5 5.0 4.5 4.0 3.5 3.0 2.5 2.0 1.5 1.0 0.5 0.0 -0.5
f1 (ppm)

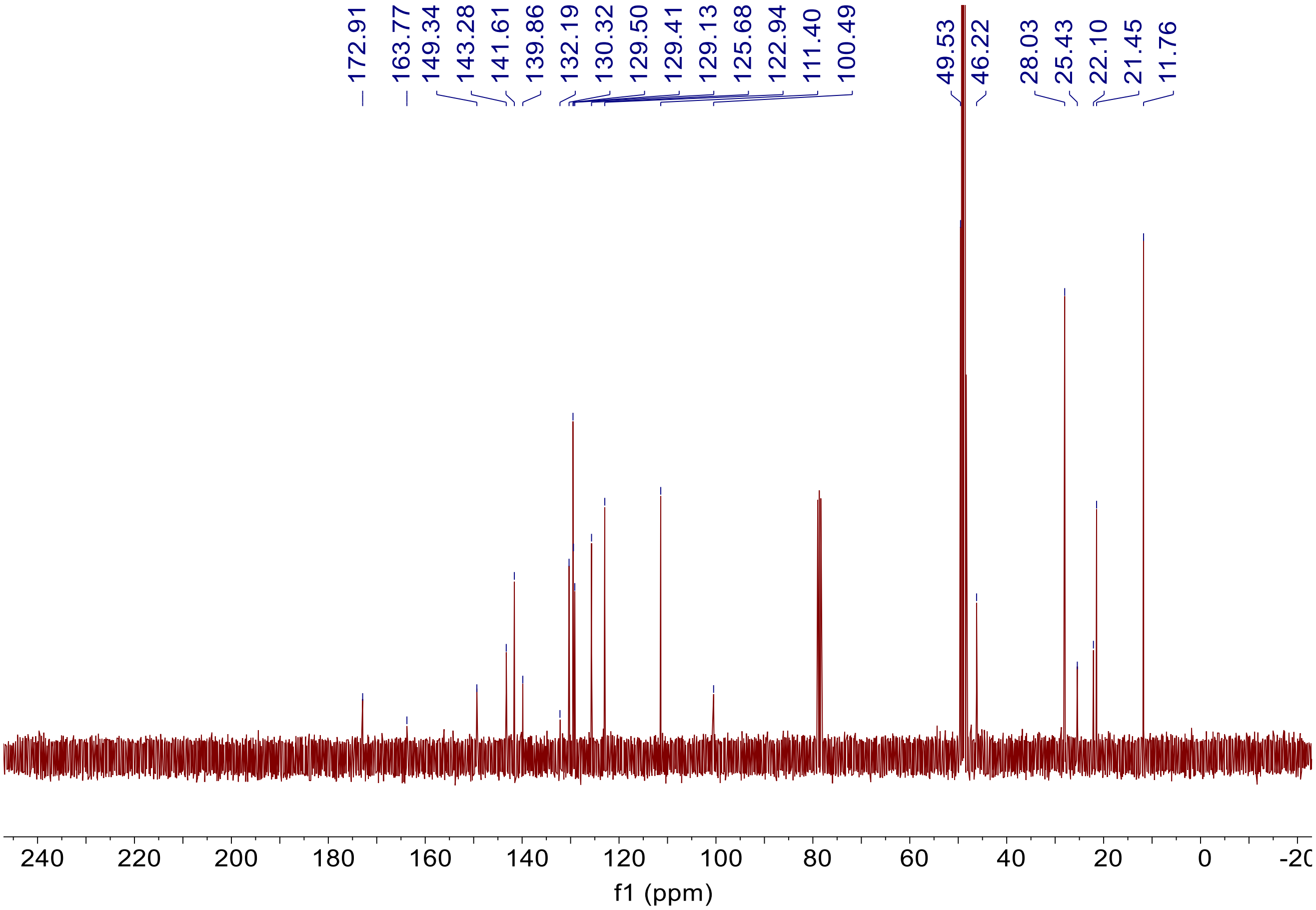
172.91 163.77 149.34 143.28 141.61 139.86 132.19 130.32 129.50 129.41 129.13 125.68 122.94 111.40 100.49 49.53 46.22 28.03 25.43 22.10 21.45 11.76
240 220 200 180 160 140 120 100 80 60 40 20 0 -20
f1 (ppm)

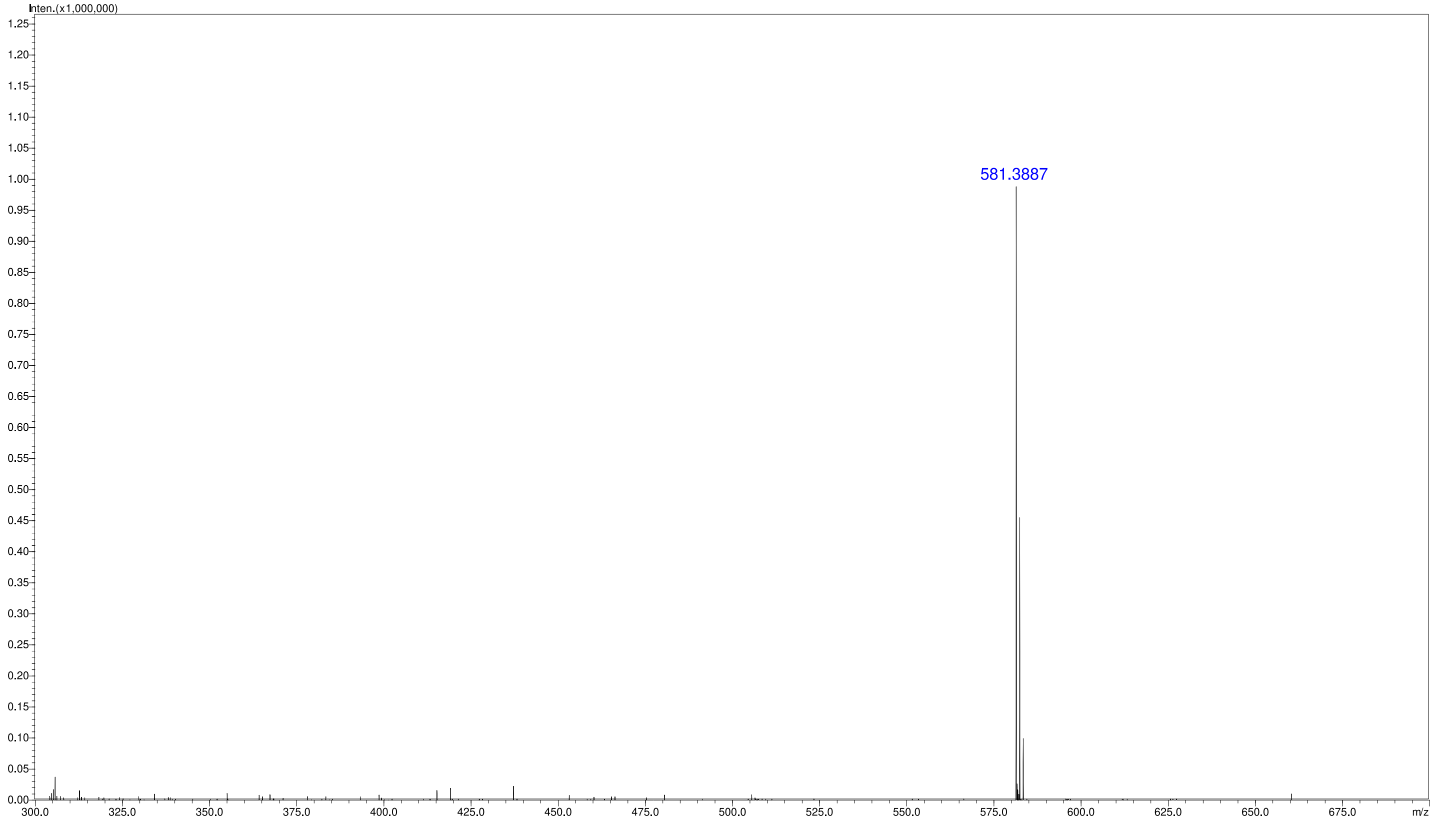
Inten.(x1,000,000)
581.3887
m/z
1.25
1.20
1.15
1.10
1.05
1.00
0.95
0.90
0.85
0.80
0.75
0.70
0.65
0.60
0.55
0.50
0.45
0.40
0.35
0.30
0.25
0.20
0.15
0.10
0.05
0.00
300.0
325.0
350.0
375.0
400.0
425.0
450.0
475.0
500.0
525.0
550.0
575.0
600.0
625.0
650.0
675.0

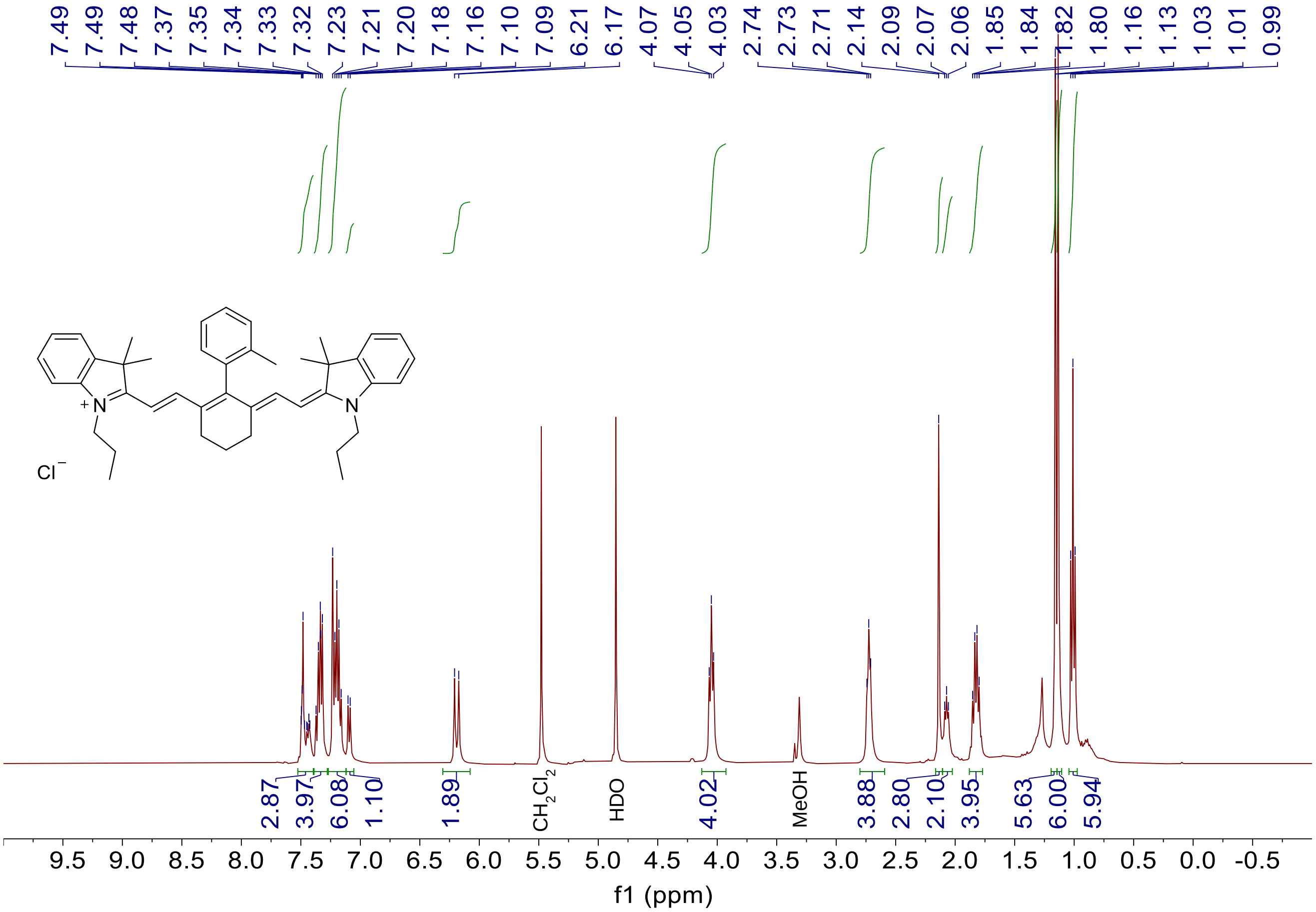
7.49
7.49
7.48
7.37
7.35
7.34
7.33
7.32
7.23
7.21
7.20
7.18
7.16
7.10
7.09
6.21
6.17
4.07
4.05
4.03
2.74
2.73
2.71
2.14
2.09
2.07
2.06
1.85
1.84
1.82
1.80
1.16
1.13
1.03
1.01
0.99
Cl⁻
2.87
3.97
6.08
1.10
1.89
CH2Cl2
HDO
4.02
MeOH
3.88
2.80
2.10
3.95
5.63
6.00
5.94
9.5 9.0 8.5 8.0 7.5 7.0 6.5 6.0 5.5 5.0 4.5 4.0 3.5 3.0 2.5 2.0 1.5 1.0 0.5 0.0 -0.5
f1 (ppm)

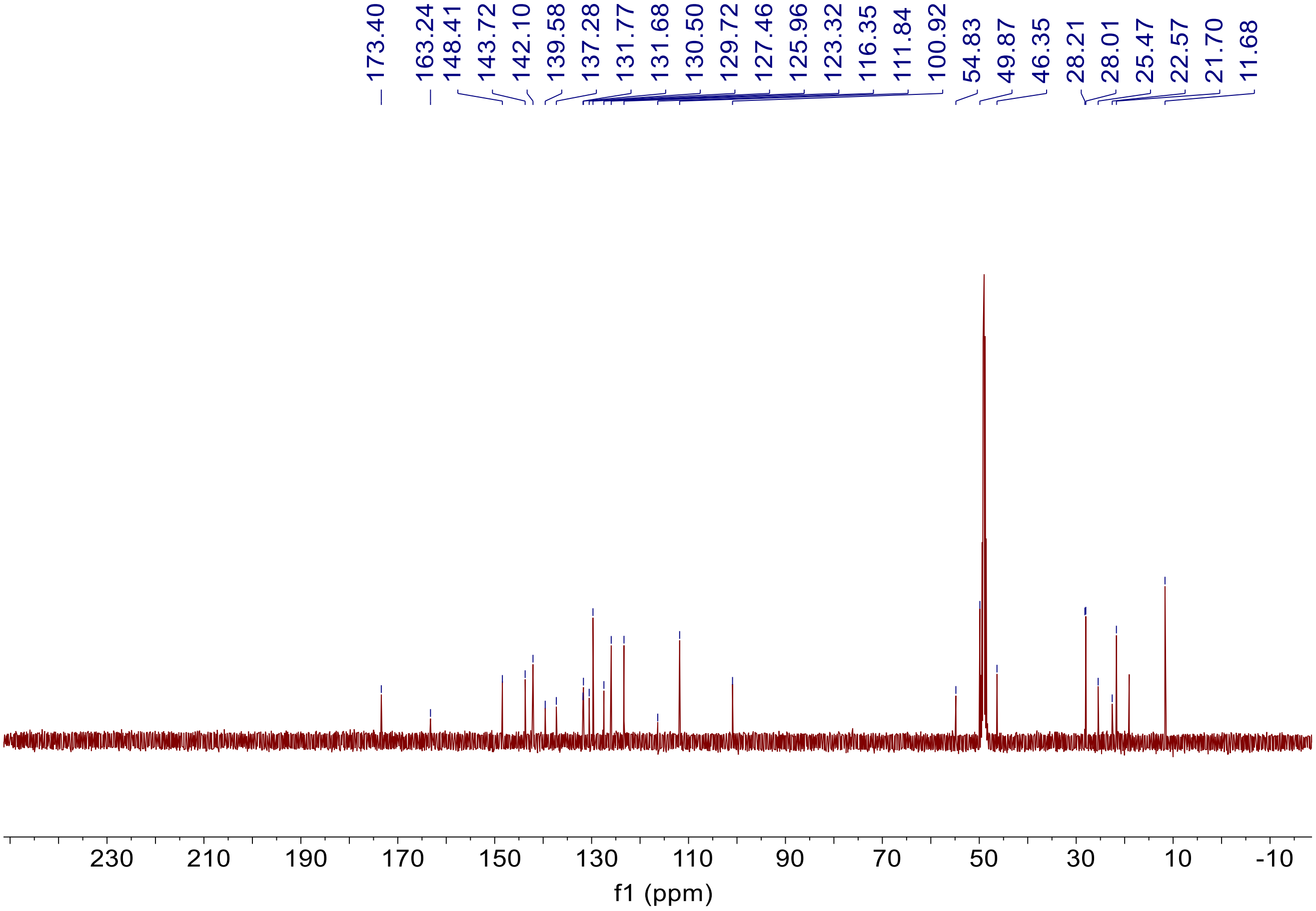
173.40
163.24
148.41
143.72
142.10
139.58
137.28
131.77
131.68
130.50
129.72
127.46
125.96
123.32
116.35
111.84
100.92
54.83
49.87
46.35
28.21
28.01
25.47
22.57
21.70
11.68
230 210 190 170 150 130 110 90 70 50 30 10 -10
f1 (ppm)

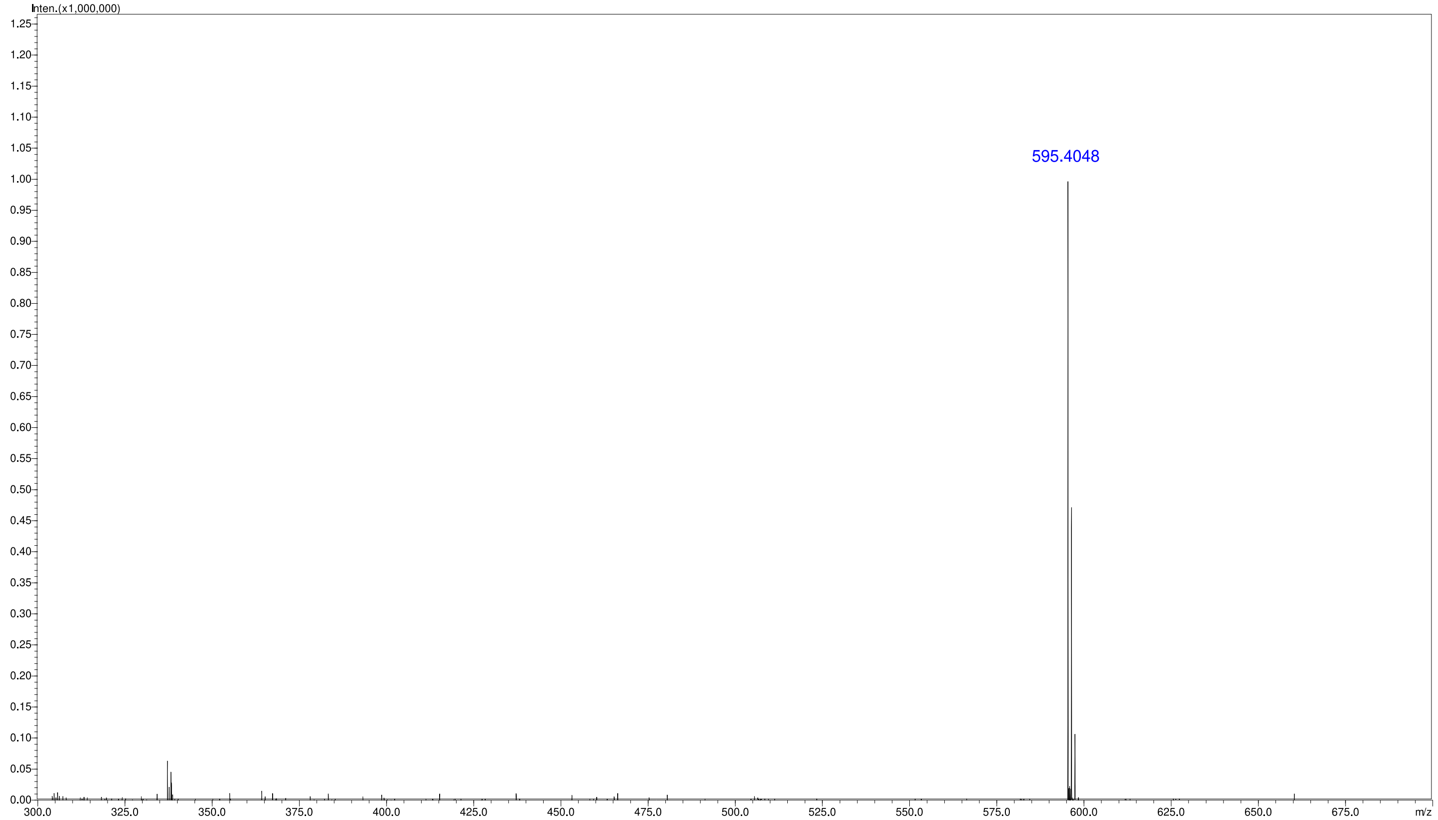
Inten.(x1,000,000)
595.4048
m/z

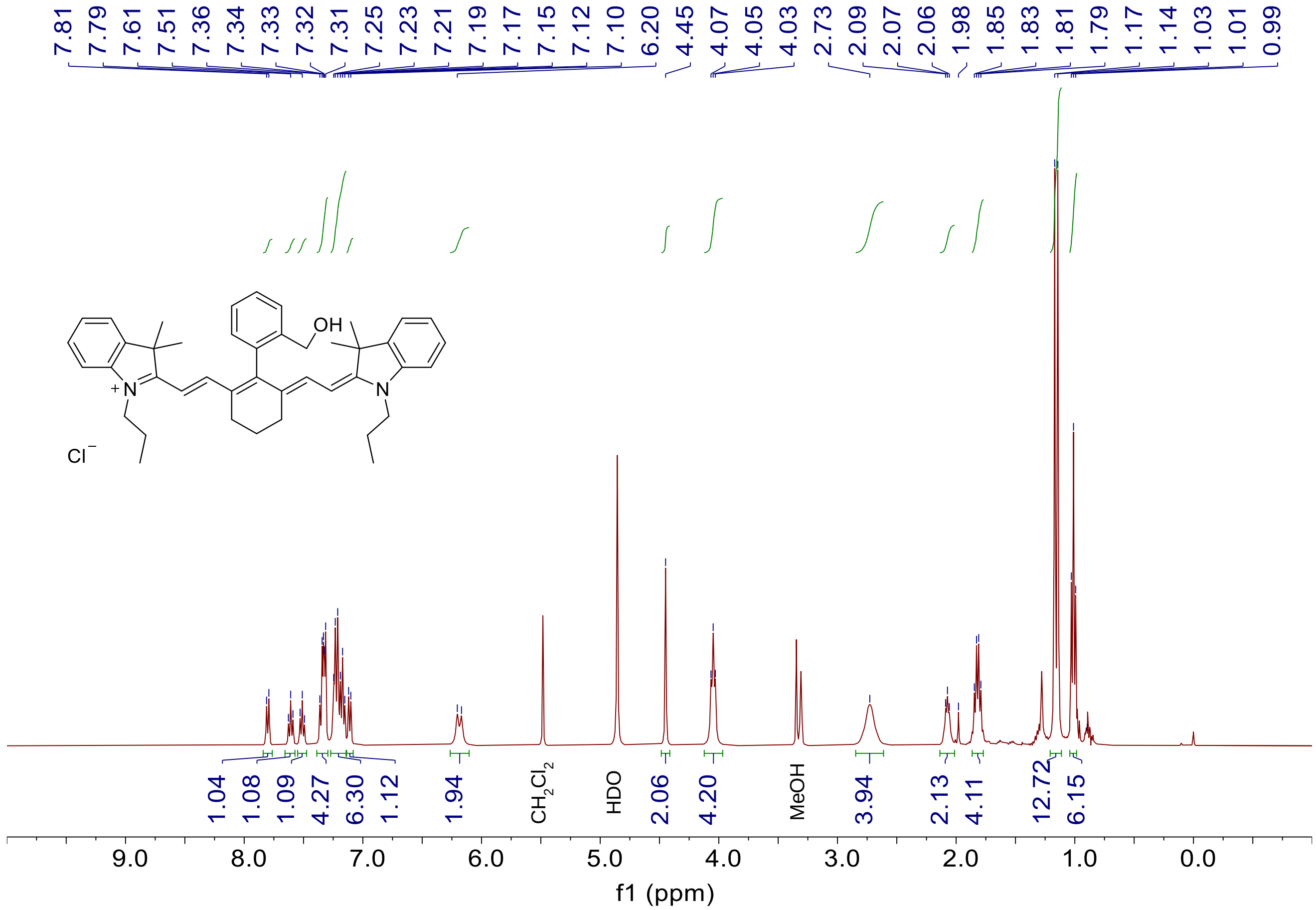
7.81 7.79 7.61 7.51 7.36 7.34 7.33 7.32 7.31 7.25 7.23 7.21 7.19 7.17 7.15 7.12 7.10 6.20 4.45 4.07 4.05 4.03 2.73 2.09 2.07 2.06 1.98 1.85 1.83 1.81 1.79 1.17 1.14 1.03 1.01 0.99
OH
N
+N
Cl⁻
1.04 1.08 1.09 4.27 6.30 1.12 1.94
CH2Cl2
HDO
2.06 4.20
MeOH
3.94 2.13 4.11 12.72 6.15
9.0 8.0 7.0 6.0 5.0 4.0 3.0 2.0 1.0 0.0
f1 (ppm)

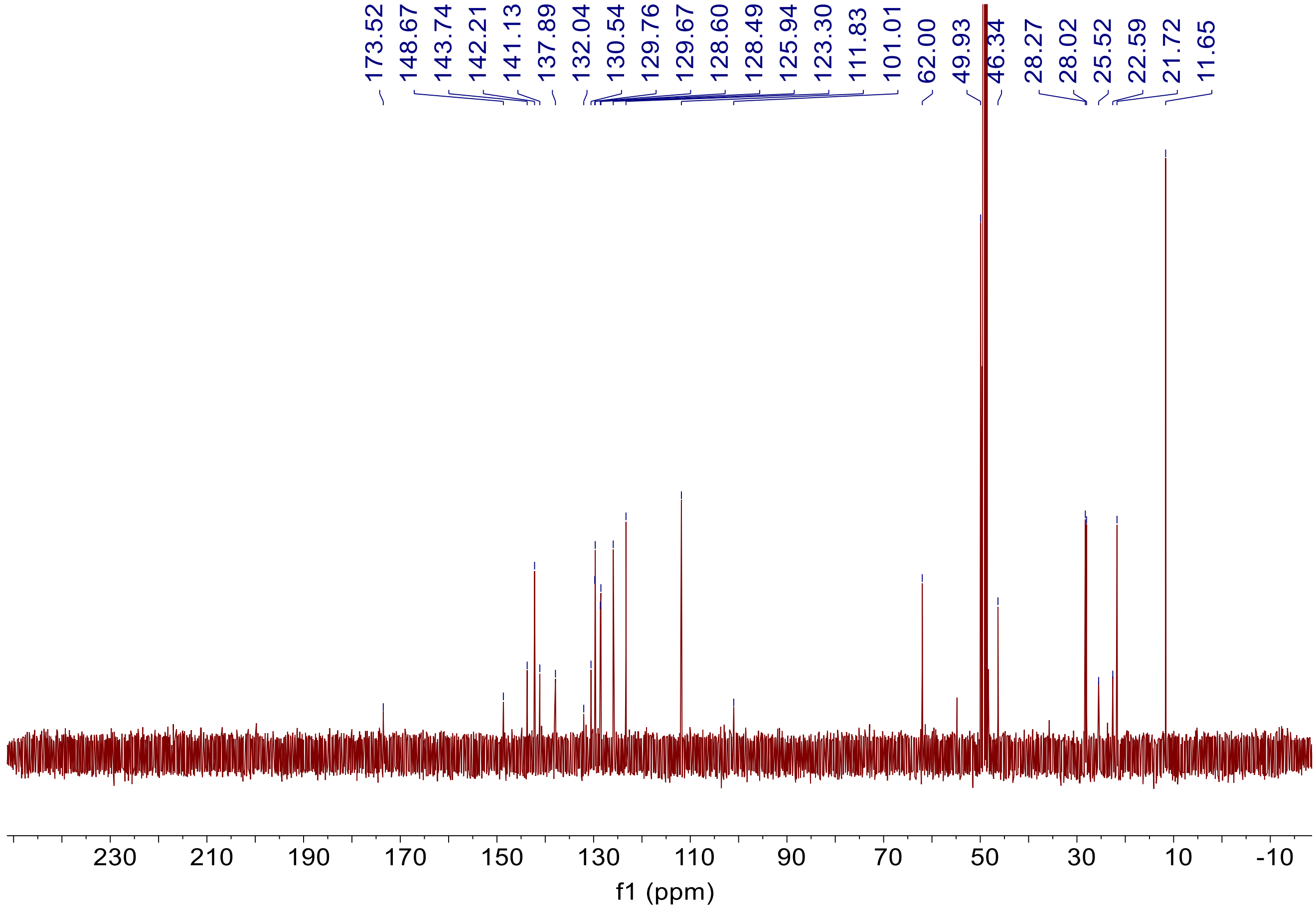
173.52 148.67 143.74 142.21 141.13 137.89 132.04 130.54 129.76 129.67 128.60 128.49 125.94 123.30 111.83 101.01 62.00 49.93 46.34 28.27 28.02 25.52 22.59 21.72 11.65
230 210 190 170 150 130 110 90 70 50 30 10 -10
f1 (ppm)

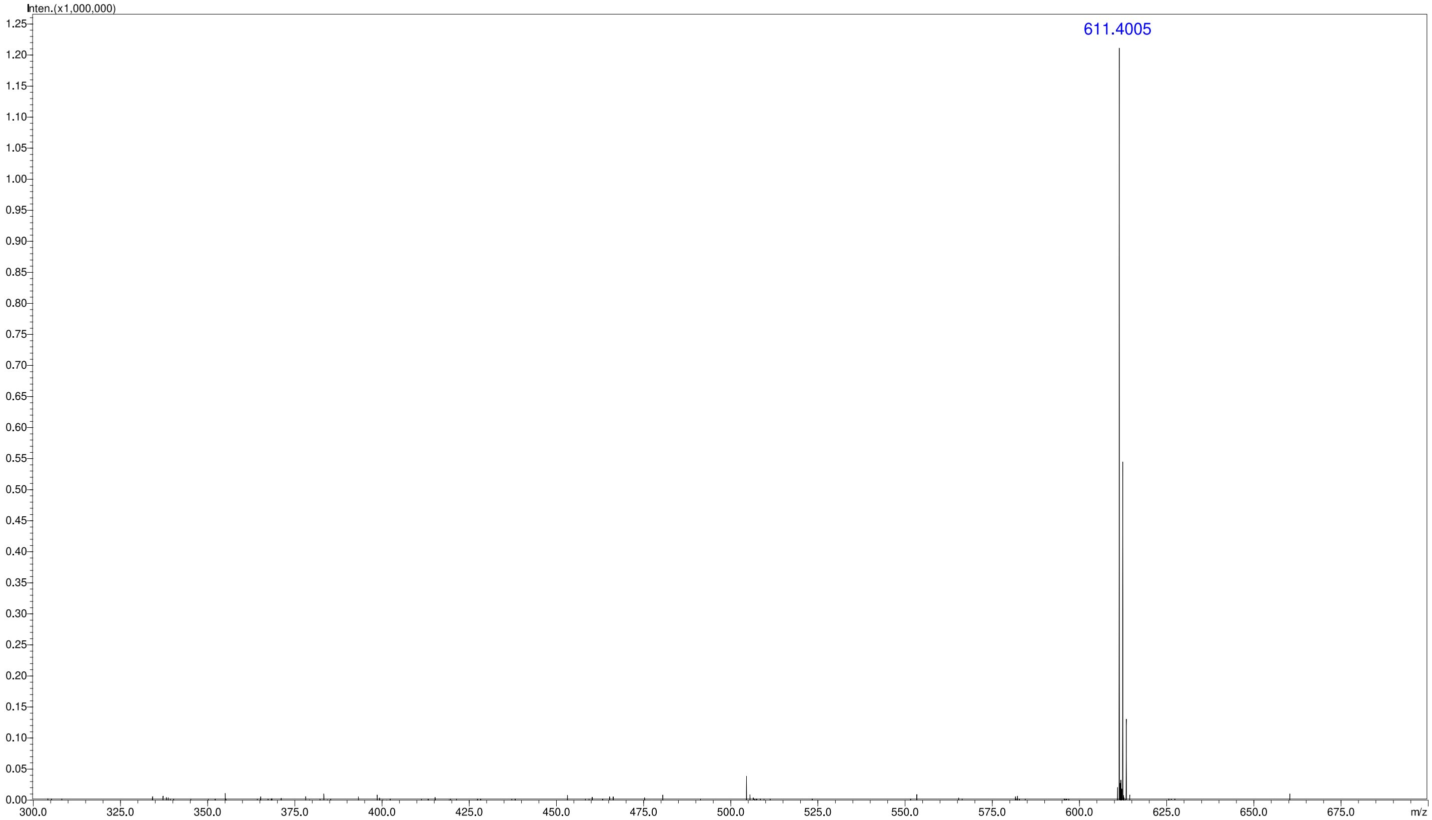
Inten.(x1,000,000)
611.4005
m/z

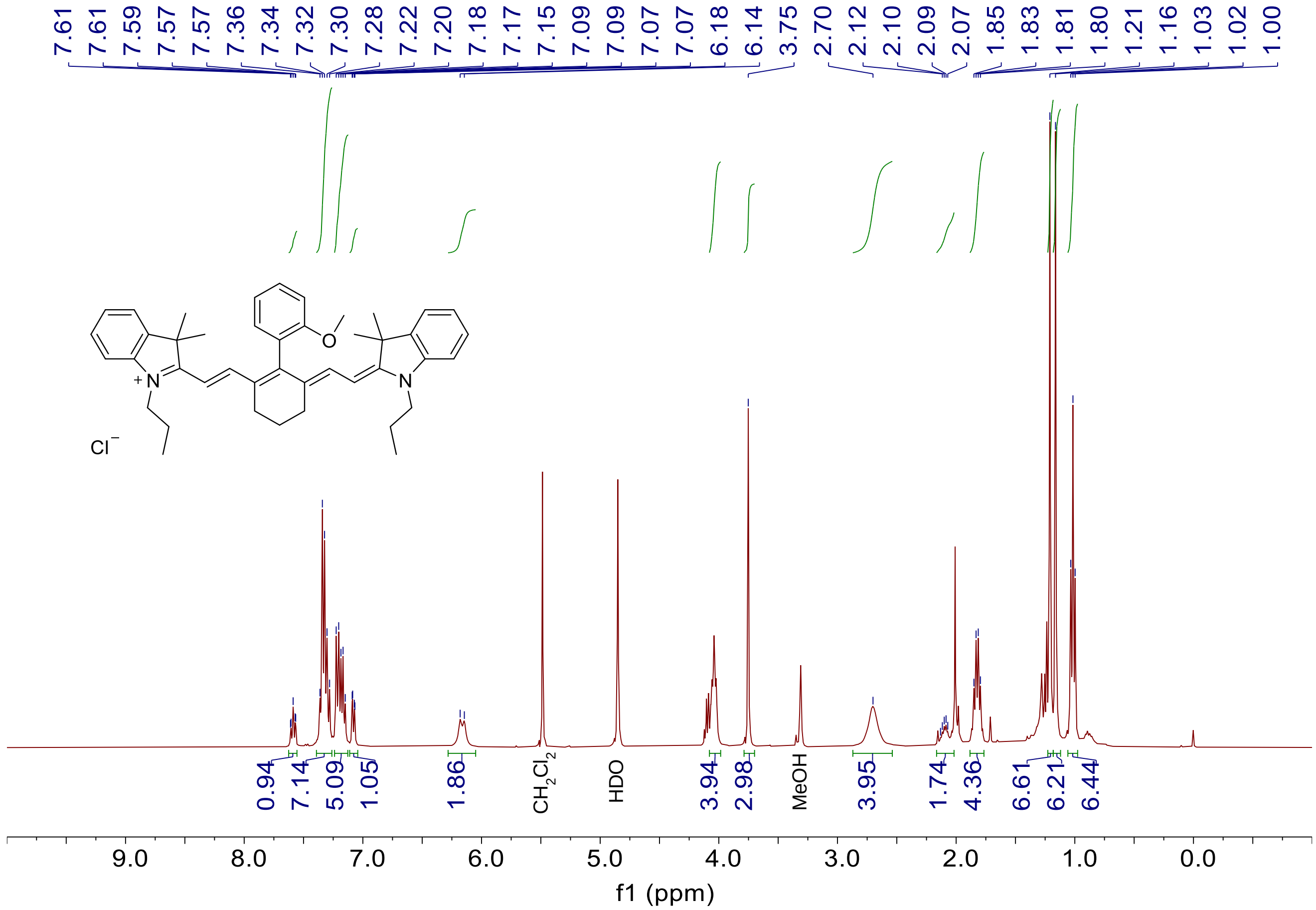

7.61 7.61 7.59 7.57 7.57 7.36 7.34 7.32 7.30 7.28 7.22 7.20 7.18 7.17 7.15 7.09 7.09 7.07 7.07 6.18 6.14 3.75 2.70 2.12 2.10 2.09 2.07 1.85 1.83 1.81 1.80 1.21 1.16 1.03 1.02 1.00
0.94 7.14 5.09 1.05 1.86 CH_2Cl_2 HDO 3.94 2.98 MeOH 3.95 1.74 4.36 6.61 6.21 6.44
9.0 8.0 7.0 6.0 5.0 4.0 3.0 2.0 1.0 0.0
f1 (ppm)
O
N
N
Cl−

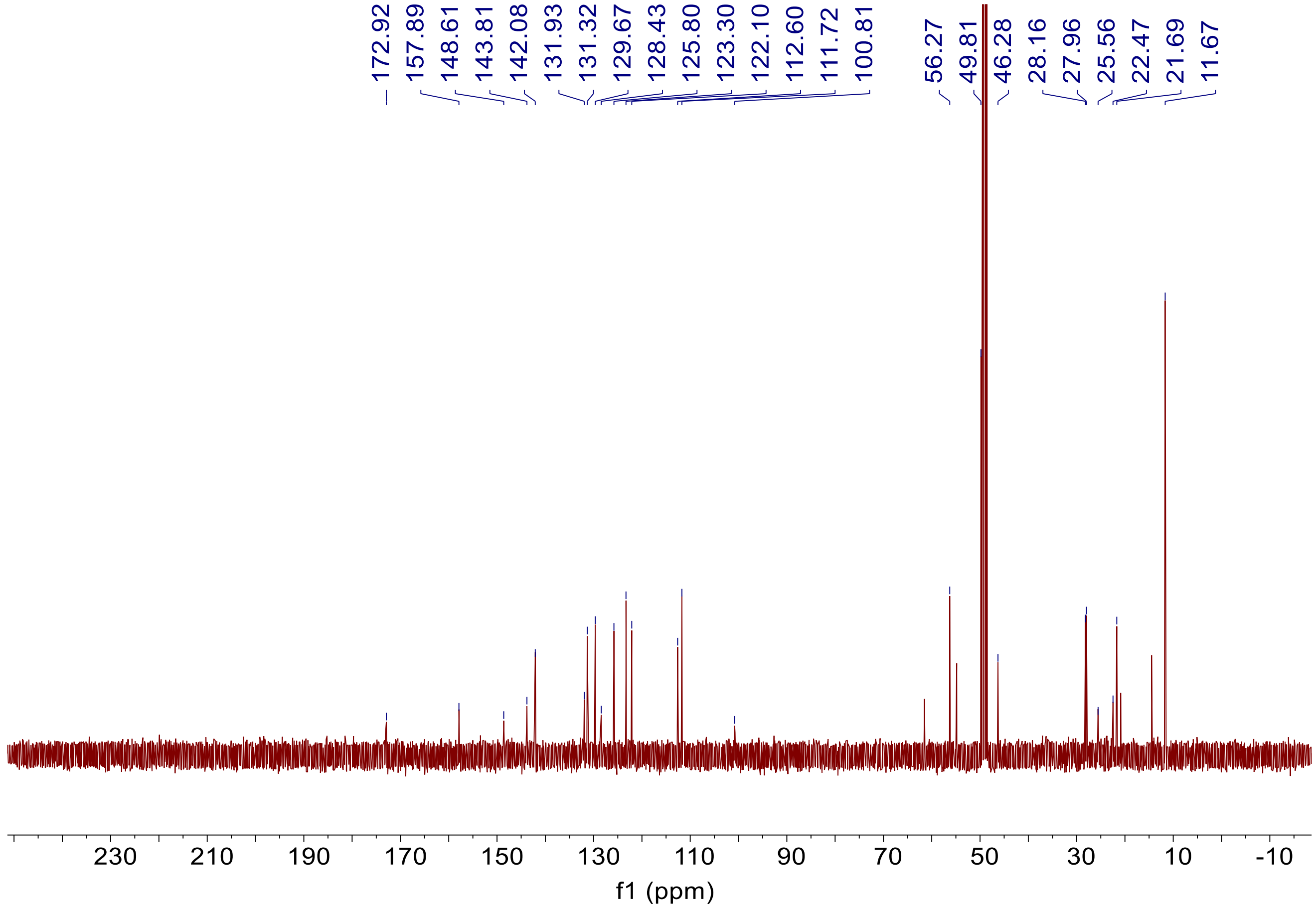

172.92 157.89 148.61 143.81 142.08 131.93 131.32 129.67 128.43 125.80 123.30 122.10 112.60 111.72 100.81 56.27 49.81 46.28 28.16 27.96 25.56 22.47 21.69 11.67
230 210 190 170 150 130 110 90 70 50 30 10 -10
f1 (ppm)

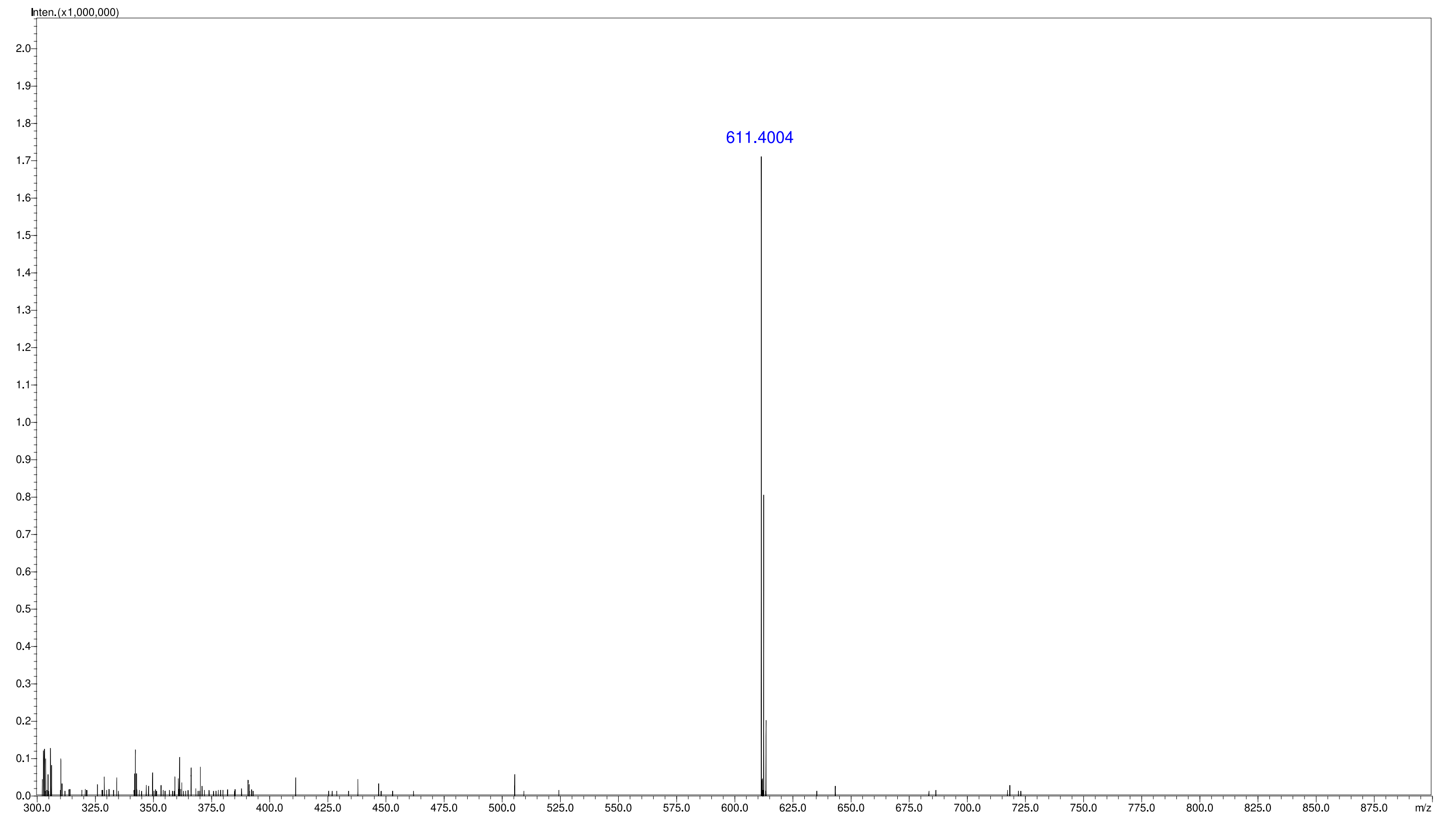
Inten.(x1,000,000)
611.4004
m/z

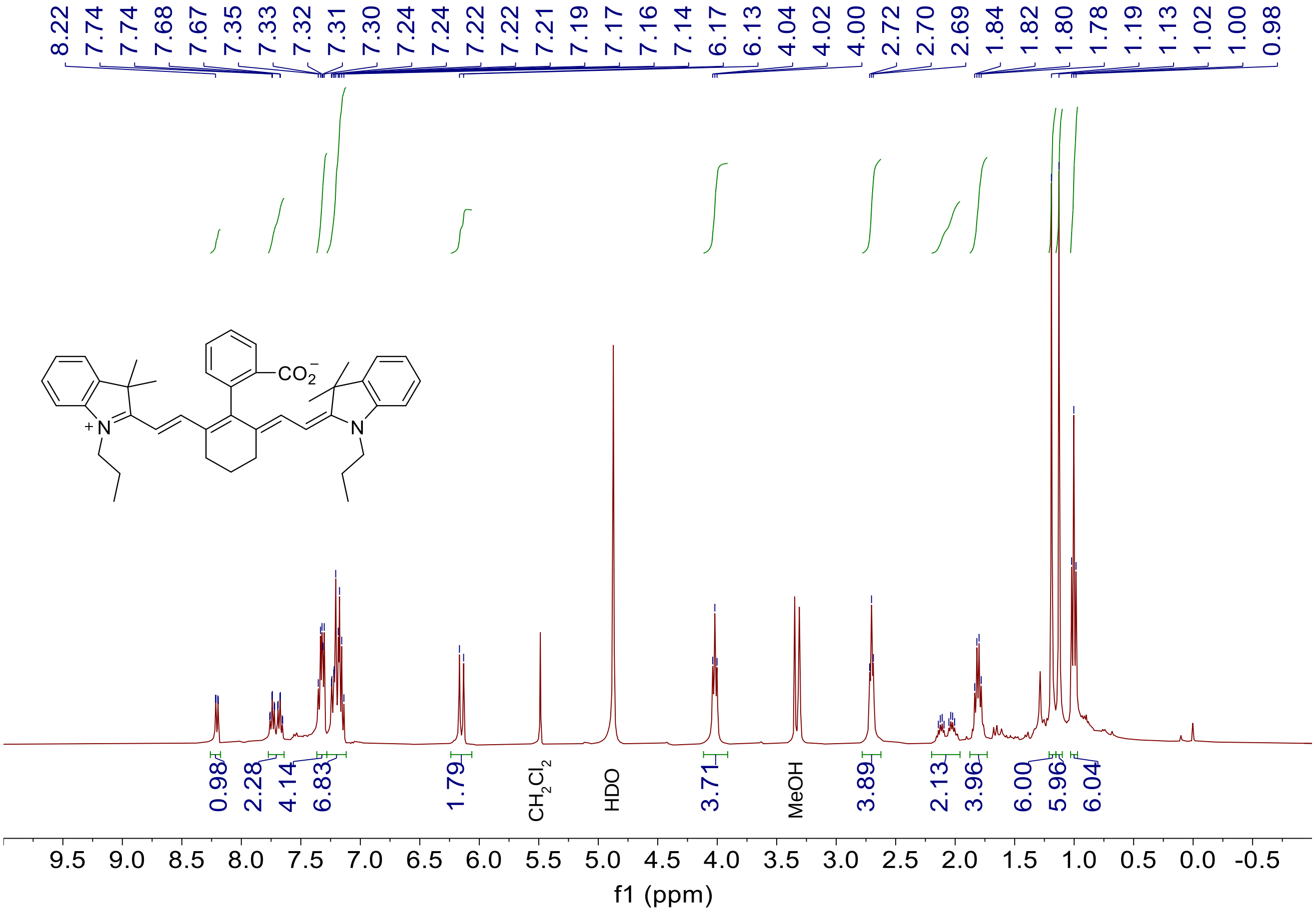
8.22 7.74 7.74 7.68 7.67 7.35 7.33 7.32 7.31 7.30 7.24 7.24 7.22 7.22 7.21 7.19 7.17 7.16 7.14 6.17 6.13 4.04 4.02 4.00 2.72 2.70 2.69 1.84 1.82 1.80 1.78 1.19 1.13 1.02 1.00 0.98
CO_2^-
N
$^+$N
0.98 2.28 4.14 6.83 1.79 3.71 3.89 2.13 3.96 6.00 5.96 6.04
CH_2Cl_2
HDO
MeOH
9.5 9.0 8.5 8.0 7.5 7.0 6.5 6.0 5.5 5.0 4.5 4.0 3.5 3.0 2.5 2.0 1.5 1.0 0.5 0.0 -0.5
f1 (ppm)

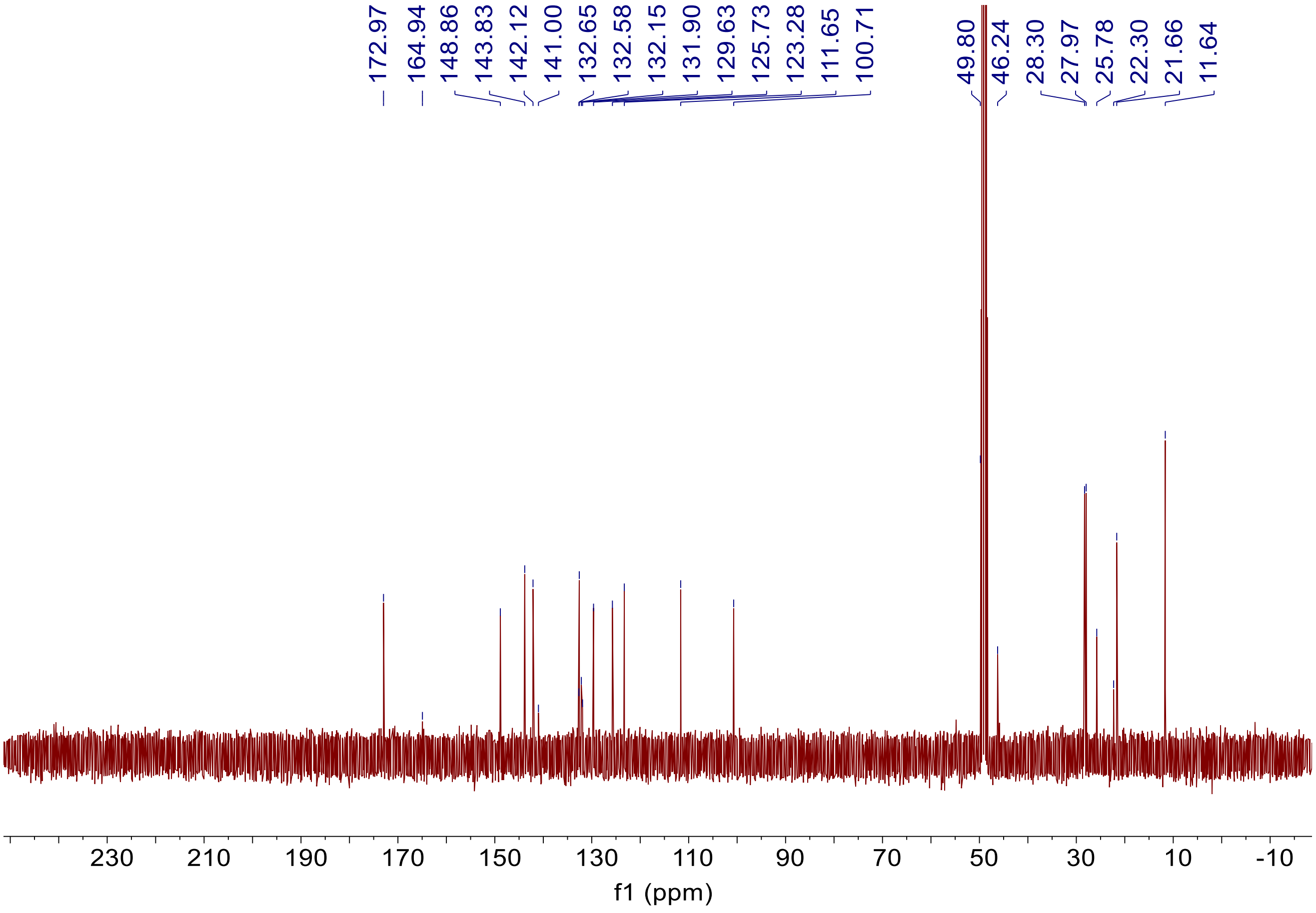
172.97 164.94 148.86 143.83 142.12 141.00 132.65 132.58 132.15 131.90 129.63 125.73 123.28 111.65 100.71
49.80 46.24 28.30 27.97 25.78 22.30 21.66 11.64
230 210 190 170 150 130 110 90 70 50 30 10 -10
f1 (ppm)

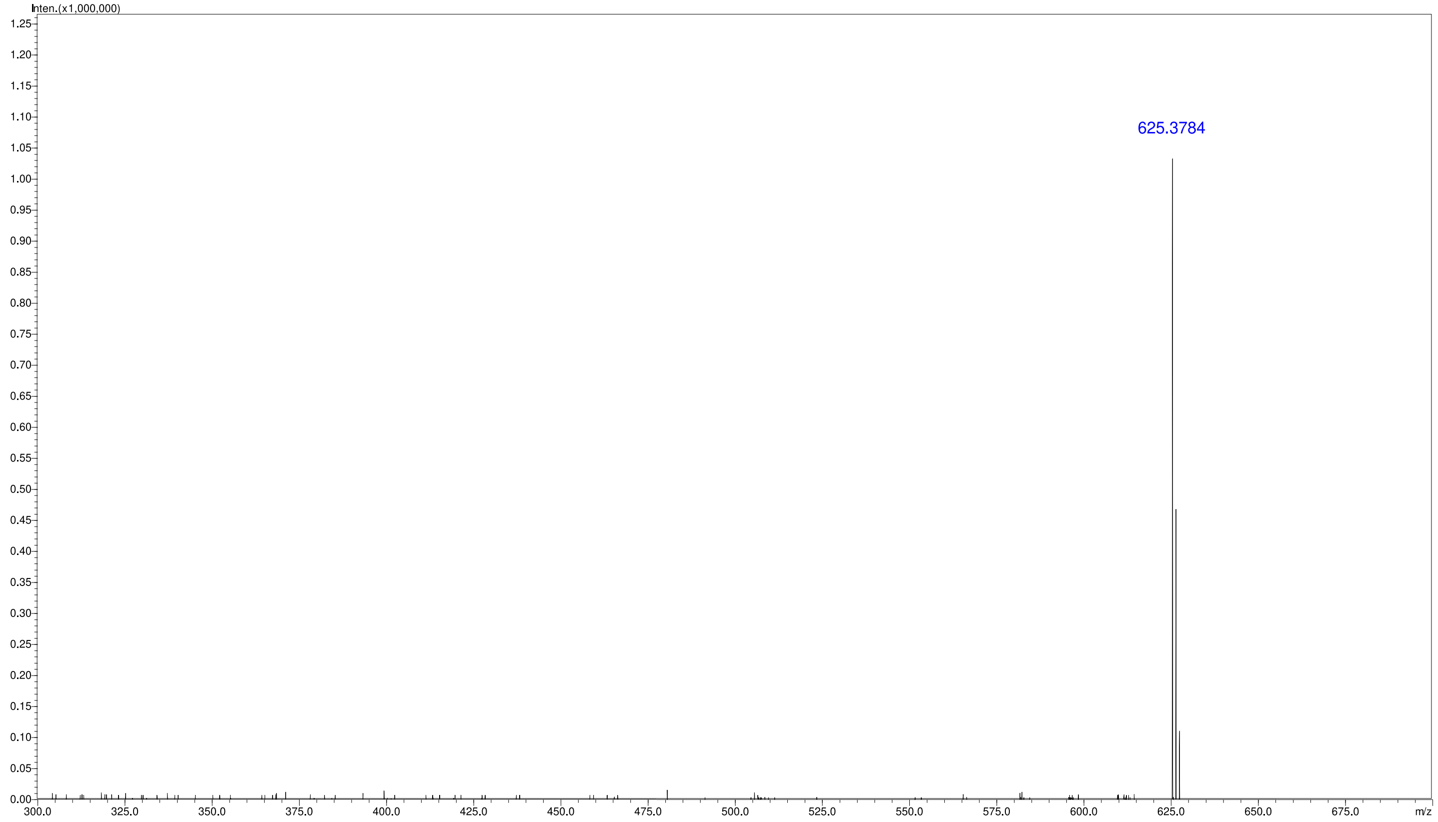
Inten.(x1,000,000)
625.3784
m/z

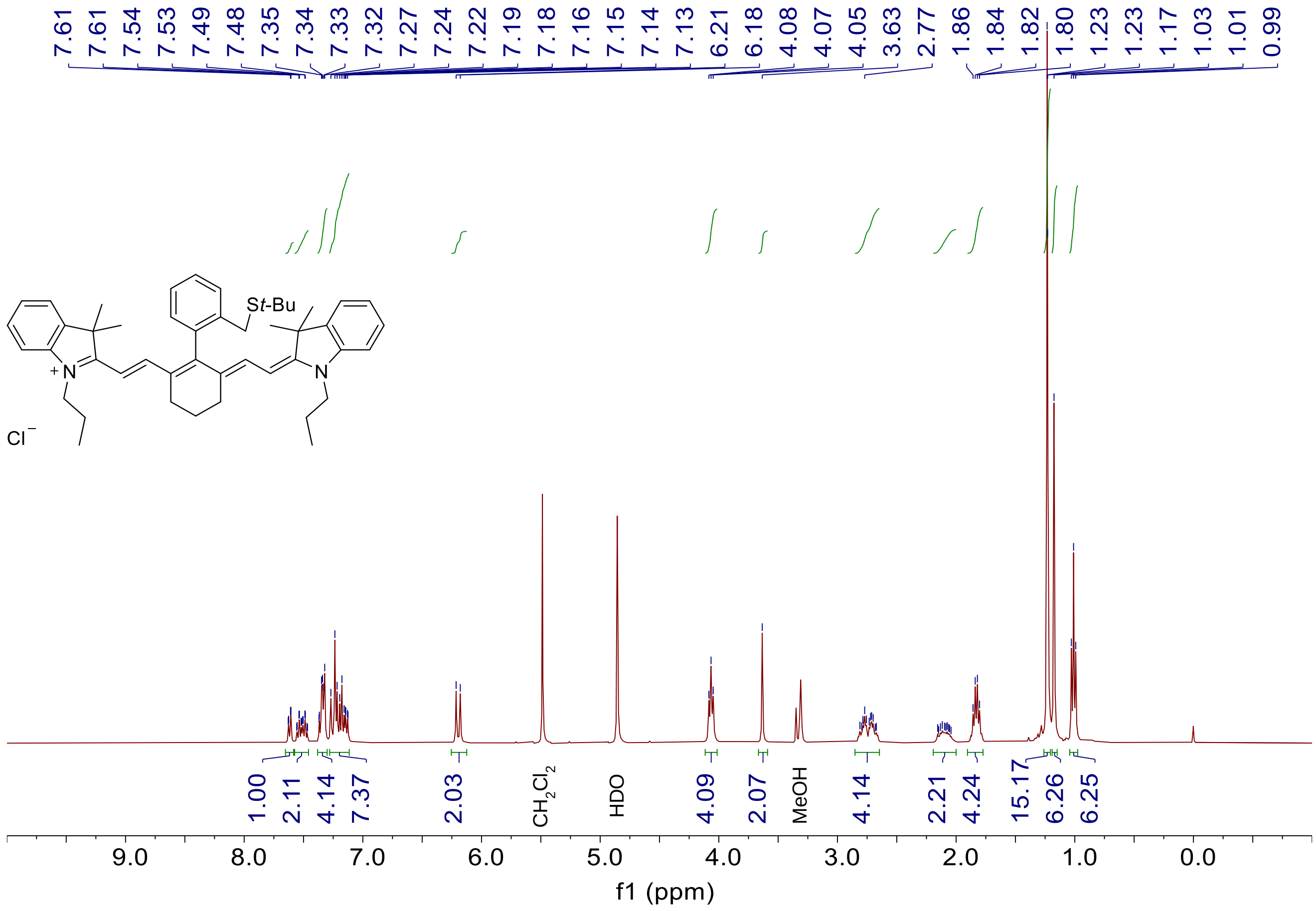
7.61 7.61 7.54 7.53 7.49 7.48 7.35 7.34 7.33 7.32 7.27 7.24 7.22 7.19 7.18 7.16 7.15 7.14 7.13 6.21 6.18 4.08 4.07 4.05 3.63 2.77 1.86 1.84 1.82 1.80 1.23 1.23 1.17 1.03 1.01 0.99
St-Bu
N
+N
Cl−
1.00 2.11 4.14 7.37 2.03 4.09 2.07 4.14 2.21 4.24 15.17 6.26 6.25
CH2Cl2
HDO
MeOH
9.0 8.0 7.0 6.0 5.0 4.0 3.0 2.0 1.0 0.0
f1 (ppm)

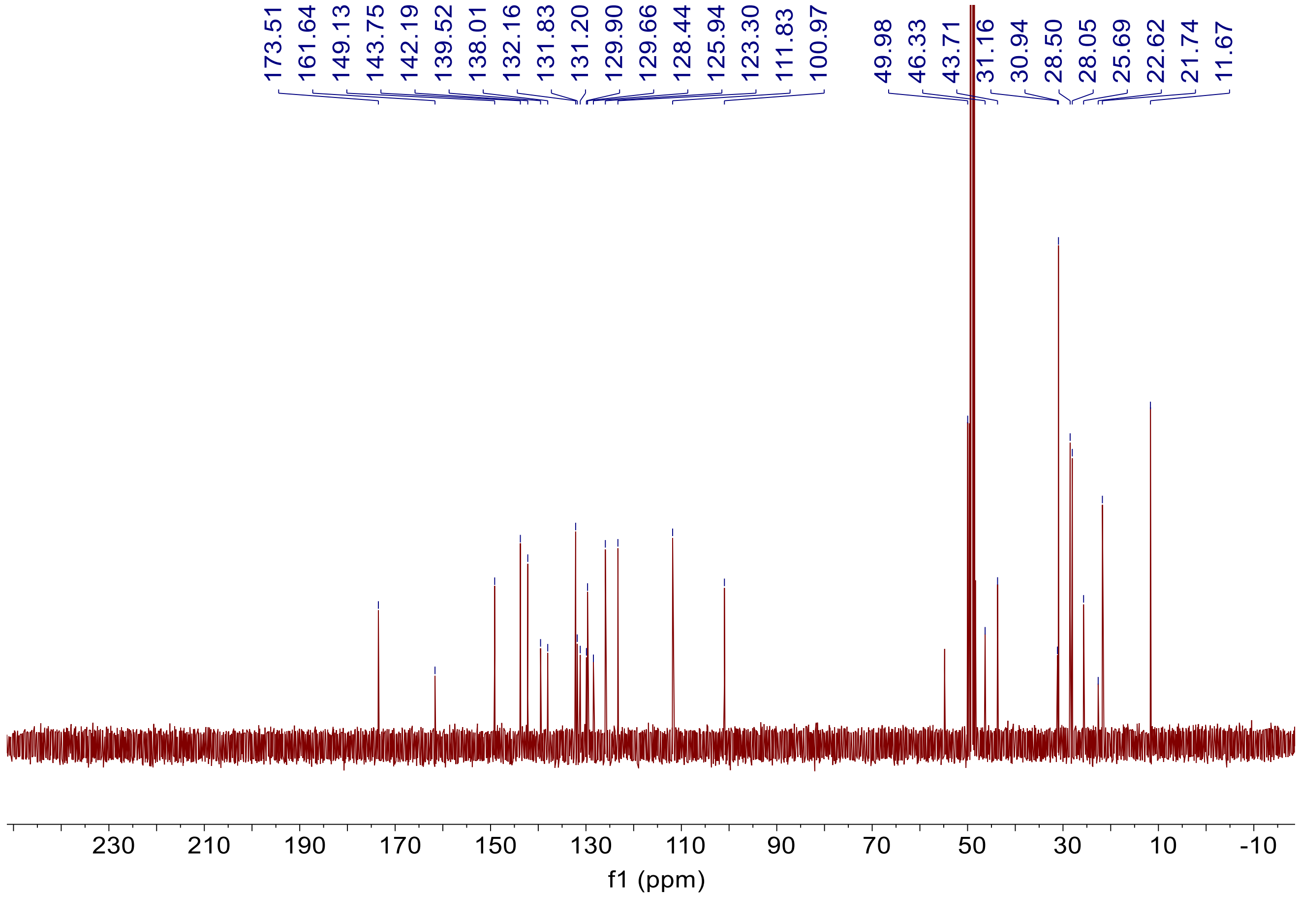
173.51 161.64 149.13 143.75 142.19 139.52 138.01 132.16 131.83 131.20 129.90 129.66 128.44 125.94 123.30 111.83 100.97
49.98 46.33 43.71 31.16 30.94 28.50 28.05 25.69 22.62 21.74 11.67
230 210 190 170 150 130 110 90 70 50 30 10 -10
f1 (ppm)

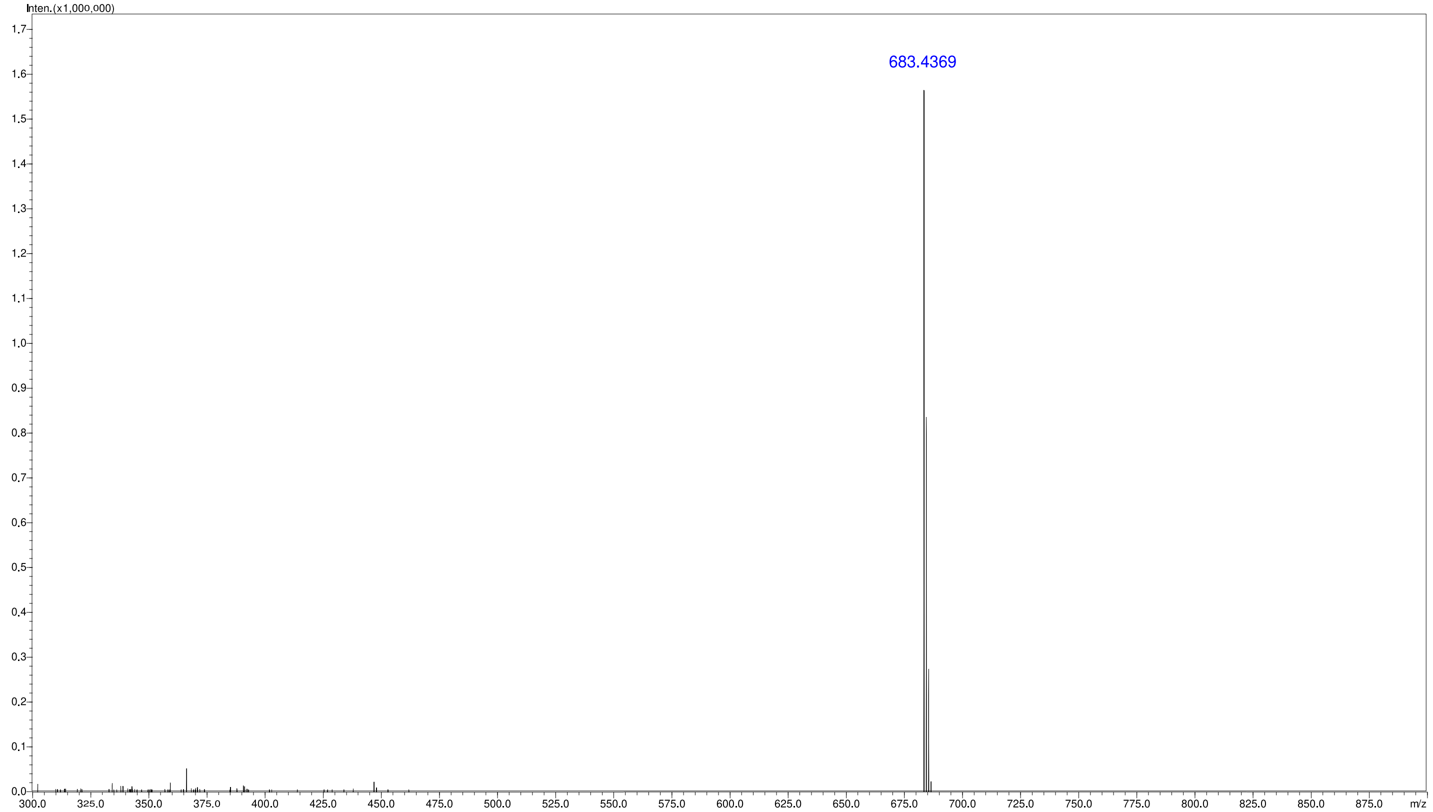
Inten.(x1,000,000)
683.4369
m/z

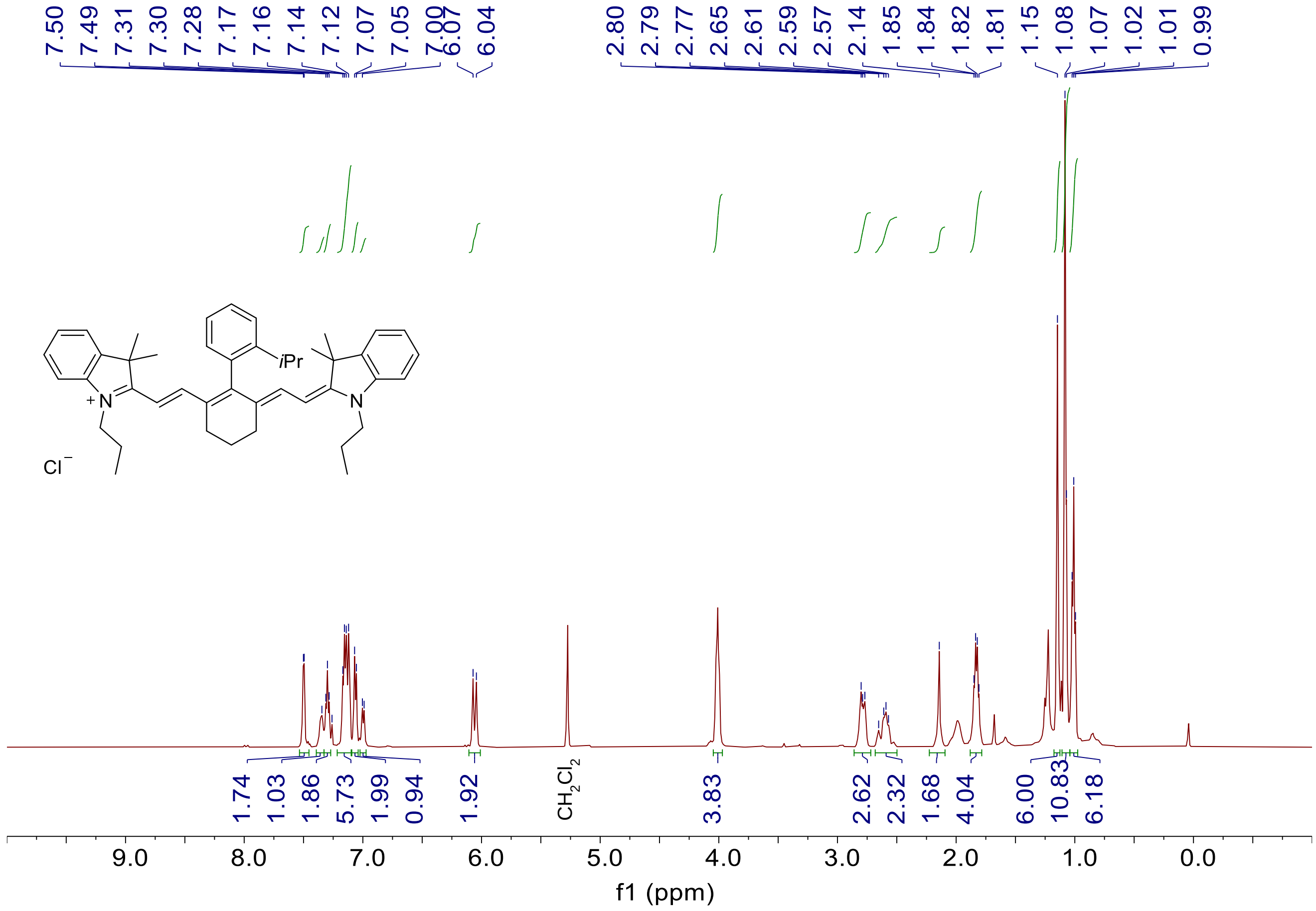

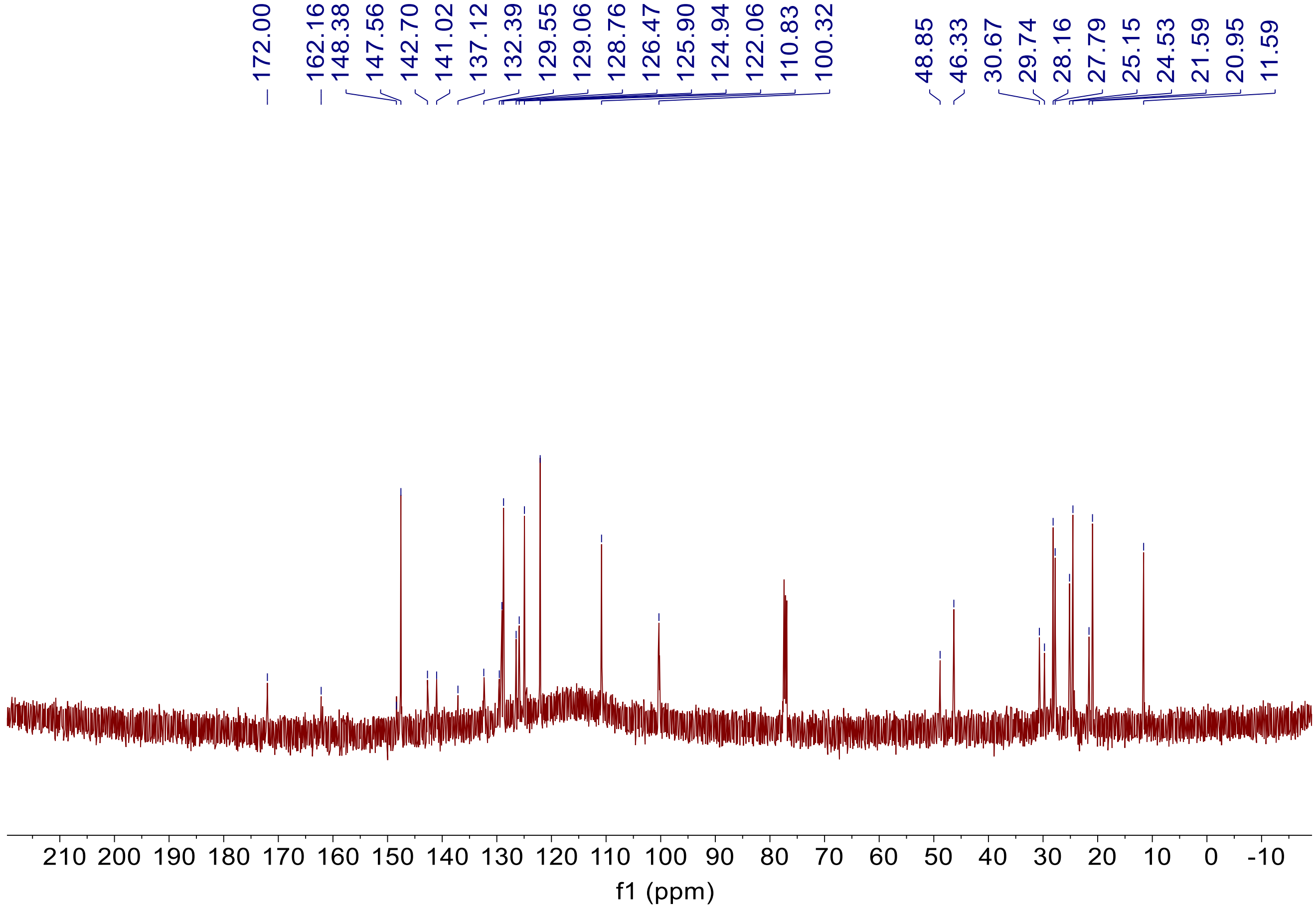

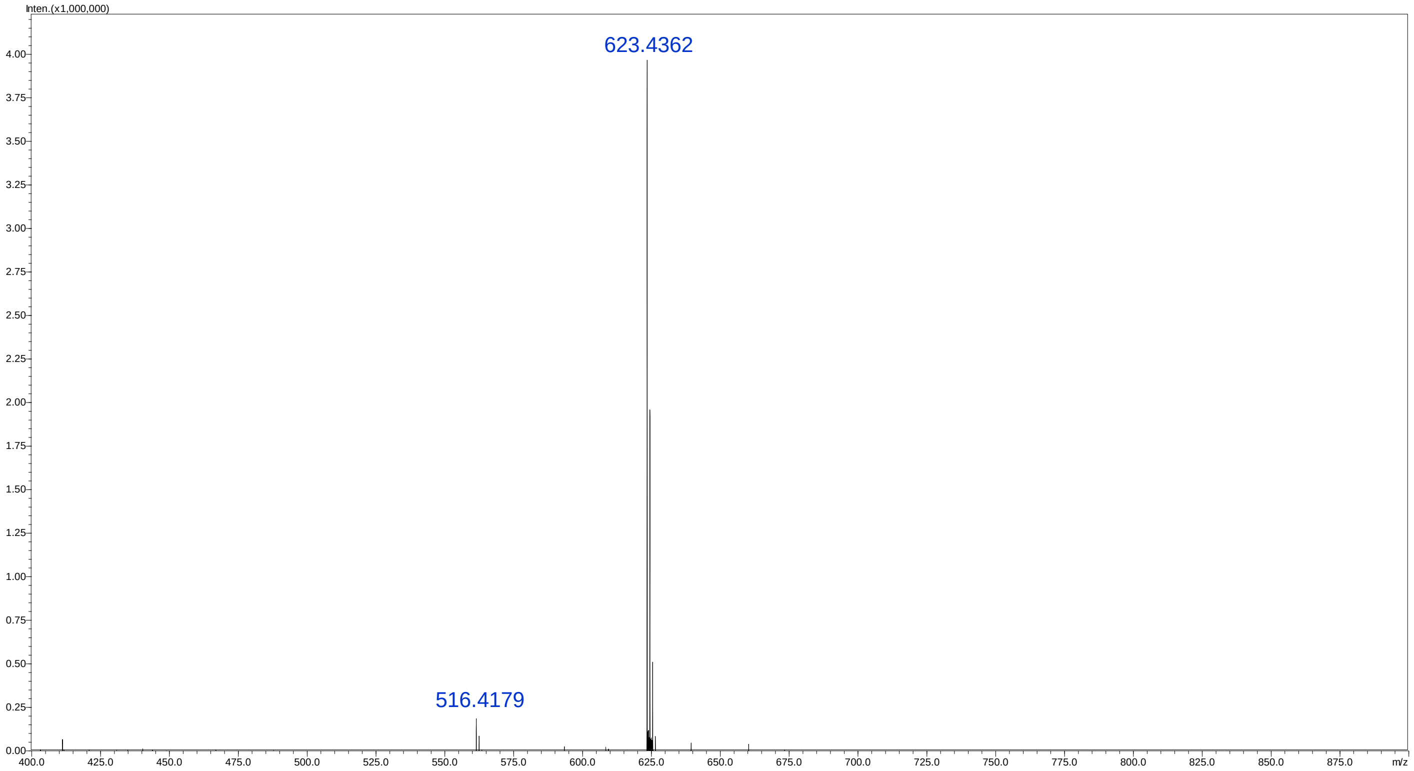

Inten.(x1,000,000)
623.4362
516.4179
4.00
3.75
3.50
3.25
3.00
2.75
2.50
2.25
2.00
1.75
1.50
1.25
1.00
0.75
0.50
0.25
0.00
400.0
425.0
450.0
475.0
500.0
525.0
550.0
575.0
600.0
625.0
650.0
675.0
700.0
725.0
750.0
775.0
800.0
825.0
850.0
875.0
m/z

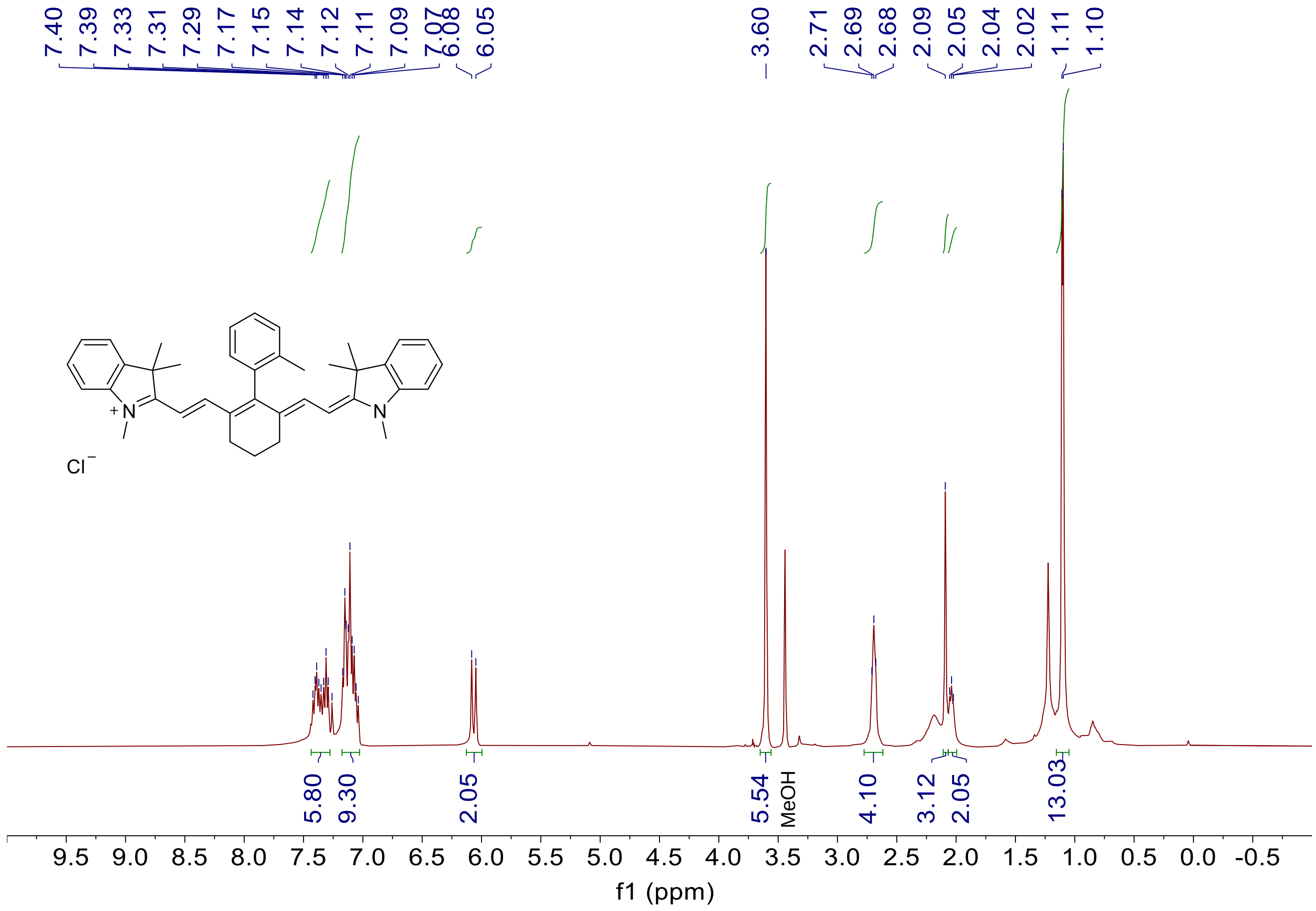
7.40 7.39 7.33 7.31 7.29 7.17 7.15 7.14 7.12 7.11 7.09 7.07 6.08 6.05
3.60
2.71 2.69 2.68 2.09 2.05 2.04 2.02 1.11 1.10
Cl⁻
5.80 9.30 2.05 5.54 MeOH 4.10 3.12 2.05 13.03
9.5 9.0 8.5 8.0 7.5 7.0 6.5 6.0 5.5 5.0 4.5 4.0 3.5 3.0 2.5 2.0 1.5 1.0 0.5 0.0 -0.5
f1 (ppm)

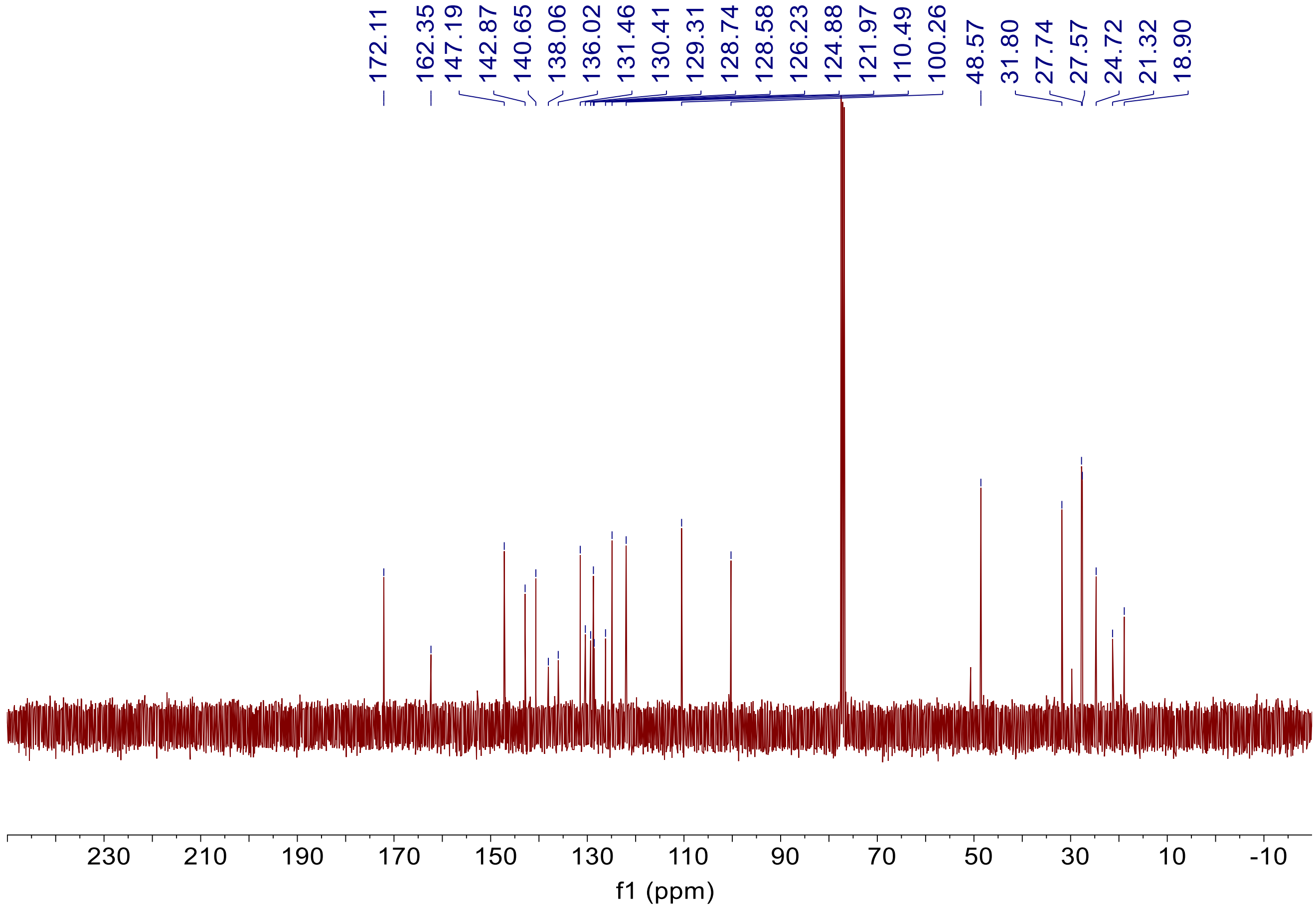
172.11 162.35 147.19 142.87 140.65 138.06 136.02 131.46 130.41 129.31 128.74 128.58 126.23 124.88 121.97 110.49 100.26 48.57 31.80 27.74 27.57 24.72 21.32 18.90
230 210 190 170 150 130 110 90 70 50 30 10 -10
f1 (ppm)

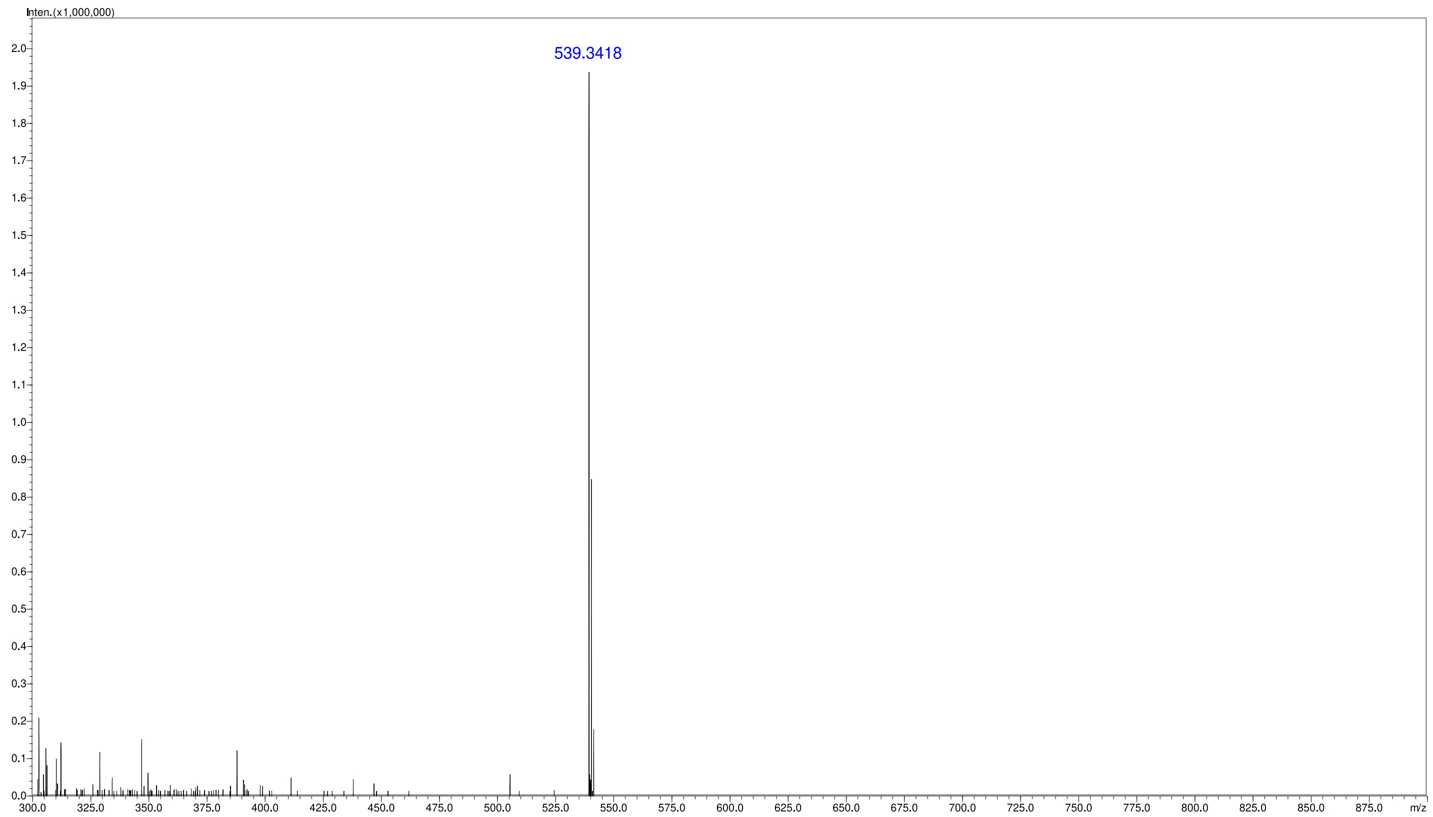
Inten.(x1,000,000)
539.3418
m/z
2.0
1.9
1.8
1.7
1.6
1.5
1.4
1.3
1.2
1.1
1.0
0.9
0.8
0.7
0.6
0.5
0.4
0.3
0.2
0.1
0.0
300.0
325.0
350.0
375.0
400.0
425.0
450.0
475.0
500.0
525.0
550.0
575.0
600.0
625.0
650.0
675.0
700.0
725.0
750.0
775.0
800.0
825.0
850.0
875.0

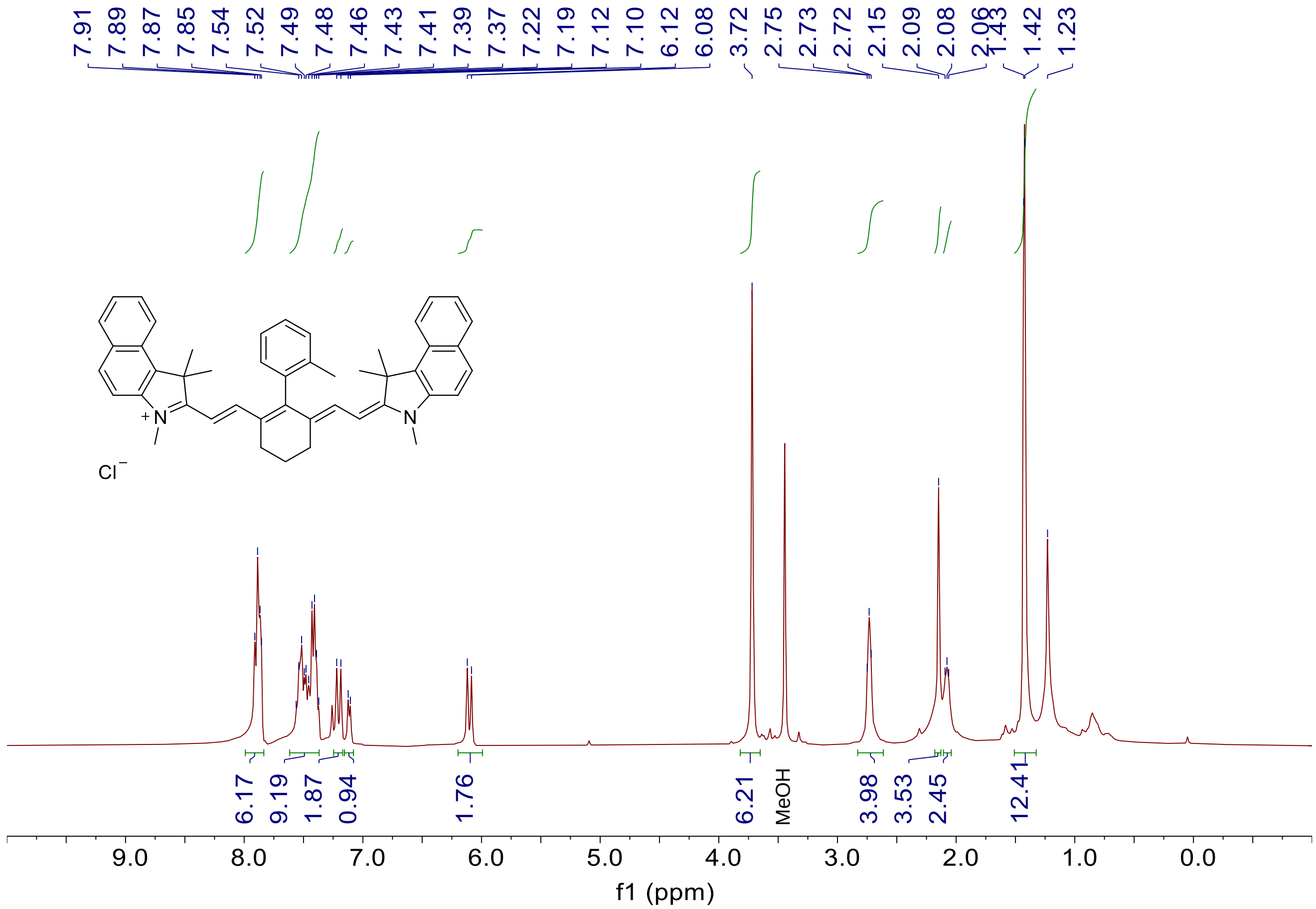
7.91 7.89 7.87 7.85 7.54 7.52 7.49 7.48 7.46 7.43 7.41 7.39 7.37 7.22 7.19 7.12 7.10 6.12 6.08 3.72 2.75 2.73 2.72 2.15 2.09 2.08 2.06 1.43 1.42 1.23
N
+N
Cl⁻
6.17 9.19 1.87 0.94 1.76 6.21 MeOH 3.98 3.53 2.45 12.41
9.0 8.0 7.0 6.0 5.0 4.0 3.0 2.0 1.0 0.0
f1 (ppm)

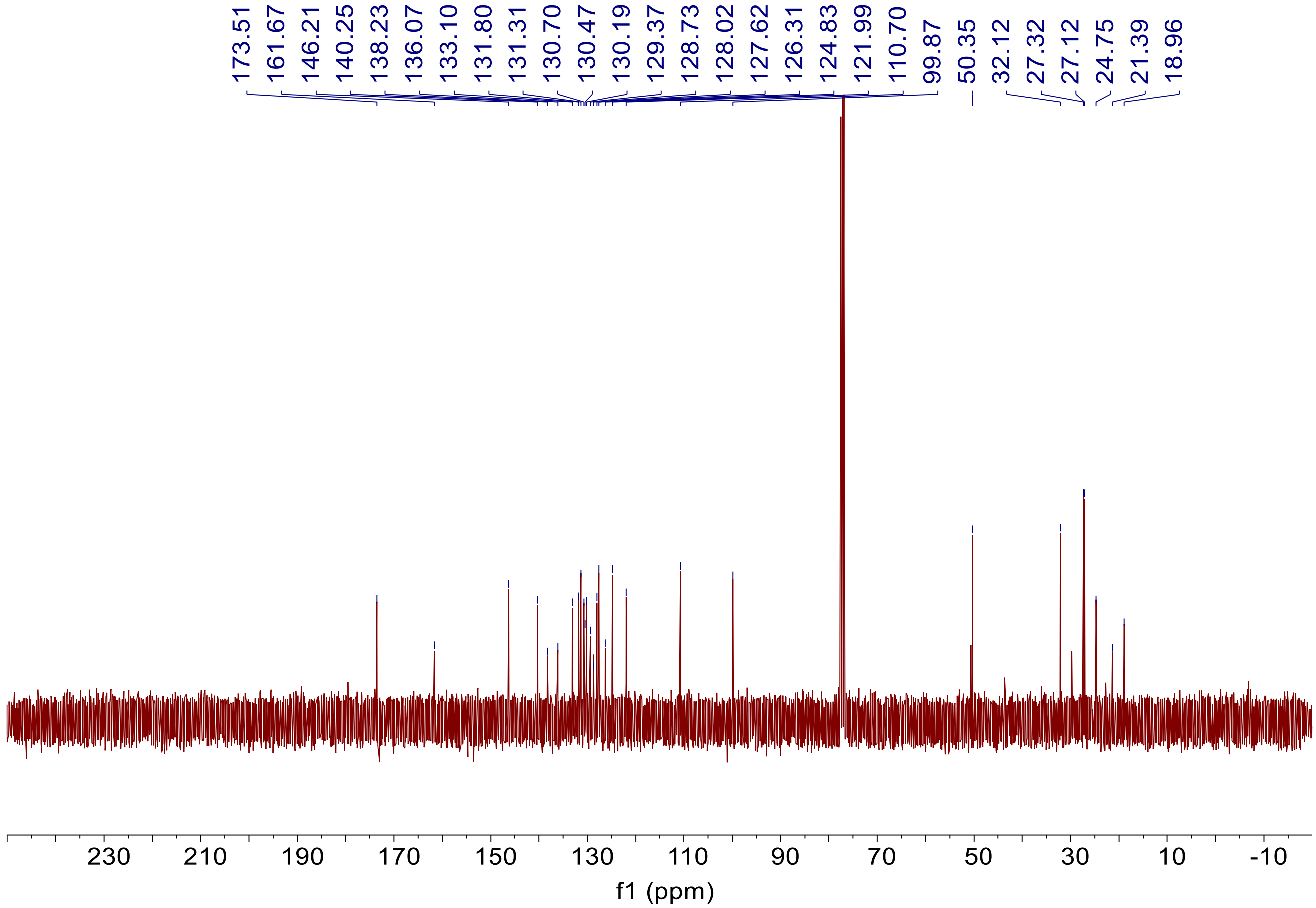
173.51 161.67 146.21 140.25 138.23 136.07 133.10 131.80 131.31 130.70 130.47 130.19 129.37 128.73 128.02 127.62 126.31 124.83 121.99 110.70 99.87 50.35 32.12 27.32 27.12 24.75 21.39 18.96
230 210 190 170 150 130 110 90 70 50 30 10 -10
f1 (ppm)

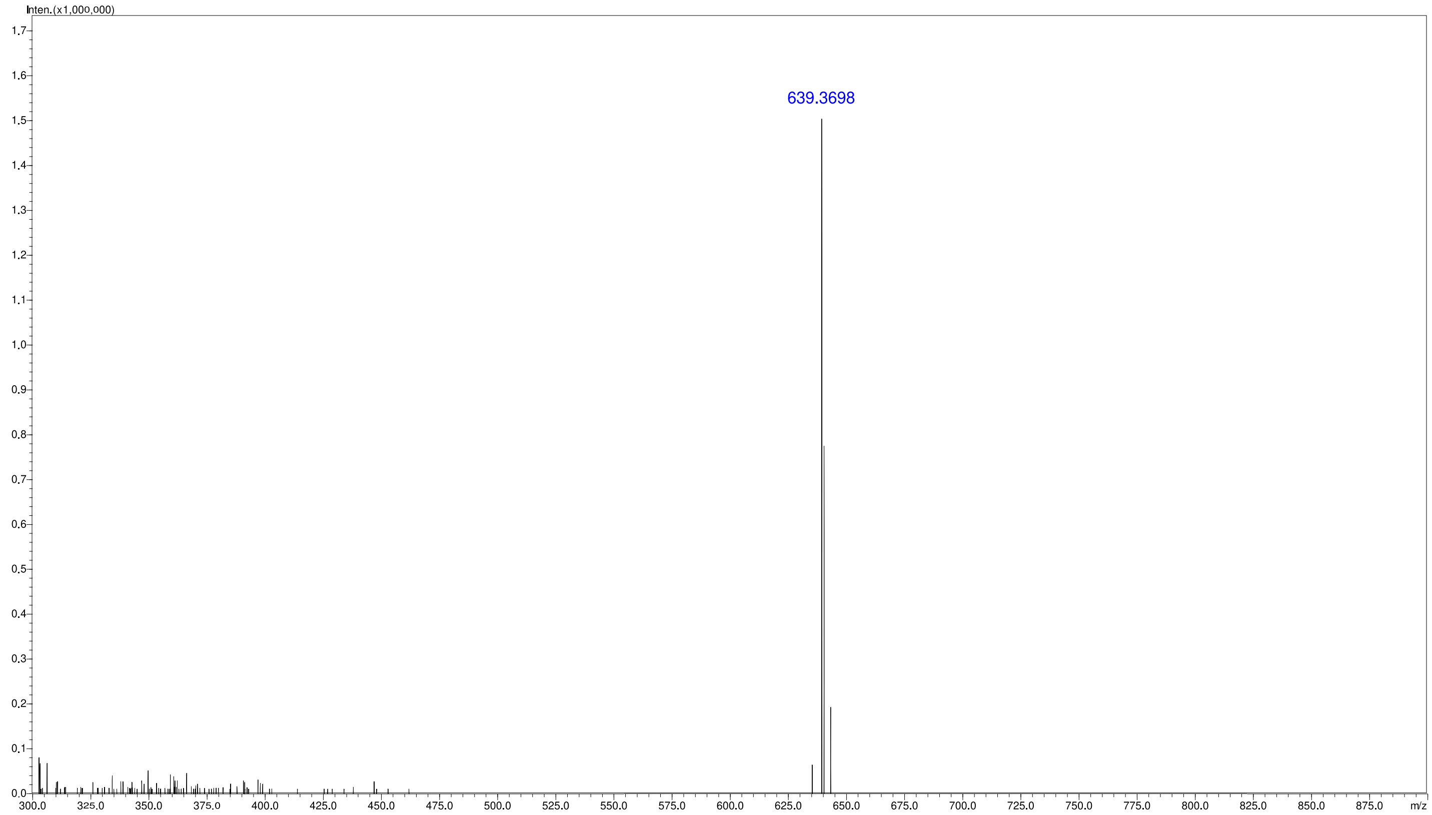
Inten.(x1,000,000)
639.3698
m/z

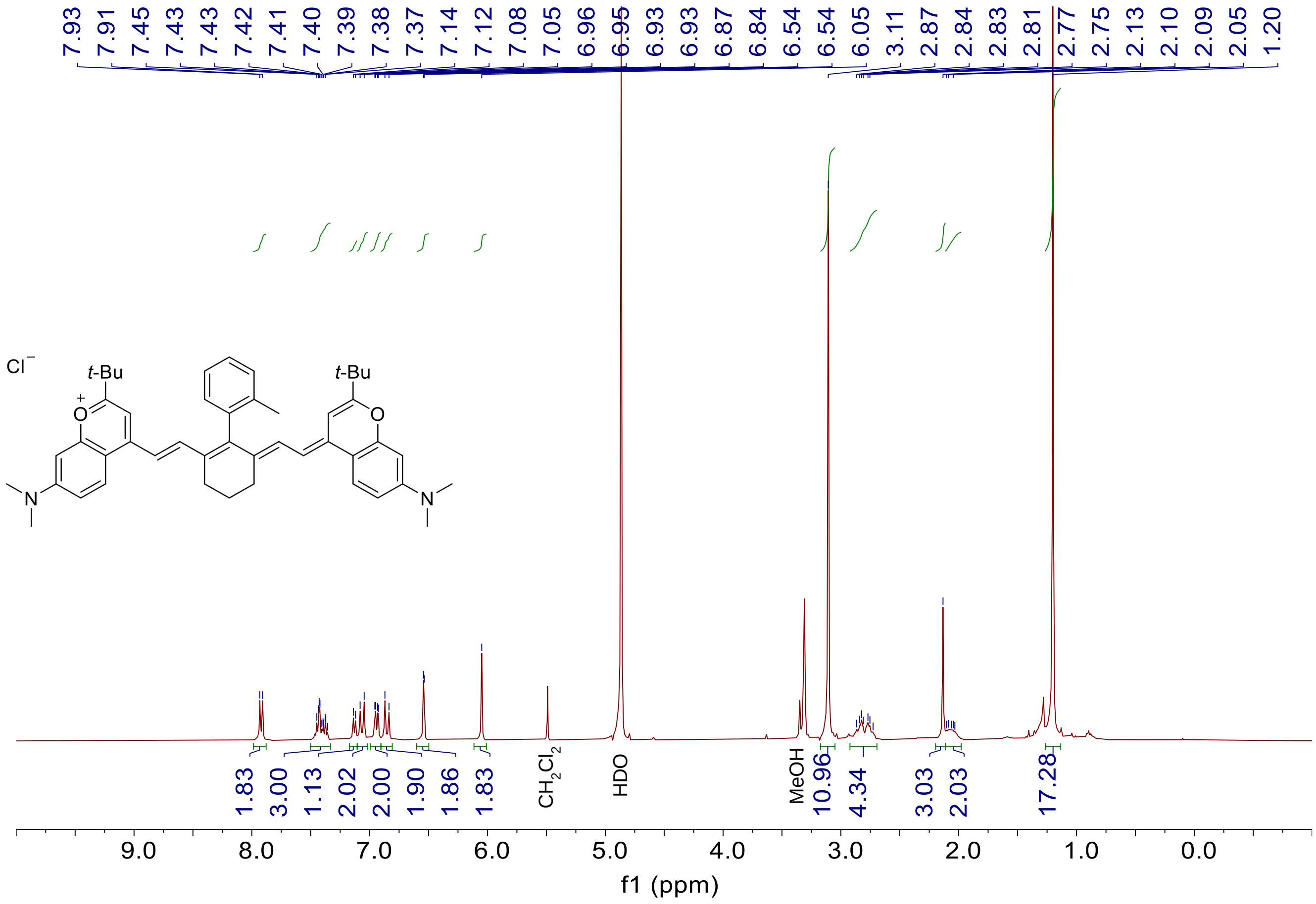
7.93
7.91
7.45
7.43
7.43
7.42
7.41
7.40
7.39
7.38
7.37
7.14
7.12
7.08
7.05
6.96
6.95
6.93
6.93
6.87
6.84
6.54
6.54
6.05
3.11
2.87
2.84
2.83
2.81
2.77
2.75
2.13
2.10
2.09
2.05
1.20
Cl⁻
t-Bu
t-Bu
1.83
3.00
1.13
2.02
2.00
1.90
1.86
1.83
CH2Cl2
HDO
MeOH
10.96
4.34
3.03
2.03
17.28
9.0
8.0
7.0
6.0
5.0
4.0
3.0
2.0
1.0
0.0
f1 (ppm)

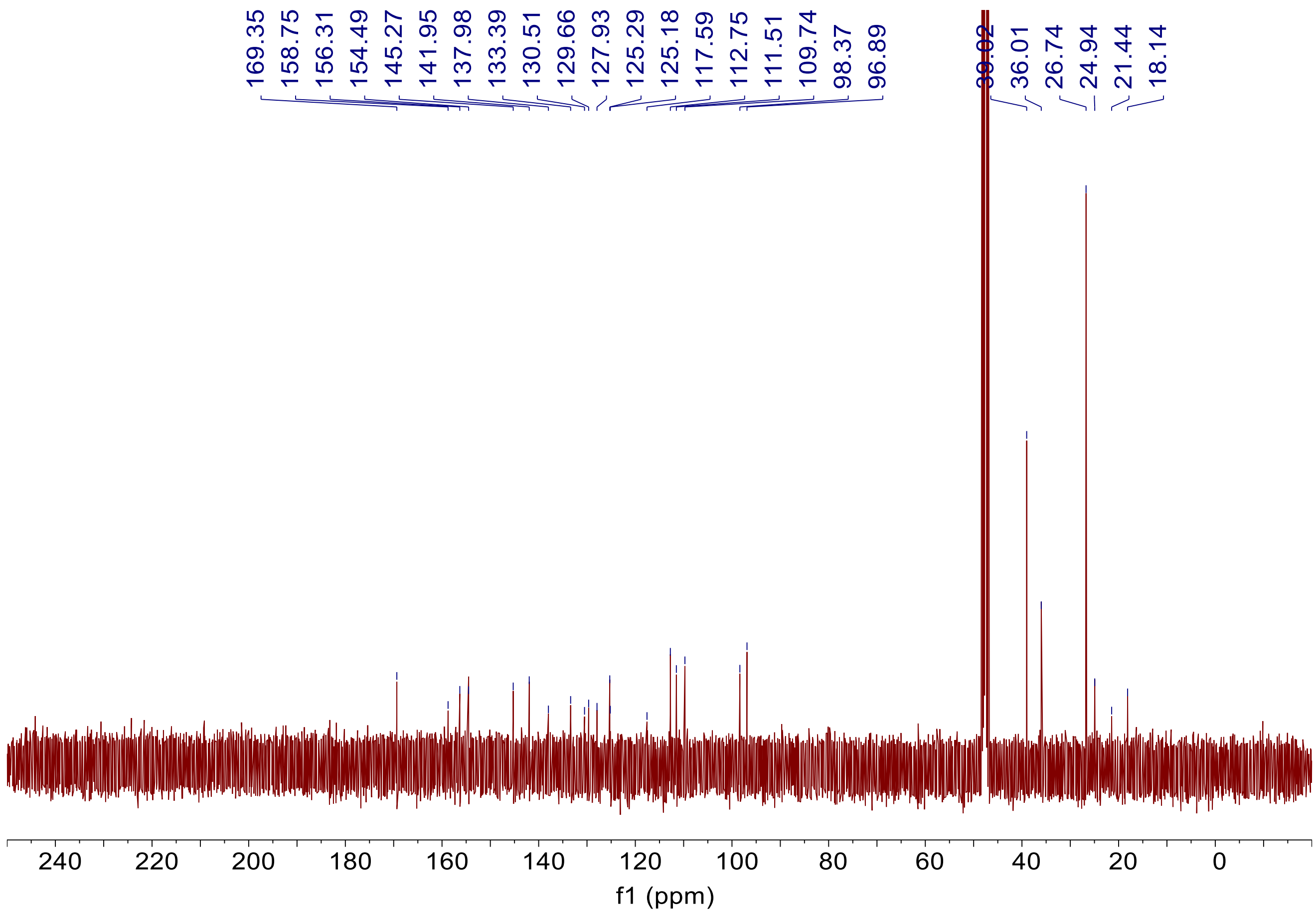
169.35
158.75
156.31
154.49
145.27
141.95
137.98
133.39
130.51
129.66
127.93
125.29
125.18
117.59
112.75
111.51
109.74
98.37
96.89
36.01
26.74
24.94
21.44
18.14
240
220
200
180
160
140
120
100
80
60
40
20
0
f1 (ppm)

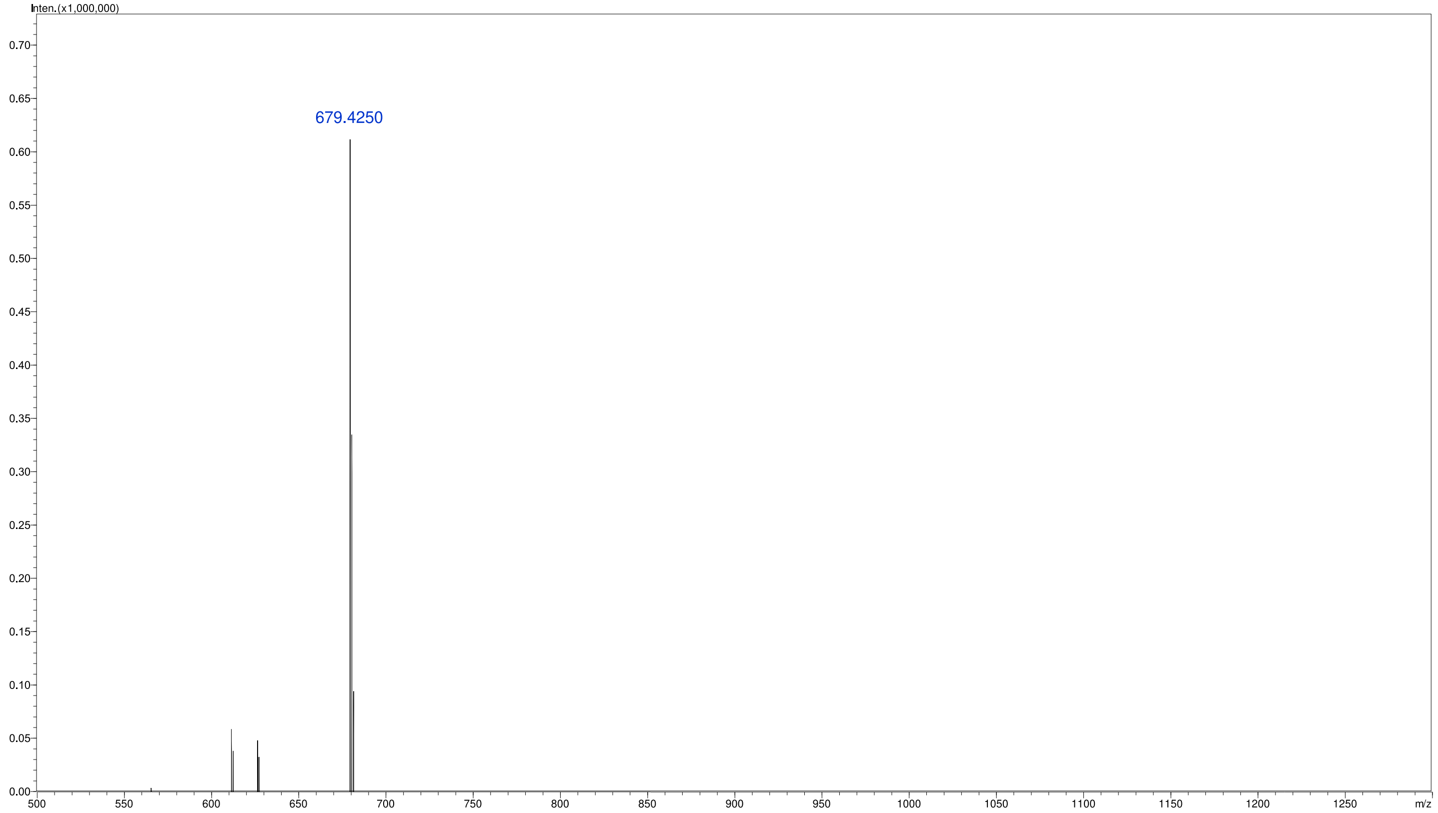
Inten.(x1,000,000)
679.4250
m/z

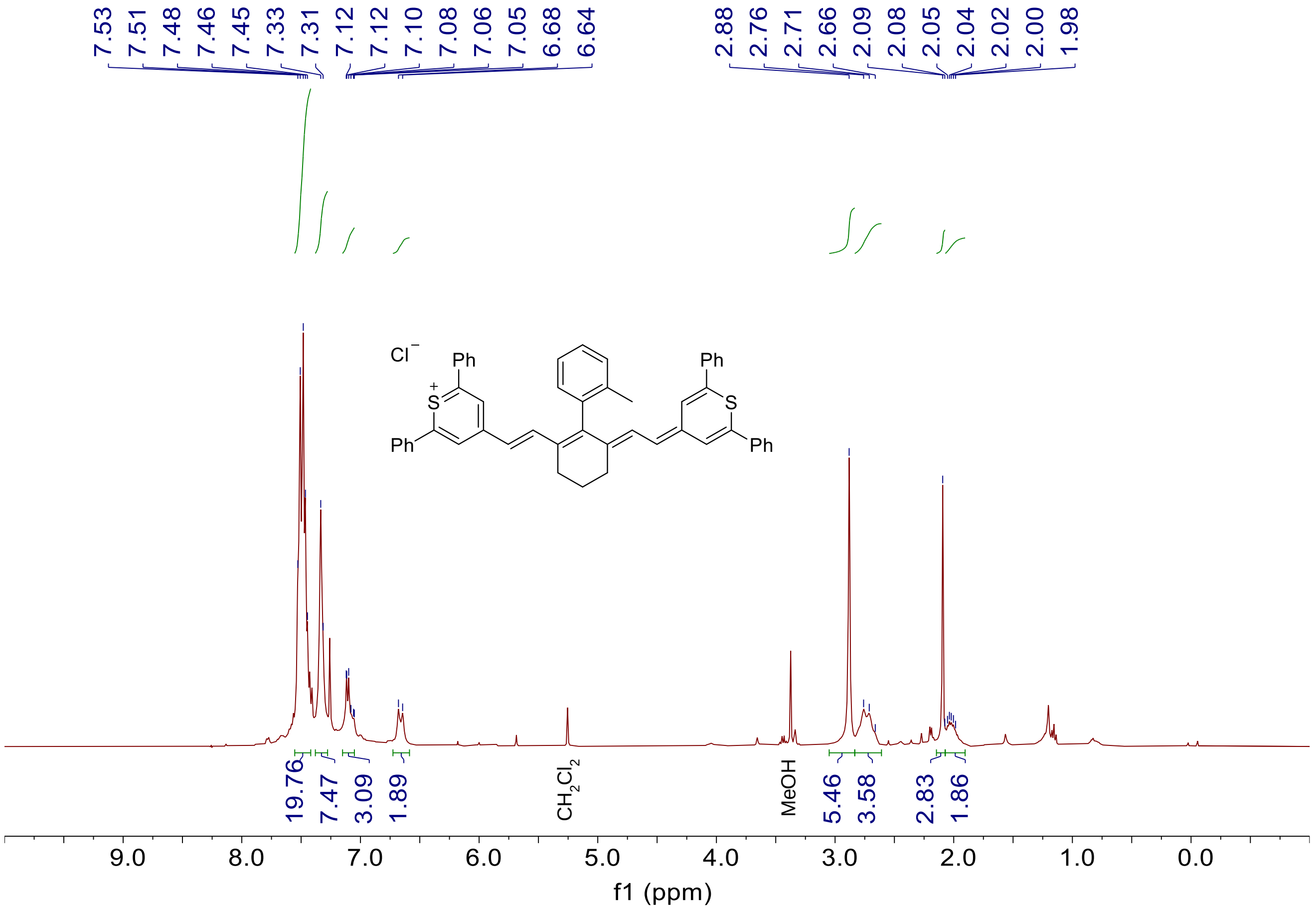
7.53 7.51 7.48 7.46 7.45 7.33 7.31 7.12 7.12 7.10 7.08 7.06 7.05 6.68 6.64
2.88 2.76 2.71 2.66 2.09 2.08 2.05 2.04 2.02 2.00 1.98
Cl⁻
Ph
S
Ph
Ph
S
Ph
19.76 7.47 3.09 1.89
CH2Cl2
MeOH
5.46 3.58
2.83 1.86
9.0 8.0 7.0 6.0 5.0 4.0 3.0 2.0 1.0 0.0
f1 (ppm)

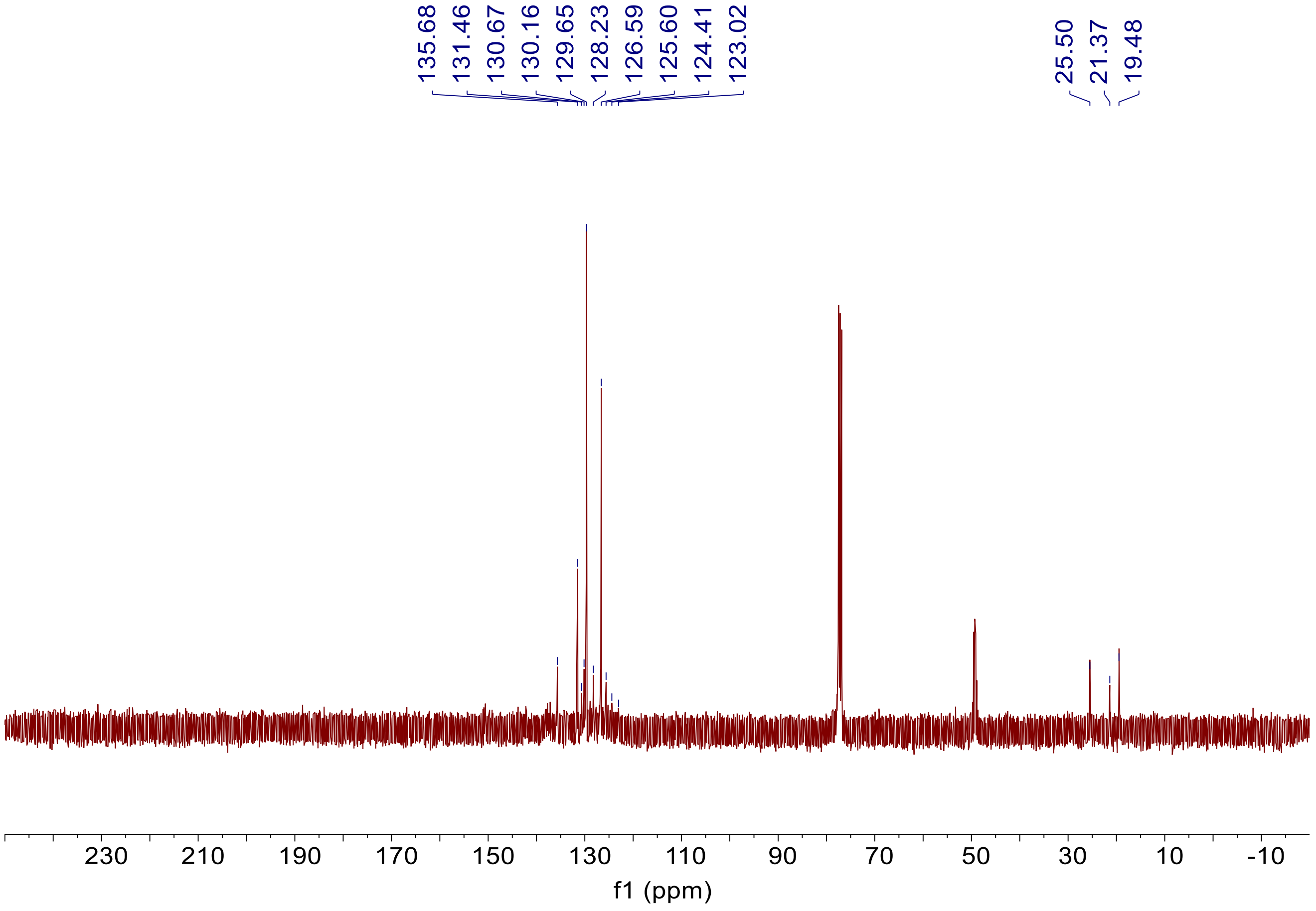
135.68 131.46 130.67 130.16 129.65 128.23 126.59 125.60 124.41 123.02
25.50 21.37 19.48
230 210 190 170 150 130 110 90 70 50 30 10 -10
f1 (ppm)

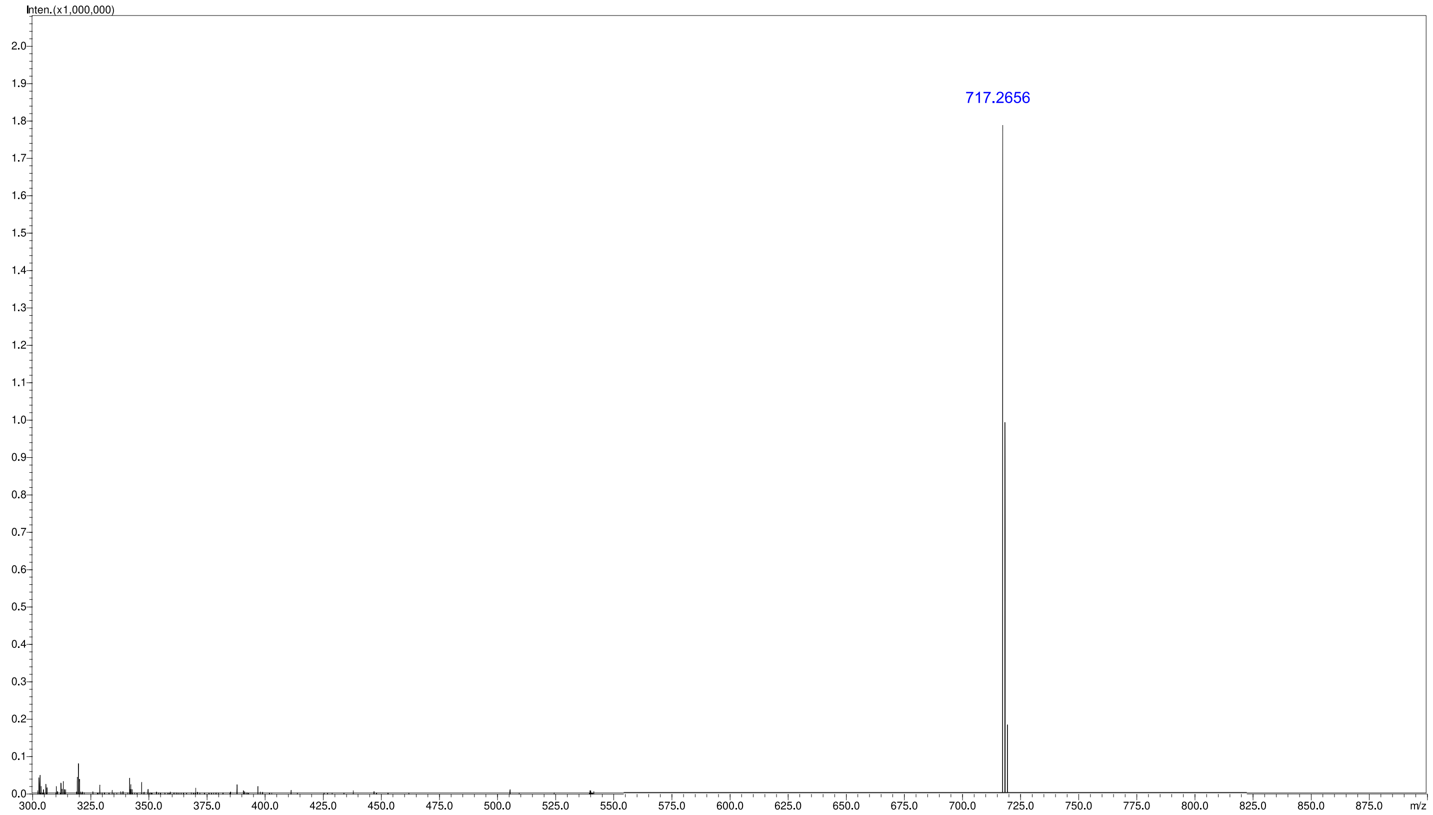
Inten.(x1,000,000)
717.2656
m/z

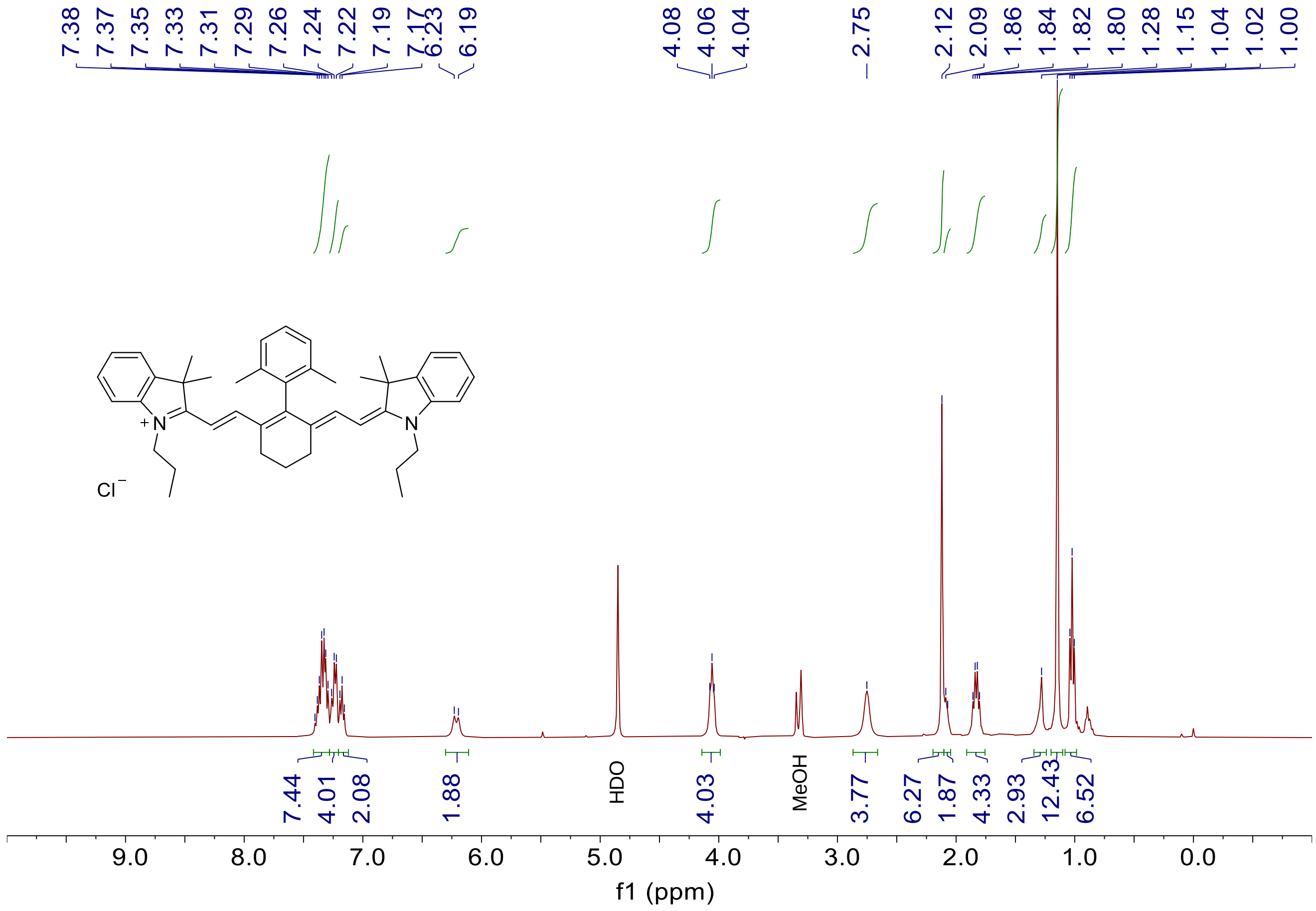
7.38 7.37 7.35 7.33 7.31 7.29 7.26 7.24 7.22 7.19 7.17 6.23 6.19
4.08 4.06 4.04
2.75
2.12 2.09 1.86 1.84 1.82 1.80 1.28 1.15 1.04 1.02 1.00
+N
N
Cl⁻
7.44 4.01 2.08 1.88 HDO 4.03 MeOH 3.77 6.27 1.87 4.33 2.93 12.43 6.52
9.0 8.0 7.0 6.0 5.0 4.0 3.0 2.0 1.0 0.0
f1 (ppm)

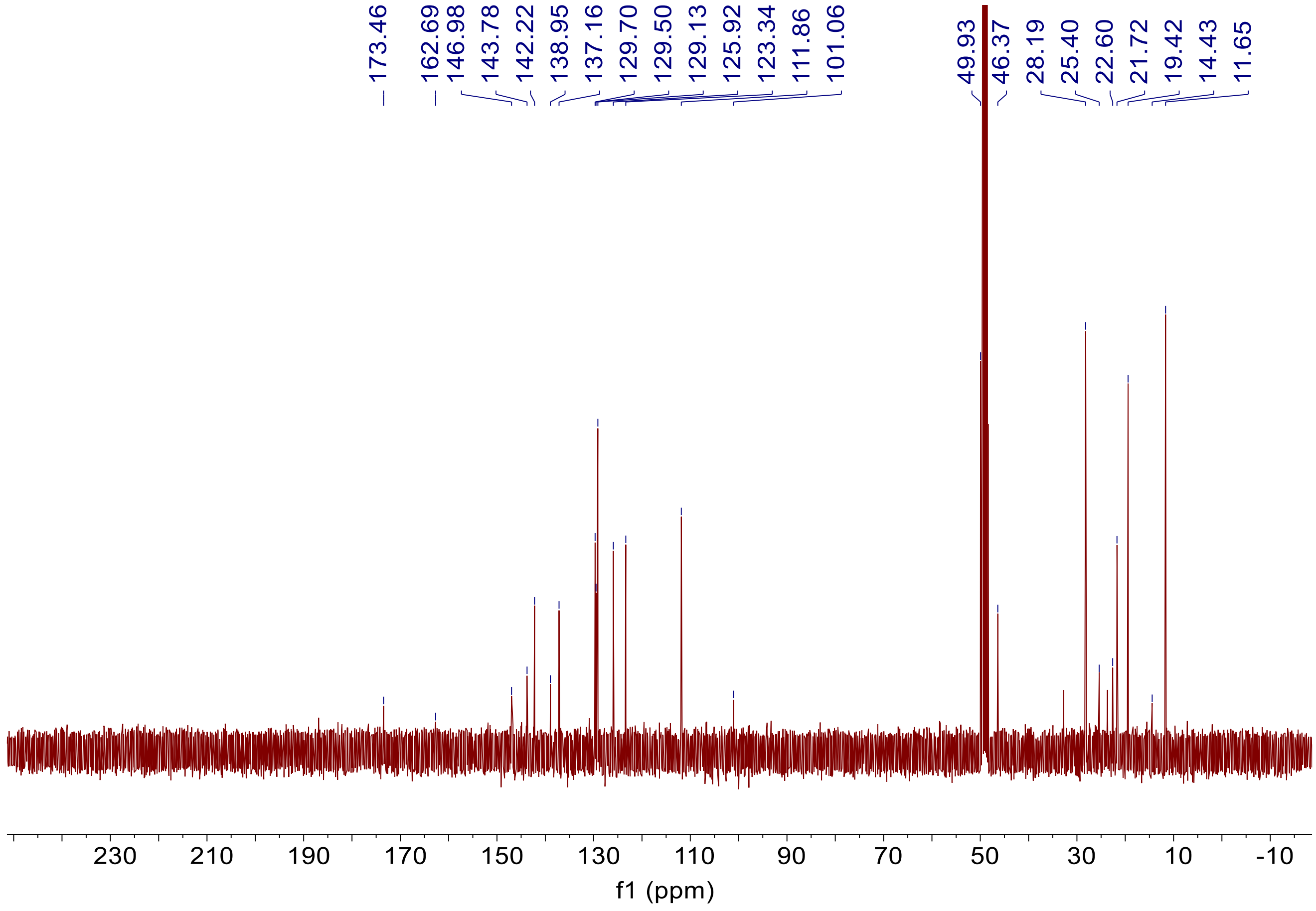
173.46
162.69 146.98 143.78 142.22 138.95 137.16 129.70 129.50 129.13 125.92 123.34 111.86 101.06
49.93 46.37 28.19 25.40 22.60 21.72 19.42 14.43 11.65
230 210 190 170 150 130 110 90 70 50 30 10 -10
f1 (ppm)

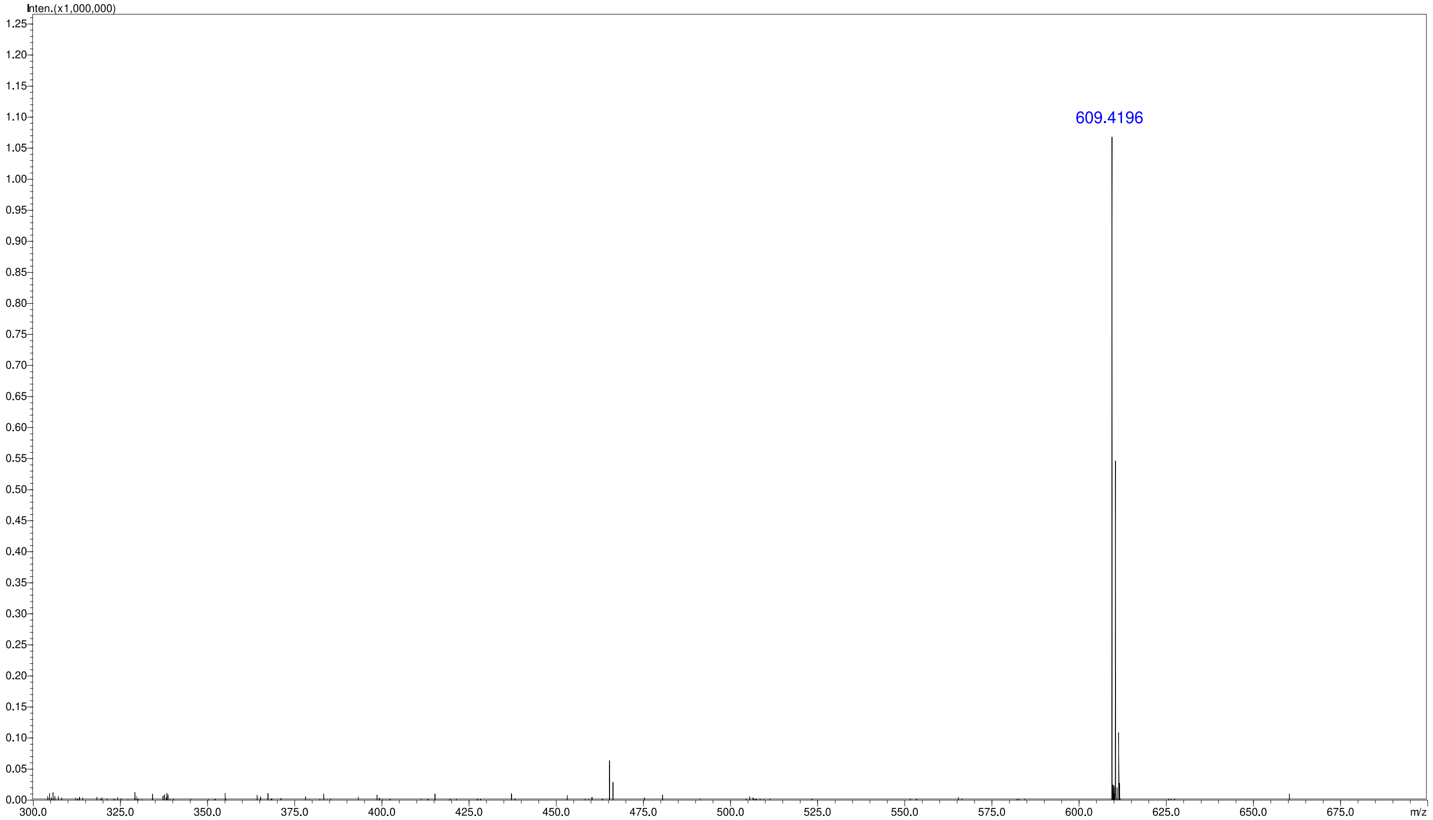
Inten.(x1,000,000)
609.4196
m/z

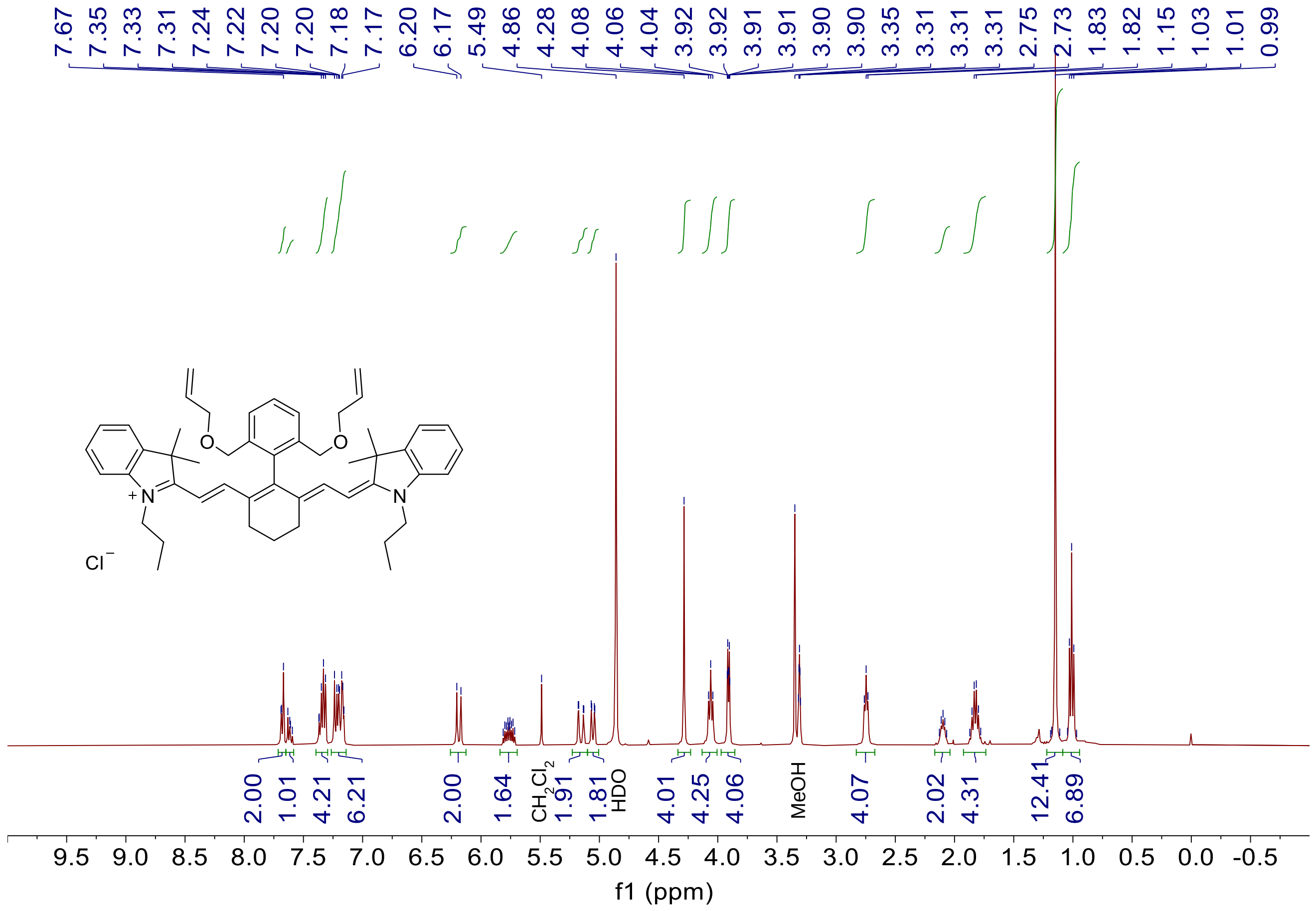
7.67 7.35 7.33 7.31 7.24 7.22 7.20 7.20 7.18 7.17 6.20 6.17 5.49 4.86 4.28 4.08 4.06 4.04 3.92 3.92 3.91 3.91 3.90 3.90 3.35 3.31 3.31 3.31 2.75 2.73 1.83 1.82 1.15 1.03 1.01 0.99
N
+
O
O
N
Cl⁻
2.00 1.01 4.21 6.21 2.00 1.64 CH2Cl2 1.91 1.81 HDO 4.01 4.25 4.06 MeOH 4.07 2.02 4.31 12.41 6.89
9.5 9.0 8.5 8.0 7.5 7.0 6.5 6.0 5.5 5.0 4.5 4.0 3.5 3.0 2.5 2.0 1.5 1.0 0.5 0.0 -0.5
f1 (ppm)

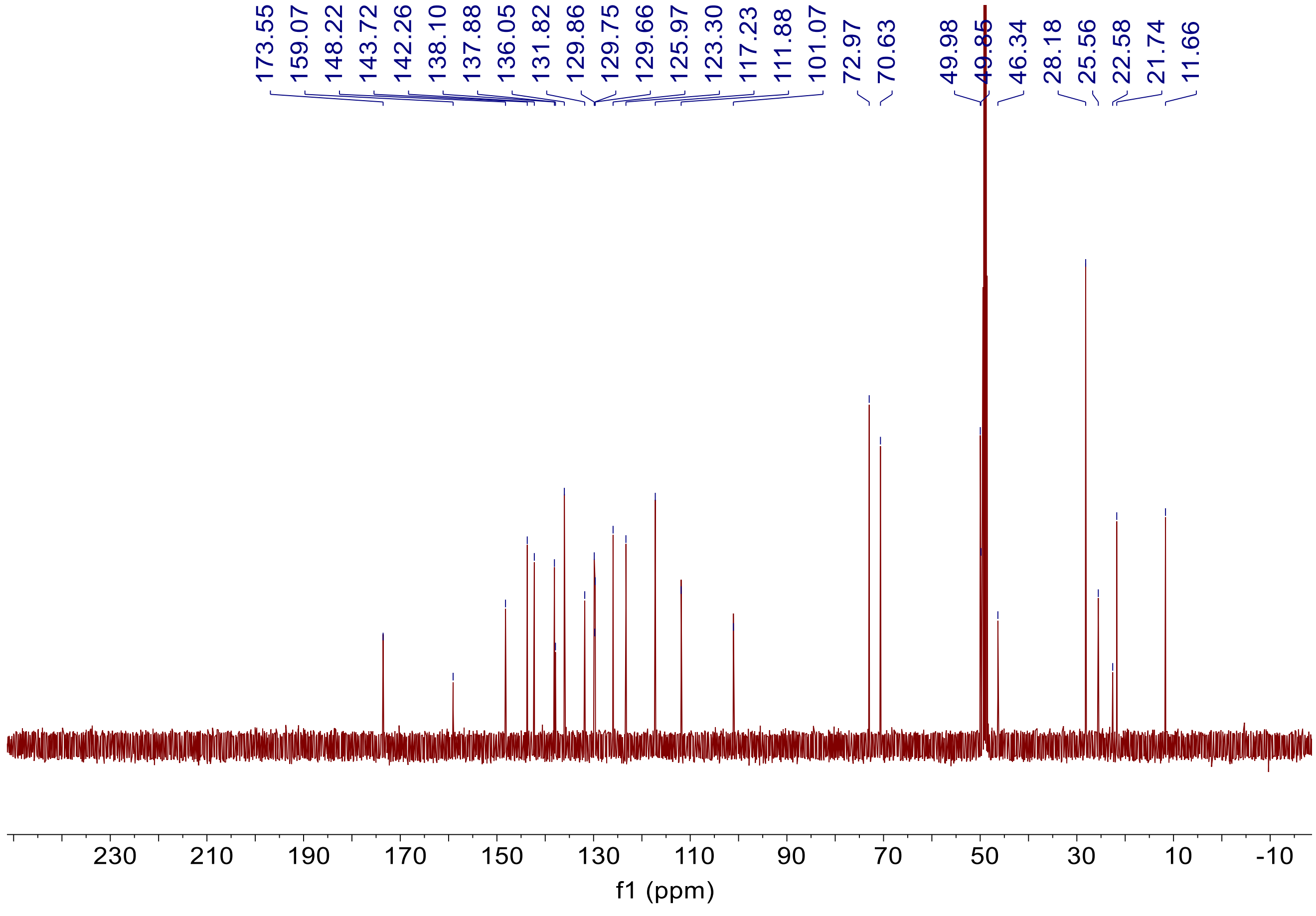
173.55 159.07 148.22 143.72 142.26 138.10 137.88 136.05 131.82 129.86 129.75 129.66 125.97 123.30 117.23 111.88 101.07 72.97 70.63 49.98 49.85 46.34 28.18 25.56 22.58 21.74 11.66
230 210 190 170 150 130 110 90 70 50 30 10 -10
f1 (ppm)

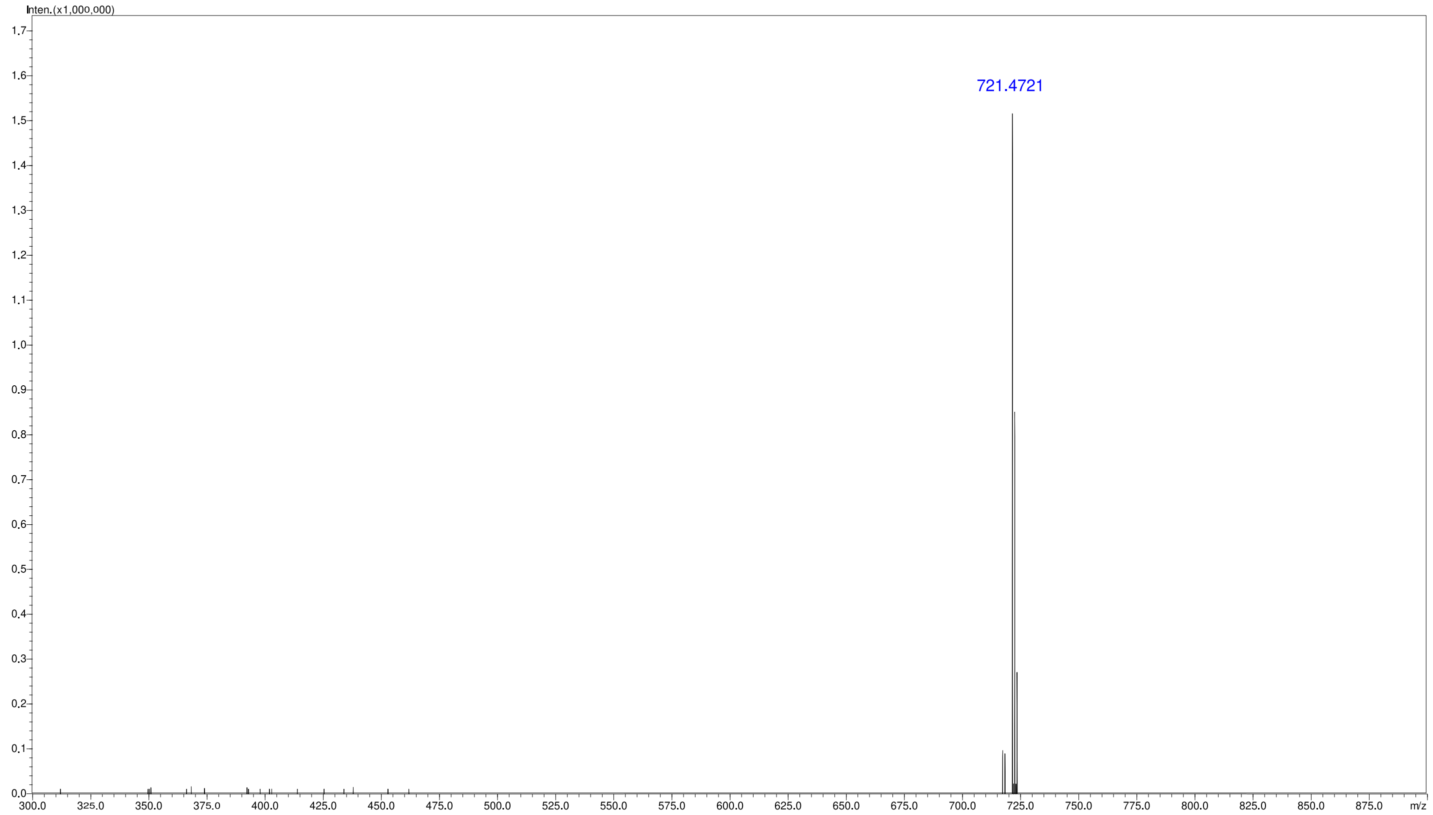

Inten.(x1,000,000)
1.7
1.6
1.5
1.4
1.3
1.2
1.1
1.0
0.9
0.8
0.7
0.6
0.5
0.4
0.3
0.2
0.1
0.0
721.4721
300.0
325.0
350.0
375.0
400.0
425.0
450.0
475.0
500.0
525.0
550.0
575.0
600.0
625.0
650.0
675.0
700.0
725.0
750.0
775.0
800.0
825.0
850.0
875.0
m/z

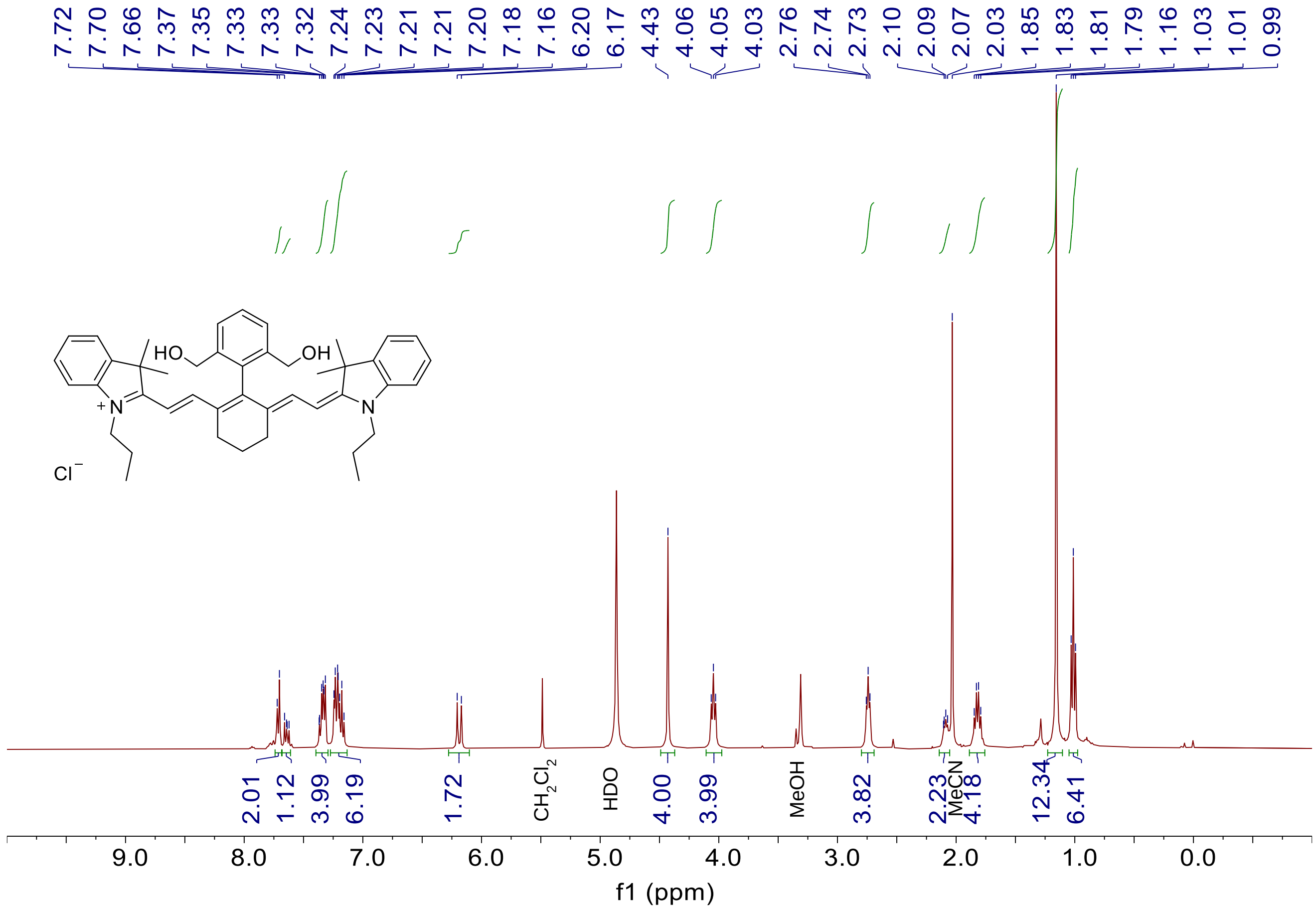
7.72
7.70
7.66
7.37
7.35
7.33
7.33
7.32
7.24
7.23
7.21
7.21
7.20
7.18
7.16
6.20
6.17
4.43
4.06
4.05
4.03
2.76
2.74
2.73
2.10
2.09
2.07
2.03
1.85
1.83
1.81
1.79
1.16
1.03
1.01
0.99
HO
OH
N
N
Cl⁻
2.01
1.12
3.99
6.19
1.72
CH2Cl2
HDO
4.00
3.99
MeOH
3.82
2.23
MeCN
4.18
12.34
6.41
9.0
8.0
7.0
6.0
5.0
4.0
3.0
2.0
1.0
0.0
f1 (ppm)

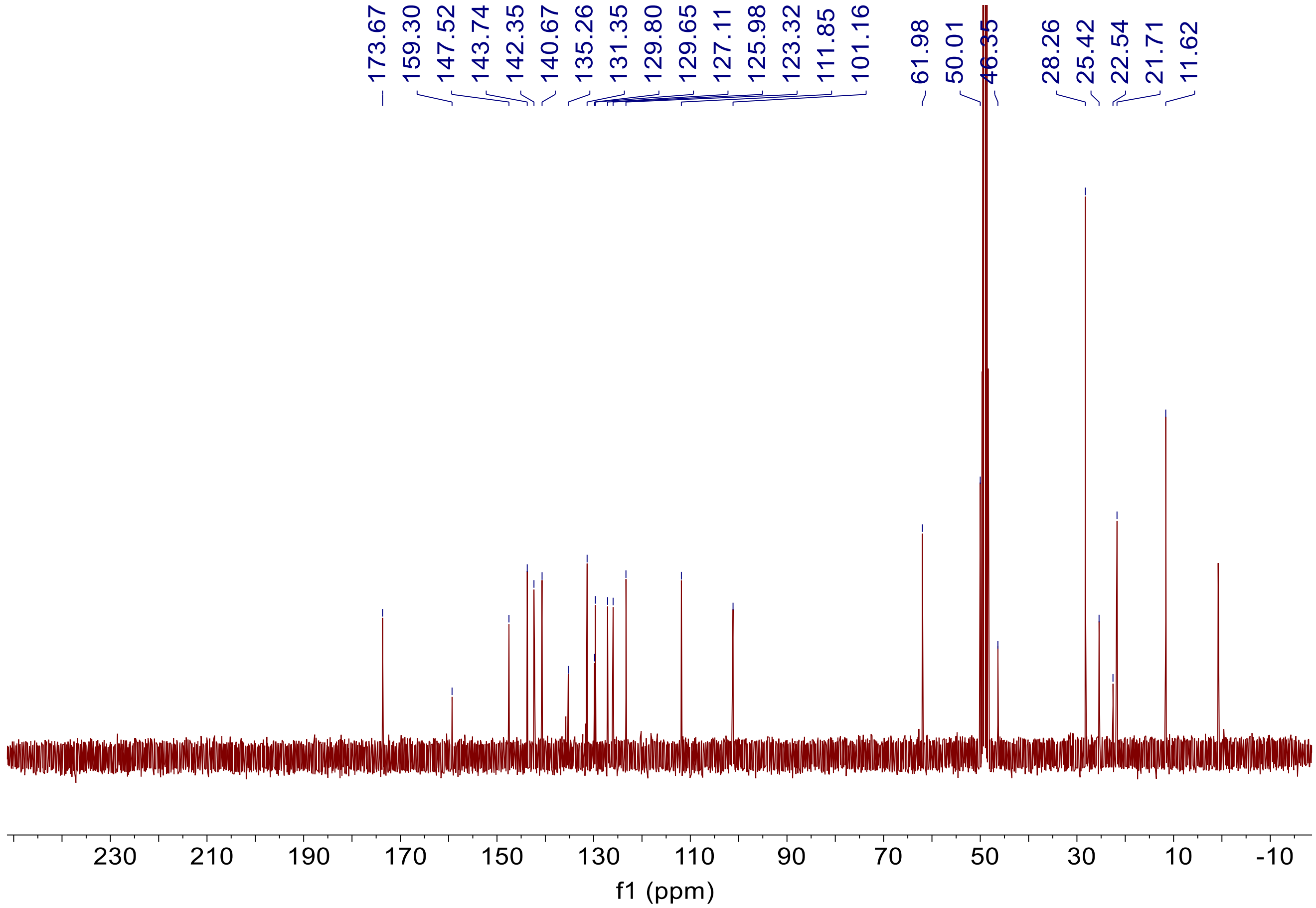
173.67
159.30
147.52
143.74
142.35
140.67
135.26
131.35
129.80
129.65
127.11
125.98
123.32
111.85
101.16
61.98
50.01
46.35
28.26
25.42
22.54
21.71
11.62
230
210
190
170
150
130
110
90
70
50
30
10
-10
f1 (ppm)

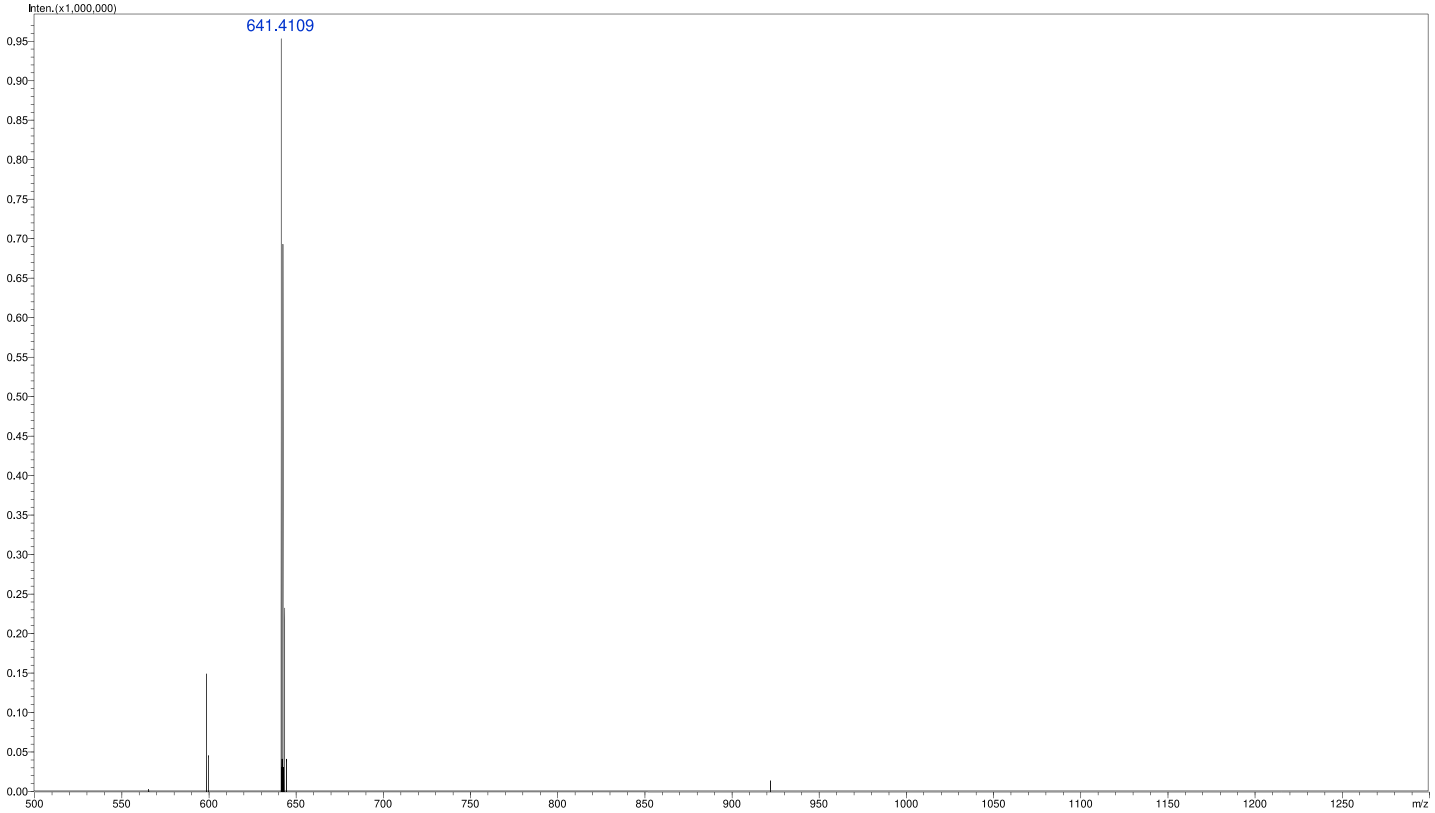
Inten.(x1,000,000)
641.4109
0.95
0.90
0.85
0.80
0.75
0.70
0.65
0.60
0.55
0.50
0.45
0.40
0.35
0.30
0.25
0.20
0.15
0.10
0.05
0.00
500
550
600
650
700
750
800
850
900
950
1000
1050
1100
1150
1200
1250
m/z

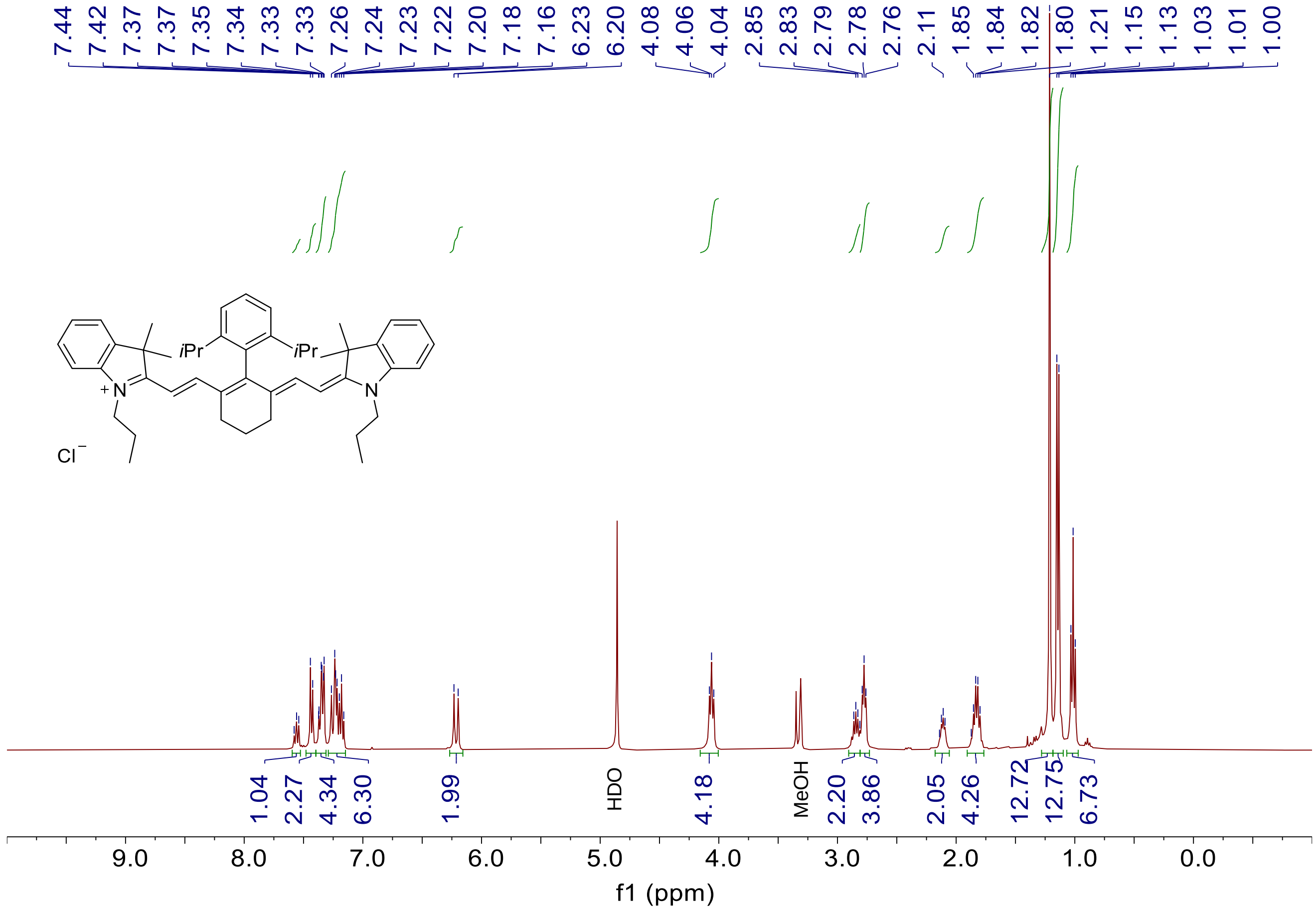
7.44 7.42 7.37 7.37 7.35 7.34 7.33 7.33 7.26 7.24 7.23 7.22 7.20 7.18 7.16 6.23 6.20 4.08 4.06 4.04 2.85 2.83 2.79 2.78 2.76 2.11 1.85 1.84 1.82 1.80 1.21 1.15 1.13 1.03 1.01 1.00
iPr
iPr
N
N
Cl−
1.04 2.27 4.34 6.30 1.99 HDO 4.18 MeOH 2.20 3.86 2.05 4.26 12.72 12.75 6.73
9.0 8.0 7.0 6.0 5.0 4.0 3.0 2.0 1.0 0.0
f1 (ppm)

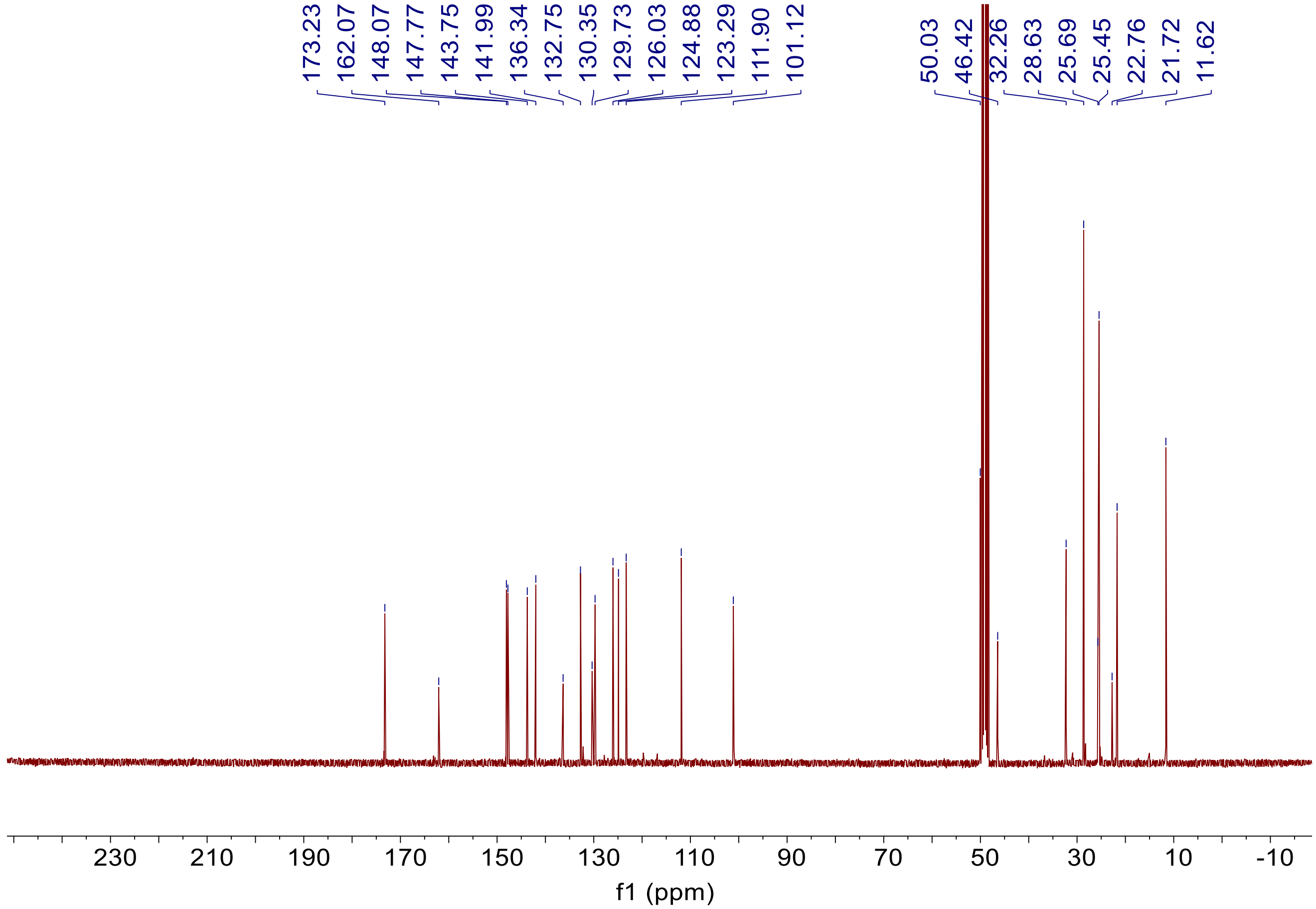
173.23 162.07 148.07 147.77 143.75 141.99 136.34 132.75 130.35 129.73 126.03 124.88 123.29 111.90 101.12
50.03 46.42 32.26 28.63 25.69 25.45 22.76 21.72 11.62
230 210 190 170 150 130 110 90 70 50 30 10 -10
f1 (ppm)

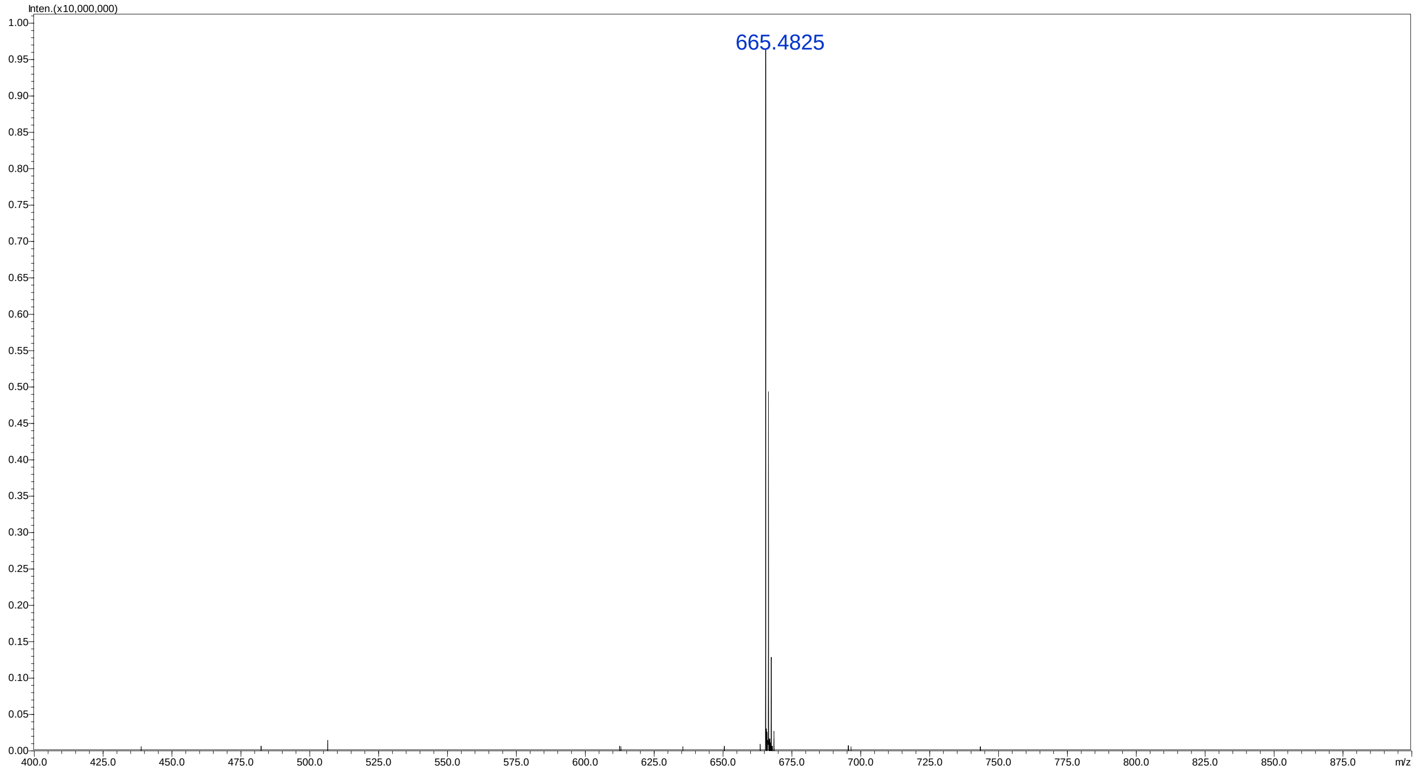
Inten.(x10,000,000)
665.4825
1.00
0.95
0.90
0.85
0.80
0.75
0.70
0.65
0.60
0.55
0.50
0.45
0.40
0.35
0.30
0.25
0.20
0.15
0.10
0.05
0.00
400.0
425.0
450.0
475.0
500.0
525.0
550.0
575.0
600.0
625.0
650.0
675.0
700.0
725.0
750.0
775.0
800.0
825.0
850.0
875.0
m/z

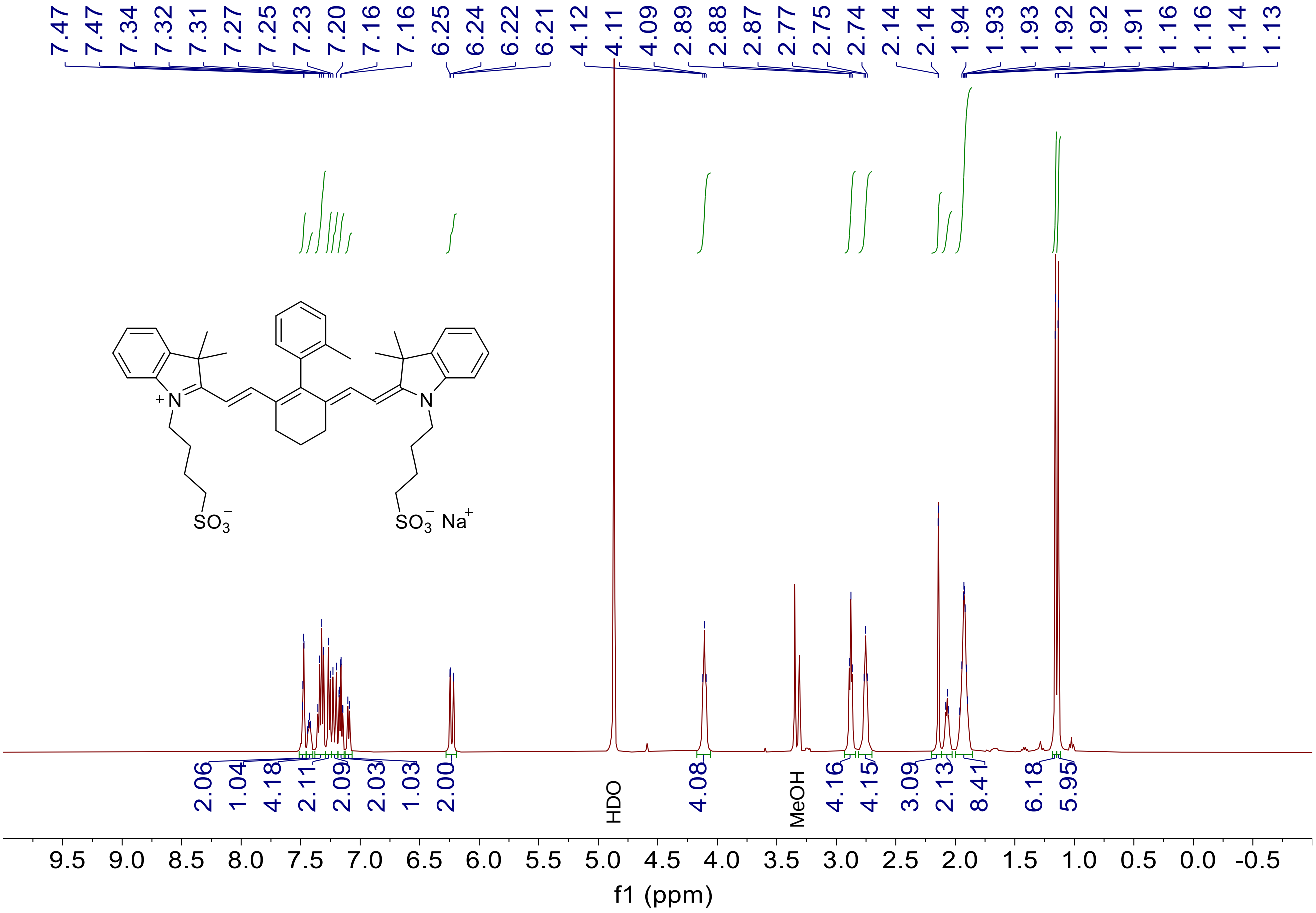
7.47 7.47 7.34 7.32 7.31 7.27 7.25 7.23 7.20 7.16 7.16 6.25 6.24 6.22 6.21 4.12 4.11 4.09 2.89 2.88 2.87 2.77 2.75 2.74 2.14 2.14 1.94 1.93 1.93 1.92 1.92 1.91 1.16 1.16 1.14 1.13
SO_3^-
SO_3^- Na^+
2.06 1.04 4.18 2.11 2.09 2.03 1.03 2.00
HDO
4.08
MeOH
4.16 4.15 3.09 2.13 8.41 6.18 5.95
9.5 9.0 8.5 8.0 7.5 7.0 6.5 6.0 5.5 5.0 4.5 4.0 3.5 3.0 2.5 2.0 1.5 1.0 0.5 0.0 -0.5
f1 (ppm)

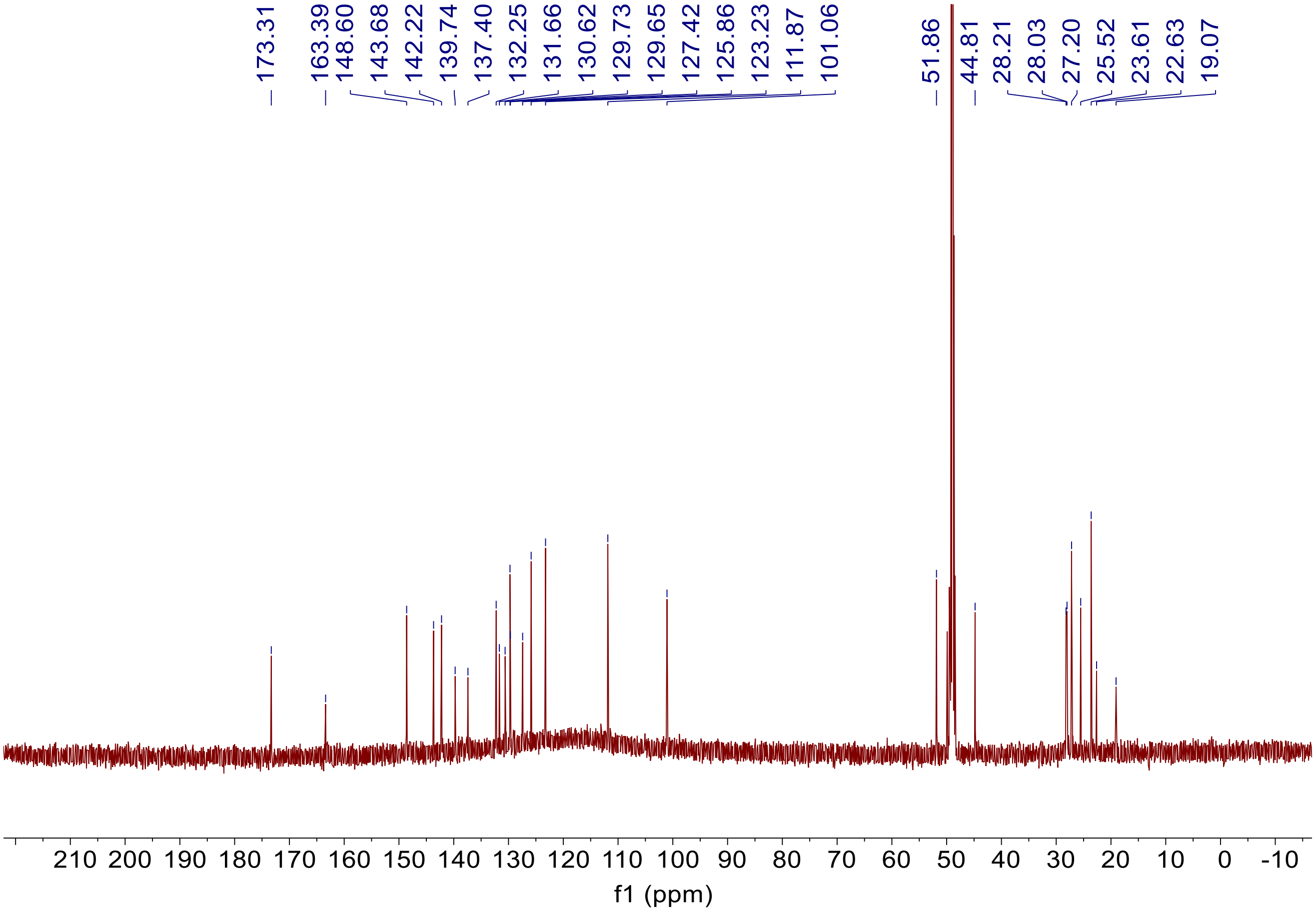
173.31
163.39 148.60 143.68 142.22 139.74 137.40 132.25 131.66 130.62 129.73 129.65 127.42 125.86 123.23 111.87 101.06
51.86
44.81
28.21 28.03 27.20 25.52 23.61 22.63 19.07
210 200 190 180 170 160 150 140 130 120 110 100 90 80 70 60 50 40 30 20 10 0 -10
f1 (ppm)

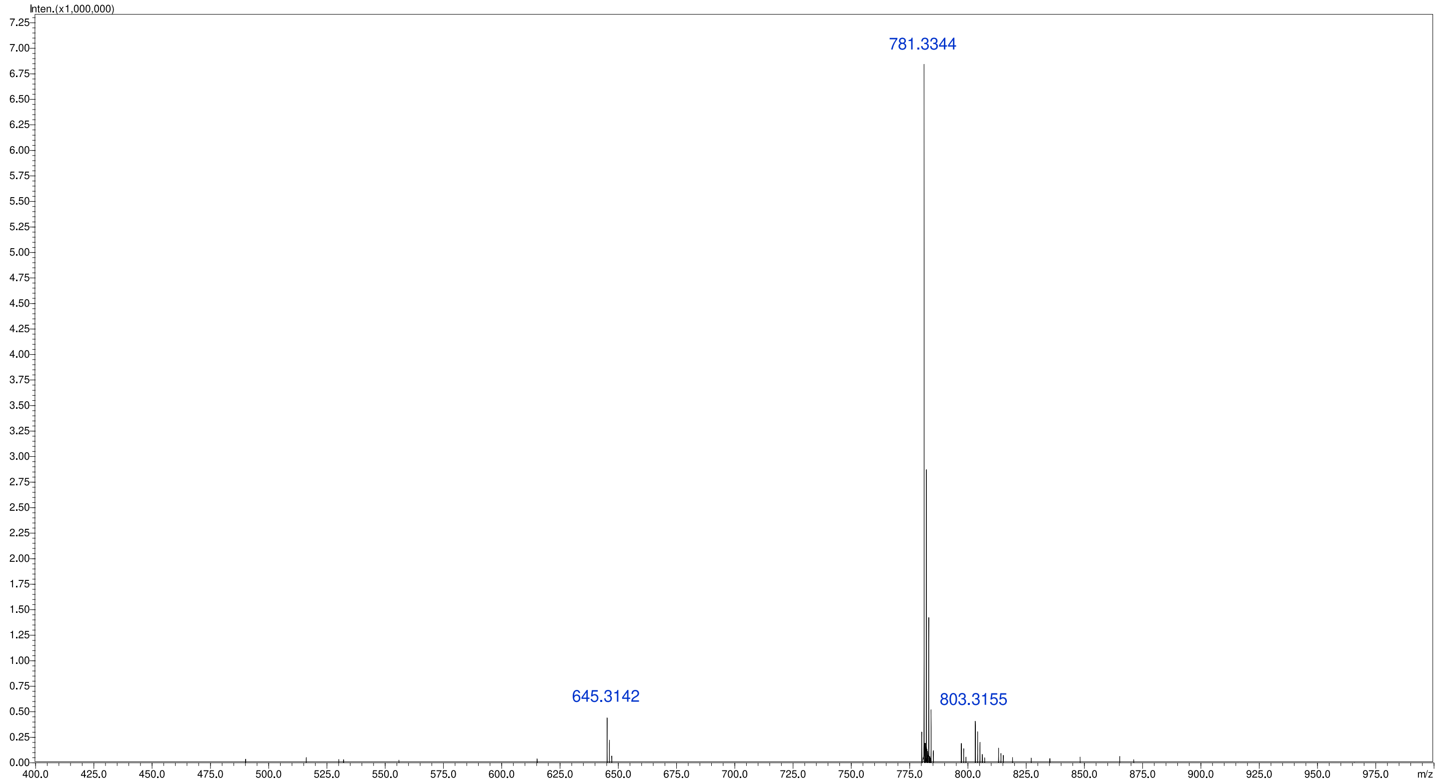
Inten.(x1,000,000)
781.3344
645.3142
803.3155
m/z

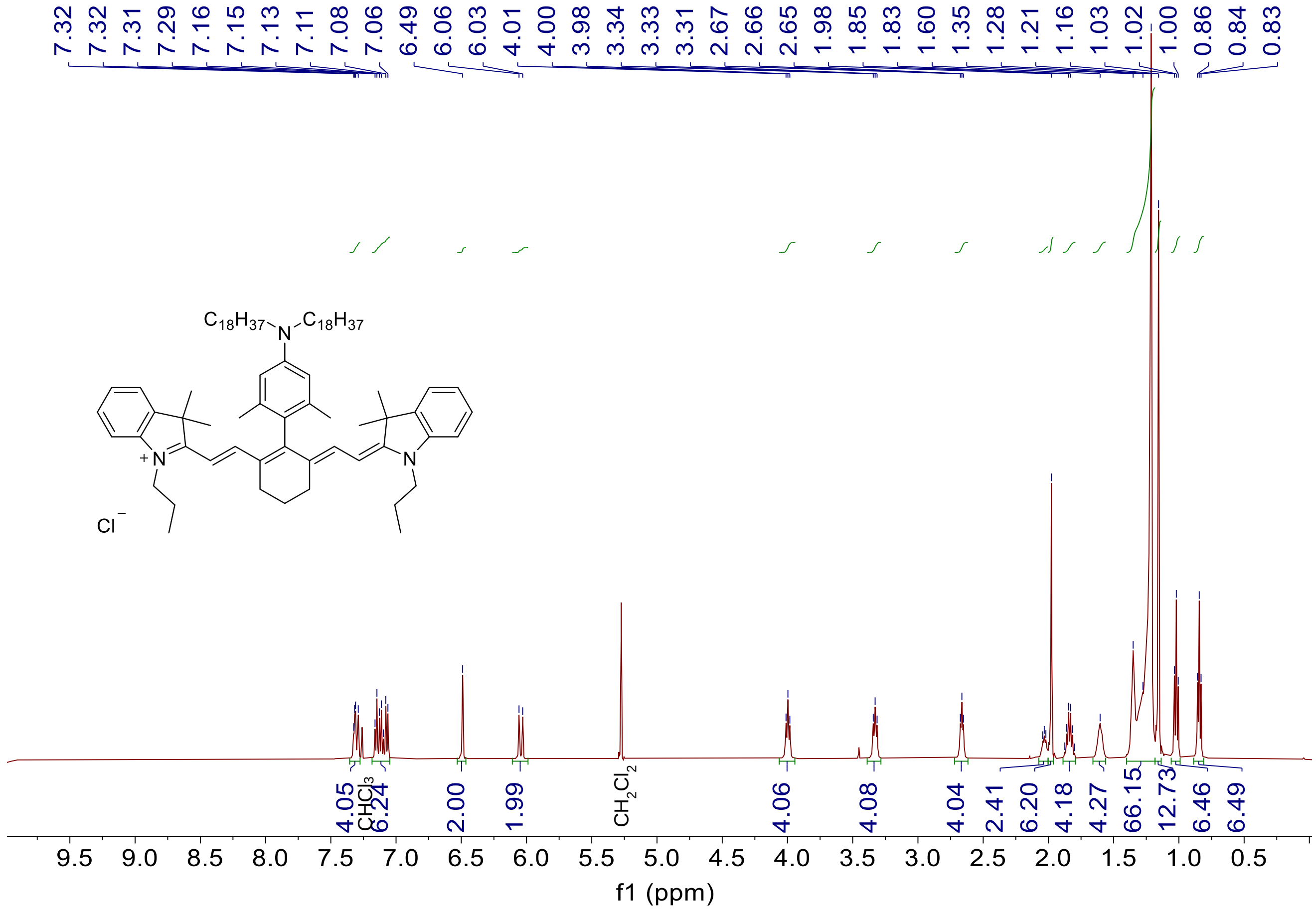
7.32 7.32 7.31 7.29 7.16 7.15 7.13 7.11 7.08 7.06 6.49 6.06 6.03 4.01 4.00 3.98 3.34 3.33 3.31 2.67 2.66 2.65 1.98 1.85 1.83 1.60 1.35 1.28 1.21 1.16 1.03 1.02 1.00 0.86 0.84 0.83
$C_{18}H_{37}$ N $C_{18}H_{37}$
N
N
Cl
4.05 CHCl3 6.24 2.00 1.99 CH2Cl2 4.06 4.08 4.04 2.41 6.20 4.18 4.27 66.15 12.73 6.46 6.49
9.5 9.0 8.5 8.0 7.5 7.0 6.5 6.0 5.5 5.0 4.5 4.0 3.5 3.0 2.5 2.0 1.5 1.0 0.5
f1 (ppm)

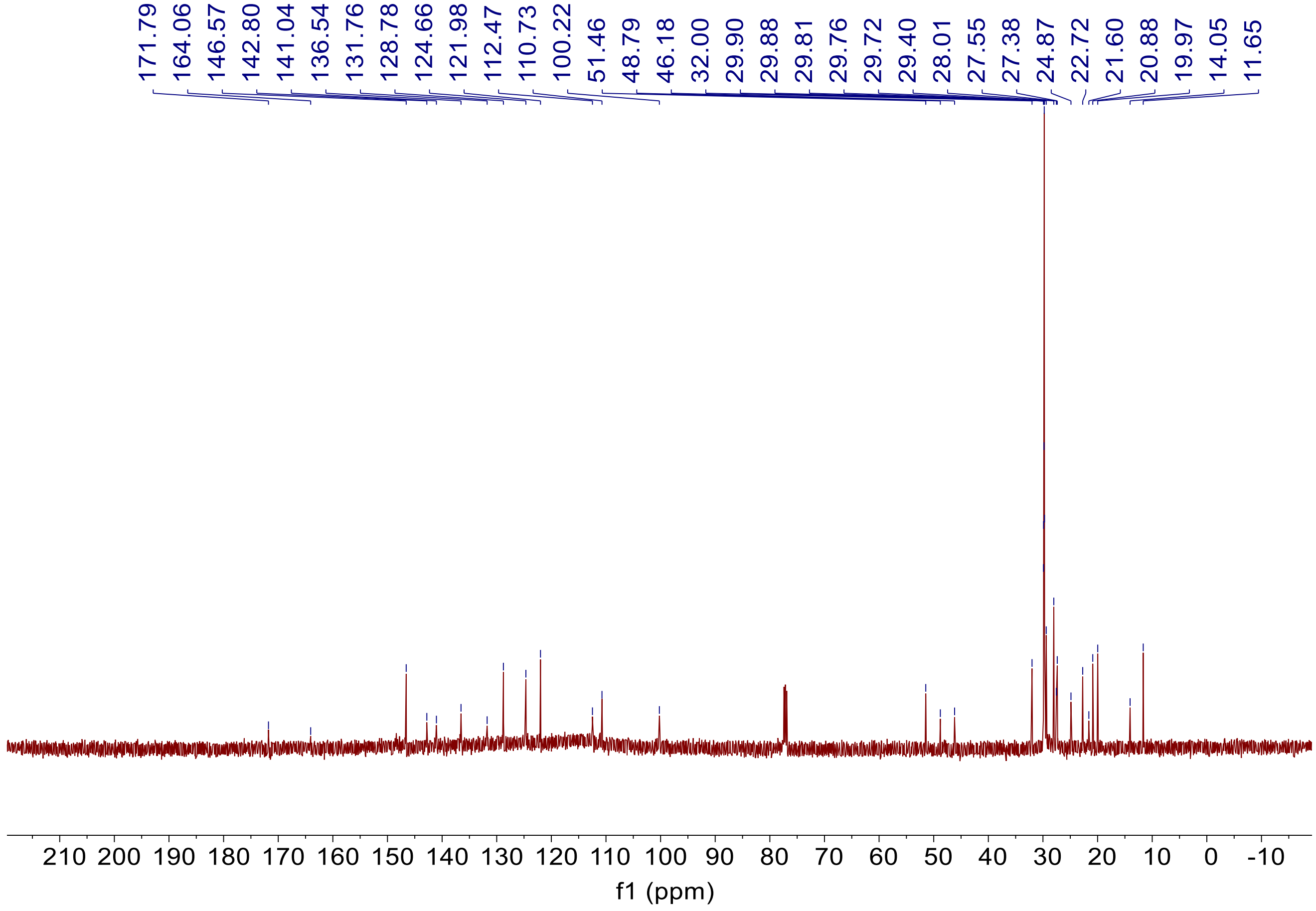
171.79 164.06 146.57 142.80 141.04 136.54 131.76 128.78 124.66 121.98 112.47 110.73 100.22 51.46 48.79 46.18 32.00 29.90 29.88 29.81 29.76 29.72 29.40 28.01 27.55 27.38 24.87 22.72 21.60 20.88 19.97 14.05 11.65
210 200 190 180 170 160 150 140 130 120 110 100 90 80 70 60 50 40 30 20 10 0 -10
f1 (ppm)

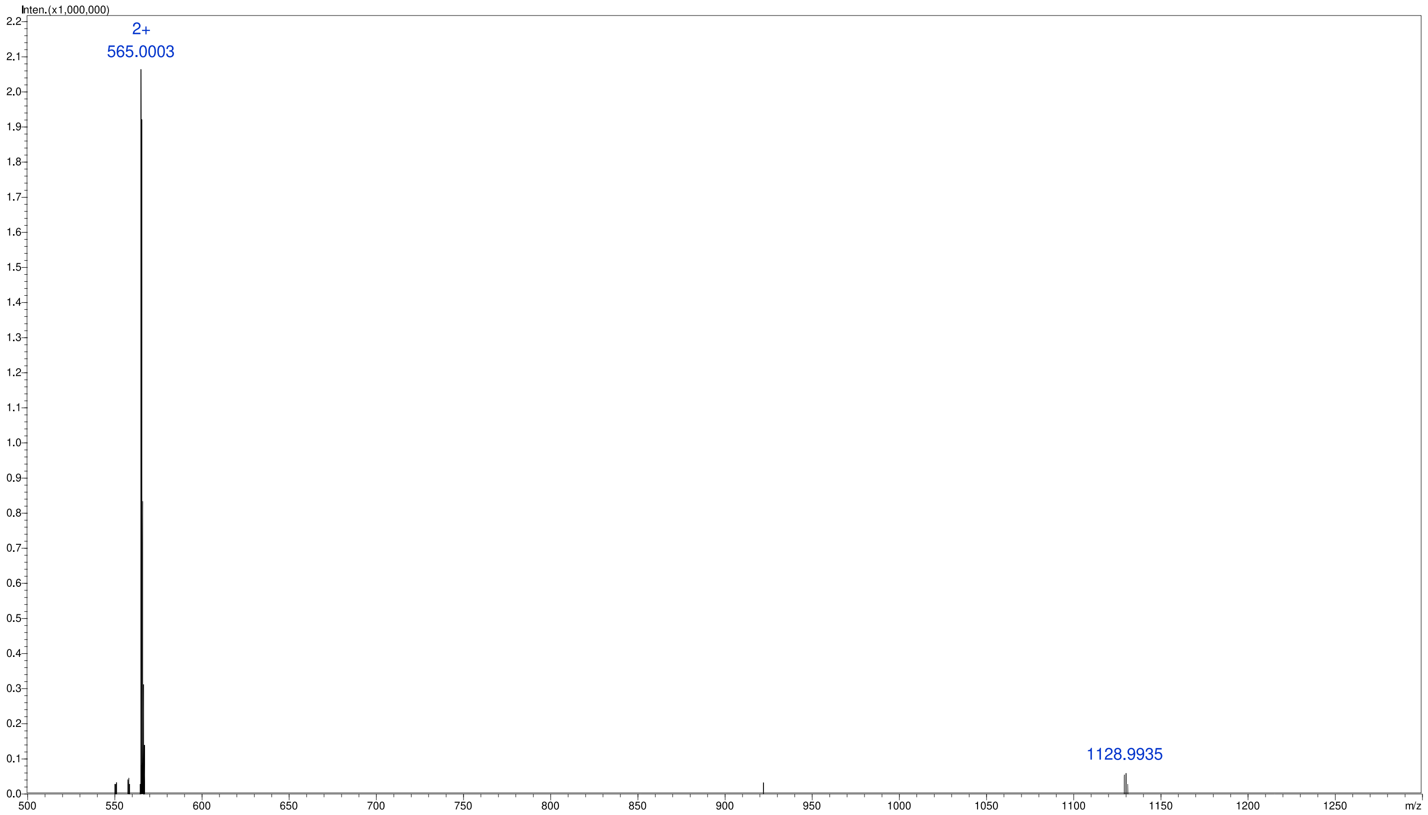
Inten.(x1,000,000)
2+
565.0003
1128.9935
m/z

References

[1] H. Lee, J. C. Mason, S. Achilefu, *J. Org. Chem.* **2008**, *73*, 723–725.
[2] A. Levitz, F. Marmarchi, M. Henary, *Photochem. Photobiol. Sci.* **2018**, *17*, 1409–1416.
[3] L. Štacková, P. Štacko, P. Klán, *J. Am. Chem. Soc.* **2019**, *141*, 7155–7162.
[4] S. M. Usama, S. C. Marker, D.-H. Li, D. R. Caldwell, M. Stroet, N. L. Patel, A. G. Tebo, S. Hernot, J. D. Kalen, M. Schnermann, *J. Am. Chem. Soc.* **2023**, *145*, 14647–14659.
[5] K. Rurack, M. Spieles, *Anal. Chem.* **2011**, *83*, 1232–1242.
[6] E. D. Cosco, I. Lim, E. M. Sletten, *ChemPhotoChem* **2021**, *5*, 727–734.
[7] E. D. Cosco, B. A. Arús, A. L. Spearman, T. L. Atallah, I. Lim, O. S. Leland, J. R. Caram, T. S. Bischof, O. T. Bruns, E. M. Sletten, *J. Am. Chem. Soc.* **2021**, *143*, 6836–6846.
[8] J. Spencer, M. Pfeffer, A. DeCian, J. Fischer, *J. Org. Chem.* **1995**, *60*, 1005–1012.
[9] L. Ye, D. Ding, Y. Feng, D. Xie, P. Wu, H. Guo, Q. Meng, H. Zhou, *Tetrahedron* **2009**, *65*, 8738–8744.
[10] I. Mohammadpoor-Baltork, M. Moghadam, S. Tangestaninejad, V. Mirkhani, A. R. Khosropour, A. Mirjafari, *J. Iran. Chem. Soc.* **2011**, *8*, 513–524.
[11] Lucjan Strekowski, J. Christian Mason, Martial Say, Hyeran Lee, Rajni Gupta, Maryam Hojjat, *Heterocycl. Commun.* **2005**, *11*, 129–134.
[12] S. Pascal, S. Denis-Quanquin, F. Appaix, A. Duperray, A. Grichine, B. Le Guennic, D. Jacquemin, J. Cuny, S.-H. Chi, J. W. Perry, B. van der Sanden, C. Monnereau, C. Andraud, O. Maury, *Chem. Sci.* **2017**, *8*, 381–394.
